# Microphthalmia in Texel Sheep Is Associated with a Missense Mutation in the Paired-Like Homeodomain 3 (*PITX3*) Gene

**DOI:** 10.1371/journal.pone.0008689

**Published:** 2010-01-13

**Authors:** Doreen Becker, Jens Tetens, Adrian Brunner, Daniela Bürstel, Martin Ganter, James Kijas, for the International Sheep Genomics Consortium, Cord Drögemüller

**Affiliations:** 1 Institute of Genetics, Vetsuisse Faculty, University of Berne, Berne, Switzerland; 2 Institute for Animal Breeding and Husbandry, Christian-Albrechts-University Kiel, Kiel, Germany; 3 Clinic for Pigs and Small Ruminants, Forensic Medicine and Ambulatory Service Small Animals, University of Veterinary Medicine Hannover, Hannover, Germany; 4 Commonwealth Scientific and Industrial Research Organisation Livestock Industries, St Lucia, Brisbane, Queensland, Australia; University of Florida, United States of America

## Abstract

Microphthalmia in sheep is an autosomal recessive inherited congenital anomaly found within the Texel breed. It is characterized by extremely small or absent eyes and affected lambs are absolutely blind. For the first time, we use a genome-wide ovine SNP array for positional cloning of a Mendelian trait in sheep. Genotyping 23 cases and 23 controls using Illumina's OvineSNP50 BeadChip allowed us to localize the causative mutation for microphthalmia to a 2.4 Mb interval on sheep chromosome 22 by association and homozygosity mapping. The *PITX3* gene is located within this interval and encodes a homeodomain-containing transcription factor involved in vertebrate lens formation. An abnormal development of the lens vesicle was shown to be the primary event in ovine microphthalmia. Therefore, we considered *PITX3* a positional and functional candidate gene. An ovine BAC clone was sequenced, and after full-length cDNA cloning the *PITX3* gene was annotated. Here we show that the ovine microphthalmia phenotype is perfectly associated with a missense mutation (c.338G>C, p.R113P) in the evolutionary conserved homeodomain of *PITX3*. Selection against this candidate causative mutation can now be used to eliminate microphthalmia from Texel sheep in production systems. Furthermore, the identification of a naturally occurring *PITX3* mutation offers the opportunity to use the Texel as a genetically characterized large animal model for human microphthalmia.

## Introduction

Human microphthalmia, characterized by small eyes and other ocular abnormalities in newborns, is highly variable with the most severe cases anophthalmic [1], [2]. Anophthalmia and microphthalmia cause congenital blindness and affect up to 30 per 100,000 people worldwide [2]. Both anophthalmia and microphthalmia may occur in isolation or as part of a syndrome, as in one-third of cases [2]. Morphological studies showed that impaired lens formation seems to be the major cause of anophthalmia and microphthalmia, although the precise pathogenesis of these phenotypes remains unknown [3]. Lens development is a critical embryonic period in vertebrate eye development during which many inductive signals are exchanged between the optic vesicle and surface ectoderm [1], [3]. This stage is characterized by formation of the lens placode, a thickening of the surface ectoderm that comes into contact with the optic vesicle [1], [3]. Coordinated invagination of the lens placode and the optic vesicle results in the formation of the lens vesicle and a double-layered optic cup and provides the first indication of the final shape of the eye [1], [3]. Genetic studies have identified some of the critical determinants of eye formation. A set of putative transcription factors required for the earliest step of eye development were identified in *Drosophila*. The involvement of homologous proteins in vertebrate lens development was subsequently elucidated by the characterization of mutations that cause congenital human or murine ocular disorders and their comparison to mutations in model organisms [1]. Analyzing inherited isolated microphthalmia/anophthalmia in humans revealed a total of eight genes (*SOX2, PAX6, OTX2, RAX, CHX10, FOXE3, PITX3, CRYBA4*) carrying causative mutations [4]–[11]. For some human non-syndromic microphthalmia cases the underlying mutation has not yet been found [2]. The role of the eight genes during lens development was confirmed by studying spontaneous mouse mutants and genetically engineered mice with more or less similar ocular phenotypes as in human [12]. Besides *CRYBA4*, encoding a lens specific structural protein, seven of these genes encode transcription factors which are required for appropriate lens formation during eye development [1].

**Figure 1:**
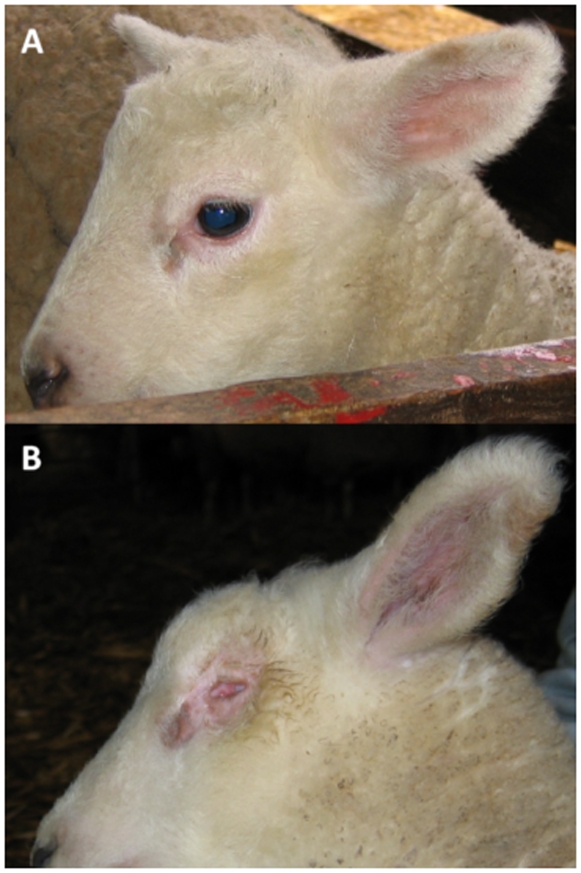
Microphthalmia phenotype in Texel sheep. (**A**) Normal newborn lamb. (**B**) Microphthalmia affected lamb.

Isolated congenital microphthalmia occurs in various mammalian species [1], [3], [13] including the Texel breed of sheep (Figure 1) [14]–[20]. In Texel sheep microphthalmia behaves as a monogenic autosomal recessive trait [16], [20]. An abnormal development of the lens vesicle was shown to be the primary event [19], but so far the underlying genetic defect has not been elucidated. In an initial analysis, we performed a partial genome scan and observed genetic linkage to microsatellite markers on sheep chromosome 23 [21]. After significant extension of the available material we were not able to confirm these results.

Therefore, we hypothesized that a more comprehensive genome-wide mapping strategy may lead to the identification of the microphthalmia locus in Texel sheep. The ability to assay 50,000 evenly spaced SNP across the sheep genome was recently made possible by development of Illumina's OvineSNP50 BeadChip. This study demonstrates the effectiveness of using this SNP array for the finescale mapping of an inherited defect when testing only a modest number of cases and controls.

## Results

### Sample Collection

We collected samples from 134 microphthalmia affected lambs and 269 unaffected sheep from different sheep farms with Texel purebred or Texel/Whiteheaded mutton crossbred sheep, and from our experimentally established pedigree [20], [21]. Parents of affected offspring were classified as obligate carriers (n = 133). Partial pedigree records were available and allowed us to establish 73 two-generation spanning sheep families segregating for microphthalmia containing 254 of the individuals collected (Figure S1). Due to incomplete pedigree records, it was impossible to determine if affected sheep from the Texel breeding population share common ancestors and trace back to a single common founder. Besides the experimental mating of an affected male to known disease carriers [20], [21], the parents of all available cases were healthy (Figure S1). The pedigrees were consistent with a monogenic autosomal recessive inheritance.

### Mapping of the Causative Mutation

**Figure 2:**
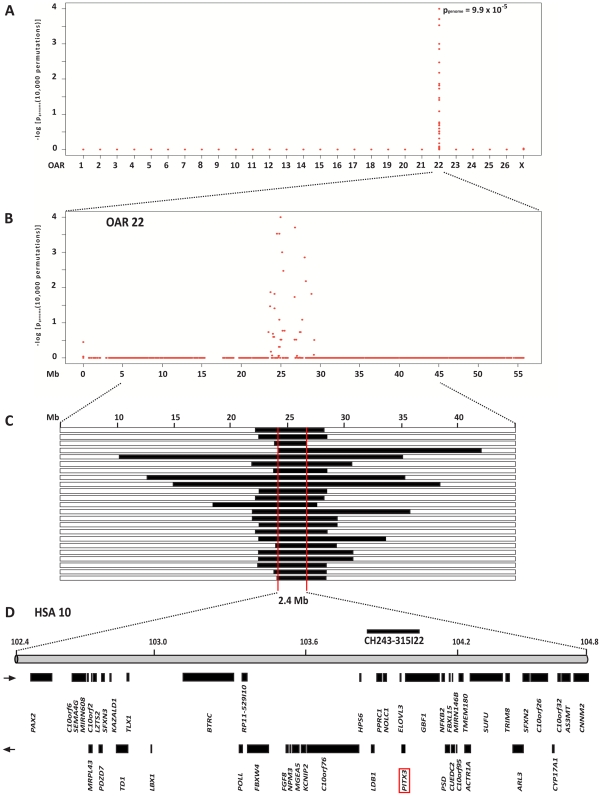
Genome-wide association mapping of microphthalmia. (**A**) Case-control whole genome association analysis finds significant association to SNPs on chromosome 22. (**B**) Single SNP association statistic across OAR 22. (**C**) Homozygosity mapping of the microphthalmia mutation. The analysis of SNP genotypes from affected sheep indicated that they had extended overlapping homozygous regions on OAR 22 (indicated as black blocks). Thus – assuming that it resides on the common haplotype block – the causative mutation is located within a 2.4 Mb interval on OAR 22 (indicated as red box). All 23 affected sheep had homozygous identical by state intervals with shared alleles between 24.5 Mb and 26.9 Mb. (**D**) Gene content of the corresponding human chromosome 10 segment.

We genotyped approximately 50'000 evenly spaced SNPs from 23 microphthalmia affected lambs and 23 control sheep. A genome-wide significant association was shown for SNPs on sheep chromosome 22 (OAR 22) (Figure 2A). Of the remaining 44,865 SNPs with a genotyping rate >99% and minor allele frequency >5%, eights SNPs, over the region 24,529,089 to 28,147,610 on OAR 22, showed strongest association with the microphthalmia phenotype, with a genome-wide corrected p<0.01 (Figure 2B). No other region in the genome showed genome-wide associated SNPs. The OAR 22 SNP at position 24,952,721 showed strongest association with the microphthalmia phenotype, with an asymptotic raw p of 7.6×10^−11^ and a genome-wide corrected of 9.9×10^−5^.

Subsequently, we applied a homozygosity mapping approach to narrow the region containing the microphthalmia mutation. Based on the reported occurrence of microphthalmia some generations after the introgression of Texel sheep from the Netherlands we hypothesized that the affected sheep most likely were inbred to one single founder animal. Under this scenario the affected lambs were expected to be identical by descent (IBD) for the causative mutation and flanking chromosomal segments. We analyzed the cases for extended regions of homozygosity with simultaneous allele sharing. Only one genome region fulfilled our search criteria (Table S1). On OAR 22 all 23 affected genotyped sheep were homozygous and shared identical alleles over 39 SNP markers corresponding to a 2.4 Mb interval from 24.5–26.9 Mb (Figure 2C).

In order to further examine the critical interval defined using SNP data, we genotyped three microsatellite markers derived from the surrounding virtual genome sequence of OAR 22 (Table S2). The analysis of the microsatellite genotypes confirmed an increased homozygosity within the microphthalmia affected lambs compared to the controls. The observed microsatellite heterozygosity in the cases ranged from 3–52% compared to 84–92% in the controls (Table S2). A total of 130 out of 134 microphthalmia affected lambs showed homozygosity at microsatellite *INRA81* located at 24.9 Mb on the virtual genome map of OAR 22 (Table S1). The obtained LOD score of 10.5 conclusively confirmed the linkage of microphthalmia to the candidate gene region in the family material.

### Identification and Characterization of Functional Candidate Genes

**Figure 3:**
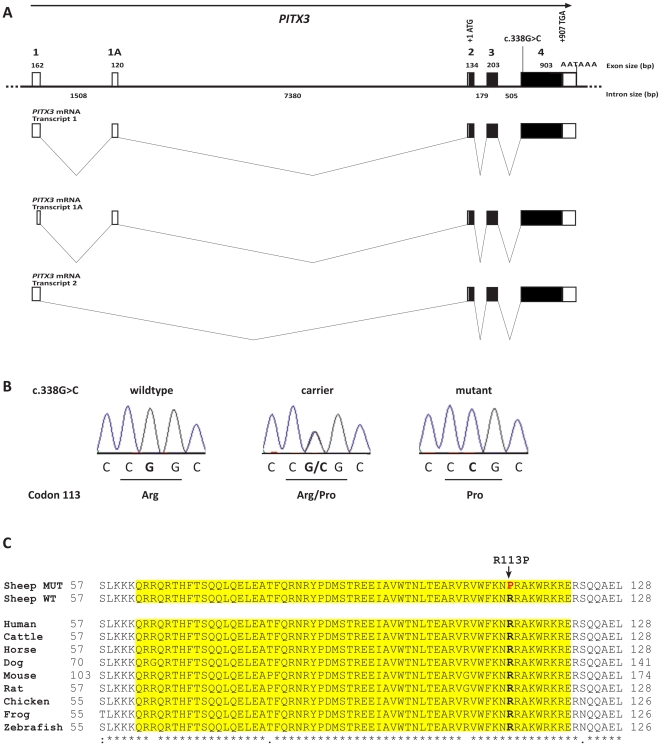
*PITX3* mutation analysis. (**A**) Ovine *PITX3* gene structure. (**B**) Electropherograms of the *PITX3* c. 338G>C mutation. Representative sequence traces of PCR products amplified from genomic DNA of three sheep with the different genotypes are shown. (**C**) Multispecies alignment of the evolutionary conserved homeodomain of the PITX3 protein sequence. The p.R113P mutation in PITX3is indicated by an arrow. It affects an arginine residue, which is perfectly conserved from human to zebrafish across all investigated species. The sequences for the alignment were taken from the following accessions: (sheep, NP_005020 (human), XP_589431 (cattle), XP_001499185 (horse), EDL41981 (mouse), NP_062120 (rat), XP_421631 (chicken), NP_001082023 (*Xenopus laevis*), NP_991238 (*Danio rerio*).

As the resolution of the virtual sheep genome annotation is still far from perfect [22], we inferred the gene annotation of the mapped interval from the corresponding human interval (Figure 2D). The sheep microphthalmia interval corresponds to a segment from 102.2–104.8 Mb on HSA 10. This human interval contains 46 annotated genes and 5 hypothetical loci (NCBI MapViewer, build 36.3). A careful inspection of these genes and database searches of their presumed function revealed *PAX2* and *PITX3* as possible functional candidate genes within the critical interval at 102.5 Mb and 103.9 Mb on HSA 10, respectively. The paired box gene 2 (*PAX2*) is a transcription factor expressed during embryonic eye development and mutations in the human and mouse homologs are accompanied by eye malformations [1]. *PITX3* encodes the paired-like homeodomain 3 (PITX3) transcription factor, expressed during early vertebrate lens development [23]. Deletions in the promoter of this gene cause abnormal lens development in the *aphakia* mouse mutant, which has only rudimentary lenses [24], [25]. An abnormal development of the lens vesicle was shown to be the primary event in ovine microphthalmia as well [19]. Therefore, we initially investigated whether mutations in the ovine *PITX3* gene might be responsible for the microphthalmia phenotype. As neither mRNA nor genomic sequence data of ovine *PITX3* was publicly available, we determined the complete sequence from an ovine BAC clone containing the *PITX3* gene. In order to further evaluate *PITX3* as a positional candidate, we analyzed its expression in head tissue of d30 sheep fetuses because *PITX3* shows a lens specific expression in human, mice, and cattle embryos, respectively. We detected three transcripts, which were verified by direct DNA-sequencing of the RT-PCR products. Full-length *PITX3* cDNAs obtained by RACE differed in their 5′ untranslated regions, whereas they shared a common open reading frame (Figure 3A). These cDNA sequences were used for comparison with the genomic sequence. These analyses indicated that the ovine *PITX3* gene consists of five exons separated by four introns. In accordance to the human gene annotation, we numbered the two alternatively spliced 5′ exons 1 and 1A and the three coding exons 2–4 (Figure 3A). All splice donor/splice acceptor sites conform to the GT/AG rule. The experimentally verified existence of the alternatively spliced exon 1A is in agreement with an identified cattle 5′-EST sequence (GenBank: EG705801). The coding sequence of ovine *PITX3* displays 97%, 90% and 88% similarity to the bovine, human and murine homologue, respectively. Ovine *PITX3* encodes a protein of 302 amino acids containing a DNA binding homeobox domain and, like in other *paired*-like homeodomain containing proteins, an otp, aristaless, and rax (OAR) domain of 14 amino acids within the C-terminal region. PITX3 along with PITX1 and PITX2 form the PITX/RIEG sub-family of the *Paired*-like class of homeobox proteins which are highly conserved across species [26], [27]. In addition, we obtained cDNA sequence of ovine *PAX2* by sequencing RT-PCR products of head tissue of d30 sheep fetuses. We detected a single *PAX2* transcript with an open reading frame of 1182 bp encoding a protein of 393 amino acids containing a N-terminal paired box (PAX) domain which is 98% identical to human PAX2 isoform b.

### Mutation Analysis

We designed PCR primers for the amplification of a 14.5 kb segment containing the entire *PITX3* gene and determined the genomic sequence of four microphthalmia affected and four healthy control sheep. This analysis revealed just a single sequence polymorphism (Figure 3A). This non-synonymous SNP located in *PITX3* exon 4 (c.338G>C; Figure 3B) showed perfect association to the microphthalmia phenotype (Table 1). All 134 affected sheep were homozygous C/C and all 133 known carriers were heterozygous G/C. Twelve out of 47 healthy full- and half-sibs of microphthalmia affected sheep were also heterozygous G/C. Testing a population sample of 89 healthy Texels which had unknown relatedness revealed none carried the homozygous C/C genotype, while four were presumed carriers with the G/C genotype. Thus the allele frequency of the deleterious C-allele within a population sample of Texel sheep was 2.2%. The mutant C-allele was absent from 115 control sheep from 14 diverse sheep breeds. RT-PCR on cDNA and subsequent sequencing confirmed that the *PITX3* RNA is normally spliced in d30 fetuses of both available genotypes, homozygous C/C and heterozygous G/C, respectively. The c.338G>C substitution is predicted to result in an exchange of an arginine to a proline in the homeodomain of PITX3 protein sequence (p.R113P). This suggests that the G>C SNP is located in a functionally important region. The potential impact of this microphthalmia associated substitution was evaluated by multiple species alignment of PITX3 homeodomain (Figure 3C). PITX3 belongs to the *paired*-like homeodomain containing transcription factor group and a *bicoid*-like subgroup [25]. The *bicoid*-like homeodomains are characterized by a lysine at position 50 in the homeodomain, which is known to selectively recognize the 3′ CC dinucleotide adjacent to the TAAT core DNA binding sequence. The microphthalmia associated p.R113P substitution is located at the perfectly conserved position 52 of the homeodomain. To evaluate possible functional consequences we searched for experimentally determined protein structures of PITX family members. The structure of the human PITX2 homeodomain-DNA complex showed that the p.R113P mutation is indeed likely to affect the DNA-binding function of PITX3 [27]. The mutant proline shows an uncharged side chain and it cannot act as a hydrogen bond donor (Figure S2). Therefore, as proline disrupts alpha helixes in general, it may act as a structural disruptor in the middle of third alpha helix of the PITX3 homeodomain. Furthermore, the putative consequences of the polymorphisms on the modified proteins were analyzed using two independent computer based amino acid conservation analysis software. These programs are sequence homology-based tools that sort intolerant from tolerant amino acid substitutions and predict whether an amino acid substitution in a protein has a possible phenotypic effect. According to PolyPhen, the PITX3 p.R113P substitution is damaging with a high probability (score 2.6) and the PMut calculation predicts a highly reliable pathological effect.

**Table 1:** Association of the *PITX3* mutation with the microphthalmia phenotype.

		Texel or Texel/Whiteheaded mutton crossbred sheep				Other breeds
*PITX3*		microphthalmia affected 1 (n = 134)	microphthalmia carrier 2 (n = 133)	Control, related 3 (n = 47)	Control, unknown relationship (n = 89)	Controls (n = 115)
**c.338G>C**	**GG**			**12**	**85**	**115**
**(p.R113P)**	**GC**		**133**	**35**	**4**	
	**CC**	**134**				

1 Thirty not closely related cases and 104 affected lambs from the family material.

2 Thirty not closely related sheep recorded as parent of affected lambs and 103 parents from the family material.

3 Healthy littermates of affected lambs.

To test for possible *PAX2* mutations we sequenced RT-PCR products of the experimentally derived d30 sheep fetuses. No size difference or alternative splicing was detected and analyzing the ORF of four individuals, two homozygous and two heterozygous for the associated *INRA81* microsatellite allele, respectively, revealed no polymorphisms affecting the amino acid sequence of PAX2.

## Discussion

Historically, the development of genomic tools for the sheep genome has lagged behind those of other major livestock species such as the cattle and chicken. This has limited the ability to identify genes controlling specific traits of interest [28]. The development of low density microsatellite based linkage maps [29] have lead to the mapping of Mendelian diseases [30]–[32] and subsequent discovery of mutations underlying at least three genetic diseases in sheep [32]–[34], however many others remain uncharacterized [35]. The recent development of a set of SNP markers distributed across the sheep genome has changed the prerequisites for such gene mapping projects. For the first time, we demonstrate the use of a genome-wide ovine SNP array for efficient positional cloning of a Mendelian trait in sheep. The result illustrates the power of genome-wide association analysis in domestic animals for the genetic dissection of trait loci [36].

Twenty-three affected and 23 controls were selected for genome-wide association and were genotyped using Illumina's OvineSNP50 BeadChip. Both, case-control association analysis with genome-wide significance and homozygosity mapping identified the same OAR 22 region for microphthalmia in Texel sheep. The analysis of three flanking OAR 22 microsatellites confirmed an increased homozygosity within a total of 134 microphthalmia affected lambs compared to 212 controls. In addition, the availability of segregating families allowed us to confirm the mapping on OAR 22 by linkage analysis. Taken together, the presented statistical support showed that the previously published genetic linkage to microsatellite markers on sheep chromosome 23 using a subset of the family material now did not prove to be correct [21].

The *PITX3* gene was the most compelling functional candidate in the 2.4 Mb critical interval (Figure 2C). It was not, however, the only plausible positional candidate as the paired box gene 2 (*PAX2*) is located within the region. While it has been associated with eye malformations in human and mouse, these congenital phenotypes are usually accompanied by kidney anomalies [1] which have not been observed in the ovine form of the disease [20], [21]. Sequencing of the coding sequence of *PAX2* showed no evidence for disease causing mutations in sheep. However, DNA sequencing revealed a non-synonymous mutation in the *PITX3* gene, which is perfectly associated with the microphthalmia phenotype in Texel sheep. We confirmed the presence of this mutation on the genomic DNA and mRNA level. Although we cannot provide functional proof of the causality of the mutation at this time, the wealth of functional data, which are available for the *PITX3* gene, strongly supports the hypothesis that p.R113P is indeed the causative mutation. Human patients with point mutations in *PITX3* demonstrate congenital cataracts along with anterior segment defects (ASD) in some cases when one allele is affected and microphthalmia with brain malformations when both copies are mutated [10], [37]. ASD includes a spectrum of developmental abnormalities of the cornea (Peters anomaly), iris (Axenfeld-Rieger syndrome), iridocorneal angle, and ciliary body, which are caused by mutations in the homeodomain of *PITX2*, encoding a closely related transcription factor [27], [38]. A morphological description of microphthalmia in Texel sheep showed an abnormal development of the lens vesicle [19]. These authors also concluded that hereditary microphthalmia in Texel sheep closely resembles autosomal recessive lens aplasia in mice [39]. A similar phenotype is also the key feature in the *Pitx3* loss of function *aphakia* mouse, where the lens begins to form, but its development is abnormal [24], [25]. Morpholino-induced knockdown of *pitx3* at early embryonic stages in zebrafish resulted in a lens and retinal phenotype similar to the one seen in the *aphakia* mouse [40].

The known *PITX3* mutations in mouse and humans don't affect the homeodomain, which is different from that seen in other homeodomain proteins including its close family member, *PITX2* [26]. The reported p.R113P mutation in ovine microphthalmia lies within the conserved homeodomain and the wildtype arginine is conserved across all PITX3 sequences (Figure 3C). Therefore, it is likely that the mutation p.R113P results in an impaired lens development. In addition, the arginine residue at the similar position of PITX2 was found to be mutated to cysteine in patients with Axenfeld-Rieger syndrome [26], [27]. Furthermore, in the wildtype Arg113 is located in the middle of an alpha helix and binds to the DNA that is highly likely not possible with the imino acid proline as possible helix disruptor (Figure S2). Thus it is conceivable that this mutation affects the proper folding and stability of the native conformation, possibly inactivating the transcription factor significantly. Apparently, one copy of the PITX3 wildtype allele is sufficient for regulation an undisturbed embryonic lens development, because no visible eye phenotype has been reported in heterozygous carriers of the microphthalmia mutation [19], [20]. The p.R113P mutation in microphthalmia affected sheep probably does not affect skeletal muscular development in newborn lambs, although *Pitx3* expression during myogenesis has been reported [41]. In mice it was speculated that the lacking PITX3 function in muscles is perhaps completely compensated by the maintenance of PITX2 expression [41]. A microRNA was identified that regulates the maturation and function of midbrain dopaminergic neurons within a negative feedback circuit including the transcription factor PITX3 [42]. The authors propose a role for this feedback circuit in the fine-tuning of dopaminergic behaviors such as locomotion. We found no clinical evidence for conspicuous behavior in microphthalmia affected Texel sheep [20].

Many inherited diseases of domestic animals are analogous to human hereditary disorders and have proven to be valuable for the investigation of the pathogenesis and therapeutic trials of rare human phenotypes with identical molecular basis [43]. Recently, a mutation causing an autosomal recessive inherited disorder characterized by dysplasia of the lens, retinal detachment, persistence of the hyaloid artery, and microphthalmia in cattle was identified [44]. This large animal model demonstrated the essential role of *WFDC1*, a small secretory protein specifically expressed in the lens, retina, and optic nerves of embryonic and adult mouse eyes, in mammalian eye development for the first time. Our finding of a *PITX3* p.R113P mutation in sheep with microphthalmia provides a valuable large animal model for human medicine and confirms *PITX3* as a microphthalmia gene. Our study indicates that coding mutations of the *PITX3* gene might also be responsible for rare recessive forms of human isolated microphthalmia. The naturally occurring microphthalmia sheep model may represent a better model for human microphthalmia than *aphakia* mice because of its eye size and structure and the resulting similarity to the human situation. In addition, the longer life expectancy of sheep allows for investigations over a longer time period. Domestic production animals have the additional advantages of being economic to maintain and having been bred for easy management. Moreover, a high level of expertise in reproductive technology and veterinary care is available for them.

In conclusion, we have identified the p.R113P mutation in the ovine *PITX3* gene as the candidate causative mutation for microphthalmia in Texel sheep. In comparison to the commercially available DNA test which relies on linked markers [45] this result allows direct genetic testing and improved power to eradicate this common genetic disease from the worldwide Texel breeding population. Our study also provides a defined animal model for similar human hereditary diseases and confirms PITX3 critical function for eye development.

## Materials and Methods

### Animals

We collected samples from 134 microphthalmia affected lambs (59 male, 75 female), their available healthy siblings (n = 47), sires (n = 10), and dams (n = 93) from different sheep farms with Texel purebred or Texel/Whiteheaded mutton crossbred sheep and our experimentally established pedigree [20], [21]. In addition, we collected 30 Texel sheep recorded as parents of microphthalmia affected offspring. We also collected 89 healthy Texel sheep with unknown relatedness resulting in a total of 403 samples.

Furthermore, we sampled unrelated control sheep from different breeds (Whiteheaded mutton (n = 50), Swiss White Alpine (n = 24), Bündner Oberländer (n = 4), Engadine Red (n = 4), Swiss Black-Brown Mountain (n = 4), Swiss Mirror (n = 4), Valais Blacknose (n = 4), Valais Red (n = 4), Suffolk (n = 1)) and 16 founder animal of the International Mapping Flock (Texel, Coopworth, Perendale, Romney, Merino) [46] for the re-sequencing of *PITX3* exon 4.

### Fetal Tissue

Tissues were collected in accordance with the animal care and use protocols approved by the Lower Saxony (Germany) governmental animal rights protection authorities (Ref. No. 509.6-42502/3-04/851). A total of six d30 fetuses were surgically obtained after targeted mating of an affected male to two known disease carriers (Figure S1). Upon collection, fetuses were divided in front (head) and back section and stored in RNAlater (Qiagen).

#### DNA and RNA extraction

Genomic DNA was isolated from blood or tissue using the Nucleon Bacc2 kit (GE Healthcare). Total RNA was isolated using Trizol reagent according to the manufacturer's instructions (Invitrogen).

#### Mapping of the microphthalmia mutation

Genomic DNA from 23 cases and 23 controls, mostly selected as discordant sib-pairs, was genotyped using Illumina's OvineSNP50 BeadChip (49,034 SNPs) [47]. Two small nuclear families segregating for microphthalmia were included to check SNPs for Mendelian inheritance. The results were analyzed with PLINK [48]. After removing 5 SNPs with low genotyping success (failed calls >0.1) the average genotyping rate per individual was 99.9%. A total of 4,164 SNPs had a minor allele frequency (MAF)<0.05. A case-control analysis using the options –assoc was applied. Genome-wide corrected empirical p-values were determined applying the max(T) permutation procedure implemented in PLINK with 10,000 permutations. To identify extended homozygous regions with allele sharing across all affected animals the options –homozyg-group and –homozyg-match were applied. All given positions correspond to the virtual sheep genome v 2 [22]. The corresponding human chromosome segment was identified by BLASTN searches of ovine SNP flanking sequences to the human genome sequence.

#### Linkage analysis in candidate genes

Microsatellite markers were amplified using the Multiplex PCR Kit (Qiagen) and fragment size analyses were determined on an ABI 3730 capillary sequencer (Applied Biosystems) and analyzed with the GeneMapper 4.0 software (Applied Biosystems). Twopoint parametric linkage analysis under the assumption of microphthalmia segregating as a biallelic autosomal recessive trait with complete penetrance was performed with Merlin software version 1.1.2 [49]. The frequency of the mutant allele in the considered population was unknown and there were no data available that would have made it possible to estimate the frequency in a reliable manner. For the calculations a frequency of 0.001 for the mutant allele was assumed. The LOD score test statistic was used to estimate the proportion of linked families and the corresponding maximum heterogeneity LOD score. Within the available family material, a maximum LOD score of 11.512 would have been possible.

#### Analysis of the ovine *PITX3* gene and mutation identification

The BAC clone CH243-315I22 containing the ovine *PITX3* gene was identified by BLASTN searches of ovine BAC end sequences to the HSA 10 sequence [22]. The BAC DNA was prepared using the Qiagen Midi plasmid kit according to the modified protocol for BAC clones (Qiagen). The insert sequence of 127 kb of the CH243-315I22 BAC clone has been determined by generating 1,149,790 read pairs using the Chrysalis 36cycles v 2.0 kit on a Genome Analyzer II (Illumina) and *de novo* assembly with Velvet [50]. Remaining gaps were closed by a primer walking strategy and an ABI 3730 capillary sequencer (Applied Biosystems) until both strands were completely sequenced. The obtained sequence was submitted under accession FN432136 to the EMBL nucleotide database.

The human reference *PITX3* mRNA (GenBank: NM_005029) was used as query in cross-species BLAST searches identifying corresponding bovine EST entries (GenBank: EG705801, EG707045, EG707660). Initially, the putative ovine genomic structure was determined by alignment of bovine *PITX3* EST and human *PITX3* mRNA to the determined ovine genomic sequence using Spidey [51]. Later, the exact ovine genomic structure was determined using the experimentally derived ovine mRNA sequences.

For mutation analysis, PCR products were amplified from four microphthalmia affected and four healthy control sheep using AmpliTaq Gold 360 Master Mix (Applied Biosystems). The sequences of the primers are listed in Table S3. The subsequent re-sequencing of the PCR products was performed after rAPid alkaline phosphatase (Roche) and exonuclease I (New England Biolabs) treatment using both PCR primers with the ABI BigDye Terminator Sequencing Kit 3.1 (Applied Biosystems) on an ABI 3730. Sequence data were analyzed with Sequencher 4.9 (GeneCodes).

To test possible functional consequences, the modified protein was analyzed with two different software packages, PolyPhen [52] and PMut [53].

#### RT-PCR

Aliquots of 1 µg total RNA were reverse transcribed into cDNA using 20 pmol (T)_24_V primer and Superscript III reverse transcriptase (Invitrogen). Two microliters of the cDNA were used as a template in PCR. RT-PCR reactions were performed as described above and primer sequences are given in Table S4. The human reference *PAX2* mRNA (GenBank: NM_003987) was used as query in cross-species Spidey alignment to identifying corresponding cattle sequences. Primers for RT-PCR in sheep were derived from the bovine *PAX2* 5′UTR and 3′UTR sequence. Isolation of full length cDNA for the ovine *PITX3* gene was achieved by a rapid amplification of cDNA ends (RACE) protocol with the FirstChoice RNA ligase-mediated (RLM)-RACE kit (Applied Biosystems). The ovine cDNA sequences were deposited in the EMBL nucleotide database (*PITX3*: FN432137, FN432138, and FN432139; *PAX2*: FN600706).

## Supporting Information

Figure S1 Pedigrees of families in study. Filled symbols represent microphthalmia affected sheep, open symbols represent normal sheep. DNA samples were available from numbered sheep. Two females, which appear as mothers in different families, are marked with a rectangle. Sheep that were used for the initial whole genome association study are marked with an asterisk. The genotypes for the *PITX3* c.338G>C mutation are given below the symbols. A single affected and two healthy offspring from the experimental family 73 were used to obtain d30 fetuses (E4-E6).
(0.04 MB PDF)

Figure S2 Impact of the microphthalmia associated *PITX3* substitution. (A) Alignment of human *PITX3* (NP_005020) and human *PITX2* isoform a, b, and c (NP_700476; NP_700475; NP_000316) protein sequences. The conserved domains (homeodomain and the C-terminal OAR domain) are indicated in bold face type. The mutated arginine at *PITX3* position 113 is shown in red. (B) Structure of the *PITX2* homeodomain-DNA complex (Code 1YZ8 taken from PDB [54]). The tertiary structure of the *PITX2* homeodomain (96% identical to sheep *PITX3* homeodomain) is composed of three alpha helices (shown in light grey). The wildtype *PITX3* Arg113 equivalent *PITX2* Arg90 (shown with carbon atoms in pink) is located in the third alpha helix and binds with its positively charged capped guanidinium group (shown with blue nitrogen atoms) via two hydrogen bonds (indicated in black) to the DNA backbone (oxygens are shown in red, nitrogen atoms in blue, and carbon atoms in green and light blue).
(0.53 MB PDF)

Table S1 Results of homozygosity mapping.
(1.66 MB PDF)

Table S2 Microsatellites.
(0.01 MB PDF)

Table S3 Primer sequences for the amplification of ovine *PITX3* gene.
(0.02 MB PDF)

Table S4 Primer sequences for the amplification of ovine *PITX3* and *PAX2* cDNA.
(0.01 MB PDF)
