# Supplementary figures and images for: Microphthalmia in Texel Sheep Is Associated with a Missense Mutation in the Paired-Like Homeodomain 3 (PITX3) Gene
Source: PLoS One. 2010 Jan 13;5(1):e8689. doi: 10.1371/journal.pone.0008689 (PMC2805710; doi:10.1371/journal.pone.0008689)

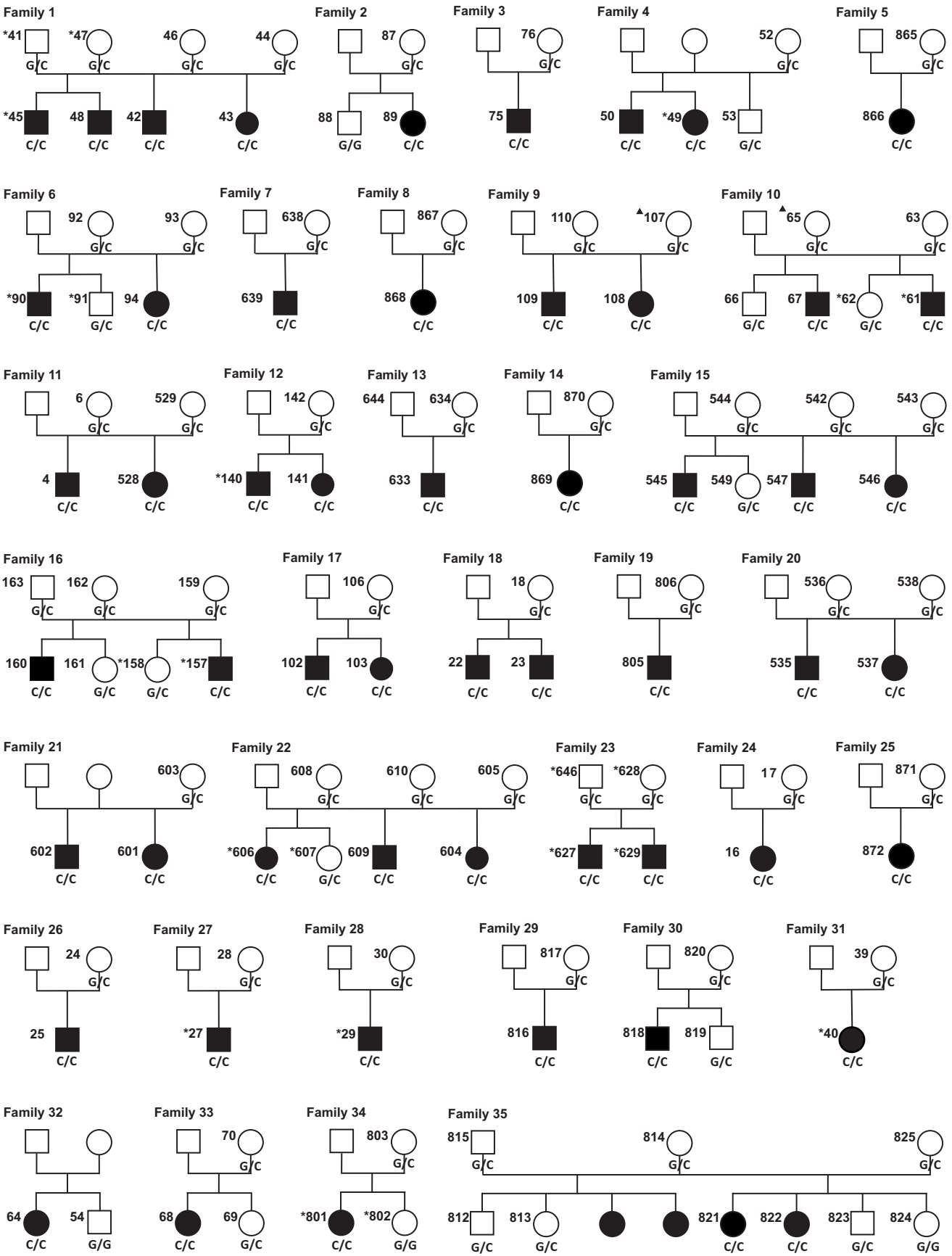

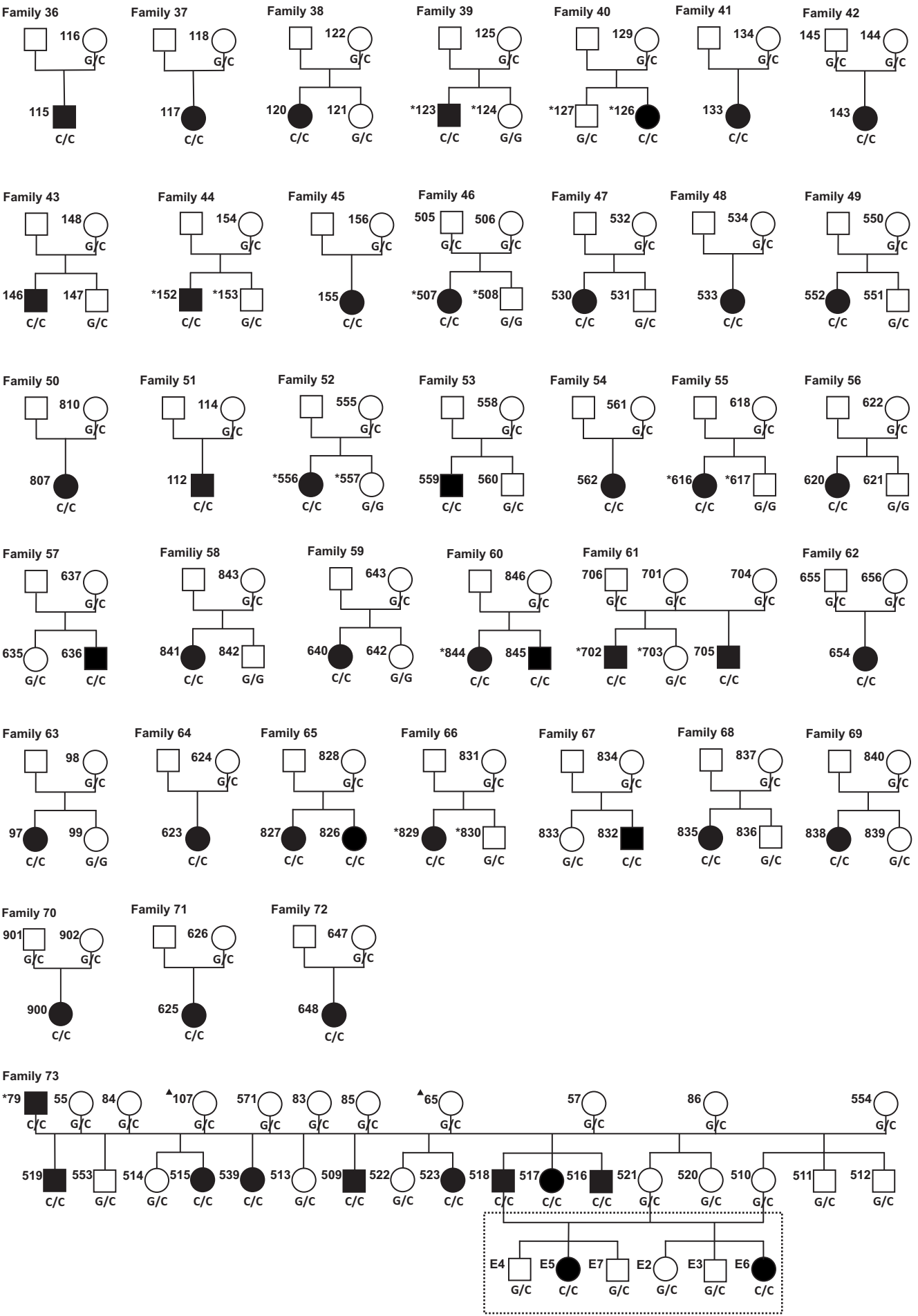

Supplement: Figure S1 — Pedigrees of families in study. Filled symbols represent microphthalmia affected sheep, open symbols represent normal sheep. DNA samples were available from numbered sheep. Two females, which appear as mothers in different families, are marked with a rectangle. Sheep that were used for the initial whole genome association study are marked with an asterisk. The genotypes for the PITX3 c.338G>C mutation are given below the symbols. A single affected and two healthy offspring from the experimental family 73 were used to obtain d30 fetuses (E4-E6). (0.04 MB PDF) [file pone.0008689.s001.pdf]
