# Supplementary material for: Microphthalmia in Texel Sheep Is Associated with a Missense Mutation in the Paired-Like Homeodomain 3 (PITX3) Gene
Source: PLoS One. 2010 Jan 13;5(1):e8689. doi: 10.1371/journal.pone.0008689 (PMC2805710; doi:10.1371/journal.pone.0008689)
Supplement: Table S1 — Results of homozygosity mapping. (1.66 MB PDF) [file pone.0008689.s003.pdf]

Table S1. Results of homozygosity mapping.

The table shows a slightly modified version of the PLINK output file "plink.hom.overlap". Only one interval on chromosome 22 showed homozygosity and shared alleles across all twenty-three affected sheep (highlighted in gray).
For the analysis the following PLINK options were used: --sheep --homozyg-kb 100 --homozyg-snp 30--homozyg-gap 2500--homozyg-density 250--homozyg-group --homozyg-window-snp 20--homozyg-window-missing 5--homozyg-match 0.95--maf 0--hwe 0--max-maf 1.0 --geno 0.10 --mind 0.15--filter-cases--allow-no-sex

POOL		IID	CHR	SNP1	SNP2	KB	NSNP NSI
S1		844	22	OAR22_24300913.1	OAR22_28815692.1	4514.78	77
S1		556	22	s70367.1	OAR22_26886276.1	2667.33	47
S1		616	22	OAR22_24105777.1	OAR22_29668920.1	5563.14	102
S1		45	22	OAR22_23956504.1	OAR22_28815692.1	4859.19	86
S1		829	22	OAR22_23956504.1	OAR22_28815692.1	4859.19	86
S1		606	22	OAR22_22756887.1	OAR22_34014868.1	11258	219
S1		90	22	OAR22_22756887.1	OAR22_29668920.1	6912.03	124
S1		152	22	OAR22_22699919.1	OAR22_29668920.1	6969	125
S1		801	22	s50496.1	OAR22_28815692.1	6200.11	108
S1		627	22	s05380.1	OAR22_31098866.1	8673.81	167
S1		629	22	s05380.1	OAR22_31098866.1	8673.81	167
S1		27	22	OAR22_22410821.1	OAR22_28815692.1	6404.87	113
S1		79	22	OAR22_22410821.1	OAR22_28815692.1	6404.87	113
S1		157	22	OAR22_22410821.1	OAR22_28815692.1	6404.87	113
S1		40	22	OAR22_22410821.1	OAR22_28320087.1	5909.27	104
S1		126	22	s11593.1	OAR22_36686850.1	14557	280
S1		29	22	OAR22_21956630.1	OAR22_31064768.1	9108.14	176
S1		140	22	s53575.1	OAR22_29668920.1	7750.65	144
S1		507	22	s06758.1	OAR22_42355166.1	24436.4	466
S1		123	22	OAR22_17622066.1	OAR22_28097363.1	10475.3	179
S1		61	22	OAR22_14956172.1	s08672.1	23753.1	404
S1		49	22	OAR22_12868251.1	OAR22_35805166.1	22936.9	381
S1		702	22	OAR22_9619017.1	s61119.1	25865.1	434
S1	CON	23	22	OAR22_24300913.1	OAR22_26886276.1	2585.36	45
S1	UNION	23	22	OAR22_9619017.1	OAR22_42355166.1	32736.1	572
S10		507	2	s20121.1	OAR2_134249434.1	7112.48	154
S10		844	2	s20121.1	OAR2_133793734.1	6656.78	145
S10		61	2	OAR2_126263077.1	OAR2_134940115.1	8677.04	181
S10		123	2	OAR2_126263077.1	OAR2_128764057.1	2500.98	45
S10		616	2	OAR2_124098623.1	OAR2_128764057.1	4665.43	89
S10		29	2	OAR2_119004082.1	OAR2_132080120.1	13076	229
S10		126	2	OAR2_116277389.1	OAR2_134940115.1	18662.7	353
S10		627	2	OAR2_116277389.1	OAR2_127514723.1	11237.3	193
S10		629	2	OAR2_116277389.1	OAR2_127514723.1	11237.3	193
S10		45	2	OAR2_116066653.1	OAR2_128764057.1	12697.4	221
S10		140	2	OAR2_116066653.1	OAR2_127514723.1	11448.1	197
S10		829	2	OAR2_116066653.1	OAR2_127514723.1	11448.1	197
S10		27	2	OAR2_115851252.1	OAR2_129745095.1	13893.8	249
S10		90	2	s70568.1	OAR2_127514723.1	15298.4	269
S10	CON	14	2	s20121.1	OAR2_127514723.1	377.77	9
S10	UNION	14	2	s70568.1	OAR2_134940115.1	22723.8	429
S11		61	2	OAR2_126263077.1	OAR2_134940115.1	8677.04	181
S11		123	2	OAR2_126263077.1	OAR2_128764057.1	2500.98	45
S11		616	2	OAR2_124098623.1	OAR2_128764057.1	4665.43	89
S11		29	2	OAR2_119004082.1	OAR2_132080120.1	13076	229
S11		126	2	OAR2_116277389.1	OAR2_134940115.1	18662.7	353
S11		627	2	OAR2_116277389.1	OAR2_127514723.1	11237.3	193

S11		629	2	OAR2_116277389.1	OAR2_127514723.1	11237.3	193
S11		507	2	OAR2_116277389.1	s75747.1	10718	183
S11		45	2	OAR2_116066653.1	OAR2_128764057.1	12697.4	221
S11		140	2	OAR2_116066653.1	OAR2_127514723.1	11448.1	197
S11		829	2	OAR2_116066653.1	OAR2_127514723.1	11448.1	197
S11		27	2	OAR2_115851252.1	OAR2_129745095.1	13893.8	249
S11		90	2	s70568.1	OAR2_127514723.1	15298.4	269
S11	CON	13	2	OAR2_126263077.1	s75747.1	732.332	11
S11	UNION	13	2	s70568.1	OAR2_134940115.1	22723.8	429
S13		79	13	s27782.1	s05134.1	2127.73	36
S13		61	13	OAR13_43334843.1	s53991.1	1929.67	33
S13		49	13	s47368.1	OAR13_44694056.1	2372.15	41
S13		616	13	OAR13_41982854.1	s13311.1	2969.55	51
S13		27	13	s37104.1	s13311.1	3054.99	53
S13		152	13	s37104.1	s13311.1	3054.99	53
S13		606	13	s37104.1	s13311.1	3054.99	53
S13		40	13	s37104.1	s13311.1	3054.99	53
S13		829	13	s37104.1	s13311.1	3054.99	53
S13		844	13	s37104.1	s13878.1	2832.35	49
S13		801	13	OAR13_40723732.1	OAR13_44858092.1	4134.36	68
S13		556	13	s18674.1	OAR13_44631782.1	5987.19	98
S13	CON	12	13	s27782.1	OAR13_44631782.1	42.078	3
S13	UNION	12	13	s18674.1	s05134.1	8072.84	131
S16		61	13	OAR13_43334843.1	s53991.1	1929.67	33
S16		49	13	s47368.1	OAR13_44694056.1	2372.15	41
S16		616	13	OAR13_41982854.1	s13311.1	2969.55	51
S16		27	13	s37104.1	s13311.1	3054.99	53
S16		152	13	s37104.1	s13311.1	3054.99	53
S16		606	13	s37104.1	s13311.1	3054.99	53
S16		40	13	s37104.1	s13311.1	3054.99	53
S16		829	13	s37104.1	s13311.1	3054.99	53
S16		844	13	s37104.1	s13878.1	2832.35	49
S16		79	13	s74098.1	s07261.1	2464.24	39
S16		801	13	OAR13_40723732.1	OAR13_44858092.1	4134.36	68
S16		556	13	s18674.1	OAR13_44631782.1	5987.19	98
S16	CON	12	13	OAR13_43334843.1	s07261.1	333.724	7
S16	UNION	12	13	s18674.1	s53991.1	6619.93	107
S19		126	5	s52618.1	OAR5_72565538.1	7244.72	139
S19		29	5	s07518.1	OAR5_68302417.1	3681.04	76
S19		801	5	OAR5_63945337.1	s66569.1	3127.86	66
S19		27	5	OAR5_63370705.1	s36664.1	5079.16	100
S19		507	5	OAR5_63370705.1	s66569.1	3702.49	72
S19		123	5	OAR5_63370705.1	s48255.1	3026.44	56
S19		844	5	OAR5_63370705.1	s48255.1	3026.44	56
S19		45	5	s49473.1	s45311.1	3758.74	71
S19		90	5	DU444709_372.1	OAR5_68350537.1	7925.86	122
S19		79	5	OAR5_57265711.1	s17800.1	9271.25	138
S19		629	5	OAR5_57983508.1	OAR5_71824766.1	13841.3	230
S19		627	5	OAR5_57983508.1	OAR5_66325807.1	8342.3	120
S19	CON	12	5	s52618.1	OAR5_66325807.1	1004.99	18
S19	UNION	12	5	OAR5_57265711.1	OAR5_72565538.1	15299.8	253

S20		616	2	OAR2_124098623.1	OAR2_128764057.1	4665.43	89
S20		152	2	OAR2_124098623.1	OAR2_125967358.1	1868.73	42
S20		29	2	OAR2_119004082.1	OAR2_132080120.1	13076	229
S20		126	2	OAR2_116277389.1	OAR2_134940115.1	18662.7	353
S20		627	2	OAR2_116277389.1	OAR2_127514723.1	11237.3	193
S20		629	2	OAR2_116277389.1	OAR2_127514723.1	11237.3	193
S20		507	2	OAR2_116277389.1	s75747.1	10718	183
S20		45	2	OAR2_116066653.1	OAR2_128764057.1	12697.4	221
S20		140	2	OAR2_116066653.1	OAR2_127514723.1	11448.1	197
S20		829	2	OAR2_116066653.1	OAR2_127514723.1	11448.1	197
S20		27	2	OAR2_115851252.1	OAR2_129745095.1	13893.8	249
S20		90	2	s70568.1	OAR2_127514723.1	15298.4	269
S20	CON	12	2	OAR2_124098623.1	OAR2_125967358.1	1868.73	42
S20	UNION	12	2	s70568.1	OAR2_134940115.1	22723.8	429
S23		606	2	OAR2_122084961.1	OAR2_123904873.1	1819.91	33
S23		29	2	OAR2_119004082.1	OAR2_132080120.1	13076	229
S23		702	2	OAR2_117988490.1	s16691.1	5544.96	79
S23		126	2	OAR2_116277389.1	OAR2_134940115.1	18662.7	353
S23		627	2	OAR2_116277389.1	OAR2_127514723.1	11237.3	193
S23		629	2	OAR2_116277389.1	OAR2_127514723.1	11237.3	193
S23		507	2	OAR2_116277389.1	s75747.1	10718	183
S23		45	2	OAR2_116066653.1	OAR2_128764057.1	12697.4	221
S23		140	2	OAR2_116066653.1	OAR2_127514723.1	11448.1	197
S23		829	2	OAR2_116066653.1	OAR2_127514723.1	11448.1	197
S23		27	2	OAR2_115851252.1	OAR2_129745095.1	13893.8	249
S23		90	2	s70568.1	OAR2_127514723.1	15298.4	269
S23	CON	12	2	OAR2_122084961.1	s16691.1	1448.49	22
S23	UNION	12	2	s70568.1	OAR2_134940115.1	22723.8	429
S24		556	2	OAR2_117362770.1	s64230.1	1619.68	31
S24		126	2	OAR2_116277389.1	OAR2_134940115.1	18662.7	353
S24		627	2	OAR2_116277389.1	OAR2_127514723.1	11237.3	193
S24		629	2	OAR2_116277389.1	OAR2_127514723.1	11237.3	193
S24		507	2	OAR2_116277389.1	s75747.1	10718	183
S24		45	2	OAR2_116066653.1	OAR2_128764057.1	12697.4	221
S24		140	2	OAR2_116066653.1	OAR2_127514723.1	11448.1	197
S24		829	2	OAR2_116066653.1	OAR2_127514723.1	11448.1	197
S24		844	2	OAR2_116066653.1	OAR2_118831042.1	2764.39	56
S24		27	2	OAR2_115851252.1	OAR2_129745095.1	13893.8	249
S24		90	2	s70568.1	OAR2_127514723.1	15298.4	269
S24		702	2	OAR2_117988490.1	s16691.1	5544.96	79
S24	CON	12	2	OAR2_117988490.1	OAR2_118831042.1	842.552	16
S24	UNION	12	2	s70568.1	OAR2_134940115.1	22723.8	429
S25		126	2	OAR2_116277389.1	OAR2_134940115.1	18662.7	353
S25		627	2	OAR2_116277389.1	OAR2_127514723.1	11237.3	193
S25		629	2	OAR2_116277389.1	OAR2_127514723.1	11237.3	193
S25		507	2	OAR2_116277389.1	s75747.1	10718	183
S25		45	2	OAR2_116066653.1	OAR2_128764057.1	12697.4	221
S25		140	2	OAR2_116066653.1	OAR2_127514723.1	11448.1	197
S25		829	2	OAR2_116066653.1	OAR2_127514723.1	11448.1	197
S25		844	2	OAR2_116066653.1	OAR2_118831042.1	2764.39	56
S25		27	2	OAR2_115851252.1	OAR2_129745095.1	13893.8	249
S25		61	2	OAR2_114908247.1	OAR2_117156831.1	2248.58	55

S25		152	2	s09491.1	OAR2_116465453.1	2167.44	50
S25		90	2	s70568.1	OAR2_127514723.1	15298.4	269
S25	CON	12	2	OAR2_116277389.1	OAR2_116465453.1	188.064	4
S25	UNION	12	2	s70568.1	OAR2_134940115.1	22723.8	429
S26		27	10	OAR10_39377612.1	OAR10_41227146.1	1849.53	37
S26		90	10	OAR10_39377612.1	OAR10_41227146.1	1849.53	37
S26		606	10	OAR10_39377612.1	OAR10_41174634.1	1797.02	36
S26		556	10	OAR10_37567308_X.1	DU310747_445.1	3232.13	49
S26		61	10	OAR10_37517326.1	OAR10_40747653.1	3230.33	50
S26		49	10	OAR10_37517326.1	OAR10_40475642.1	2958.32	46
S26		45	10	OAR10_35722333.1	OAR10_57396545.1	21674.2	405
S26		123	10	OAR10_35722333.1	OAR10_41054879.1	5332.55	89
S26		702	10	s49567.1	OAR10_48462141.1	48462.1	914
S26		829	10	OAR10_33047233.1	OAR10_51969493.1	18922.3	352
S26		801	10	s38022.1	OAR10_41435807.1	5825.97	101
S26	CON	11	10	OAR10_39377612.1	OAR10_40475642.1	1098.03	22
S26	UNION	11	10	s49567.1	OAR10_57396545.1	57396.5	1095
S27		126	22	s11593.1	OAR22_36686850.1	14557	280
S27		29	22	OAR22_21956630.1	OAR22_31064768.1	9108.14	176
S27		61	22	OAR22_14956172.1	s08672.1	23753.1	404
S27		49	22	OAR22_12868251.1	OAR22_35805166.1	22936.9	381
S27		702	22	OAR22_9619017.1	s61119.1	25865.1	434
S27		801	22	OAR22_31064768.1	OAR22_33115630.1	2050.86	47
S27		606	22	OAR22_22756887.1	OAR22_34014868.1	11258	219
S27		627	22	s05380.1	OAR22_31098866.1	8673.81	167
S27		629	22	s05380.1	OAR22_31098866.1	8673.81	167
S27		507	22	s06758.1	OAR22_42355166.1	24436.4	466
S27	CON	10	22	OAR22_31064768.1	OAR22_31064768.1	0	1
S27	UNION	10	22	OAR22_9619017.1	OAR22_42355166.1	32736.1	572
S28		45	10	OAR10_17232091.1	s09543.1	3126.98	55
S28		123	10	OAR10_17232091.1	OAR10_20247297.1	3015.21	54
S28		90	10	s33555.1	OAR10_19765032.1	3415.2	59
S28		27	10	OAR10_16162080.1	OAR10_19765032.1	3602.95	64
S28		702	10	s49567.1	OAR10_48462141.1	48462.1	914
S28		40	10	OAR10_16100262.1	OAR10_19558015.1	3457.75	62
S28		157	10	OAR10_16100262.1	OAR10_19558015.1	3457.75	62
S28		616	10	OAR10_16100262.1	OAR10_18818595.1	2718.33	48
S28		507	10	OAR10_15620012.1	OAR10_19558015.1	3938	70
S28		844	10	OAR10_17703888.1	s09622.1	13279.3	268
S28	CON	10	10	OAR10_17703888.1	OAR10_18818595.1	1114.71	19
S28	UNION	10	10	s49567.1	OAR10_48462141.1	48462.1	914
S31		27	1	OAR1_126414415.1	OAR1_138926518.1	12512.1	216
S31		629	1	OAR1_125784636.1	OAR1_130654375.1	4869.74	56
S31		627	1	s03118.1	OAR1_141731333.1	16512	283
S31		45	1	s37607.1	s04671.1	16346.9	247
S31		29	1	OAR1_126268263.1	s01965.1	6350.53	88
S31		79	1	s75317.1	s66718.1	6978.38	93
S31		702	1	s14189.1	s40751.1	42088.8	712
S31		123	1	s53597.1	OAR1_153662211.1	35798.6	631
S31		90	1	OAR1_124078281.1	OAR1_138926518.1	14848.2	257
S31		616	1	s01352.1	OAR1_130649077.1	3160.47	30

S31	CON	10	1	s01352.1	OAR1_130649077.1	3160.47	30
S31	UNION	10	1	s14189.1	OAR1_153662211.1	45938.1	791
S33		27	1	OAR1_126414415.1	OAR1_138926518.1	12512.1	216
S33		629	1	OAR1_125784636.1	OAR1_130654375.1	4869.74	56
S33		627	1	s03118.1	OAR1_141731333.1	16512	283
S33		45	1	s37607.1	s04671.1	16346.9	247
S33		90	1	OAR1_124078281.1	OAR1_138926518.1	14848.2	257
S33		606	1	OAR1_120032190.1	s74762.1	6498.59	124
S33		29	1	OAR1_126268263.1	s01965.1	6350.53	88
S33		79	1	s75317.1	s66718.1	6978.38	93
S33		702	1	s14189.1	s40751.1	42088.8	712
S33		123	1	s53597.1	OAR1_153662211.1	35798.6	631
S33	CON	10	1	OAR1_126414415.1	s74762.1	116.364	3
S33	UNION	10	1	s14189.1	OAR1_153662211.1	45938.1	791
S35		556	10	OAR10_37567308_X.1	DU310747_445.1	3232.13	49
S35		61	10	OAR10_37517326.1	OAR10_40747653.1	3230.33	50
S35		49	10	OAR10_37517326.1	OAR10_40475642.1	2958.32	46
S35		844	10	s18518.1	s35062.1	1609.48	32
S35		45	10	OAR10_35722333.1	OAR10_57396545.1	21674.2	405
S35		123	10	OAR10_35722333.1	OAR10_41054879.1	5332.55	89
S35		702	10	s49567.1	OAR10_48462141.1	48462.1	914
S35		829	10	OAR10_33047233.1	OAR10_51969493.1	18922.3	352
S35		801	10	s38022.1	OAR10_41435807.1	5825.97	101
S35	CON	9	10	OAR10_37567308_X.1	s35062.1	119.095	4
S35	UNION	9	10	s49567.1	OAR10_57396545.1	57396.5	1095
S36		79	4	OAR4_21951630.1	OAR4_23831198.1	1879.57	43
S36		157	4	OAR4_21436345.1	OAR4_23013880.1	1577.54	36
S36		152	4	OAR4_20468121.1	OAR4_24841202.1	4373.08	92
S36		123	4	OAR4_19550445.1	OAR4_22796537.1	3246.09	61
S36		606	4	OAR4_15211722_X.1	OAR4_24841202.1	9629.48	193
S36		702	4	OARUn.284_293028.1	OAR4_24251309.1	24251.3	470
S36		49	4	OAR4_22439455.1	OAR4_29317435.1	6877.98	155
S36		27	4	OAR4_21073671.1	OAR4_22765057.1	1691.39	37
S36		616	4	OAR4_22143169.1	s60027.1	4198.5	90
S36	CON	9	4	OAR4_22439455.1	OAR4_22765057.1	325.602	7
S36	UNION	9	4	OARUn.284_293028.1	OAR4_29317435.1	29317.4	582
S37		616	4	OAR4_22143169.1	s60027.1	4198.5	90
S37		844	4	OAR4_20488468.1	s32131.1	1894.04	35
S37		123	4	OAR4_19550445.1	OAR4_22796537.1	3246.09	61
S37		606	4	OAR4_15211722_X.1	OAR4_24841202.1	9629.48	193
S37		702	4	OARUn.284_293028.1	OAR4_24251309.1	24251.3	470
S37		79	4	OAR4_21951630.1	OAR4_23831198.1	1879.57	43
S37		157	4	OAR4_21436345.1	OAR4_23013880.1	1577.54	36
S37		152	4	OAR4_20468121.1	OAR4_24841202.1	4373.08	92
S37		27	4	OAR4_21073671.1	OAR4_22765057.1	1691.39	37
S37	CON	9	4	OAR4_22143169.1	s32131.1	239.337	7
S37	UNION	9	4	OARUn.284_293028.1	s60027.1	26341.7	510
S39		606	3	s14975.1	OAR3_47755179.1	3083.7	63
S39		627	3	OAR3_44553219.1	OAR3_46866444.1	2313.22	46
S39		629	3	OAR3_44553219.1	OAR3_46866444.1	2313.22	46

S39		123	3	s72091.1	s14476.1	1854.07	41
S39		507	3	OAR3_43871305.1	OAR3_52722619.1	8851.31	160
S39		49	3	OAR3_43700480.1	OAR3_50145205.1	6444.73	118
S39		702	3	DU259120_464.1	OAR3_59494261.1	59494.3	1111
S39		27	3	s12024.1	s69330.1	4167.98	82
S39		152	3	OAR3_44553219.1	OAR3_50718262.1	6165.04	110
S39	CON	9	3	s14975.1	s69330.1	476.074	10
S39	UNION	9	3	DU259120_464.1	OAR3_59494261.1	59494.3	1111
S40		126	22	s11593.1	OAR22_36686850.1	14557	280
S40		61	22	OAR22_14956172.1	s08672.1	23753.1	404
S40		49	22	OAR22_12868251.1	OAR22_35805166.1	22936.9	381
S40		702	22	OAR22_9619017.1	s61119.1	25865.1	434
S40		40	22	OAR22_35331669.1	s59235.1	1809.49	33
S40		616	22	s00078.1	OAR22_36946295.1	2082.21	36
S40		90	22	OAR22_33247933.1	s39603.1	3305.97	57
S40		507	22	s06758.1	OAR22_42355166.1	24436.4	466
S40	CON	8	22	OAR22_35331669.1	s61119.1	152.415	6
S40	UNION	8	22	OAR22_9619017.1	OAR22_42355166.1	32736.1	572
S41		126	22	s11593.1	OAR22_36686850.1	14557	280
S41		61	22	OAR22_14956172.1	s08672.1	23753.1	404
S41		49	22	OAR22_12868251.1	OAR22_35805166.1	22936.9	381
S41		702	22	OAR22_9619017.1	s61119.1	25865.1	434
S41		90	22	OAR22_33247933.1	s39603.1	3305.97	57
S41		123	22	OAR22_31412009.1	OAR22_33642320.1	2230.31	47
S41		606	22	OAR22_22756887.1	OAR22_34014868.1	11258	219
S41		507	22	s06758.1	OAR22_42355166.1	24436.4	466
S41	CON	8	22	OAR22_33247933.1	OAR22_33642320.1	394.387	9
S41	UNION	8	22	OAR22_9619017.1	OAR22_42355166.1	32736.1	572
S42		126	22	s11593.1	OAR22_36686850.1	14557	280
S42		61	22	OAR22_14956172.1	s08672.1	23753.1	404
S42		49	22	OAR22_12868251.1	OAR22_35805166.1	22936.9	381
S42		702	22	OAR22_9619017.1	s61119.1	25865.1	434
S42		123	22	OAR22_31412009.1	OAR22_33642320.1	2230.31	47
S42		606	22	OAR22_22756887.1	OAR22_34014868.1	11258	219
S42		507	22	s06758.1	OAR22_42355166.1	24436.4	466
S42		801	22	OAR22_31064768.1	OAR22_33115630.1	2050.86	47
S42	CON	8	22	OAR22_31412009.1	OAR22_33115630.1	1703.62	37
S42	UNION	8	22	OAR22_9619017.1	OAR22_42355166.1	32736.1	572
S46		61	22	OAR22_14956172.1	s08672.1	23753.1	404
S46		49	22	OAR22_12868251.1	OAR22_35805166.1	22936.9	381
S46		702	22	OAR22_9619017.1	s61119.1	25865.1	434
S46		627	22	DU413316_575.1	OAR22_20826797.1	5462.35	55
S46		629	22	DU413316_575.1	OAR22_20826797.1	5462.35	55
S46		90	22	OAR22_13734855.1	s32720.1	4933.37	51
S46		123	22	OAR22_17622066.1	OAR22_28097363.1	10475.3	179
S46		507	22	s06758.1	OAR22_42355166.1	24436.4	466
S46	CON	8	22	s06758.1	s32720.1	749.48	18
S46	UNION	8	22	OAR22_9619017.1	OAR22_42355166.1	32736.1	572
S55		29	18	s42732.1	OAR18_49121315_X.1	4279.05	76
S55		27	18	s42732.1	s05118.1	3781.96	66

S55		45	18	s42732.1	OAR18_47151307.1	2309.04	40
S55		629	18	OAR18_44175536.1	OAR18_48788444.1	4612.91	80
S55		627	18	OAR18_44175536.1	s05118.1	4448.69	77
S55		507	18	DU434490_494.1	OAR18_45912887.1	9548.61	163
S55		123	18	OAR18_42732877.1	OAR18_45912887.1	3180.01	58
S55		702	18	OAR18_43012559.1	OAR18_45763732.1	2751.17	46
S55	CON	8	18	s42732.1	OAR18_45763732.1	921.469	14
S55	UNION	8	18	DU434490_494.1	OAR18_49121315_X.1	12757	222
S59		40	11	s45020.1	OAR11_29271153.1	2783.73	30
S59		507	11	s58684.1	OAR11_29271153.1	3251.87	35
S59		79	11	s21891.1	OAR11_26956590.1	2019.1	35
S59		61	11	OAR11_24444900_X.1	s51636.1	3113.63	49
S59		90	11	s08572.1	OAR11_30551868.1	7951.23	122
S59		123	11	s17310.1	OAR11_29271153.1	9483.93	143
S59		702	11	OAR11_17172323.1	OAR11_33359712.1	16187.4	260
S59		27	11	s04627.1	s73325.1	5587.46	73
S59	CON	8	11	s45020.1	OAR11_26956590.1	469.17	9
S59	UNION	8	11	OAR11_17172323.1	OAR11_33359712.1	16187.4	260
S64		152	8	OAR8_31142913.1	s09657.1	2031.21	41
S64		829	8	OAR8_30120504.1	OAR8_31515043.1	1394.54	32
S64		27	8	OAR8_29706230.1	s32939.1	3476.82	69
S64		40	8	OAR8_29706230.1	OAR8_31515043.1	1808.81	38
S64		123	8	OAR8_29706230.1	OAR8_31515043.1	1808.81	38
S64		126	8	s69453.1	s50983.1	4232.26	88
S64		140	8	OAR8_26330799.1	OAR8_31515043.1	5184.24	103
S64		702	8	OAR8_26793121.1	OAR8_38710483.1	11917.4	231
S64	CON	8	8	OAR8_31142913.1	OAR8_31515043.1	372.13	11
S64	UNION	8	8	OAR8_26330799.1	OAR8_38710483.1	12379.7	239
S65		627	7	s18070.1	OAR7_60745094.1	1667.64	32
S65		629	7	s18070.1	OAR7_60745094.1	1667.64	32
S65		152	7	s18070.1	OAR7_60704536.1	1627.09	31
S65		801	7	OAR7_57790006.1	OAR7_60473904.1	2683.9	54
S65		507	7	OAR7_52043940.1	OAR7_71832636.1	19788.7	355
S65		702	7	OAR7_31685912.1	OAR7_72973471.1	41287.6	769
S65		616	7	OAR7_54780217.1	s36119.1	5323.48	103
S65		29	7	OAR7_56240366.1	OAR7_77667178.1	21426.8	390
S65	CON	8	7	s18070.1	s36119.1	1026.25	20
S65	UNION	8	7	OAR7_31685912.1	OAR7_77667178.1	45981.3	865
S71		140	6	s73122.1	OAR6_49463357.1	26607.7	513
S71		157	6	OAR6_22462972.1	OAR6_23771578.1	1308.61	31
S71		45	6	OAR6_21027868.1	OAR6_27183957.1	6156.09	126
S71		123	6	OAR6_20508343.1	OAR6_23771578.1	3263.24	67
S71		844	6	OAR6_23745238.1	s22136.1	3223.9	68
S71		90	6	OAR6_21784995.1	OAR6_26043279.1	4258.28	85
S71		702	6	OAR6_6278702.1	OAR6_45273992.1	38995.3	741
S71		507	6	OAR6_22984954_X.1	s20604.1	1805.52	41
S71	CON	8	6	OAR6_23745238.1	OAR6_23771578.1	26.34	2
S71	UNION	8	6	OAR6_6278702.1	OAR6_49463357.1	43184.7	833
S76		507	6	s21329.1	OAR6_10484730.1	2028.35	45
S76		627	6	s21329.1	OAR6_10118858.1	1662.48	34

S76		629	6	OAR6_6949639.1	OAR6_10118858.1	3169.22	52
S76		45	6	OAR6_6211452.1	OAR6_10161100.1	3949.65	69
S76		29	6	OAR6_9971239.1	OAR6_13482553.1	3511.31	71
S76		123	6	s48564.1	OAR6_14540611.1	5855.93	118
S76		152	6	OAR6_6254316.1	OAR6_10305227.1	4050.91	72
S76		702	6	OAR6_6278702.1	OAR6_45273992.1	38995.3	741
S76	CON	8	6	OAR6_9971239.1	OAR6_10118858.1	147.619	4
S76	UNION	8	6	OAR6_6211452.1	OAR6_45273992.1	39062.5	743
S77		507	6	s21329.1	OAR6_10484730.1	2028.35	45
S77		627	6	s21329.1	OAR6_10118858.1	1662.48	34
S77		629	6	OAR6_6949639.1	OAR6_10118858.1	3169.22	52
S77		45	6	OAR6_6211452.1	OAR6_10161100.1	3949.65	69
S77		90	6	OAR6_4458962.1	s44871.1	4179.72	70
S77		152	6	OAR6_6254316.1	OAR6_10305227.1	4050.91	72
S77		79	6	s40472.1	s30534.1	2475.33	38
S77		702	6	OAR6_6278702.1	OAR6_45273992.1	38995.3	741
S77	CON	8	6	s21329.1	s30534.1	50.785	2
S77	UNION	8	6	OAR6_4458962.1	OAR6_45273992.1	40815	774
S78		79	5	OAR5_57265711.1	s17800.1	9271.25	138
S78		157	5	OAR5_57173649.1	OAR5_58968886.1	1795.24	31
S78		829	5	s00980.1	OAR5_59243774.1	5486.89	100
S78		152	5	s12940.1	OAR5_61924626.1	14110.5	253
S78		629	5	OAR5_57983508.1	OAR5_71824766.1	13841.3	230
S78		627	5	OAR5_57983508.1	OAR5_66325807.1	8342.3	120
S78		140	5	OAR5_52351898.1	OAR5_58830573.1	6478.68	118
S78		29	5	s54250.1	OAR5_58587196.1	3259.14	61
S78	CON	8	5	OAR5_57983508.1	OAR5_58587196.1	603.688	9
S78	UNION	8	5	s12940.1	OAR5_71824766.1	24010.7	431
S82		27	4	OAR4_73050615.1	OAR4_74539177.1	1488.56	31
S82		507	4	OAR4_73050615.1	OAR4_74539177.1	1488.56	31
S82		79	4	OAR4_72150069.1	OAR4_73570548.1	1420.48	30
S82		140	4	s43361.1	OAR4_74243219.1	2138.92	42
S82		616	4	OAR4_72367529.1	OAR4_78991289.1	6623.76	132
S82		49	4	OAR4_72367529.1	OAR4_74539177.1	2171.65	43
S82		702	4	s58367.1	OAR4_85483475.1	25992.5	514
S82		627	4	OAR4_70656759_X.1	s13803.1	5088.69	101
S82	CON	8	4	OAR4_73050615.1	OAR4_73570548.1	519.933	14
S82	UNION	8	4	s58367.1	OAR4_85483475.1	25992.5	514
S83		29	4	OAR4_24184274.1	OAR4_27320940_X.1	3136.67	66
S83		126	4	OAR4_23761592.1	OAR4_26211342.1	2449.75	51
S83		616	4	OAR4_22143169.1	s60027.1	4198.5	90
S83		606	4	OAR4_15211722_X.1	OAR4_24841202.1	9629.48	193
S83		702	4	OARUn.284_293028.1	OAR4_24251309.1	24251.3	470
S83		801	4	OAR4_23416069_X.1	s49069.1	1172.73	30
S83		49	4	OAR4_22439455.1	OAR4_29317435.1	6877.98	155
S83		152	4	OAR4_20468121.1	OAR4_24841202.1	4373.08	92
S83	CON	8	4	OAR4_24184274.1	OAR4_24251309.1	67.035	3
S83	UNION	8	4	OARUn.284_293028.1	OAR4_29317435.1	29317.4	582
S84		79	4	OAR4_21951630.1	OAR4_23831198.1	1879.57	43
S84		152	4	OAR4_20468121.1	OAR4_24841202.1	4373.08	92

S84		606	4	OAR4_15211722_X.1	OAR4_24841202.1	9629.48	193
S84		702	4	OARUn.284_293028.1	OAR4_24251309.1	24251.3	470
S84		126	4	OAR4_23761592.1	OAR4_26211342.1	2449.75	51
S84		616	4	OAR4_22143169.1	s60027.1	4198.5	90
S84		801	4	OAR4_23416069_X.1	s49069.1	1172.73	30
S84		49	4	OAR4_22439455.1	OAR4_29317435.1	6877.98	155
S84	CON	8	4	OAR4_23761592.1	OAR4_23831198.1	69.606	3
S84	UNION	8	4	OARUn.284_293028.1	OAR4_29317435.1	29317.4	582
S86		157	4	OAR4_21436345.1	OAR4_23013880.1	1577.54	36
S86		844	4	OAR4_20488468.1	s32131.1	1894.04	35
S86		152	4	OAR4_20468121.1	OAR4_24841202.1	4373.08	92
S86		123	4	OAR4_19550445.1	OAR4_22796537.1	3246.09	61
S86		606	4	OAR4_15211722_X.1	OAR4_24841202.1	9629.48	193
S86		702	4	OARUn.284_293028.1	OAR4_24251309.1	24251.3	470
S86		507	4	OAR4_19140942_X.1	OAR4_21780546.1	2639.6	46
S86		27	4	OAR4_21073671.1	OAR4_22765057.1	1691.39	37
S86	CON	8	4	OAR4_21436345.1	OAR4_21780546.1	344.201	9
S86	UNION	8	4	OARUn.284_293028.1	OAR4_24841202.1	24841.2	483
S87		844	4	OAR4_20488468.1	s32131.1	1894.04	35
S87		123	4	OAR4_19550445.1	OAR4_22796537.1	3246.09	61
S87		606	4	OAR4_15211722_X.1	OAR4_24841202.1	9629.48	193
S87		702	4	OARUn.284_293028.1	OAR4_24251309.1	24251.3	470
S87		27	4	OAR4_21073671.1	OAR4_22765057.1	1691.39	37
S87		152	4	OAR4_20468121.1	OAR4_24841202.1	4373.08	92
S87		507	4	OAR4_19140942_X.1	OAR4_21780546.1	2639.6	46
S87		29	4	s70520.1	OAR4_21300620.1	4951.68	88
S87	CON	8	4	OAR4_21073671.1	OAR4_21300620.1	226.949	6
S87	UNION	8	4	OARUn.284_293028.1	OAR4_24841202.1	24841.2	483
S89		507	3	OAR3_179244393.1	OAR3_211362922_X.1	32118.5	604
S89		29	3	OAR3_179244393.1	s26854.1	4664.94	91
S89		606	3	OAR3_177909045.1	s26854.1	6000.29	115
S89		90	3	s41165.1	OAR3_184630061.1	1768.08	33
S89		702	3	OAR3_178121896.1	s16918.1	11927	230
S89		45	3	OAR3_183605277.1	s52959.1	3069.59	58
S89		61	3	s38401.1	OAR3_183605277.1	2795.96	51
S89		844	3	OAR3_182939964.1	OAR3_186988175.1	4048.21	73
S89	CON	8	3	OAR3_183605277.1	OAR3_183605277.1	0	1
S89	UNION	8	3	OAR3_177909045.1	OAR3_211362922_X.1	33453.9	628
S93		507	3	OAR3_43871305.1	OAR3_52722619.1	8851.31	160
S93		49	3	OAR3_43700480.1	OAR3_50145205.1	6444.73	118
S93		627	3	OAR3_44553219.1	OAR3_46866444.1	2313.22	46
S93		629	3	OAR3_44553219.1	OAR3_46866444.1	2313.22	46
S93		702	3	DU259120_464.1	OAR3_59494261.1	59494.3	1111
S93		152	3	OAR3_44553219.1	OAR3_50718262.1	6165.04	110
S93		606	3	s14975.1	OAR3_47755179.1	3083.7	63
S93		45	3	OAR3_45905654.1	OAR3_47595933.1	1690.28	35
S93	CON	8	3	OAR3_45905654.1	OAR3_46866444.1	960.79	19
S93	UNION	8	3	DU259120_464.1	OAR3_59494261.1	59494.3	1111
S94		556	2	OAR2_238010227.1	OAR2_240388073.1	2377.85	48
S94		123	2	OAR2_235417778.1	OAR2_244336911.1	8919.13	185

S94		507	2	OAR2_234395013.1	OAR2_240388073.1	5993.06	127
S94		627	2	OAR2_238165550.1	OAR2_246045571.1	7880.02	156
S94		629	2	OAR2_238165550.1	OAR2_241065735.1	2900.18	59
S94		79	2	OAR2_236034019.1	OAR2_241969339.1	5935.32	125
S94		61	2	OAR2_239847244.1	OAR2_242019441.1	2172.2	44
S94		29	2	OAR2_238444943.1	s43939.1	16224.6	314
S94	CON	8	2	OAR2_239847244.1	OAR2_240388073.1	540.829	11
S94	UNION	8	2	OAR2_234395013.1	s43939.1	20274.5	401
S95		627	2	OAR2_238165550.1	OAR2_246045571.1	7880.02	156
S95		629	2	OAR2_238165550.1	OAR2_241065735.1	2900.18	59
S95		556	2	OAR2_238010227.1	OAR2_240388073.1	2377.85	48
S95		79	2	OAR2_236034019.1	OAR2_241969339.1	5935.32	125
S95		123	2	OAR2_235417778.1	OAR2_244336911.1	8919.13	185
S95		507	2	OAR2_234395013.1	OAR2_240388073.1	5993.06	127
S95		29	2	OAR2_238444943.1	s43939.1	16224.6	314
S95		45	2	OAR2_235533135.1	DU260201_585.1	2944.56	66
S95	CON	8	2	OAR2_238444943.1	DU260201_585.1	32.748	2
S95	UNION	8	2	OAR2_234395013.1	s43939.1	20274.5	401
S96		507	2	OAR2_134615652.1	OAR2_136209364.1	1593.71	30
S96		90	2	s72460.1	OAR2_136209364.1	1755.22	34
S96		140	2	s72460.1	OAR2_136086521.1	1632.38	32
S96		29	2	OAR2_132531523.1	OAR2_136140871.1	3609.35	80
S96		616	2	OAR2_129409414.1	OAR2_135326401.1	5916.99	129
S96		123	2	OAR2_128971562.1	OAR2_135421095.1	6449.53	141
S96		61	2	OAR2_126263077.1	OAR2_134940115.1	8677.04	181
S96		126	2	OAR2_116277389.1	OAR2_134940115.1	18662.7	353
S96	CON	8	2	OAR2_134615652.1	OAR2_134940115.1	324.463	7
S96	UNION	8	2	OAR2_116277389.1	OAR2_136209364.1	19932	376
S97		29	2	OAR2_132531523.1	OAR2_136140871.1	3609.35	80
S97		45	2	OAR2_130020916.1	OAR2_133530461.1	3509.55	78
S97		616	2	OAR2_129409414.1	OAR2_135326401.1	5916.99	129
S97		123	2	OAR2_128971562.1	OAR2_135421095.1	6449.53	141
S97		507	2	s20121.1	OAR2_134249434.1	7112.48	154
S97		844	2	s20121.1	OAR2_133793734.1	6656.78	145
S97		61	2	OAR2_126263077.1	OAR2_134940115.1	8677.04	181
S97		126	2	OAR2_116277389.1	OAR2_134940115.1	18662.7	353
S97	CON	8	2	OAR2_132531523.1	OAR2_133530461.1	998.938	28
S97	UNION	8	2	OAR2_116277389.1	OAR2_136140871.1	19863.5	375
S99		45	2	OAR2_130020916.1	OAR2_133530461.1	3509.55	78
S99		616	2	OAR2_129409414.1	OAR2_135326401.1	5916.99	129
S99		123	2	OAR2_128971562.1	OAR2_135421095.1	6449.53	141
S99		507	2	s20121.1	OAR2_134249434.1	7112.48	154
S99		844	2	s20121.1	OAR2_133793734.1	6656.78	145
S99		61	2	OAR2_126263077.1	OAR2_134940115.1	8677.04	181
S99		29	2	OAR2_119004082.1	OAR2_132080120.1	13076	229
S99		126	2	OAR2_116277389.1	OAR2_134940115.1	18662.7	353
S99	CON	8	2	OAR2_130020916.1	OAR2_132080120.1	2059.2	40
S99	UNION	8	2	OAR2_116277389.1	OAR2_135421095.1	19143.7	362
S100		616	2	OAR2_129409414.1	OAR2_135326401.1	5916.99	129
S100		123	2	OAR2_128971562.1	OAR2_135421095.1	6449.53	141

S100		507	2	s20121.1	OAR2_134249434.1	7112.48	154
S100		844	2	s20121.1	OAR2_133793734.1	6656.78	145
S100		61	2	OAR2_126263077.1	OAR2_134940115.1	8677.04	181
S100		29	2	OAR2_119004082.1	OAR2_132080120.1	13076	229
S100		126	2	OAR2_116277389.1	OAR2_134940115.1	18662.7	353
S100		27	2	OAR2_115851252.1	OAR2_129745095.1	13893.8	249
S100	CON	8	2	OAR2_129409414.1	OAR2_129745095.1	335.681	8
S100	UNION	8	2	OAR2_115851252.1	OAR2_135421095.1	19569.8	372
S101		844	2	OAR2_63699523.1	s38855.1	1651.43	37
S101		123	2	OAR2_63165309.1	s38855.1	2185.64	46
S101		61	2	OAR2_63165309.1	s38855.1	2185.64	46
S101		49	2	OAR2_61907594.1	OAR2_74652390.1	12744.8	265
S101		40	2	OAR2_63943896.1	s59858.1	2994.2	62
S101		29	2	OAR2_63911376.1	OAR2_66880170.1	2968.79	62
S101		507	2	OAR2_63911376.1	OAR2_65914681.1	2003.31	43
S101		556	2	OAR2_62824297.1	OAR2_64484840.1	1660.54	36
S101	CON	8	2	OAR2_63943896.1	OAR2_64484840.1	540.944	13
S101	UNION	8	2	OAR2_61907594.1	OAR2_74652390.1	12744.8	265
S102		702	22	OAR22_5872287.1	OAR22_7511031.1	1638.74	34
S102		152	22	OAR22_5771792.1	OAR22_7804834.1	2033.04	41
S102		45	22	OAR22_5711915.1	OAR22_8033581.1	2321.67	47
S102		507	22	s68573.1	OAR22_8623862.1	8623.86	128
S102		126	22	OAR22_1250324.1	OAR22_7588884.1	6338.56	102
S102		27	22	OAR22_7348996.1	OAR22_8955081.1	1606.09	32
S102		616	22	OAR22_5595255.1	OAR22_7399531.1	1804.28	38
S102	CON	7	22	OAR22_7348996.1	OAR22_7399531.1	50.535	2
S102	UNION	7	22	s68573.1	OAR22_8955081.1	8955.08	134
S105		702	22	OAR22_5872287.1	OAR22_7511031.1	1638.74	34
S105		152	22	OAR22_5771792.1	OAR22_7804834.1	2033.04	41
S105		45	22	OAR22_5711915.1	OAR22_8033581.1	2321.67	47
S105		829	22	OAR22_4104153.1	OAR22_6064565.1	1960.41	39
S105		507	22	s68573.1	OAR22_8623862.1	8623.86	128
S105		126	22	OAR22_1250324.1	OAR22_7588884.1	6338.56	102
S105		616	22	OAR22_5595255.1	OAR22_7399531.1	1804.28	38
S105	CON	7	22	OAR22_5872287.1	OAR22_6064565.1	192.278	5
S105	UNION	7	22	s68573.1	OAR22_8623862.1	8623.86	128
S106		45	21	DU196132_525.1	s51905.1	2968.99	32
S106		844	21	s75988.1	OAR21_54464294.1	7093.13	95
S106		123	21	s30026.1	s11366.1	5205.16	71
S106		507	21	OAR21_43646542.1	s49922.1	8141.23	108
S106		702	21	OAR21_38087037.1	s49922.1	13700.7	160
S106		627	21	s03044.1	OAR21_54143578.1	3949.9	61
S106		90	21	s23363.1	s53160.1	4496.72	61
S106	CON	7	21	s23363.1	s49922.1	808.117	9
S106	UNION	7	21	OAR21_38087037.1	s53160.1	17389.3	212
S107		507	21	OAR21_43646542.1	s49922.1	8141.23	108
S107		140	21	s22832.1	s67111.1	5518.15	45
S107		27	21	s25809.1	s13923.1	11828.3	168
S107		629	21	DU383863_376.1	s61876.1	2892.57	40
S107		90	21	s62516.1	s75876.1	6474.15	60

S107		702	21	OAR21_38087037.1	s49922.1	13700.7	160
S107		616	21	DU383863_376.1	OAR21_45728443_X.1	1979.97	30
S107	CON	7	21	DU383863_376.1	s13923.1	502.999	10
S107	UNION	7	21	s25809.1	s49922.1	19364.6	265
S108		829	21	s75799.1	s08492.1	2047.17	32
S108		79	21	OAR21_33044180.1	s61235.1	3111.66	57
S108		45	21	OAR21_33336974.1	OAR21_36484190_X.1	3147.22	53
S108		606	21	OAR21_21865772.1	OAR21_36571389.1	14705.6	232
S108		27	21	s25809.1	s13923.1	11828.3	168
S108		90	21	s75804.1	s55303.1	6697.66	125
S108		140	21	s75799.1	OAR21_38087037.1	2579.68	44
S108	CON	7	21	s75799.1	s61235.1	648.482	5
S108	UNION	7	21	OAR21_21865772.1	s13923.1	22385.7	328
S111		629	19	OAR19_13260250.1	OAR19_15065899.1	1805.65	32
S111		627	19	s40605.1	OAR19_15065899.1	2211.24	41
S111		61	19	OAR19_10268706.1	s25958.1	4848.61	90
S111		152	19	OAR19_9837128.1	s12224.1	3663.3	70
S111		79	19	OAR19_11147748.1	s12224.1	2352.68	44
S111		45	19	OAR19_3773707.1	OAR19_13260250.1	9486.54	172
S111		702	19	OAR19_7253713.1	s27711.1	16961.5	313
S111	CON	7	19	OAR19_13260250.1	OAR19_13260250.1	0	1
S111	UNION	7	19	OAR19_3773707.1	s27711.1	20441.5	368
S112		29	18	s42732.1	OAR18_49121315_X.1	4279.05	76
S112		27	18	s42732.1	s05118.1	3781.96	66
S112		45	18	s42732.1	OAR18_47151307.1	2309.04	40
S112		629	18	OAR18_44175536.1	OAR18_48788444.1	4612.91	80
S112		627	18	OAR18_44175536.1	s05118.1	4448.69	77
S112		79	18	OAR18_47094663.1	OAR18_48997274.1	1902.61	34
S112		90	18	s65781.1	OAR18_49121315_X.1	2310.04	44
S112	CON	7	18	OAR18_47094663.1	OAR18_47151307.1	56.644	2
S112	UNION	7	18	OAR18_44175536.1	OAR18_49121315_X.1	4945.78	87
S113		79	17	OAR17_55061923.1	OAR17_62559974.1	7498.05	120
S113		829	17	s44509.1	s69675.1	5770.21	91
S113		507	17	s47176.1	s49020.1	26754.9	433
S113		702	17	OAR17_36966397.1	OAR17_74039265.1	37072.9	600
S113		556	17	s69675.1	s13013.1	2066.88	35
S113		627	17	s46474.1	OAR17_65490341.1	8079.56	133
S113		61	17	s36776.1	OAR17_61822807.1	2992.36	51
S113	CON	7	17	s69675.1	s69675.1	0	1
S113	UNION	7	17	OAR17_36966397.1	OAR17_74039265.1	37072.9	600
S114		27	17	OAR17_56962976.1	DU493579_579.1	2311.53	34
S114		79	17	OAR17_55061923.1	OAR17_62559974.1	7498.05	120
S114		829	17	s44509.1	s69675.1	5770.21	91
S114		507	17	s47176.1	s49020.1	26754.9	433
S114		702	17	OAR17_36966397.1	OAR17_74039265.1	37072.9	600
S114		627	17	s46474.1	OAR17_65490341.1	8079.56	133
S114		61	17	s36776.1	OAR17_61822807.1	2992.36	51
S114	CON	7	17	s36776.1	DU493579_579.1	444.061	7
S114	UNION	7	17	OAR17_36966397.1	OAR17_74039265.1	37072.9	600

S115		27	17	OAR17_56962976.1	DU493579_579.1	2311.53	34
S115		79	17	OAR17_55061923.1	OAR17_62559974.1	7498.05	120
S115		829	17	s44509.1	s69675.1	5770.21	91
S115		152	17	s44509.1	s33211.1	2693.86	44
S115		507	17	s47176.1	s49020.1	26754.9	433
S115		702	17	OAR17_36966397.1	OAR17_74039265.1	37072.9	600
S115		627	17	s46474.1	OAR17_65490341.1	8079.56	133
S115	CON	7	17	s46474.1	s33211.1	306.569	8
S115	UNION	7	17	OAR17_36966397.1	OAR17_74039265.1	37072.9	600
S116		40	16	s38913.1	OAR16_18865349.1	5499.5	103
S116		27	16	s38913.1	s01680.1	2273.09	44
S116		844	16	OAR16_13101603.1	OAR16_16543821.1	3442.22	62
S116		90	16	OAR16_12311919.1	s45825.1	1470.32	33
S116		123	16	OAR16_12276858.1	OAR16_13935744.1	1658.89	36
S116		702	16	OAR16_11050320.1	OAR16_20062365.1	9012.05	173
S116		29	16	OAR16_11445590.1	OAR16_15329984.1	3884.39	78
S116	CON	7	16	s38913.1	s45825.1	416.394	10
S116	UNION	7	16	OAR16_11050320.1	OAR16_20062365.1	9012.05	173
S118		27	15	OAR15_75169402.1	OAR15_81649572.1	6480.17	139
S118		45	15	s32676.1	OAR15_76419512.1	2091.67	36
S118		29	15	OAR15_73660126.1	OAR15_75673025.1	2012.9	35
S118		829	15	OAR15_73610660.1	OAR15_77297861.1	3687.2	75
S118		507	15	s65800.1	s24876.1	2625.56	43
S118		606	15	s60467.1	s57222.1	4219.52	71
S118		702	15	OAR15_71907029.1	OAR15_76144996.1	4237.97	71
S118	CON	7	15	OAR15_75169402.1	OAR15_75673025.1	503.623	9
S118	UNION	7	15	OAR15_71907029.1	OAR15_81649572.1	9742.54	193
S129		40	15	OAR15_43565431.1	s34379.1	9826.51	115
S129		801	15	s71520.1	OAR15_45313629.1	2299.97	46
S129		507	15	s71520.1	OAR15_44828179.1	1814.52	35
S129		702	15	s45101.1	OAR15_45753226.1	3043.09	59
S129		140	15	OAR15_41964058.1	OAR15_45753226.1	3789.17	74
S129		90	15	OAR15_41465316.1	OAR15_46781842.1	5316.53	97
S129		627	15	OAR15_41465316.1	OAR15_53192586.1	11727.3	150
S129	CON	7	15	OAR15_43565431.1	OAR15_44828179.1	1262.75	24
S129	UNION	7	15	OAR15_41465316.1	s34379.1	11926.6	153
S130		49	14	s42994.1	s41160.1	2649.81	37
S130		629	14	OAR14_53138348.1	OAR14_55447588.1	2309.24	39
S130		123	14	s16936.1	OAR14_55447588.1	2612.12	44
S130		126	14	s39024.1	s04653.1	4645.22	62
S130		90	14	OAR14_55447588.1	OAR14_58537282.1	3089.69	43
S130		140	14	OAR14_54070310.1	OAR14_56421333.1	2351.02	30
S130		152	14	OAR14_52929077.1	OAR14_56868551.1	3939.47	57
S130	CON	7	14	OAR14_55447588.1	OAR14_55447588.1	0	1
S130	UNION	7	14	s39024.1	OAR14_58537282.1	6386.1	91
S131		49	14	s42994.1	s41160.1	2649.81	37
S131		629	14	OAR14_53138348.1	OAR14_55447588.1	2309.24	39
S131		507	14	OAR14_52929077.1	s10360.1	1894.68	32
S131		123	14	s16936.1	OAR14_55447588.1	2612.12	44
S131		126	14	s39024.1	s04653.1	4645.22	62

S131		152	14	OAR14_52929077.1	OAR14_56868551.1	3939.47	57
S131		140	14	OAR14_54070310.1	OAR14_56421333.1	2351.02	30
S131	CON	7	14	OAR14_54070310.1	s10360.1	753.445	11
S131	UNION	7	14	s39024.1	OAR14_56868551.1	4717.37	64
S132		29	13	s62936.1	OAR13_35198687.1	9473.28	175
S132		606	13	OAR13_22595627.1	OAR13_26635884.1	4040.26	80
S132		629	13	OAR13_22595627.1	OAR13_26635884.1	4040.26	80
S132		49	13	OAR13_21176498.1	OAR13_26177858.1	5001.36	93
S132		829	13	s29601.1	OAR13_37876562.1	14843.5	275
S132		616	13	OAR13_19366874.1	s47163.1	7083.23	139
S132		123	13	s43905.1	OAR13_26635884.1	6436.53	127
S132	CON	7	13	s62936.1	OAR13_26177858.1	452.455	10
S132	UNION	7	13	OAR13_19366874.1	OAR13_37876562.1	18509.7	349
S133		40	12	OAR12_65650955.1	s59916.1	1646.15	33
S133		606	12	OAR12_64858268.1	s09441.1	2113.07	42
S133		627	12	OAR12_64690849.1	s22773.1	2642.42	52
S133		629	12	OAR12_64690849.1	s22773.1	2642.42	52
S133		702	12	s22960.1	s60710.1	58755.9	1088
S133		152	12	s30614.1	s20113.1	2334.94	49
S133		27	12	OAR12_64944672.1	OAR12_67663336.1	2718.66	55
S133	CON	7	12	OAR12_65650955.1	s20113.1	1148.72	26
S133	UNION	7	12	s22960.1	s60710.1	58755.9	1088
S134		79	11	s21891.1	OAR11_26956590.1	2019.1	35
S134		61	11	OAR11_24444900_X.1	s51636.1	3113.63	49
S134		126	11	s27941.1	s31598.1	2423.43	41
S134		90	11	s08572.1	OAR11_30551868.1	7951.23	122
S134		702	11	OAR11_17172323.1	OAR11_33359712.1	16187.4	260
S134		123	11	s17310.1	OAR11_29271153.1	9483.93	143
S134		27	11	s04627.1	s73325.1	5587.46	73
S134	CON	7	11	s21891.1	s31598.1	180.155	5
S134	UNION	7	11	OAR11_17172323.1	OAR11_33359712.1	16187.4	260
S135		45	10	OAR10_35722333.1	OAR10_57396545.1	21674.2	405
S135		123	10	OAR10_35722333.1	OAR10_41054879.1	5332.55	89
S135		702	10	s49567.1	OAR10_48462141.1	48462.1	914
S135		829	10	OAR10_33047233.1	OAR10_51969493.1	18922.3	352
S135		801	10	s38022.1	OAR10_41435807.1	5825.97	101
S135		606	10	OAR10_35692572.1	OAR10_37319785_X.1	1627.21	30
S135		844	10	s18518.1	s35062.1	1609.48	32
S135	CON	7	10	s18518.1	OAR10_37319785_X.1	1242.86	23
S135	UNION	7	10	s49567.1	OAR10_57396545.1	57396.5	1095
S137		40	10	OAR10_28598904.1	s18834.1	1227.84	31
S137		507	10	OAR10_28598904.1	s18834.1	1227.84	31
S137		627	10	OAR10_25752393.1	OAR10_29546872.1	3794.48	85
S137		629	10	OAR10_25752393.1	OAR10_29546872.1	3794.48	85
S137		152	10	OAR10_25624621.1	OAR10_29538398.1	3913.78	87
S137		702	10	s49567.1	OAR10_48462141.1	48462.1	914
S137		844	10	OAR10_17703888.1	s09622.1	13279.3	268
S137	CON	7	10	OAR10_28598904.1	OAR10_29538398.1	939.494	25
S137	UNION	7	10	s49567.1	OAR10_48462141.1	48462.1	914

S138		61	9	OAR9_81293209.1	s03389.1	2086.52	36
S138		152	9	s68078.1	OAR9_82953999.1	2041.55	36
S138		627	9	s68078.1	OAR9_82953999.1	2041.55	36
S138		629	9	s68078.1	OAR9_82953999.1	2041.55	36
S138		702	9	OAR9_38323108.1	OAR9_96544082.1	58221	1087
S138		140	9	s10879.1	s05406.1	2485.9	47
S138		123	9	OAR9_80918118.1	OAR9_82953999.1	2035.88	35
S138	CON	7	9	OAR9_81293209.1	s05406.1	1390.51	22
S138	UNION	7	9	OAR9_38323108.1	OAR9_96544082.1	58221	1087
S139		140	8	s61158.1	OAR8_52791160.1	9209.77	166
S139		126	8	OAR8_42338337.1	OAR8_45925190.1	3586.85	72
S139		627	8	s28792.1	s61158.1	2639.09	53
S139		629	8	s28792.1	s61158.1	2639.09	53
S139		829	8	s66809.1	OAR8_49120824.1	9189.29	175
S139		123	8	OAR8_34202419.1	OAR8_45617687.1	11415.3	218
S139		45	8	OAR8_41415390.1	OAR8_43915897.1	2500.51	46
S139	CON	7	8	s61158.1	s61158.1	0	1
S139	UNION	7	8	OAR8_34202419.1	OAR8_52791160.1	18588.7	347
S140		801	7	OAR7_71047974.1	OAR7_73062925.1	2014.95	34
S140		606	7	OAR7_68768125.1	s56478.1	3002.79	51
S140		616	7	s45070.1	s56478.1	3604.95	59
S140		45	7	s45070.1	s56478.1	3604.95	59
S140		702	7	OAR7_31685912.1	OAR7_72973471.1	41287.6	769
S140		507	7	OAR7_52043940.1	OAR7_71832636.1	19788.7	355
S140		29	7	OAR7_56240366.1	OAR7_77667178.1	21426.8	390
S140	CON	7	7	OAR7_71047974.1	s56478.1	722.943	15
S140	UNION	7	7	OAR7_31685912.1	OAR7_77667178.1	45981.3	865
S141		606	7	OAR7_68768125.1	s56478.1	3002.79	51
S141		616	7	s45070.1	s56478.1	3604.95	59
S141		45	7	s45070.1	s56478.1	3604.95	59
S141		702	7	OAR7_31685912.1	OAR7_72973471.1	41287.6	769
S141		507	7	OAR7_52043940.1	OAR7_71832636.1	19788.7	355
S141		29	7	OAR7_56240366.1	OAR7_77667178.1	21426.8	390
S141		123	7	s03425.1	OAR7_70071978.1	2562.47	40
S141	CON	7	7	OAR7_68768125.1	OAR7_70071978.1	1303.85	16
S141	UNION	7	7	OAR7_31685912.1	OAR7_77667178.1	45981.3	865
S142		29	6	OAR6_121651498.1	OAR6_124727445.1	3075.95	59
S142		844	6	OAR6_121433524.1	OAR6_123886396.1	2452.87	43
S142		801	6	OAR6_120674641.1	s42254.1	3373.02	61
S142		45	6	OAR6_119942135.1	OAR6_122276062.1	2333.93	39
S142		507	6	OAR6_119942135.1	OAR6_122276062.1	2333.93	39
S142		61	6	OAR6_119546066.1	s55094.1	2796.93	50
S142		140	6	s08027.1	s18701.1	4432.34	84
S142	CON	7	6	OAR6_121651498.1	OAR6_122276062.1	624.564	5
S142	UNION	7	6	OAR6_119546066.1	OAR6_124727445.1	5181.38	102
S143		61	6	s40686.1	s10844.1	3055.35	42
S143		844	6	s59122.1	OAR6_119125205.1	3781.3	51
S143		29	6	s03175.1	s10844.1	3259.64	45
S143		606	6	s25188.1	s10844.1	3280.93	46
S143		140	6	s40686.1	s19234.1	3575.59	48

S143		616	6	s40686.1	s65263.1	3530.51	47
S143		702	6	s03175.1	s49558.1	3896.24	53
S143	CON	7	6	s40686.1	s10844.1	3055.35	42
S143	UNION	7	6	s25188.1	s49558.1	3917.52	54
S144		123	5	OAR5_67426945.1	OAR5_70029869.1	2602.92	48
S144		126	5	s52618.1	OAR5_72565538.1	7244.72	139
S144		29	5	s07518.1	OAR5_68302417.1	3681.04	76
S144		90	5	DU444709_372.1	OAR5_68350537.1	7925.86	122
S144		801	5	s11947.1	OAR5_69909638.1	1818.53	34
S144		27	5	OAR5_63370705.1	s36664.1	5079.16	100
S144		629	5	OAR5_57983508.1	OAR5_71824766.1	13841.3	230
S144	CON	7	5	s11947.1	OAR5_68302417.1	211.304	4
S144	UNION	7	5	OAR5_57983508.1	OAR5_72565538.1	14582	241
S145		627	3	OAR3_206227595.1	OAR3_209042148.1	2814.55	62
S145		629	3	OAR3_206227595.1	OAR3_209042148.1	2814.55	62
S145		829	3	OAR3_205737877.1	OAR3_208666709.1	2928.83	65
S145		507	3	OAR3_179244393.1	OAR3_211362922_X.1	32118.5	604
S145		606	3	OAR3_208666709.1	s05793.1	2048.02	40
S145		123	3	OAR3_207289038.1	OAR3_208794039.1	1505	31
S145		157	3	OAR3_206856083.1	OAR3_209162669.1	2306.59	49
S145	CON	7	3	OAR3_208666709.1	OAR3_208666709.1	0	1
S145	UNION	7	3	OAR3_179244393.1	OAR3_211362922_X.1	32118.5	604
S146		45	3	OAR3_25294644.1	OAR3_27011374.1	1716.73	37
S146		123	3	OAR3_24592746.1	s13240.1	2053.62	40
S146		27	3	OAR3_24402850.1	s28199.1	1947.6	38
S146		29	3	OAR3_24402850.1	s19023.1	1577.89	31
S146		152	3	OAR3_24047322.1	OAR3_27322763.1	3275.44	68
S146		507	3	s49768.1	OAR3_28567790.1	9061.66	193
S146		702	3	DU259120_464.1	OAR3_59494261.1	59494.3	1111
S146	CON	7	3	OAR3_25294644.1	s19023.1	686.1	14
S146	UNION	7	3	DU259120_464.1	OAR3_59494261.1	59494.3	1111
S149		123	3	OAR3_24592746.1	s13240.1	2053.62	40
S149		27	3	OAR3_24402850.1	s28199.1	1947.6	38
S149		29	3	OAR3_24402850.1	s19023.1	1577.89	31
S149		152	3	OAR3_24047322.1	OAR3_27322763.1	3275.44	68
S149		90	3	OAR3_22936544.1	s54478.1	1985.68	39
S149		702	3	DU259120_464.1	OAR3_59494261.1	59494.3	1111
S149		507	3	s49768.1	OAR3_28567790.1	9061.66	193
S149	CON	7	3	OAR3_24592746.1	s54478.1	329.479	5
S149	UNION	7	3	DU259120_464.1	OAR3_59494261.1	59494.3	1111
S151		27	3	OAR3_24402850.1	s28199.1	1947.6	38
S151		29	3	OAR3_24402850.1	s19023.1	1577.89	31
S151		152	3	OAR3_24047322.1	OAR3_27322763.1	3275.44	68
S151		90	3	OAR3_22936544.1	s54478.1	1985.68	39
S151		79	3	OAR3_17718190.1	OAR3_24559969.1	6841.78	153
S151		702	3	DU259120_464.1	OAR3_59494261.1	59494.3	1111
S151		507	3	s49768.1	OAR3_28567790.1	9061.66	193
S151	CON	7	3	OAR3_24402850.1	OAR3_24559969.1	157.119	5
S151	UNION	7	3	DU259120_464.1	OAR3_59494261.1	59494.3	1111

S152		29	2	s20556.1	s56215.1	1280.3	30
S152		616	2	OAR2_172094342.1	OAR2_175943638.1	3849.3	82
S152		829	2	OAR2_171930761.1	OAR2_175660582.1	3729.82	83
S152		844	2	OAR2_171247972.1	OAR2_172663891.1	1415.92	32
S152		123	2	OAR2_167959232.1	OAR2_176091475.1	8132.24	171
S152		507	2	OAR2_167852063.1	OAR2_173821801.1	5969.74	125
S152		90	2	OAR2_170073165.1	OAR2_173940252.1	3867.09	87
S152	CON	7	2	s20556.1	OAR2_172663891.1	68.911	3
S152	UNION	7	2	OAR2_167852063.1	OAR2_176091475.1	8239.41	174
S154		829	2	OAR2_171930761.1	OAR2_175660582.1	3729.82	83
S154		844	2	OAR2_171247972.1	OAR2_172663891.1	1415.92	32
S154		123	2	OAR2_167959232.1	OAR2_176091475.1	8132.24	171
S154		616	2	OAR2_172094342.1	OAR2_175943638.1	3849.3	82
S154		90	2	OAR2_170073165.1	OAR2_173940252.1	3867.09	87
S154		140	2	OAR2_169232830.1	OAR2_172379160.1	3146.33	68
S154		507	2	OAR2_167852063.1	OAR2_173821801.1	5969.74	125
S154	CON	7	2	OAR2_172094342.1	OAR2_172379160.1	284.818	5
S154	UNION	7	2	OAR2_167852063.1	OAR2_176091475.1	8239.41	174
S155		123	2	OAR2_140051989_X.1	OAR2_144244441.1	4192.45	95
S155		29	2	s49391.1	OAR2_141900838.1	2573.2	56
S155		45	2	OAR2_141764296.1	OAR2_143949087.1	2184.79	45
S155		829	2	OAR2_141723000.1	DU292901_688.1	2328.84	49
S155		140	2	OAR2_140086515.1	s51093.1	1777.37	43
S155		40	2	OAR2_139988851.1	OAR2_143429707_X.1	3440.86	78
S155		702	2	s54108.1	OAR2_162167662.1	23623.2	482
S155	CON	7	2	OAR2_141764296.1	s51093.1	99.592	2
S155	UNION	7	2	s54108.1	OAR2_162167662.1	23623.2	482
S157		27	2	OAR2_90226425.1	s52970.1	1662.46	34
S157		45	2	OAR2_90110357.1	s52970.1	1778.53	36
S157		606	2	s14873.1	OAR2_96066817.1	6724.02	118
S157		49	2	OAR2_90239503.1	OAR2_96436987_X.1	6197.48	106
S157		90	2	OAR2_90239503.1	s66693.1	3509.49	72
S157		79	2	OAR2_90239503.1	OAR2_91821070.1	1581.57	31
S157		801	2	OAR2_91185502.1	s48473.1	1817.05	39
S157	CON	7	2	OAR2_91185502.1	OAR2_91821070.1	635.568	16
S157	UNION	7	2	s14873.1	OAR2_96436987_X.1	7094.19	123
S158		627	1	s09614.1	s18847.1	2947.5	42
S158		629	1	s09614.1	s18847.1	2947.5	42
S158		606	1	s35781.1	OAR1_287434870.1	2608.39	48
S158		61	1	s35781.1	OAR1_287434870.1	2608.39	48
S158		29	1	OAR1_283569518.1	s39199.1	5956.3	95
S158		507	1	s22571.1	s56772.1	15398.3	291
S158		702	1	OAR1_282017475.1	s67262.1	9551.48	171
S158	CON	7	1	s09614.1	OAR1_287434870.1	1752.35	32
S158	UNION	7	1	s22571.1	s56772.1	15398.3	291
S160		90	1	OAR1_124078281.1	OAR1_138926518.1	14848.2	257
S160		606	1	OAR1_120032190.1	s74762.1	6498.59	124
S160		702	1	s14189.1	s40751.1	42088.8	712
S160		45	1	s37607.1	s04671.1	16346.9	247
S160		123	1	s53597.1	OAR1_153662211.1	35798.6	631

S160		27	1	OAR1_119613390.1	s02795.1	5219.57	100
S160		79	1	s75317.1	s66718.1	6978.38	93
S160	CON	7	1	s75317.1	s02795.1	207.814	5
S160	UNION	7	1	s14189.1	OAR1_153662211.1	45938.1	791
S161		90	1	OAR1_121425150.1	OAR1_123217898.1	1792.75	30
S161		606	1	OAR1_120032190.1	s74762.1	6498.59	124
S161		61	1	OAR1_117978374.1	OAR1_122656397.1	4678.02	78
S161		45	1	s37607.1	s04671.1	16346.9	247
S161		702	1	s14189.1	s40751.1	42088.8	712
S161		123	1	s53597.1	OAR1_153662211.1	35798.6	631
S161		27	1	OAR1_119613390.1	s02795.1	5219.57	100
S161	CON	7	1	OAR1_121425150.1	OAR1_122656397.1	1231.25	19
S161	UNION	7	1	s14189.1	OAR1_153662211.1	45938.1	791
S165		829	1	OAR1_117951301.1	OAR1_120197222.1	2245.92	34
S165		702	1	s14189.1	s40751.1	42088.8	712
S165		606	1	OAR1_120032190.1	s74762.1	6498.59	124
S165		61	1	OAR1_117978374.1	OAR1_122656397.1	4678.02	78
S165		45	1	s37607.1	s04671.1	16346.9	247
S165		123	1	s53597.1	OAR1_153662211.1	35798.6	631
S165		27	1	OAR1_119613390.1	s02795.1	5219.57	100
S165	CON	7	1	OAR1_120032190.1	OAR1_120197222.1	165.032	5
S165	UNION	7	1	s14189.1	OAR1_153662211.1	45938.1	791
S176		49	1	OAR1_113402761.1	s75030.1	4288.36	49
S176		556	1	OAR1_112929344.1	OAR1_117762259.1	4832.91	61
S176		40	1	s69088.1	OAR1_114069491_X.1	2477.52	40
S176		801	1	OAR1_110989185.1	s10939.1	7522.21	114
S176		507	1	OAR1_109879519.1	OAR1_114069491_X.1	4189.97	75
S176		79	1	OAR1_108764473.1	s01025.1	4913.19	93
S176		702	1	s14189.1	s40751.1	42088.8	712
S176	CON	7	1	OAR1_113402761.1	s01025.1	274.904	3
S176	UNION	7	1	s14189.1	s40751.1	42088.8	712
S180		801	1	OAR1_110989185.1	s10939.1	7522.21	114
S180		507	1	OAR1_109879519.1	OAR1_114069491_X.1	4189.97	75
S180		45	1	OAR1_109726063.1	s17035.1	1601.31	34
S180		90	1	OAR1_108774968.1	s74483.1	2594.22	49
S180		79	1	OAR1_108764473.1	s01025.1	4913.19	93
S180		27	1	s00446.1	s04333.1	4130.65	80
S180		702	1	s14189.1	s40751.1	42088.8	712
S180	CON	7	1	OAR1_110989185.1	s17035.1	338.19	8
S180	UNION	7	1	s14189.1	s40751.1	42088.8	712
S181		40	1	s40404.1	s30526.1	3182.16	55
S181		152	1	s40404.1	s01842.1	3048.15	51
S181		123	1	s67590.1	s45674.1	11170	204
S181		45	1	s45674.1	OAR1_37896369_X.1	3544.34	59
S181		829	1	s70438.1	s26451.1	1459.78	31
S181		702	1	OAR1_7717464.1	OAR1_58938560.1	51221.1	934
S181		61	1	OAR1_32040848.1	OAR1_37282541.1	5241.69	99
S181	CON	7	1	s45674.1	s45674.1	0	1
S181	UNION	7	1	OAR1_7717464.1	OAR1_58938560.1	51221.1	934

S182		126	1	OAR1_24633611.1	OAR1_27431644.1	2798.03	43
S182		829	1	s54037.1	s69885.1	10257.1	174
S182		606	1	OAR1_15578534.1	s69885.1	12559.2	218
S182		702	1	OAR1_7717464.1	OAR1_58938560.1	51221.1	934
S182		123	1	s67590.1	s45674.1	11170	204
S182		801	1	OAR1_25052307.1	s69885.1	3085.43	47
S182		40	1	OAR1_27215234.1	s14002.1	2024.45	36
S182	CON	7	1	OAR1_27215234.1	OAR1_27431644.1	216.41	5
S182	UNION	7	1	OAR1_7717464.1	OAR1_58938560.1	51221.1	934
S185		829	1	s54037.1	s69885.1	10257.1	174
S185		49	1	s38279.1	OAR1_19214328_X.1	1987.17	36
S185		606	1	OAR1_15578534.1	s69885.1	12559.2	218
S185		627	1	OAR1_15256499.1	OAR1_18052854.1	2796.36	53
S185		702	1	OAR1_7717464.1	OAR1_58938560.1	51221.1	934
S185		90	1	OAR1_16750809.1	OAR1_19152280.1	2401.47	43
S185		507	1	OAR1_16852861.1	s20207.1	1863.11	30
S185	CON	7	1	s54037.1	OAR1_18052854.1	172.176	3
S185	UNION	7	1	OAR1_7717464.1	OAR1_58938560.1	51221.1	934
S186		90	26	s17399.1	s68466.1	2193.9	38
S186		61	26	OAR26_36101444.1	OAR26_38739733.1	2638.29	38
S186		27	26	s70442.1	OAR26_38635621.1	2879.24	43
S186		629	26	OAR26_35704585.1	OAR26_38568519.1	2863.93	43
S186		45	26	s70488.1	OAR26_42514266.1	6953.15	122
S186		49	26	s55357.1	OAR26_38251830.1	2778.42	39
S186	CON	6	26	s17399.1	OAR26_38251830.1	321.706	4
S186	UNION	6	26	s55357.1	OAR26_42514266.1	7040.86	123
S187		844	26	OAR26_28236209.1	OAR26_30036643.1	1800.43	37
S187		29	26	OAR26_26341151.1	OAR26_31513764.1	5172.61	112
S187		27	26	OAR26_19029894.1	s08067.1	9710.75	157
S187		507	26	s09495.1	s30198.1	2950.89	60
S187		702	26	OAR26_28236209.1	OAR26_30762736.1	2526.53	55
S187		140	26	OAR26_28570026.1	OAR26_30797071.1	2227.05	49
S187	CON	6	26	OAR26_28570026.1	s08067.1	170.622	6
S187	UNION	6	26	OAR26_19029894.1	OAR26_31513764.1	12483.9	219
S193		126	25	s51480.1	OAR25_48288071_X.1	3036.69	51
S193		801	25	OAR25_44198124.1	s41617.1	1631.45	33
S193		27	25	OAR25_44198124.1	s37589.1	1568.76	31
S193		45	25	OAR25_43801573.1	s37589.1	1965.31	37
S193		29	25	OAR25_43581009.1	s51234.1	3907.33	73
S193		123	25	OAR25_43417329.1	OAR25_48288071_X.1	4870.74	91
S193	CON	6	25	s51480.1	s37589.1	515.497	6
S193	UNION	6	25	OAR25_43417329.1	OAR25_48288071_X.1	4870.74	91
S196		27	23	s58136.1	OAR23_60124720.1	9675.72	139
S196		29	23	s58136.1	OAR23_60124720.1	9675.72	139
S196		507	23	s47120.1	OAR23_56845264.1	5236.32	78
S196		844	23	OAR23_52077991.1	s45849.1	3992.95	58
S196		45	23	OAR23_55487746.1	OAR23_58270558.1	2782.81	41
S196		123	23	OAR23_55806264.1	OAR23_59401394.1	3595.13	53
S196	CON	6	23	OAR23_55806264.1	s45849.1	264.677	4
S196	UNION	6	23	s58136.1	OAR23_60124720.1	9675.72	139

S199		616	22	OAR22_5595255.1	OAR22_7399531.1	1804.28	38
S199		140	22	s26220.1	OAR22_5711915.1	5711.91	80
S199		45	22	OAR22_5711915.1	OAR22_8033581.1	2321.67	47
S199		829	22	OAR22_4104153.1	OAR22_6064565.1	1960.41	39
S199		507	22	s68573.1	OAR22_8623862.1	8623.86	128
S199		126	22	OAR22_1250324.1	OAR22_7588884.1	6338.56	102
S199	CON	6	22	OAR22_5711915.1	OAR22_5711915.1	0	1
S199	UNION	6	22	s26220.1	OAR22_8623862.1	8623.86	139
S200		702	21	OAR21_38087037.1	s49922.1	13700.7	160
S200		507	21	s26077.1	s59900.1	2557.94	53
S200		140	21	s75799.1	OAR21_38087037.1	2579.68	44
S200		27	21	s25809.1	s13923.1	11828.3	168
S200		90	21	s75804.1	s55303.1	6697.66	125
S200		829	21	OAR21_37641150.1	OAR21_39223598.1	1582.45	32
S200	CON	6	21	OAR21_38087037.1	OAR21_38087037.1	0	1
S200	UNION	6	21	s25809.1	s49922.1	19364.6	265
S201		45	21	OAR21_33336974.1	OAR21_36484190_X.1	3147.22	53
S201		556	21	s68739.1	OAR21_34906939.1	1760.9	38
S201		79	21	OAR21_33044180.1	s61235.1	3111.66	57
S201		606	21	OAR21_21865772.1	OAR21_36571389.1	14705.6	232
S201		27	21	s25809.1	s13923.1	11828.3	168
S201		90	21	s75804.1	s55303.1	6697.66	125
S201	CON	6	21	OAR21_33336974.1	OAR21_34906939.1	1569.96	33
S201	UNION	6	21	OAR21_21865772.1	s13923.1	22385.7	328
S202		844	21	OAR21_23862060.1	s47821.1	8308.09	126
S202		140	21	OAR21_21752249.1	OAR21_24084777.1	2332.53	39
S202		45	21	OAR21_19280450.1	OAR21_24102988.1	4822.54	78
S202		49	21	s74518.1	s38134.1	13941.9	212
S202		79	21	OAR21_16500504.1	OAR21_24084777.1	7584.27	125
S202		606	21	OAR21_21865772.1	OAR21_36571389.1	14705.6	232
S202	CON	6	21	OAR21_23862060.1	OAR21_24084777.1	222.717	7
S202	UNION	6	21	OAR21_16500504.1	OAR21_36571389.1	20070.9	319
S203		507	19	OAR19_14916596.1	OAR19_17059871.1	2143.28	42
S203		79	19	OAR19_14916596.1	OAR19_16594474.1	1677.88	33
S203		629	19	OAR19_13260250.1	OAR19_15065899.1	1805.65	32
S203		627	19	s40605.1	OAR19_15065899.1	2211.24	41
S203		61	19	OAR19_10268706.1	s25958.1	4848.61	90
S203		702	19	OAR19_7253713.1	s27711.1	16961.5	313
S203	CON	6	19	OAR19_14916596.1	OAR19_15065899.1	149.303	3
S203	UNION	6	19	OAR19_7253713.1	s27711.1	16961.5	313
S204		79	18	s19585.1	s20451.1	2188.81	41
S204		844	18	OAR18_66384520.1	s52137.1	1484.08	32
S204		29	18	s07565.1	s67955.1	1775.03	40
S204		507	18	OAR18_65660908.1	OAR18_68832375.1	3171.47	67
S204		627	18	s28124.1	s67734.1	2172.87	45
S204		90	18	s25861.1	s27043.1	5385.42	82
S204	CON	6	18	s19585.1	s52137.1	545.822	10
S204	UNION	6	18	OAR18_65660908.1	s27043.1	6819.35	115

S205		140	18	OAR18_51628425.1	OAR18_53728497.1	2100.07	32
S205		627	18	OAR18_51628425.1	OAR18_53590500.1	1962.08	30
S205		29	18	OAR18_51313531.1	OAR18_53728497.1	2414.97	40
S205		152	18	OAR18_51594268.1	OAR18_55121773.1	3527.51	53
S205		606	18	OAR18_52219091.1	s59424.1	5545.32	70
S205		507	18	OAR18_52361774.1	OAR18_56351698.1	3989.92	47
S205	CON	6	18	OAR18_52361774.1	OAR18_53590500.1	1228.73	14
S205	UNION	6	18	OAR18_51313531.1	s59424.1	6450.88	91
S206		829	17	s44509.1	s69675.1	5770.21	91
S206		152	17	s44509.1	s33211.1	2693.86	44
S206		507	17	s47176.1	s49020.1	26754.9	433
S206		702	17	OAR17_36966397.1	OAR17_74039265.1	37072.9	600
S206		79	17	OAR17_55061923.1	OAR17_62559974.1	7498.05	120
S206		627	17	OAR17_53440227.1	s65370.1	1678.53	30
S206	CON	6	17	OAR17_55061923.1	s65370.1	56.831	2
S206	UNION	6	17	OAR17_36966397.1	OAR17_74039265.1	37072.9	600
S207		844	16	OAR16_13101603.1	OAR16_16543821.1	3442.22	62
S207		79	16	s46646.1	OAR16_13101603.1	2266.33	44
S207		123	16	OAR16_12276858.1	OAR16_13935744.1	1658.89	36
S207		702	16	OAR16_11050320.1	OAR16_20062365.1	9012.05	173
S207		29	16	OAR16_11445590.1	OAR16_15329984.1	3884.39	78
S207		90	16	OAR16_12311919.1	s45825.1	1470.32	33
S207	CON	6	16	OAR16_13101603.1	OAR16_13101603.1	0	1
S207	UNION	6	16	s46646.1	OAR16_20062365.1	9227.09	175
S208		123	16	OAR16_12276858.1	OAR16_13935744.1	1658.89	36
S208		702	16	OAR16_11050320.1	OAR16_20062365.1	9012.05	173
S208		79	16	s46646.1	OAR16_13101603.1	2266.33	44
S208		616	16	OAR16_10423797.1	OAR16_12590773.1	2166.98	39
S208		29	16	OAR16_11445590.1	OAR16_15329984.1	3884.39	78
S208		90	16	OAR16_12311919.1	s45825.1	1470.32	33
S208	CON	6	16	OAR16_12311919.1	OAR16_12590773.1	278.854	5
S208	UNION	6	16	OAR16_10423797.1	OAR16_20062365.1	9638.57	184
S209		29	15	OAR15_73660126.1	OAR15_75673025.1	2012.9	35
S209		829	15	OAR15_73610660.1	OAR15_77297861.1	3687.2	75
S209		507	15	s65800.1	s24876.1	2625.56	43
S209		606	15	s60467.1	s57222.1	4219.52	71
S209		629	15	OAR15_70639412.1	OAR15_74238319.1	3598.91	64
S209		702	15	OAR15_71907029.1	OAR15_76144996.1	4237.97	71
S209	CON	6	15	OAR15_73660126.1	OAR15_74238319.1	578.193	12
S209	UNION	6	15	OAR15_70639412.1	OAR15_77297861.1	6658.45	126
S210		45	15	s54590.1	OAR15_72854094.1	2193.47	40
S210		507	15	s36214.1	OAR15_72854094.1	2761.84	49
S210		606	15	s60467.1	s57222.1	4219.52	71
S210		629	15	OAR15_70639412.1	OAR15_74238319.1	3598.91	64
S210		49	15	s33931.1	OAR15_72412908.1	2808.28	55
S210		702	15	OAR15_71907029.1	OAR15_76144996.1	4237.97	71
S210	CON	6	15	s60467.1	OAR15_72412908.1	45.23	2
S210	UNION	6	15	s33931.1	s57222.1	6982.57	124
S218		27	15	OAR15_50594253.1	s18566.1	9220.78	110

S218		616	15	OAR15_49569253.1	OAR15_56883596.1	7314.34	74
S218		629	15	s45046.1	OAR15_53192586.1	6195.51	50
S218		606	15	OAR15_46501057.1	s04139.1	4370.39	54
S218		40	15	OAR15_43565431.1	s34379.1	9826.51	115
S218		627	15	OAR15_41465316.1	OAR15_53192586.1	11727.3	150
S218	CON	6	15	OAR15_50594253.1	s04139.1	277.19	3
S218	UNION	6	15	OAR15_41465316.1	s18566.1	18349.7	254
S221		606	15	OAR15_46501057.1	s04139.1	4370.39	54
S221		844	15	DU426312_454.1	OAR15_49170452.1	2798.33	41
S221		140	15	s20575.1	OAR15_49170452.1	3200.37	47
S221		40	15	OAR15_43565431.1	s34379.1	9826.51	115
S221		629	15	s45046.1	OAR15_53192586.1	6195.51	50
S221		627	15	OAR15_41465316.1	OAR15_53192586.1	11727.3	150
S221	CON	6	15	s45046.1	OAR15_49170452.1	2173.37	31
S221	UNION	6	15	OAR15_41465316.1	s34379.1	11926.6	153
S223		606	15	OAR15_46501057.1	s04139.1	4370.39	54
S223		844	15	DU426312_454.1	OAR15_49170452.1	2798.33	41
S223		140	15	s20575.1	OAR15_49170452.1	3200.37	47
S223		40	15	OAR15_43565431.1	s34379.1	9826.51	115
S223		90	15	OAR15_41465316.1	OAR15_46781842.1	5316.53	97
S223		627	15	OAR15_41465316.1	OAR15_53192586.1	11727.3	150
S223	CON	6	15	OAR15_46501057.1	OAR15_46781842.1	280.785	4
S223	UNION	6	15	OAR15_41465316.1	s34379.1	11926.6	153
S224		702	14	s52270.1	s56709.1	2277.8	36
S224		90	14	OAR14_55447588.1	OAR14_58537282.1	3089.69	43
S224		140	14	OAR14_54070310.1	OAR14_56421333.1	2351.02	30
S224		49	14	s42994.1	s41160.1	2649.81	37
S224		126	14	s39024.1	s04653.1	4645.22	62
S224		152	14	OAR14_52929077.1	OAR14_56868551.1	3939.47	57
S224	CON	6	14	s52270.1	OAR14_56421333.1	64.195	2
S224	UNION	6	14	s39024.1	s56709.1	6483.76	92
S225		152	14	s39747.1	OAR14_27841684.1	3193.62	54
S225		123	14	s39747.1	OAR14_27342805.1	2694.74	42
S225		629	14	OAR14_24191061.1	OAR14_28394503.1	4203.44	73
S225		49	14	s48615.1	OAR14_33475732.1	10584	174
S225		702	14	s22016.1	s49153.1	8137.25	142
S225		606	14	s68592.1	OAR14_27152874.1	3078	49
S225	CON	6	14	s39747.1	s49153.1	1391.17	17
S225	UNION	6	14	s22016.1	OAR14_33475732.1	15573.7	265
S228		27	13	s57248.1	OAR13_87964841.1	3134.51	57
S228		844	13	OAR13_82596775_X.1	s66542.1	3352.33	60
S228		702	13	OAR13_82596775_X.1	s37902.1	2628.29	51
S228		140	13	OAR13_85176472.1	OAR13_89063022.1	3886.55	70
S228		45	13	OAR13_82895452.1	OAR13_85176472.1	2281.02	45
S228		507	13	OAR13_82659110.1	OAR13_89063022.1	6403.91	118
S228	CON	6	13	OAR13_85176472.1	OAR13_85176472.1	0	1
S228	UNION	6	13	OAR13_82596775_X.1	OAR13_89063022.1	6466.25	119
S229		90	13	s03928.1	s05093.1	1659.03	33
S229		507	13	OAR13_82659110.1	OAR13_89063022.1	6403.91	118

S229		844	13	OAR13_82596775_X.1	s66542.1	3352.33	60
S229		702	13	OAR13_82596775_X.1	s37902.1	2628.29	51
S229		616	13	OAR13_81897604.1	s03117.1	1965.48	36
S229		45	13	OAR13_82895452.1	OAR13_85176472.1	2281.02	45
S229	CON	6	13	s03928.1	s03117.1	740.826	14
S229	UNION	6	13	OAR13_81897604.1	OAR13_89063022.1	7165.42	132
S236		79	13	s74098.1	s07261.1	2464.24	39
S236		801	13	OAR13_40723732.1	OAR13_44858092.1	4134.36	68
S236		45	13	OAR13_39447377.1	s32471.1	2176.24	31
S236		49	13	OAR13_39447377.1	s32471.1	2176.24	31
S236		556	13	s18674.1	OAR13_44631782.1	5987.19	98
S236		29	13	OAR13_39146165.1	OAR13_41484865.1	2338.7	37
S236	CON	6	13	s74098.1	OAR13_41484865.1	280.539	5
S236	UNION	6	13	s18674.1	OAR13_44858092.1	6213.5	103
S238		90	13	OAR13_31557833.1	OAR13_35413855.1	3856.02	66
S238		152	13	OAR13_29150493.1	OAR13_33035124.1	3884.63	69
S238		27	13	s73266.1	s57045.1	4828.41	91
S238		29	13	s62936.1	OAR13_35198687.1	9473.28	175
S238		829	13	s29601.1	OAR13_37876562.1	14843.5	275
S238		123	13	s05740.1	OAR13_33983722.1	4640.74	88
S238	CON	6	13	OAR13_31557833.1	s57045.1	618.773	11
S238	UNION	6	13	s29601.1	OAR13_37876562.1	14843.5	275
S239		606	13	OAR13_22595627.1	OAR13_26635884.1	4040.26	80
S239		629	13	OAR13_22595627.1	OAR13_26635884.1	4040.26	80
S239		49	13	OAR13_21176498.1	OAR13_26177858.1	5001.36	93
S239		61	13	OAR13_20869936.1	OAR13_22642702.1	1772.77	33
S239		616	13	OAR13_19366874.1	s47163.1	7083.23	139
S239		123	13	s43905.1	OAR13_26635884.1	6436.53	127
S239	CON	6	13	OAR13_22595627.1	OAR13_22642702.1	47.075	2
S239	UNION	6	13	OAR13_19366874.1	OAR13_26635884.1	7269.01	144
S242		606	12	s49565.1	OAR12_63132677.1	1788.24	36
S242		90	12	OAR12_58680505.1	OAR12_61974786.1	3294.28	48
S242		627	12	s05958.1	OAR12_61618681.1	8919.27	136
S242		629	12	s05958.1	OAR12_61618681.1	8919.27	136
S242		123	12	OAR12_30882746.1	OAR12_62371899.1	31489.2	574
S242		702	12	s22960.1	s60710.1	58755.9	1088
S242	CON	6	12	s49565.1	OAR12_61618681.1	274.248	6
S242	UNION	6	12	s22960.1	s60710.1	58755.9	1088
S243		627	12	s05958.1	OAR12_61618681.1	8919.27	136
S243		629	12	s05958.1	OAR12_61618681.1	8919.27	136
S243		123	12	OAR12_30882746.1	OAR12_62371899.1	31489.2	574
S243		29	12	s38264.1	s74943.1	2477.01	48
S243		801	12	s52537.1	s15193.1	2447.59	48
S243		702	12	s22960.1	s60710.1	58755.9	1088
S243	CON	6	12	s38264.1	s15193.1	48.518	2
S243	UNION	6	12	s22960.1	s60710.1	58755.9	1088
S244		801	12	s52537.1	s15193.1	2447.59	48
S244		45	12	OAR12_49617704.1	OAR12_51498192.1	1880.49	37
S244		627	12	s63637.1	OAR12_51498192.1	2978.12	59

S244		629	12	s63637.1	OAR12_51498192.1	2978.12	59
S244		123	12	OAR12_30882746.1	OAR12_62371899.1	31489.2	574
S244		702	12	s22960.1	s60710.1	58755.9	1088
S244	CON	6	12	s52537.1	OAR12_51498192.1	564.148	10
S244	UNION	6	12	s22960.1	s60710.1	58755.9	1088
S245		126	12	s35634.1	s14364.1	1775.17	30
S245		123	12	OAR12_30882746.1	OAR12_62371899.1	31489.2	574
S245		702	12	s22960.1	s60710.1	58755.9	1088
S245		606	12	OAR12_43758965.1	OAR12_49228109.1	5469.14	102
S245		90	12	s71547.1	s44148.1	2398.95	43
S245		152	12	OAR12_46087352.1	s29250.1	2063.39	34
S245	CON	6	12	OAR12_46087352.1	s44148.1	906.443	14
S245	UNION	6	12	s22960.1	s60710.1	58755.9	1088
S246		629	12	OAR12_21022562.1	OAR12_22840561.1	1818	37
S246		140	12	OAR12_20168901.1	OAR12_22539169.1	2370.27	50
S246		27	12	OAR12_20168901.1	OAR12_21638552.1	1469.65	30
S246		616	12	OAR12_18138547.1	OAR12_21638552.1	3500.01	74
S246		507	12	s13792.1	s57817.1	31188.2	514
S246		702	12	s22960.1	s60710.1	58755.9	1088
S246	CON	6	12	OAR12_21022562.1	OAR12_21638552.1	615.99	13
S246	UNION	6	12	s13792.1	s60710.1	71900.8	1272
S249		140	12	OAR12_20168901.1	OAR12_22539169.1	2370.27	50
S249		27	12	OAR12_20168901.1	OAR12_21638552.1	1469.65	30
S249		616	12	OAR12_18138547.1	OAR12_21638552.1	3500.01	74
S249		45	12	OAR12_16839464.1	OAR12_20195850.1	3356.39	72
S249		507	12	s13792.1	s57817.1	31188.2	514
S249		702	12	s22960.1	s60710.1	58755.9	1088
S249	CON	6	12	OAR12_20168901.1	OAR12_20195850.1	26.949	2
S249	UNION	6	12	s13792.1	s60710.1	71900.8	1272
S252		79	11	s47275.1	OAR11_21925396.1	2007.05	32
S252		45	11	s52587.1	OAR11_21925396.1	2718.17	43
S252		829	11	s00452.1	s27347.1	1796.09	37
S252		140	11	s32976.1	OAR11_21925396.1	3582.11	65
S252		702	11	OAR11_17172323.1	OAR11_33359712.1	16187.4	260
S252		123	11	s17310.1	OAR11_29271153.1	9483.93	143
S252	CON	6	11	s47275.1	s27347.1	280.355	6
S252	UNION	6	11	OAR11_17172323.1	OAR11_33359712.1	16187.4	260
S253		123	11	s17310.1	OAR11_29271153.1	9483.93	143
S253		45	11	s52587.1	OAR11_21925396.1	2718.17	43
S253		140	11	s32976.1	OAR11_21925396.1	3582.11	65
S253		79	11	s32976.1	s33117.1	1526.31	32
S253		702	11	OAR11_17172323.1	OAR11_33359712.1	16187.4	260
S253		829	11	s00452.1	s27347.1	1796.09	37
S253	CON	6	11	s17310.1	s33117.1	82.375	4
S253	UNION	6	11	OAR11_17172323.1	OAR11_33359712.1	16187.4	260
S254		29	10	OAR10_59323880.1	OAR10_62073309_X.1	2749.43	37
S254		27	10	OAR10_58094557.1	OAR10_62988399.1	4893.84	90
S254		157	10	OAR10_57171204.1	OAR10_62988399.1	5817.19	110
S254		152	10	OAR10_56312119.1	OAR10_72914358.1	16602.2	277

S254		507	10	OAR10_54127756.1	s31288.1	7114.03	124
S254		90	10	s25185.1	OAR10_68005037.1	11797.8	188
S254	CON	6	10	OAR10_59323880.1	s31288.1	1917.9	22
S254	UNION	6	10	OAR10_54127756.1	OAR10_72914358.1	18786.6	320
S255		629	10	s45528.1	s10365.1	1833.37	34
S255		79	10	OAR10_19841785.1	OAR10_22164858.1	2323.07	43
S255		45	10	OAR10_17232091.1	s09543.1	3126.98	55
S255		123	10	OAR10_17232091.1	OAR10_20247297.1	3015.21	54
S255		702	10	s49567.1	OAR10_48462141.1	48462.1	914
S255		844	10	OAR10_17703888.1	s09622.1	13279.3	268
S255	CON	6	10	s45528.1	OAR10_20247297.1	360.637	8
S255	UNION	6	10	s49567.1	OAR10_48462141.1	48462.1	914
S256		49	9	OAR9_33777151.1	s71002.1	1328.61	32
S256		606	9	OAR9_32239874.1	DU427730_369.1	4216.13	94
S256		123	9	OAR9_32131604.1	OAR9_34495564.1	2363.96	53
S256		79	9	s73465.1	OAR9_40771150.1	8319.46	179
S256		45	9	OAR9_26972939.1	OAR9_35203437.1	8230.5	170
S256		61	9	OAR9_31664097.1	OAR9_36052313.1	4388.22	101
S256	CON	6	9	OAR9_33777151.1	OAR9_34495564.1	718.413	18
S256	UNION	6	9	OAR9_26972939.1	OAR9_40771150.1	13798.2	283
S259		27	9	s30783.1	OAR9_32605349.1	3103.89	69
S259		79	9	s73465.1	OAR9_40771150.1	8319.46	179
S259		45	9	OAR9_26972939.1	OAR9_35203437.1	8230.5	170
S259		606	9	OAR9_32239874.1	DU427730_369.1	4216.13	94
S259		123	9	OAR9_32131604.1	OAR9_34495564.1	2363.96	53
S259		61	9	OAR9_31664097.1	OAR9_36052313.1	4388.22	101
S259	CON	6	9	s73465.1	OAR9_32605349.1	153.655	4
S259	UNION	6	9	OAR9_26972939.1	OAR9_40771150.1	13798.2	283
S262		627	8	OAR8_37276015.1	OAR8_39847976.1	2571.96	50
S262		629	8	OAR8_37276015.1	OAR8_39847976.1	2571.96	50
S262		844	8	OAR8_35694056.1	OAR8_37524612.1	1830.56	40
S262		123	8	OAR8_34202419.1	OAR8_45617687.1	11415.3	218
S262		61	8	s42406.1	OAR8_37553450.1	5987.56	109
S262		702	8	OAR8_26793121.1	OAR8_38710483.1	11917.4	231
S262	CON	6	8	OAR8_37276015.1	OAR8_37524612.1	248.597	3
S262	UNION	6	8	OAR8_26793121.1	OAR8_45617687.1	18824.6	363
S263		616	7	s03655.1	OAR7_83970535.1	2138.66	41
S263		606	7	s20865.1	s35464.1	1561.95	33
S263		29	7	s20865.1	OAR7_83073383.1	1353.51	30
S263		844	7	s03357.1	s61284.1	4208.81	84
S263		61	7	OAR7_79189143.1	s15556.1	5289.18	104
S263		123	7	s13905.1	OAR7_91483736.1	9054.19	169
S263	CON	6	7	s13905.1	OAR7_83073383.1	643.839	12
S263	UNION	6	7	OAR7_79189143.1	OAR7_91483736.1	12294.6	237
S264		801	7	OAR7_57790006.1	OAR7_60473904.1	2683.9	54
S264		507	7	OAR7_52043940.1	OAR7_71832636.1	19788.7	355
S264		45	7	OAR7_31172405.1	OAR7_58958307.1	27785.9	542
S264		702	7	OAR7_31685912.1	OAR7_72973471.1	41287.6	769
S264		616	7	OAR7_54780217.1	s36119.1	5323.48	103

S264		29	7	OAR7_56240366.1	OAR7_77667178.1	21426.8	390
S264	CON	6	7	OAR7_57790006.1	OAR7_58958307.1	1168.3	26
S264	UNION	6	7	OAR7_31172405.1	OAR7_77667178.1	46494.8	878
S265		507	7	OAR7_52043940.1	OAR7_71832636.1	19788.7	355
S265		844	7	OAR7_50718877.1	OAR7_52124140.1	1405.26	30
S265		616	7	OAR7_50594184.1	OAR7_52043940.1	1449.76	31
S265		45	7	OAR7_31172405.1	OAR7_58958307.1	27785.9	542
S265		702	7	OAR7_31685912.1	OAR7_72973471.1	41287.6	769
S265		27	7	OAR7_51824628.1	s56915.1	1393.42	30
S265	CON	6	7	OAR7_52043940.1	OAR7_52043940.1	0	1
S265	UNION	6	7	OAR7_31172405.1	OAR7_72973471.1	41801.1	782
S266		801	7	s15119.1	OAR7_40985103.1	1282.67	32
S266		616	7	OAR7_39499497.1	OAR7_40869088.1	1369.59	35
S266		140	7	s56284.1	OAR7_39885967.1	2987.33	57
S266		45	7	OAR7_31172405.1	OAR7_58958307.1	27785.9	542
S266		702	7	OAR7_31685912.1	OAR7_72973471.1	41287.6	769
S266		61	7	OAR7_38702831.1	s18956.1	1507.44	35
S266	CON	6	7	s15119.1	OAR7_39885967.1	183.539	4
S266	UNION	6	7	OAR7_31172405.1	OAR7_72973471.1	41801.1	782
S267		61	7	OAR7_19080089.1	s58125.1	1926.97	36
S267		157	7	OAR7_18037906.1	s17110.1	1676.07	38
S267		507	7	OAR7_18037906.1	s17110.1	1676.07	38
S267		140	7	s04477.1	s17110.1	2008.95	47
S267		79	7	s04477.1	s17110.1	2008.95	47
S267		123	7	s13188.1	s71719.1	12317	181
S267	CON	6	7	OAR7_19080089.1	s17110.1	633.889	14
S267	UNION	6	7	s13188.1	s71719.1	12317	181
S271		801	6	OAR6_120674641.1	s42254.1	3373.02	61
S271		45	6	OAR6_119942135.1	OAR6_122276062.1	2333.93	39
S271		507	6	OAR6_119942135.1	OAR6_122276062.1	2333.93	39
S271		61	6	OAR6_119546066.1	s55094.1	2796.93	50
S271		606	6	OAR6_119125205.1	OAR6_121181023.1	2055.82	46
S271		140	6	s08027.1	s18701.1	4432.34	84
S271	CON	6	6	OAR6_120674641.1	OAR6_121181023.1	506.382	10
S271	UNION	6	6	OAR6_119125205.1	s42254.1	4922.45	97
S272		157	6	s63186.1	OAR6_46455936.1	3277.03	62
S272		140	6	s73122.1	OAR6_49463357.1	26607.7	513
S272		627	6	OAR6_42576838.1	OAR6_44769116.1	2192.28	42
S272		45	6	OAR6_38919831.1	OAR6_50590550.1	11670.7	219
S272		702	6	OAR6_6278702.1	OAR6_45273992.1	38995.3	741
S272		801	6	OAR6_42763868.1	s19086.1	1923.69	37
S272	CON	6	6	s63186.1	s19086.1	1508.66	28
S272	UNION	6	6	OAR6_6278702.1	OAR6_50590550.1	44311.8	859
S273		627	6	OAR6_42576838.1	OAR6_44769116.1	2192.28	42
S273		45	6	OAR6_38919831.1	OAR6_50590550.1	11670.7	219
S273		801	6	OAR6_42763868.1	s19086.1	1923.69	37
S273		79	6	OAR6_41044118.1	OAR6_43034224.1	1990.11	37
S273		702	6	OAR6_6278702.1	OAR6_45273992.1	38995.3	741
S273		140	6	s73122.1	OAR6_49463357.1	26607.7	513

S273	CON	6	6	OAR6_42763868.1	OAR6_43034224.1	270.356	7
S273	UNION	6	6	OAR6_6278702.1	OAR6_50590550.1	44311.8	859
S278		45	6	OAR6_38919831.1	OAR6_50590550.1	11670.7	219
S278		844	6	OAR6_38805979_X.1	OAR6_42208195.1	3402.22	45
S278		140	6	s73122.1	OAR6_49463357.1	26607.7	513
S278		79	6	OAR6_41044118.1	OAR6_43034224.1	1990.11	37
S278		829	6	OAR6_39029427.1	OAR6_41936490.1	2907.06	36
S278		702	6	OAR6_6278702.1	OAR6_45273992.1	38995.3	741
S278	CON	6	6	OAR6_41044118.1	OAR6_41936490.1	892.372	14
S278	UNION	6	6	OAR6_6278702.1	OAR6_50590550.1	44311.8	859
S283		844	6	OAR6_38805979_X.1	OAR6_42208195.1	3402.22	45
S283		140	6	s73122.1	OAR6_49463357.1	26607.7	513
S283		45	6	OAR6_38919831.1	OAR6_50590550.1	11670.7	219
S283		61	6	OAR6_36486409_X.1	s43499.1	3721.22	57
S283		702	6	OAR6_6278702.1	OAR6_45273992.1	38995.3	741
S283		829	6	OAR6_39029427.1	OAR6_41936490.1	2907.06	36
S283	CON	6	6	OAR6_39029427.1	s43499.1	1178.2	9
S283	UNION	6	6	OAR6_6278702.1	OAR6_50590550.1	44311.8	859
S284		829	6	OAR6_30406285.1	OAR6_33075221.1	2668.94	56
S284		123	6	s65803.1	OAR6_35548649.1	7909.9	153
S284		627	6	OAR6_27463172_X.1	OAR6_30478260.1	3015.09	53
S284		629	6	OAR6_27463172_X.1	OAR6_30478260.1	3015.09	53
S284		140	6	s73122.1	OAR6_49463357.1	26607.7	513
S284		702	6	OAR6_6278702.1	OAR6_45273992.1	38995.3	741
S284	CON	6	6	OAR6_30406285.1	OAR6_30478260.1	71.975	3
S284	UNION	6	6	OAR6_6278702.1	OAR6_49463357.1	43184.7	833
S286		702	6	OAR6_6278702.1	OAR6_45273992.1	38995.3	741
S286		152	6	OAR6_6254316.1	OAR6_10305227.1	4050.91	72
S286		79	6	s40472.1	s30534.1	2475.33	38
S286		507	6	OAR6_4458962.1	OAR6_6482986_X.1	2024.03	38
S286		90	6	OAR6_4458962.1	s44871.1	4179.72	70
S286		45	6	OAR6_6211452.1	OAR6_10161100.1	3949.65	69
S286	CON	6	6	OAR6_6278702.1	OAR6_6482986_X.1	204.285	5
S286	UNION	6	6	OAR6_4458962.1	OAR6_45273992.1	40815	774
S288		90	5	DU444709_372.1	OAR5_68350537.1	7925.86	122
S288		79	5	OAR5_57265711.1	s17800.1	9271.25	138
S288		152	5	s12940.1	OAR5_61924626.1	14110.5	253
S288		629	5	OAR5_57983508.1	OAR5_71824766.1	13841.3	230
S288		627	5	OAR5_57983508.1	OAR5_66325807.1	8342.3	120
S288		801	5	OAR5_59243774.1	s14903.1	2840.84	35
S288	CON	6	5	DU444709_372.1	OAR5_61924626.1	1499.95	10
S288	UNION	6	5	s12940.1	OAR5_71824766.1	24010.7	431
S292		801	5	OAR5_59243774.1	s14903.1	2840.84	35
S292		79	5	OAR5_57265711.1	s17800.1	9271.25	138
S292		829	5	s00980.1	OAR5_59243774.1	5486.89	100
S292		152	5	s12940.1	OAR5_61924626.1	14110.5	253
S292		629	5	OAR5_57983508.1	OAR5_71824766.1	13841.3	230
S292		627	5	OAR5_57983508.1	OAR5_66325807.1	8342.3	120
S292	CON	6	5	OAR5_59243774.1	OAR5_59243774.1	0	1

S292	UNION	6	5	s12940.1	OAR5_71824766.1	24010.7	431
S293		29	5	s54250.1	OAR5_58587196.1	3259.14	61
S293		829	5	s00980.1	OAR5_59243774.1	5486.89	100
S293		152	5	s12940.1	OAR5_61924626.1	14110.5	253
S293		801	5	OAR5_47774570.1	s54250.1	7553.49	151
S293		140	5	OAR5_52351898.1	OAR5_58830573.1	6478.68	118
S293		702	5	OAR5_53435489.1	OAR5_55651844.1	2216.36	43
S293	CON	6	5	s54250.1	s54250.1	0	1
S293	UNION	6	5	OAR5_47774570.1	OAR5_61924626.1	14150.1	254
S294		507	4	OAR4_76898220.1	OAR4_83911244.1	7013.02	135
S294		27	4	OAR4_76552142.1	OAR4_78216552.1	1664.41	33
S294		29	4	OAR4_74705318.1	OAR4_78216552.1	3511.23	72
S294		702	4	s58367.1	OAR4_85483475.1	25992.5	514
S294		616	4	OAR4_72367529.1	OAR4_78991289.1	6623.76	132
S294		627	4	s56367.1	OAR4_80229268.1	3866.18	75
S294	CON	6	4	OAR4_76898220.1	OAR4_78216552.1	1318.33	25
S294	UNION	6	4	s58367.1	OAR4_85483475.1	25992.5	514
S295		152	4	OAR4_60126261.1	OAR4_62657058.1	2530.8	55
S295		45	4	OAR4_55744716.1	OAR4_60926085.1	5181.37	106
S295		627	4	OAR4_55669092.1	OAR4_60497897.1	4828.81	98
S295		27	4	DU194821_342.1	OAR4_61284730.1	14228.1	279
S295		29	4	DU194821_342.1	OAR4_61284730.1	14228.1	279
S295		702	4	s58367.1	OAR4_85483475.1	25992.5	514
S295	CON	6	4	OAR4_60126261.1	OAR4_60497897.1	371.636	9
S295	UNION	6	4	DU194821_342.1	OAR4_85483475.1	38426.9	752
S297		45	4	OAR4_55744716.1	OAR4_60926085.1	5181.37	106
S297		606	4	OAR4_37940876.1	OAR4_55963926.1	18023	344
S297		27	4	DU194821_342.1	OAR4_61284730.1	14228.1	279
S297		29	4	DU194821_342.1	OAR4_61284730.1	14228.1	279
S297		61	4	OAR4_39813653.1	OAR4_56340637.1	16527	308
S297		627	4	OAR4_55669092.1	OAR4_60497897.1	4828.81	98
S297	CON	6	4	OAR4_55744716.1	OAR4_55963926.1	219.21	7
S297	UNION	6	4	OAR4_37940876.1	OAR4_61284730.1	23343.9	451
S298		629	4	OAR4_46599830.1	s05903.1	2265.52	38
S298		45	4	OAR4_43198108.1	s67196.1	8975.51	158
S298		61	4	OAR4_39813653.1	OAR4_56340637.1	16527	308
S298		27	4	DU194821_342.1	OAR4_61284730.1	14228.1	279
S298		29	4	DU194821_342.1	OAR4_61284730.1	14228.1	279
S298		606	4	OAR4_37940876.1	OAR4_55963926.1	18023	344
S298	CON	6	4	DU194821_342.1	s05903.1	1808.75	32
S298	UNION	6	4	OAR4_37940876.1	OAR4_61284730.1	23343.9	451
S299		829	4	OAR4_3610903.1	OAR4_5767890.1	2156.99	39
S299		79	4	OAR4_3610903.1	s41814.1	1891.47	36
S299		702	4	OARUn.284_293028.1	OAR4_24251309.1	24251.3	470
S299		29	4	OAR4_742452.1	OAR4_5847332.1	5104.88	85
S299		45	4	OAR4_2368769.1	s62909.1	2560.16	42
S299		61	4	OAR4_3630456.1	s45567.1	2316.36	42
S299	CON	6	4	OAR4_3630456.1	s62909.1	1298.47	23
S299	UNION	6	4	OARUn.284_293028.1	OAR4_24251309.1	24251.3	470

S300		61	3	OAR3_216958700.1	OAR3_218311285.1	1352.59	31
S300		627	3	OAR3_216714207.1	s32572.1	1813.47	38
S300		629	3	OAR3_216714207.1	s32572.1	1813.47	38
S300		606	3	s07450.1	OAR3_218311285.1	2546.7	53
S300		616	3	OAR3_209072421.1	OAR3_222052522.1	12980.1	230
S300		79	3	OAR3_217297404.1	OAR3_218733455.1	1436.05	32
S300	CON	6	3	OAR3_217297404.1	OAR3_218311285.1	1013.88	23
S300	UNION	6	3	OAR3_209072421.1	OAR3_222052522.1	12980.1	230
S301		627	3	OAR3_206227595.1	OAR3_209042148.1	2814.55	62
S301		629	3	OAR3_206227595.1	OAR3_209042148.1	2814.55	62
S301		829	3	OAR3_205737877.1	OAR3_208666709.1	2928.83	65
S301		152	3	OAR3_205307357.1	OAR3_207087501_X.1	1780.14	40
S301		507	3	OAR3_179244393.1	OAR3_211362922_X.1	32118.5	604
S301		157	3	OAR3_206856083.1	OAR3_209162669.1	2306.59	49
S301	CON	6	3	OAR3_206856083.1	OAR3_207087501_X.1	231.419	7
S301	UNION	6	3	OAR3_179244393.1	OAR3_211362922_X.1	32118.5	604
S302		616	3	OAR3_185468392.1	s17858.1	1913.95	35
S302		507	3	OAR3_179244393.1	OAR3_211362922_X.1	32118.5	604
S302		123	3	s55427.1	s32545.1	2793.06	53
S302		45	3	OAR3_183605277.1	s52959.1	3069.59	58
S302		702	3	OAR3_178121896.1	s16918.1	11927	230
S302		844	3	OAR3_182939964.1	OAR3_186988175.1	4048.21	73
S302	CON	6	3	s55427.1	s52959.1	760.127	11
S302	UNION	6	3	OAR3_178121896.1	OAR3_211362922_X.1	33241	624
S307		507	3	OAR3_179244393.1	OAR3_211362922_X.1	32118.5	604
S307		29	3	OAR3_179244393.1	s26854.1	4664.94	91
S307		616	3	OAR3_179244393.1	OAR3_181236274.1	1991.88	45
S307		606	3	OAR3_177909045.1	s26854.1	6000.29	115
S307		702	3	OAR3_178121896.1	s16918.1	11927	230
S307		61	3	s38401.1	OAR3_183605277.1	2795.96	51
S307	CON	6	3	s38401.1	OAR3_181236274.1	426.953	11
S307	UNION	6	3	OAR3_177909045.1	OAR3_211362922_X.1	33453.9	628
S310		627	3	OAR3_155236610.1	s75549.1	19415.4	375
S310		629	3	OAR3_155236610.1	s75549.1	19415.4	375
S310		126	3	OAR3_152741614.1	OAR3_163942175.1	11200.6	212
S310		61	3	s26286.1	s51772.1	2640.78	48
S310		29	3	s27935.1	DU358217_394.1	2232.57	35
S310		152	3	OAR3_158876220.1	OAR3_162346644.1	3470.42	61
S310	CON	6	3	s26286.1	DU358217_394.1	1263.16	24
S310	UNION	6	3	OAR3_152741614.1	s75549.1	21910.4	419
S311		606	3	OAR3_119895458.1	OAR3_130397300.1	10501.8	199
S311		45	3	OAR3_119895458.1	OAR3_121938058.1	2042.6	42
S311		616	3	OAR3_119168695.1	OAR3_121189388.1	2020.69	44
S311		140	3	OAR3_118738718.1	OAR3_121938058.1	3199.34	65
S311		702	3	OAR3_120965794.1	OAR3_123473821.1	2508.03	48
S311		629	3	OAR3_112096089.1	OAR3_121762751.1	9666.66	172
S311	CON	6	3	OAR3_120965794.1	OAR3_121189388.1	223.594	4
S311	UNION	6	3	OAR3_112096089.1	OAR3_130397300.1	18301.2	332

S314		606	3	OAR3_119895458.1	OAR3_130397300.1	10501.8	199
S314		45	3	OAR3_119895458.1	OAR3_121938058.1	2042.6	42
S314		616	3	OAR3_119168695.1	OAR3_121189388.1	2020.69	44
S314		140	3	OAR3_118738718.1	OAR3_121938058.1	3199.34	65
S314		123	3	OAR3_118383093.1	OAR3_120348297.1	1965.2	39
S314		629	3	OAR3_112096089.1	OAR3_121762751.1	9666.66	172
S314	CON	6	3	OAR3_119895458.1	OAR3_120348297.1	452.839	10
S314	UNION	6	3	OAR3_112096089.1	OAR3_130397300.1	18301.2	332
S315		152	3	OAR3_78511530.1	OAR3_80763380.1	2251.85	51
S315		507	3	OAR3_77718162.1	OAR3_82856188.1	5138.03	112
S315		606	3	OAR3_77361732.1	OAR3_78678231.1	1316.5	34
S315		49	3	s53138.1	OAR3_78678231.1	1353.04	35
S315		702	3	OAR3_78338656.1	s45788.1	2098.32	48
S315		123	3	OAR3_78338656.1	s45788.1	2098.32	48
S315	CON	6	3	OAR3_78511530.1	OAR3_78678231.1	166.701	5
S315	UNION	6	3	s53138.1	OAR3_82856188.1	5531	124
S318		27	3	OAR3_64474656.1	OAR3_67137932.1	2663.28	53
S318		40	3	OAR3_64474656.1	OAR3_66798124.1	2323.47	45
S318		627	3	OAR3_64474656.1	OAR3_66317995.1	1843.34	37
S318		45	3	s45049.1	OAR3_66317995.1	4167.71	82
S318		123	3	s24884.1	OAR3_67751047.1	1949.75	42
S318		61	3	s40974.1	OAR3_68372978.1	2366.7	50
S318	CON	6	3	s40974.1	OAR3_66317995.1	311.717	7
S318	UNION	6	3	s45049.1	OAR3_68372978.1	6222.69	125
S319		507	3	OAR3_43871305.1	OAR3_52722619.1	8851.31	160
S319		49	3	OAR3_43700480.1	OAR3_50145205.1	6444.73	118
S319		123	3	s72091.1	s14476.1	1854.07	41
S319		140	3	OAR3_42310885.1	OAR3_43889039.1	1578.15	34
S319		702	3	DU259120_464.1	OAR3_59494261.1	59494.3	1111
S319		27	3	s12024.1	s69330.1	4167.98	82
S319	CON	6	3	OAR3_43871305.1	OAR3_43889039.1	17.734	2
S319	UNION	6	3	DU259120_464.1	OAR3_59494261.1	59494.3	1111
S320		140	3	OAR3_42310885.1	OAR3_43889039.1	1578.15	34
S320		49	3	OAR3_43700480.1	OAR3_50145205.1	6444.73	118
S320		123	3	s72091.1	s14476.1	1854.07	41
S320		556	3	OAR3_42529367.1	OAR3_43817056.1	1287.69	30
S320		702	3	DU259120_464.1	OAR3_59494261.1	59494.3	1111
S320		27	3	s12024.1	s69330.1	4167.98	82
S320	CON	6	3	OAR3_43700480.1	OAR3_43817056.1	116.576	5
S320	UNION	6	3	DU259120_464.1	OAR3_59494261.1	59494.3	1111
S324		40	2	s50777.1	s71655.1	2404.82	56
S324		152	2	OAR2_245114849.1	s06384.1	1732.11	37
S324		29	2	OAR2_238444943.1	s43939.1	16224.6	314
S324		629	2	OAR2_242229131.1	DU503161_123.1	7148.91	146
S324		45	2	OAR2_246045571.1	OAR2_248329507.1	2283.94	47
S324		90	2	s50777.1	s27851.1	1738.78	39
S324	CON	6	2	s50777.1	s06384.1	178.181	6
S324	UNION	6	2	OAR2_238444943.1	s43939.1	16224.6	314
S325		45	2	OAR2_246045571.1	OAR2_248329507.1	2283.94	47

S325		507	2	OAR2_244460688.1	OAR2_246045571.1	1584.88	33
S325		629	2	OAR2_242229131.1	DU503161_123.1	7148.91	146
S325		627	2	OAR2_238165550.1	OAR2_246045571.1	7880.02	156
S325		29	2	OAR2_238444943.1	s43939.1	16224.6	314
S325		152	2	OAR2_245114849.1	s06384.1	1732.11	37
S325	CON	6	2	OAR2_246045571.1	OAR2_246045571.1	0	1
S325	UNION	6	2	OAR2_238165550.1	s43939.1	16504	318
S328		507	2	OAR2_244460688.1	OAR2_246045571.1	1584.88	33
S328		629	2	OAR2_242229131.1	DU503161_123.1	7148.91	146
S328		627	2	OAR2_238165550.1	OAR2_246045571.1	7880.02	156
S328		29	2	OAR2_238444943.1	s43939.1	16224.6	314
S328		844	2	OAR2_243206127.1	OAR2_245289710.1	2083.58	41
S328		152	2	OAR2_245114849.1	s06384.1	1732.11	37
S328	CON	6	2	OAR2_245114849.1	OAR2_245289710.1	174.861	5
S328	UNION	6	2	OAR2_238165550.1	s43939.1	16504	318
S329		79	2	OAR2_181847398.1	s12968.1	13971.1	260
S329		45	2	OAR2_180239034.1	s02554.1	3237.53	65
S329		702	2	OAR2_178886050.1	s29646.1	4550.66	94
S329		152	2	s61174.1	s26570.1	7267.15	138
S329		90	2	OAR2_181512709.1	OAR2_190616592.1	9103.88	175
S329		123	2	OAR2_181847398.1	OAR2_183941873.1	2094.47	47
S329	CON	6	2	OAR2_181847398.1	s29646.1	1589.32	35
S329	UNION	6	2	OAR2_178886050.1	s12968.1	16932.4	319
S331		126	2	OAR2_144244441.1	OAR2_149604966.1	5360.52	105
S331		61	2	OAR2_144244441.1	OAR2_148238035.1	3993.59	78
S331		507	2	OAR2_144244441.1	OAR2_146111824.1	1867.38	37
S331		627	2	OAR2_142684241.1	OAR2_148048660.1	5364.42	105
S331		123	2	OAR2_140051989_X.1	OAR2_144244441.1	4192.45	95
S331		702	2	s54108.1	OAR2_162167662.1	23623.2	482
S331	CON	6	2	OAR2_144244441.1	OAR2_144244441.1	0	1
S331	UNION	6	2	s54108.1	OAR2_162167662.1	23623.2	482
S332		627	2	OAR2_142684241.1	OAR2_148048660.1	5364.42	105
S332		829	2	OAR2_141723000.1	DU292901_688.1	2328.84	49
S332		123	2	OAR2_140051989_X.1	OAR2_144244441.1	4192.45	95
S332		40	2	OAR2_139988851.1	OAR2_143429707_X.1	3440.86	78
S332		702	2	s54108.1	OAR2_162167662.1	23623.2	482
S332		45	2	OAR2_141764296.1	OAR2_143949087.1	2184.79	45
S332	CON	6	2	OAR2_142684241.1	OAR2_143429707_X.1	745.467	13
S332	UNION	6	2	s54108.1	OAR2_162167662.1	23623.2	482
S333		507	1	s22571.1	s56772.1	15398.3	291
S333		90	1	OAR1_280918505.1	s59682.1	4159.05	86
S333		606	1	s35781.1	OAR1_287434870.1	2608.39	48
S333		61	1	s35781.1	OAR1_287434870.1	2608.39	48
S333		29	1	OAR1_283569518.1	s39199.1	5956.3	95
S333		702	1	OAR1_282017475.1	s67262.1	9551.48	171
S333	CON	6	1	s35781.1	s59682.1	251.074	4
S333	UNION	6	1	OAR1_280918505.1	s56772.1	15787.5	299
S334		61	1	OAR1_225094822.1	OAR1_233116174.1	8021.35	151
S334		829	1	OAR1_219136030.1	OAR1_236103073.1	16967	322

S334		629	1	OAR1_214417389.1	OAR1_233116174.1	18698.8	341
S334		702	1	OAR1_228131256.1	OAR1_233756581.1	5625.32	96
S334		40	1	OAR1_230374318.1	OAR1_234437646.1	4063.33	74
S334		152	1	OAR1_230994116.1	OAR1_233249488.1	2255.37	43
S334	CON	6	1	OAR1_230994116.1	OAR1_233116174.1	2122.06	41
S334	UNION	6	1	OAR1_214417389.1	OAR1_236103073.1	21685.7	402

S335		61	1	OAR1_225094822.1	OAR1_233116174.1	8021.35	151
S335		829	1	OAR1_219136030.1	OAR1_236103073.1	16967	322
S335		629	1	OAR1_214417389.1	OAR1_233116174.1	18698.8	341
S335		40	1	OAR1_230374318.1	OAR1_234437646.1	4063.33	74
S335		90	1	OAR1_222537672.1	OAR1_230460155.1	7922.48	157
S335		702	1	OAR1_228131256.1	OAR1_233756581.1	5625.32	96
S335	CON	6	1	OAR1_230374318.1	OAR1_230460155.1	85.837	3
S335	UNION	6	1	OAR1_214417389.1	OAR1_236103073.1	21685.7	402

S336		61	1	OAR1_225094822.1	OAR1_233116174.1	8021.35	151
S336		123	1	OAR1_223721671.1	OAR1_229347642.1	5625.97	108
S336		90	1	OAR1_222537672.1	OAR1_230460155.1	7922.48	157
S336		829	1	OAR1_219136030.1	OAR1_236103073.1	16967	322
S336		629	1	OAR1_214417389.1	OAR1_233116174.1	18698.8	341
S336		702	1	OAR1_228131256.1	OAR1_233756581.1	5625.32	96
S336	CON	6	1	OAR1_228131256.1	OAR1_229347642.1	1216.39	18
S336	UNION	6	1	OAR1_214417389.1	OAR1_236103073.1	21685.7	402

S338		61	1	OAR1_225094822.1	OAR1_233116174.1	8021.35	151
S338		123	1	OAR1_223721671.1	OAR1_229347642.1	5625.97	108
S338		45	1	OAR1_223622781.1	OAR1_225595665.1	1972.88	39
S338		90	1	OAR1_222537672.1	OAR1_230460155.1	7922.48	157
S338		829	1	OAR1_219136030.1	OAR1_236103073.1	16967	322
S338		629	1	OAR1_214417389.1	OAR1_233116174.1	18698.8	341
S338	CON	6	1	OAR1_225094822.1	OAR1_225595665.1	500.843	12
S338	UNION	6	1	OAR1_214417389.1	OAR1_236103073.1	21685.7	402

S339		123	1	OAR1_223721671.1	OAR1_229347642.1	5625.97	108
S339		45	1	OAR1_223622781.1	OAR1_225595665.1	1972.88	39
S339		90	1	OAR1_222537672.1	OAR1_230460155.1	7922.48	157
S339		829	1	OAR1_219136030.1	OAR1_236103073.1	16967	322
S339		629	1	OAR1_214417389.1	OAR1_233116174.1	18698.8	341
S339		702	1	OAR1_201628760.1	OAR1_224818104.1	23189.3	424
S339	CON	6	1	OAR1_223721671.1	OAR1_224818104.1	1096.43	21
S339	UNION	6	1	OAR1_201628760.1	OAR1_236103073.1	34474.3	639

S342		507	1	s71677.1	OAR1_206274474.1	4131.31	65
S342		629	1	DU316524_436.1	OAR1_207345817.1	5355.54	90
S342		829	1	s66109.1	s65811.1	2617.61	35
S342		801	1	OAR1_198827329.1	s56333.1	6897.16	121
S342		157	1	DU287626_225.1 250	506CS3900283200001_442.1	2218.24	32
S342		702	1	OAR1_201628760.1	OAR1_224818104.1	23189.3	424
S342	CON	6	1	s71677.1 250	506CS3900283200001_442.1	1146.48	11
S342	UNION	6	1	OAR1_198827329.1	OAR1_224818104.1	25990.8	478

S343		801	1	OAR1_173556038.1	s55456.1	1880.2	38
S343		627	1	OAR1_173251355.1	s30482.1	2379.26	46
S343		629	1	OAR1_173251355.1	s30482.1	2379.26	46

S343		79	1	OAR1_172310048.1	OAR1_174084771.1	1774.72	35
S343		123	1	OAR1_172310048.1	OAR1_174084771.1	1774.72	35
S343		27	1	OAR1_172859107.1	OAR1_177098302.1	4239.19	71
S343	CON	6	1	OAR1_173556038.1	OAR1_174084771.1	528.733	12
S343	UNION	6	1	OAR1_172310048.1	OAR1_177098302.1	4788.25	83
S344		40	1	OAR1_170034165.1	OAR1_171775698.1	1741.53	34
S344		606	1	OAR1_169720464.1	OAR1_171775698.1	2055.23	38
S344		45	1	OAR1_169643592.1	OAR1_171775698.1	2132.11	40
S344		27	1	OAR1_169609478.1	OAR1_171775698.1	2166.22	41
S344		627	1	OAR1_169609478.1	OAR1_171275473.1	1665.99	32
S344		629	1	OAR1_169609478.1	OAR1_171275473.1	1665.99	32
S344	CON	6	1	OAR1_170034165.1	OAR1_171275473.1	1241.31	25
S344	UNION	6	1	OAR1_169609478.1	OAR1_171775698.1	2166.22	41
S346		79	1	OAR1_149565545.1	OAR1_151312088.1	1746.54	42
S346		61	1	OAR1_148262943.1	OAR1_151312088.1	3049.14	67
S346		123	1	s53597.1	OAR1_153662211.1	35798.6	631
S346		27	1	OAR1_151206081.1	DU499024_430.1	2767.31	53
S346		40	1	OAR1_150005378.1	OAR1_151330117.1	1324.74	32
S346		90	1	OAR1_148901159.1	OAR1_151247138.1	2345.98	54
S346	CON	6	1	OAR1_151206081.1	OAR1_151247138.1	41.057	2
S346	UNION	6	1	s53597.1	DU499024_430.1	36109.8	636
S350		61	1	OAR1_117978374.1	OAR1_122656397.1	4678.02	78
S350		801	1	OAR1_110989185.1	s10939.1	7522.21	114
S350		829	1	OAR1_117951301.1	OAR1_120197222.1	2245.92	34
S350		702	1	s14189.1	s40751.1	42088.8	712
S350		45	1	s37607.1	s04671.1	16346.9	247
S350		123	1	s53597.1	OAR1_153662211.1	35798.6	631
S350	CON	6	1	OAR1_117978374.1	s10939.1	533.02	10
S350	UNION	6	1	s14189.1	OAR1_153662211.1	45938.1	791
S351		40	1	s69088.1	OAR1_114069491_X.1	2477.52	40
S351		801	1	OAR1_110989185.1	s10939.1	7522.21	114
S351		507	1	OAR1_109879519.1	OAR1_114069491_X.1	4189.97	75
S351		79	1	OAR1_108764473.1	s01025.1	4913.19	93
S351		27	1	s00446.1	s04333.1	4130.65	80
S351		702	1	s14189.1	s40751.1	42088.8	712
S351	CON	6	1	s69088.1	s04333.1	1002.09	20
S351	UNION	6	1	s14189.1	s40751.1	42088.8	712
S359		507	1	OAR1_77772595.1	OAR1_80338579.1	2565.98	53
S359		40	1	OAR1_77541835.1	OAR1_79381781.1	1839.95	37
S359		49	1	OAR1_77478775.1	OAR1_79381781.1	1903.01	38
S359		29	1	s09749.1	OAR1_77993538.1	1692.39	35
S359		45	1	OAR1_76111336.1	OAR1_77993538.1	1882.2	39
S359		61	1	s58781.1	s29884.1	9573.23	170
S359	CON	6	1	OAR1_77772595.1	s29884.1	91.737	3
S359	UNION	6	1	s58781.1	OAR1_80338579.1	12047.5	220
S360		507	1	OAR1_67421568.1	OAR1_71254279.1	3832.71	57
S360		123	1	OAR1_67236647.1	OAR1_69224190.1	1987.54	39
S360		606	1	OAR1_66298910.1	OAR1_68758775.1	2459.86	47
S360		90	1	OAR1_66156513.1	OAR1_74623022.1	8466.51	147

S360		702	1	OAR1_65265541_X.1	OAR1_73281352.1	8015.81	136
S360		61	1	s58781.1	s29884.1	9573.23	170
S360	CON	6	1	s58781.1	OAR1_68758775.1	467.676	8
S360	UNION	6	1	OAR1_65265541_X.1	s29884.1	12598.8	227
S361		507	1	OAR1_67421568.1	OAR1_71254279.1	3832.71	57
S361		123	1	OAR1_67236647.1	OAR1_69224190.1	1987.54	39
S361		606	1	OAR1_66298910.1	OAR1_68758775.1	2459.86	47
S361		90	1	OAR1_66156513.1	OAR1_74623022.1	8466.51	147
S361		702	1	OAR1_65265541_X.1	OAR1_73281352.1	8015.81	136
S361		29	1	OAR1_66062371.1	OAR1_68115107.1	2052.74	43
S361	CON	6	1	OAR1_67421568.1	OAR1_68115107.1	693.539	17
S361	UNION	6	1	OAR1_65265541_X.1	OAR1_74623022.1	9357.48	161
S362		61	26	s63463.1	OAR26_46995252.1	7812.86	140
S362		801	26	OAR26_38693145_X.1	s45791.1	2078.09	36
S362		45	26	s70488.1	OAR26_42514266.1	6953.15	122
S362		90	26	s17399.1	s68466.1	2193.9	38
S362		627	26	OAR26_38739733.1	s24097.1	2610.51	48
S362	CON	5	26	s63463.1	s68466.1	941.629	17
S362	UNION	5	26	s70488.1	OAR26_46995252.1	11434.1	195
S363		627	26	OAR26_38739733.1	s24097.1	2610.51	48
S363		801	26	OAR26_38693145_X.1	s45791.1	2078.09	36
S363		90	26	s17399.1	s68466.1	2193.9	38
S363		61	26	OAR26_36101444.1	OAR26_38739733.1	2638.29	38
S363		45	26	s70488.1	OAR26_42514266.1	6953.15	122
S363	CON	5	26	OAR26_38739733.1	OAR26_38739733.1	0	1
S363	UNION	5	26	s70488.1	OAR26_42514266.1	6953.15	122
S364		45	26	s70488.1	OAR26_42514266.1	6953.15	122
S364		49	26	s55357.1	OAR26_38251830.1	2778.42	39
S364		27	26	s70442.1	OAR26_38635621.1	2879.24	43
S364		629	26	OAR26_35704585.1	OAR26_38568519.1	2863.93	43
S364		40	26	s62504.1	s75723.1	1638.78	30
S364	CON	5	26	s70442.1	s75723.1	128.391	4
S364	UNION	5	26	s62504.1	OAR26_42514266.1	8268.27	145
S365		79	25	s25708.1	s19885.1	1565.13	31
S365		61	25	OAR25_12492866.1	OAR25_21044148.1	8551.28	175
S365		507	25	s10320.1	s12031.1	20755.8	401
S365		844	25	OAR25_19516269.1	OAR25_25861480.1	6345.21	122
S365		126	25	s72138.1	s47975.1	1901.42	35
S365	CON	5	25	s72138.1	s19885.1	369.773	9
S365	UNION	5	25	s10320.1	s12031.1	20755.8	401
S366		61	25	OAR25_12492866.1	OAR25_21044148.1	8551.28	175
S366		79	25	OAR25_12366791.1	OAR25_14279951.1	1913.16	35
S366		152	25	OAR25_10480244.1	OAR25_14279951.1	3799.71	71
S366		90	25	OAR25_10160789.1	s21640.1	3276.28	60
S366		507	25	s10320.1	s12031.1	20755.8	401
S366	CON	5	25	OAR25_12492866.1	s21640.1	944.203	16
S366	UNION	5	25	s10320.1	s12031.1	20755.8	401
S367		152	24	OAR24_11445879.1	OAR24_13675640.1	2229.76	30

S367		123	24	OAR24_10812839.1	s50198.1	5516.68	74
S367		629	24	s49862.1	OAR24_13169307.1	3385.14	46
S367		507	24	OAR24_8989305.1	s61066.1	4513.75	65
S367		702	24	s35472.1	OAR24_22201078.1	16855.5	259
S367	CON	5	24	OAR24_11445879.1	OAR24_13169307.1	1723.43	23
S367	UNION	5	24	s35472.1	OAR24_22201078.1	16855.5	259
S368		27	23	s58136.1	OAR23_60124720.1	9675.72	139
S368		29	23	s58136.1	OAR23_60124720.1	9675.72	139
S368		507	23	s47120.1	OAR23_56845264.1	5236.32	78
S368		844	23	OAR23_52077991.1	s45849.1	3992.95	58
S368		606	23	OAR23_52091614.1	OAR23_54737060.1	2645.45	44
S368	CON	5	23	OAR23_52091614.1	OAR23_54737060.1	2645.45	44
S368	UNION	5	23	s58136.1	OAR23_60124720.1	9675.72	139
S375		152	23	OAR23_46850769.1	OAR23_48878158.1	2027.39	34
S375		45	23	s37648.1	OAR23_48086126.1	4188.47	55
S375		629	23	s37648.1	OAR23_48086126.1	4188.47	55
S375		606	23	OAR23_47138977.1	s72429.1	3767.44	67
S375		844	23	s65540.1	OAR23_47913202.1	2599.54	32
S375	CON	5	23	OAR23_47138977.1	OAR23_47913202.1	774.225	13
S375	UNION	5	23	s37648.1	s72429.1	7008.76	105
S378		627	23	s19815.1	OAR23_41224486.1	2235.44	36
S378		629	23	s19815.1	OAR23_41224486.1	2235.44	36
S378		45	23	OAR23_32717464.1	s03900.1	8953.01	139
S378		702	23	OAR23_32970524.1	s47536.1	10557	158
S378		123	23	s68212.1	OAR23_40483343.1	2784.39	44
S378	CON	5	23	s19815.1	OAR23_40483343.1	1494.3	26
S378	UNION	5	23	OAR23_32717464.1	s47536.1	10810.1	163
S381		140	23	OAR23_30906493.1	OAR23_32970524.1	2064.03	32
S381		27	23	OAR23_28704954.1	OAR23_31443579.1	2738.62	41
S381		29	23	s58159.1	OAR23_31443579.1	5396.2	77
S381		79	23	OAR23_25154247.1	OAR23_31443579.1	6289.33	95
S381		616	23	OAR23_30686953.1	OAR23_32932392_X.1	2245.44	33
S381	CON	5	23	OAR23_30906493.1	OAR23_31443579.1	537.086	4
S381	UNION	5	23	OAR23_25154247.1	OAR23_32970524.1	7816.28	123
S382		79	23	s35508.1	OAR23_11959774.1	4010.75	66
S382		140	23	s62451.1	s50084.1	2433.33	42
S382		27	23	s01824.1	OAR23_8935783.1	2220.2	35
S382		702	23	s07716.1	OAR23_12771349.1	4783.72	80
S382		606	23	s33116.1	OAR23_10243385_X.1	2196.39	38
S382	CON	5	23	s33116.1	OAR23_8935783.1	888.784	12
S382	UNION	5	23	s01824.1	OAR23_12771349.1	6055.77	102
S383		152	21	OAR21_52385914.1	s53160.1	3090.45	40
S383		844	21	s75988.1	OAR21_54464294.1	7093.13	95
S383		123	21	s30026.1	s11366.1	5205.16	71
S383		627	21	s03044.1	OAR21_54143578.1	3949.9	61
S383		90	21	s23363.1	s53160.1	4496.72	61
S383	CON	5	21	OAR21_52385914.1	s11366.1	44.973	2
S383	UNION	5	21	s30026.1	s53160.1	8250.64	109

S390		507	21	s26077.1	s59900.1	2557.94	53
S390		140	21	s75799.1	OAR21_38087037.1	2579.68	44
S390		27	21	s25809.1	s13923.1	11828.3	168
S390		90	21	s75804.1	s55303.1	6697.66	125
S390		829	21	s75799.1	s08492.1	2047.17	32
S390	CON	5	21	s26077.1	s08492.1	805.544	19
S390	UNION	5	21	s25809.1	s13923.1	11828.3	168
S391		140	20	s16031.1	s12267.1	1880.87	40
S391		27	20	s58254.1	s12267.1	2049.4	44
S391		61	20	s03715.1	s68014.1	1734.93	32
S391		29	20	s38719.1	OAR20_17775022.1	4200.7	84
S391		123	20	s03715.1	OAR20_15759478.1	1449.45	30
S391	CON	5	20	s16031.1	OAR20_15759478.1	602.633	14
S391	UNION	5	20	s38719.1	OAR20_17775022.1	4200.7	84
S394		123	19	s06628.1	s48005.1	14962.5	245
S394		61	19	s68645.1	s10765.1	3926.28	59
S394		616	19	OAR19_49208953.1	s75263.1	1971.38	34
S394		702	19	OAR19_50399194.1	s46131.1	2585.32	37
S394		140	19	s45366.1	s34745.1	3419.3	49
S394	CON	5	19	s45366.1	s75263.1	553.421	10
S394	UNION	5	19	s06628.1	s48005.1	14962.5	245
S398		844	18	OAR18_66384520.1	s52137.1	1484.08	32
S398		29	18	s07565.1	s67955.1	1775.03	40
S398		79	18	OAR18_65638912.1	s07559.1	1232.2	30
S398		507	18	OAR18_65660908.1	OAR18_68832375.1	3171.47	67
S398		627	18	s28124.1	s67734.1	2172.87	45
S398	CON	5	18	s28124.1	s07559.1	305.595	9
S398	UNION	5	18	OAR18_65638912.1	OAR18_68832375.1	3193.46	68
S399		27	17	s67104.1	OAR17_67852025.1	2502.39	44
S399		606	17	s72201.1	OAR17_70179693.1	5790.57	96
S399		627	17	s46474.1	OAR17_65490341.1	8079.56	133
S399		507	17	s47176.1	s49020.1	26754.9	433
S399		702	17	OAR17_36966397.1	OAR17_74039265.1	37072.9	600
S399	CON	5	17	s67104.1	OAR17_65490341.1	140.707	3
S399	UNION	5	17	OAR17_36966397.1	OAR17_74039265.1	37072.9	600
S400		140	17	OAR17_46861268.1	s22393.1	3230.08	51
S400		702	17	OAR17_36966397.1	OAR17_74039265.1	37072.9	600
S400		157	17	OAR17_43682916.1	s10999.1	5855.49	91
S400		79	17	OAR17_44276820.1	OAR17_50838067.1	6561.25	100
S400		507	17	s47176.1	s49020.1	26754.9	433
S400	CON	5	17	OAR17_46861268.1	s10999.1	2677.14	44
S400	UNION	5	17	OAR17_36966397.1	OAR17_74039265.1	37072.9	600
S403		702	17	OAR17_36966397.1	OAR17_74039265.1	37072.9	600
S403		140	17	s32577.1	s24812.1	4527.89	80
S403		157	17	OAR17_43682916.1	s10999.1	5855.49	91
S403		79	17	OAR17_44276820.1	OAR17_50838067.1	6561.25	100
S403		507	17	s47176.1	s49020.1	26754.9	433
S403	CON	5	17	s47176.1	s24812.1	1835.4	23
S403	UNION	5	17	OAR17_36966397.1	OAR17_74039265.1	37072.9	600

S404		79	17	OAR17_23630662.1	OAR17_25712322.1	2081.66	41
S404		702	17	OAR17_18822501.1	OAR17_23630662.1	4808.16	81
S404		627	17	OAR17_13607110.1	OAR17_23905156.1	10298	171
S404		49	17	OAR17_15114925.1	OAR17_33024300.1	17909.4	313
S404		606	17	OAR17_17231699.1	OAR17_23630662.1	6398.96	108
S404	CON	5	17	OAR17_23630662.1	OAR17_23630662.1	0	1
S404	UNION	5	17	OAR17_13607110.1	OAR17_33024300.1	19417.2	336
S405		61	17	OAR17_15290555.1	s34648.1	5841.11	97
S405		627	17	OAR17_13607110.1	OAR17_23905156.1	10298	171
S405		49	17	OAR17_15114925.1	OAR17_33024300.1	17909.4	313
S405		606	17	OAR17_17231699.1	OAR17_23630662.1	6398.96	108
S405		702	17	OAR17_18822501.1	OAR17_23630662.1	4808.16	81
S405	CON	5	17	OAR17_18822501.1	s34648.1	2309.16	37
S405	UNION	5	17	OAR17_13607110.1	OAR17_33024300.1	19417.2	336
S406		606	16	s60183.1	OAR16_49094655.1	2068.68	35
S406		629	16	s60183.1	OAR16_49094655.1	2068.68	35
S406		61	16	OAR16_32465649.1	OAR16_50643262_X.1	18177.6	326
S406		616	16	OAR16_40749182.1	OAR16_55771610.1	15022.4	264
S406		627	16	OAR16_48105833.1	OAR16_51462528.1	3356.7	61
S406	CON	5	16	OAR16_48105833.1	OAR16_49094655.1	988.822	21
S406	UNION	5	16	OAR16_32465649.1	OAR16_55771610.1	23306	414
S407		61	16	OAR16_23680997.1	OAR16_25765286.1	2084.29	37
S407		629	16	OAR16_23634510.1	OAR16_26684182.1	3049.67	52
S407		123	16	s42829.1	s00347.1	3521.21	61
S407		507	16	OAR16_22384075.1	OAR16_23939306.1	1555.23	31
S407		606	16	s61872.1	OAR16_32278875.1	10181.3	193
S407	CON	5	16	OAR16_23680997.1	OAR16_23939306.1	258.309	3
S407	UNION	5	16	s61872.1	OAR16_32278875.1	10181.3	193
S413		507	16	OAR16_18182900.1	s47010.1	1410.34	34
S413		140	16	OAR16_18125193.1	s55433.1	1342.03	32
S413		79	16	OAR16_16552788.1	OAR16_19068087.1	2515.3	52
S413		40	16	s38913.1	OAR16_18865349.1	5499.5	103
S413		702	16	OAR16_11050320.1	OAR16_20062365.1	9012.05	173
S413	CON	5	16	OAR16_18182900.1	OAR16_18865349.1	682.449	17
S413	UNION	5	16	OAR16_11050320.1	OAR16_20062365.1	9012.05	173
S420		627	15	OAR15_41465316.1	OAR15_53192586.1	11727.3	150
S420		629	15	s05399.1	s61861.1	1488.93	30
S420		126	15	s60771.1	s31640.1	1782.1	38
S420		40	15	OAR15_39858472.1	s31640.1	1857.95	39
S420		90	15	OAR15_41465316.1	OAR15_46781842.1	5316.53	97
S420	CON	5	15	OAR15_41465316.1	s31640.1	251.104	4
S420	UNION	5	15	OAR15_39858472.1	OAR15_53192586.1	13334.1	185
S425		702	13	s43103.1	s69997.1	32663.3	559
S425		844	13	s17883.1	OAR13_68528151.1	4897.41	78
S425		123	13	s28942.1	s21696.1	2426.28	36
S425		616	13	s35279.1	OAR13_69559303_X.1	2999.91	45
S425		49	13	OAR13_66795163.1	s49551.1	2397.51	37
S425	CON	5	13	OAR13_66795163.1	OAR13_68528151.1	1732.99	25

S425	UNION	5	13	s43103.1	s69997.1	32663.3	559
S426		616	13	s35279.1	OAR13_69559303_X.1	2999.91	45
S426		90	13	s00952.1	s35279.1	2317.41	44
S426		702	13	s43103.1	s69997.1	32663.3	559
S426		844	13	s17883.1	OAR13_68528151.1	4897.41	78
S426		123	13	s28942.1	s21696.1	2426.28	36
S426	CON	5	13	s35279.1	s35279.1	0	1
S426	UNION	5	13	s43103.1	s69997.1	32663.3	559
S430		801	13	s62501.1	s18127.1	1817.63	37
S430		844	13	OAR13_47720904.1	s20479.1	6338.95	104
S430		702	13	s43103.1	s69997.1	32663.3	559
S430		123	13	s61846.1	OAR13_50758390.1	3333.51	67
S430		556	13	s36887.1	OAR13_53493947.1	4913.95	79
S430	CON	5	13	s36887.1	s18127.1	1090.22	21
S430	UNION	5	13	s43103.1	s69997.1	32663.3	559
S435		45	13	OAR13_39447377.1	s32471.1	2176.24	31
S435		49	13	OAR13_39447377.1	s32471.1	2176.24	31
S435		556	13	s18674.1	OAR13_44631782.1	5987.19	98
S435		29	13	OAR13_39146165.1	OAR13_41484865.1	2338.7	37
S435		629	13	OAR13_34785098.1	s67974.1	5171.07	99
S435	CON	5	13	OAR13_39447377.1	s67974.1	508.789	8
S435	UNION	5	13	OAR13_34785098.1	OAR13_44631782.1	9846.68	171
S439		606	13	OAR13_35686621.1	s56749.1	1739.76	38
S439		90	13	OAR13_35648372.1	s26568.1	2163.61	45
S439		629	13	OAR13_34785098.1	s67974.1	5171.07	99
S439		829	13	s29601.1	OAR13_37876562.1	14843.5	275
S439		27	13	OAR13_35076469.1	OAR13_37625153_X.1	2548.68	49
S439	CON	5	13	OAR13_35686621.1	s56749.1	1739.76	38
S439	UNION	5	13	s29601.1	s67974.1	16923.1	318
S440		27	13	OAR13_35076469.1	OAR13_37625153_X.1	2548.68	49
S440		629	13	OAR13_34785098.1	s67974.1	5171.07	99
S440		90	13	OAR13_31557833.1	OAR13_35413855.1	3856.02	66
S440		29	13	s62936.1	OAR13_35198687.1	9473.28	175
S440		829	13	s29601.1	OAR13_37876562.1	14843.5	275
S440	CON	5	13	OAR13_35076469.1	OAR13_35198687.1	122.218	3
S440	UNION	5	13	s29601.1	s67974.1	16923.1	318
S441		627	12	s63637.1	OAR12_51498192.1	2978.12	59
S441		629	12	s63637.1	OAR12_51498192.1	2978.12	59
S441		123	12	OAR12_30882746.1	OAR12_62371899.1	31489.2	574
S441		702	12	s22960.1	s60710.1	58755.9	1088
S441		606	12	OAR12_43758965.1	OAR12_49228109.1	5469.14	102
S441	CON	5	12	s63637.1	OAR12_49228109.1	708.042	15
S441	UNION	5	12	s22960.1	s60710.1	58755.9	1088
S442		629	12	OAR12_32481363.1	OAR12_34756947.1	2275.58	51
S442		844	12	OAR12_31951136.1	OAR12_34168645.1	2217.51	52
S442		702	12	s22960.1	s60710.1	58755.9	1088
S442		27	12	OAR12_28316888.1	s57344.1	11454	242
S442		123	12	OAR12_30882746.1	OAR12_62371899.1	31489.2	574

S442	CON	5	12	OAR12_32481363.1	OAR12_34168645.1	1687.28	40
S442	UNION	5	12	s22960.1	s60710.1	58755.9	1088
S443		123	12	OAR12_30882746.1	OAR12_62371899.1	31489.2	574
S443		61	12	s57656.1	s23923.1	1878.72	36
S443		507	12	s13792.1	s57817.1	31188.2	514
S443		702	12	s22960.1	s60710.1	58755.9	1088
S443		27	12	OAR12_28316888.1	s57344.1	11454	242
S443	CON	5	12	OAR12_30882746.1	s23923.1	248.28	3
S443	UNION	5	12	s13792.1	s60710.1	71900.8	1272
S444		61	12	s57656.1	s23923.1	1878.72	36
S444		507	12	s13792.1	s57817.1	31188.2	514
S444		152	12	OAR12_29307422.1	OAR12_30823763.1	1516.34	30
S444		702	12	s22960.1	s60710.1	58755.9	1088
S444		27	12	OAR12_28316888.1	s57344.1	11454	242
S444	CON	5	12	OAR12_29307422.1	OAR12_30823763.1	1516.34	30
S444	UNION	5	12	s13792.1	s60710.1	71900.8	1272
S445		629	12	OAR12_21022562.1	OAR12_22840561.1	1818	37
S445		140	12	OAR12_20168901.1	OAR12_22539169.1	2370.27	50
S445		507	12	s13792.1	s57817.1	31188.2	514
S445		702	12	s22960.1	s60710.1	58755.9	1088
S445		606	12	OAR12_21699429.1	s14801.1	3790.5	59
S445	CON	5	12	OAR12_21699429.1	OAR12_22539169.1	839.74	20
S445	UNION	5	12	s13792.1	s60710.1	71900.8	1272
S460		627	11	OAR11_55335521.1	s36067.1	1982.73	35
S460		629	11	s13742.1	OAR11_61687914.1	12951	212
S460		152	11	OAR11_57189920.1	OAR11_61713996.1	4524.08	72
S460		702	11	OAR11_57135966.1	OAR11_63846580.1	6710.61	109
S460		61	11	OAR11_55421823.1	s36067.1	1896.43	33
S460	CON	5	11	OAR11_57189920.1	s36067.1	128.336	3
S460	UNION	5	11	s13742.1	OAR11_63846580.1	15109.6	249
S469		152	11	s26188.1	s54753.1	1956.04	35
S469		90	11	s07556.1	OAR11_55421823.1	8158.13	135
S469		627	11	OAR11_55335521.1	s36067.1	1982.73	35
S469		629	11	s13742.1	OAR11_61687914.1	12951	212
S469		61	11	OAR11_55421823.1	s36067.1	1896.43	33
S469	CON	5	11	OAR11_55421823.1	OAR11_55421823.1	0	1
S469	UNION	5	11	s07556.1	OAR11_61687914.1	14424.2	235
S470		140	11	s32976.1	OAR11_21925396.1	3582.11	65
S470		79	11	s32976.1	s33117.1	1526.31	32
S470		627	11	OAR11_17704915.1	s29885.1	1485.24	31
S470		702	11	OAR11_17172323.1	OAR11_33359712.1	16187.4	260
S470		829	11	s00452.1	s27347.1	1796.09	37
S470	CON	5	11	s00452.1	s29885.1	787.547	20
S470	UNION	5	11	OAR11_17172323.1	OAR11_33359712.1	16187.4	260
S471		61	10	OAR10_89779166.1	s26573.1	2219.27	47
S471		629	10	OAR10_89733307.1	s37245.1	2199.09	46
S471		556	10	s12759.1	s10349.1	2436.29	52
S471		45	10	OAR10_88694929.1	s26573.1	3303.51	74

S471		829	10	OAR10_89779166.1	s49917.1	1486.99	30
S471	CON	5	10	OAR10_89779166.1	s49917.1	1486.99	30
S471	UNION	5	10	OAR10_88694929.1	s10349.1	3374.14	75
S474		40	10	OAR10_62361045.1	OAR10_66989523.1	4628.48	66
S474		27	10	OAR10_58094557.1	OAR10_62988399.1	4893.84	90
S474		157	10	OAR10_57171204.1	OAR10_62988399.1	5817.19	110
S474		152	10	OAR10_56312119.1	OAR10_72914358.1	16602.2	277
S474		90	10	s25185.1	OAR10_68005037.1	11797.8	188
S474	CON	5	10	OAR10_62361045.1	OAR10_62988399.1	627.354	19
S474	UNION	5	10	s25185.1	OAR10_72914358.1	16707.1	280
S475		157	10	OAR10_57171204.1	OAR10_62988399.1	5817.19	110
S475		152	10	OAR10_56312119.1	OAR10_72914358.1	16602.2	277
S475		45	10	OAR10_35722333.1	OAR10_57396545.1	21674.2	405
S475		507	10	OAR10_54127756.1	s31288.1	7114.03	124
S475		90	10	s25185.1	OAR10_68005037.1	11797.8	188
S475	CON	5	10	OAR10_57171204.1	OAR10_57396545.1	225.341	6
S475	UNION	5	10	OAR10_35722333.1	OAR10_72914358.1	37192	664
S476		157	10	OAR10_44615697.1	OAR10_46220373.1	1604.68	31
S476		844	10	OAR10_42283457.1	OAR10_45140128.1	2856.67	42
S476		702	10	s49567.1	OAR10_48462141.1	48462.1	914
S476		829	10	OAR10_33047233.1	OAR10_51969493.1	18922.3	352
S476		45	10	OAR10_35722333.1	OAR10_57396545.1	21674.2	405
S476	CON	5	10	OAR10_44615697.1	OAR10_45140128.1	524.431	10
S476	UNION	5	10	s49567.1	OAR10_57396545.1	57396.5	1095
S487		627	10	s14601.1	OAR10_7959969.1	1766.61	31
S487		629	10	OAR10_5271954.1	OAR10_7507728.1	2235.77	34
S487		702	10	s49567.1	OAR10_48462141.1	48462.1	914
S487		126	10	s49567.1	OAR10_9585529.1	9585.53	173
S487		61	10	OAR10_5663903.1	OAR10_7959969.1	2296.07	37
S487	CON	5	10	s14601.1	OAR10_7507728.1	1314.37	22
S487	UNION	5	10	s49567.1	OAR10_48462141.1	48462.1	914
S490		27	9	s28418.1	s60249.1	1605.72	34
S490		616	9	OAR9_85044633.1	s64103.1	2852.17	62
S490		29	9	s64667.1	OAR9_86159320.1	1397.64	37
S490		702	9	OAR9_38323108.1	OAR9_96544082.1	58221	1087
S490		140	9	s32207.1	OAR9_86395078.1	1208.25	31
S490	CON	5	9	s28418.1	OAR9_86159320.1	704.634	20
S490	UNION	5	9	OAR9_38323108.1	OAR9_96544082.1	58221	1087
S491		49	9	OAR9_61830651.1	OAR9_64551132.1	2720.48	58
S491		629	9	OAR9_55140044.1	OAR9_63193973.1	8053.93	166
S491		702	9	OAR9_38323108.1	OAR9_96544082.1	58221	1087
S491		79	9	OAR9_59326545.1	OAR9_62308628.1	2982.08	60
S491		27	9	OAR9_61212902.1	OAR9_65555411.1	4342.51	93
S491	CON	5	9	OAR9_61830651.1	OAR9_62308628.1	477.977	13
S491	UNION	5	9	OAR9_38323108.1	OAR9_96544082.1	58221	1087
S492		45	9	s68634.1	s50783.1	21048.3	358
S492		61	9	OAR9_31664097.1	OAR9_36052313.1	4388.22	101
S492		49	9	OAR9_35522648_X.1	s32613.1	1441.19	33

S492		606	9	OAR9_32239874.1	DU427730_369.1	4216.13	94
S492		79	9	s73465.1	OAR9_40771150.1	8319.46	179
S492	CON	5	9	s68634.1	OAR9_36052313.1	113.97	4
S492	UNION	5	9	OAR9_31664097.1	s50783.1	25322.6	455
S493		507	9	s75518.1	OAR9_26587263.1	2962.39	66
S493		61	9	OAR9_23382060.1	s56435.1	7434.21	145
S493		45	9	OAR9_18493683.1	s19052.1	5564.34	125
S493		629	9	s60705.1	OAR9_25022978.1	2352.47	57
S493		90	9	s19462.1	s01999.1	3928.4	85
S493	CON	5	9	s75518.1	s19052.1	433.147	12
S493	UNION	5	9	OAR9_18493683.1	s56435.1	12322.6	252
S495		801	9	s54533.1	s34916.1	1404.37	32
S495		829	9	s08221.1	OAR9_22512802.1	3869.72	83
S495		157	9	OAR9_20964415.1	OAR9_22488751.1	1524.34	35
S495		844	9	OAR9_20308781.1	s36366.1	1348.29	30
S495		45	9	OAR9_18493683.1	s19052.1	5564.34	125
S495	CON	5	9	OAR9_20964415.1	s36366.1	692.657	18
S495	UNION	5	9	OAR9_18493683.1	s19052.1	5564.34	125
S496		801	9	s54533.1	s34916.1	1404.37	32
S496		829	9	s08221.1	OAR9_22512802.1	3869.72	83
S496		126	9	s59135.1	OAR9_20804687.1	2341.2	49
S496		45	9	OAR9_18493683.1	s19052.1	5564.34	125
S496		844	9	OAR9_20308781.1	s36366.1	1348.29	30
S496	CON	5	9	s54533.1	OAR9_20804687.1	369.545	8
S496	UNION	5	9	s59135.1	s19052.1	5594.54	126
S501		606	8	OAR8_95188605.1	OAR8_97813729.1	2625.12	46
S501		157	8	s63436.1	OAR8_97813729.1	2689.46	47
S501		140	8	s28231.1	OAR8_97813729.1	2862.02	51
S501		829	8	s26722.1	OAR8_95919216.1	1453.08	31
S501		702	8	DU492158_335.1	OAR8_97813729.1	1947.16	32
S501	CON	5	8	DU492158_335.1	OAR8_95919216.1	52.651	4
S501	UNION	5	8	s26722.1	OAR8_97813729.1	3347.59	59
S502		507	8	OAR8_85811057.1	s41250.1	2810.84	53
S502		829	8	OAR8_84622702.1	OAR8_85978703.1	1356	32
S502		152	8	OAR8_84448034.1	s73153.1	1906.25	44
S502		79	8	OAR8_81635650.1	OAR8_87540028.1	5904.38	127
S502		49	8	OAR8_77578890.1	s01826.1	8664.11	175
S502	CON	5	8	OAR8_85811057.1	OAR8_85978703.1	167.646	5
S502	UNION	5	8	OAR8_77578890.1	s41250.1	11043	217
S503		627	8	OAR8_58134022.1	OAR8_62425494.1	4291.47	87
S503		629	8	OAR8_58134022.1	OAR8_62425494.1	4291.47	87
S503		152	8	s15412.1	OAR8_60953046.1	3152.82	68
S503		801	8	s15412.1	s59082.1	2721.52	57
S503		507	8	s00060.1	OAR8_60076974.1	6422.45	116
S503	CON	5	8	OAR8_58134022.1	OAR8_60076974.1	1942.95	41
S503	UNION	5	8	s00060.1	OAR8_62425494.1	8770.97	162
S506		152	8	OAR8_31142913.1	s09657.1	2031.21	41
S506		126	8	s69453.1	s50983.1	4232.26	88

S506		61	8	s42406.1	OAR8_37553450.1	5987.56	109
S506		27	8	OAR8_29706230.1	s32939.1	3476.82	69
S506		702	8	OAR8_26793121.1	OAR8_38710483.1	11917.4	231
S506	CON	5	8	s42406.1	s50983.1	836.493	18
S506	UNION	5	8	OAR8_26793121.1	OAR8_38710483.1	11917.4	231
S507		507	7	s63808.1	s09265.1	2218.56	49
S507		606	7	OAR7_86067111.1	s67662.1	5534.67	111
S507		123	7	s13905.1	OAR7_91483736.1	9054.19	169
S507		79	7	s24581.1	s45225.1	1594.56	35
S507		629	7	s63353.1	OAR7_89553402.1	1361.65	30
S507	CON	5	7	s24581.1	OAR7_89553402.1	1138.35	25
S507	UNION	5	7	s13905.1	s67662.1	9172.24	171
S512		616	7	s03655.1	OAR7_83970535.1	2138.66	41
S512		844	7	s03357.1	s61284.1	4208.81	84
S512		61	7	OAR7_79189143.1	s15556.1	5289.18	104
S512		123	7	s13905.1	OAR7_91483736.1	9054.19	169
S512		627	7	OAR7_83694999.1	s51006.1	1980.89	35
S512	CON	5	7	OAR7_83694999.1	OAR7_83970535.1	275.536	6
S512	UNION	5	7	OAR7_79189143.1	OAR7_91483736.1	12294.6	237
S513		90	7	OAR7_75710157.1	OAR7_78025265.1	2315.11	46
S513		123	7	s26564.1	OAR7_77993710.1	3439.34	64
S513		29	7	OAR7_56240366.1	OAR7_77667178.1	21426.8	390
S513		507	7	OAR7_74283088.1	s36799.1	3100.68	60
S513		49	7	OAR7_75483123.1	OAR7_78083739.1	2600.62	54
S513	CON	5	7	OAR7_75710157.1	s36799.1	1673.61	35
S513	UNION	5	7	OAR7_56240366.1	OAR7_78083739.1	21843.4	398
S515		123	7	s03425.1	OAR7_70071978.1	2562.47	40
S515		606	7	OAR7_64620807.1	OAR7_67914634.1	3293.83	62
S515		702	7	OAR7_31685912.1	OAR7_72973471.1	41287.6	769
S515		507	7	OAR7_52043940.1	OAR7_71832636.1	19788.7	355
S515		29	7	OAR7_56240366.1	OAR7_77667178.1	21426.8	390
S515	CON	5	7	s03425.1	OAR7_67914634.1	405.124	10
S515	UNION	5	7	OAR7_31685912.1	OAR7_77667178.1	45981.3	865
S516		844	7	OAR7_62972902.1	OAR7_64550152.1	1577.25	30
S516		606	7	OAR7_61396840.1	OAR7_63848145.1	2451.3	41
S516		702	7	OAR7_31685912.1	OAR7_72973471.1	41287.6	769
S516		507	7	OAR7_52043940.1	OAR7_71832636.1	19788.7	355
S516		29	7	OAR7_56240366.1	OAR7_77667178.1	21426.8	390
S516	CON	5	7	OAR7_62972902.1	OAR7_63848145.1	875.243	17
S516	UNION	5	7	OAR7_31685912.1	OAR7_77667178.1	45981.3	865
S520		45	7	OAR7_31172405.1	OAR7_58958307.1	27785.9	542
S520		702	7	OAR7_31685912.1	OAR7_72973471.1	41287.6	769
S520		507	7	OAR7_52043940.1	OAR7_71832636.1	19788.7	355
S520		844	7	OAR7_52759167.1	s62604.1	2272.8	40
S520		616	7	OAR7_54780217.1	s36119.1	5323.48	103
S520	CON	5	7	OAR7_54780217.1	s62604.1	251.749	7
S520	UNION	5	7	OAR7_31172405.1	OAR7_72973471.1	41801.1	782
S521		45	7	OAR7_31172405.1	OAR7_58958307.1	27785.9	542

S521		702	7	OAR7_31685912.1	OAR7_72973471.1	41287.6	769
S521		27	7	OAR7_51824628.1	s56915.1	1393.42	30
S521		507	7	OAR7_52043940.1	OAR7_71832636.1	19788.7	355
S521		844	7	OAR7_52759167.1	s62604.1	2272.8	40
S521	CON	5	7	OAR7_52759167.1	s56915.1	458.877	10
S521	UNION	5	7	OAR7_31172405.1	OAR7_72973471.1	41801.1	782
S522		45	7	OAR7_31172405.1	OAR7_58958307.1	27785.9	542
S522		702	7	OAR7_31685912.1	OAR7_72973471.1	41287.6	769
S522		606	7	s01468.1	OAR7_50490234.1	5451.27	98
S522		49	7	OAR7_46559347.1	s73999.1	2867.31	47
S522		123	7	s02669.1	OAR7_50322674.1	1244.94	30
S522	CON	5	7	s02669.1	s73999.1	348.919	8
S522	UNION	5	7	OAR7_31172405.1	OAR7_72973471.1	41801.1	782
S523		49	7	OAR7_46559347.1	s73999.1	2867.31	47
S523		40	7	OAR7_43593010.1	OAR7_46818598.1	3225.59	57
S523		45	7	OAR7_31172405.1	OAR7_58958307.1	27785.9	542
S523		702	7	OAR7_31685912.1	OAR7_72973471.1	41287.6	769
S523		606	7	s01468.1	OAR7_50490234.1	5451.27	98
S523	CON	5	7	OAR7_46559347.1	OAR7_46818598.1	259.251	5
S523	UNION	5	7	OAR7_31172405.1	OAR7_72973471.1	41801.1	782
S524		40	7	OAR7_43593010.1	OAR7_46818598.1	3225.59	57
S524		90	7	OAR7_43374818.1	OAR7_46455757.1	3080.94	57
S524		45	7	OAR7_31172405.1	OAR7_58958307.1	27785.9	542
S524		702	7	OAR7_31685912.1	OAR7_72973471.1	41287.6	769
S524		606	7	s01468.1	OAR7_50490234.1	5451.27	98
S524	CON	5	7	s01468.1	OAR7_46455757.1	1416.8	23
S524	UNION	5	7	OAR7_31172405.1	OAR7_72973471.1	41801.1	782
S525		140	6	OAR6_52856067.1	OAR6_55285058.1	2428.99	47
S525		79	6	OAR6_51660421.1	s21191.1	10134.5	185
S525		629	6	OAR6_50229073_X.1	DU523483_560.1	2868.91	57
S525		627	6	OAR6_48108133.1	DU523483_560.1	4989.86	104
S525		702	6	s10352.1	OAR6_55321610.1	5954.38	118
S525	CON	5	6	OAR6_52856067.1	DU523483_560.1	241.922	7
S525	UNION	5	6	OAR6_48108133.1	s21191.1	13686.8	263
S529		629	6	OAR6_50229073_X.1	DU523483_560.1	2868.91	57
S529		627	6	OAR6_48108133.1	DU523483_560.1	4989.86	104
S529		45	6	OAR6_38919831.1	OAR6_50590550.1	11670.7	219
S529		29	6	s50591.1	OAR6_50860226.1	1669.38	39
S529		702	6	s10352.1	OAR6_55321610.1	5954.38	118
S529	CON	5	6	OAR6_50229073_X.1	OAR6_50590550.1	361.476	9
S529	UNION	5	6	OAR6_38919831.1	OAR6_55321610.1	16401.8	308
S530		29	6	s50591.1	OAR6_50860226.1	1669.38	39
S530		140	6	s73122.1	OAR6_49463357.1	26607.7	513
S530		627	6	OAR6_48108133.1	DU523483_560.1	4989.86	104
S530		45	6	OAR6_38919831.1	OAR6_50590550.1	11670.7	219
S530		702	6	s10352.1	OAR6_55321610.1	5954.38	118
S530	CON	5	6	s10352.1	OAR6_49463357.1	96.13	3
S530	UNION	5	6	s73122.1	OAR6_55321610.1	32465.9	628

S531		29	5	OAR5_99590053_X.1	OAR5_102165646.1	2575.59	53
S531		61	5	s48412.1	OAR5_102547933.1	4624.25	102
S531		627	5	s09048.1	OAR5_100090515.1	6457.02	134
S531		702	5	OAR5_77977258.1	OAR5_100127181.1	22149.9	428
S531		606	5	OAR5_98030869.1	OAR5_100699982.1	2669.11	58
S531	CON	5	5	OAR5_99590053_X.1	OAR5_100090515.1	500.461	11
S531	UNION	5	5	OAR5_77977258.1	OAR5_102547933.1	24570.7	480
S534		27	5	OAR5_79542638.1	s15500.1	5963.32	115
S534		629	5	OAR5_76098058.1	OAR5_81196810.1	5098.75	99
S534		606	5	OAR5_70256606.1	OAR5_82901477.1	12644.9	245
S534		702	5	OAR5_77977258.1	OAR5_100127181.1	22149.9	428
S534		844	5	OAR5_79669328.1	OAR5_82093890.1	2424.56	46
S534	CON	5	5	OAR5_79669328.1	OAR5_81196810.1	1527.48	30
S534	UNION	5	5	OAR5_70256606.1	OAR5_100127181.1	29870.6	580
S535		627	5	OAR5_69570106.1	OAR5_71156734.1	1586.63	30
S535		801	5	s11947.1	OAR5_69909638.1	1818.53	34
S535		123	5	OAR5_67426945.1	OAR5_70029869.1	2602.92	48
S535		126	5	s52618.1	OAR5_72565538.1	7244.72	139
S535		629	5	OAR5_57983508.1	OAR5_71824766.1	13841.3	230
S535	CON	5	5	OAR5_69570106.1	OAR5_69909638.1	339.532	7
S535	UNION	5	5	OAR5_57983508.1	OAR5_72565538.1	14582	241
S536		507	5	OAR5_49532219.1	s07760.1	3268.6	64
S536		152	5	s12940.1	OAR5_61924626.1	14110.5	253
S536		801	5	OAR5_47774570.1	s54250.1	7553.49	151
S536		140	5	OAR5_52351898.1	OAR5_58830573.1	6478.68	118
S536		556	5	OAR5_50919104.1	s07760.1	1881.71	35
S536	CON	5	5	OAR5_52351898.1	s07760.1	448.918	8
S536	UNION	5	5	OAR5_47774570.1	OAR5_61924626.1	14150.1	254
S540		627	5	s71007.1	s04774.1	5048.15	85
S540		629	5	s71007.1	s04774.1	5048.15	85
S540		152	5	s12940.1	OAR5_61924626.1	14110.5	253
S540		801	5	OAR5_47774570.1	s54250.1	7553.49	151
S540		157	5	s37412.1	s21988.1	1297.99	34
S540	CON	5	5	s37412.1	s04774.1	390.301	11
S540	UNION	5	5	s71007.1	OAR5_61924626.1	18107.3	314
S541		627	5	s71007.1	s04774.1	5048.15	85
S541		629	5	s71007.1	s04774.1	5048.15	85
S541		45	5	s71007.1	s06750.1	4481.91	70
S541		152	5	s12940.1	OAR5_61924626.1	14110.5	253
S541		801	5	OAR5_47774570.1	s54250.1	7553.49	151
S541	CON	5	5	s12940.1	s06750.1	485.088	9
S541	UNION	5	5	s71007.1	OAR5_61924626.1	18107.3	314
S544		844	5	s59427.1	s48689.1	1975.08	36
S544		140	5	s59427.1	OAR5_19459135.1	1893.42	34
S544		629	5	OAR5_17235395.1	s17924.1	1949.73	32
S544		61	5	OAR5_12770106.1	OAR5_22276454.1	9506.35	152
S544		45	5	OAR5_10884558.1	s06935.1	11513.7	188
S544	CON	5	5	s59427.1	s17924.1	1619.41	28
S544	UNION	5	5	OAR5_10884558.1	s06935.1	11513.7	188

S545		157	4	OAR4_61284730.1	s64810.1	2274.83	45
S545		27	4	DU194821_342.1	OAR4_61284730.1	14228.1	279
S545		29	4	DU194821_342.1	OAR4_61284730.1	14228.1	279
S545		702	4	s58367.1	OAR4_85483475.1	25992.5	514
S545		152	4	OAR4_60126261.1	OAR4_62657058.1	2530.8	55
S545	CON	5	4	OAR4_61284730.1	OAR4_61284730.1	0	1
S545	UNION	5	4	DU194821_342.1	OAR4_85483475.1	38426.9	752
S546		606	4	OAR4_56704521.1	s00703.1	2537.16	45
S546		27	4	DU194821_342.1	OAR4_61284730.1	14228.1	279
S546		29	4	DU194821_342.1	OAR4_61284730.1	14228.1	279
S546		627	4	OAR4_55669092.1	OAR4_60497897.1	4828.81	98
S546		45	4	OAR4_55744716.1	OAR4_60926085.1	5181.37	106
S546	CON	5	4	OAR4_56704521.1	s00703.1	2537.16	45
S546	UNION	5	4	DU194821_342.1	OAR4_61284730.1	14228.1	279
S553		79	4	OAR4_44621948.1	s02754.1	1716.92	31
S553		45	4	OAR4_43198108.1	s67196.1	8975.51	158
S553		844	4	OAR4_42488888.1	OAR4_45213543_X.1	2724.66	51
S553		61	4	OAR4_39813653.1	OAR4_56340637.1	16527	308
S553		606	4	OAR4_37940876.1	OAR4_55963926.1	18023	344
S553	CON	5	4	OAR4_44621948.1	OAR4_45213543_X.1	591.596	13
S553	UNION	5	4	OAR4_37940876.1	OAR4_56340637.1	18399.8	352
S554		627	4	OAR4_31796734.1	OAR4_36438393.1	4641.66	71
S554		152	4	OAR4_30536811.1	OAR4_32059279.1	1522.47	34
S554		844	4	OAR4_29851251.1	OAR4_31996653.1	2145.4	46
S554		157	4	OAR4_29369957.1	OAR4_33022606_X.1	3652.65	73
S554		606	4	OAR4_29369957.1	OAR4_32671208.1	3301.25	67
S554	CON	5	4	OAR4_31796734.1	OAR4_31996653.1	199.919	4
S554	UNION	5	4	OAR4_29369957.1	OAR4_36438393.1	7068.44	120
S555		829	4	OAR4_3610903.1	OAR4_5767890.1	2156.99	39
S555		702	4	OARUn.284_293028.1	OAR4_24251309.1	24251.3	470
S555		629	4	OAR4_5638574.1	OAR4_7712644.1	2074.07	44
S555		29	4	OAR4_742452.1	OAR4_5847332.1	5104.88	85
S555		61	4	OAR4_3630456.1	s45567.1	2316.36	42
S555	CON	5	4	OAR4_5638574.1	OAR4_5767890.1	129.316	3
S555	UNION	5	4	OARUn.284_293028.1	OAR4_24251309.1	24251.3	470
S568		152	3	s32572.1	s24635.1	3235.68	40
S568		79	3	OAR3_217297404.1	OAR3_218733455.1	1436.05	32
S568		627	3	OAR3_216714207.1	s32572.1	1813.47	38
S568		629	3	OAR3_216714207.1	s32572.1	1813.47	38
S568		616	3	OAR3_209072421.1	OAR3_222052522.1	12980.1	230
S568	CON	5	3	s32572.1	s32572.1	0	1
S568	UNION	5	3	OAR3_209072421.1	OAR3_222052522.1	12980.1	230
S569		123	3	s09050.1	OAR3_202943170.1	11054.8	191
S569		627	3	s00725.1	OAR3_192440268.1	1619.58	33
S569		629	3	s00725.1	OAR3_192440268.1	1619.58	33
S569		702	3	OAR3_190128314.1	OAR3_194730311.1	4602	79
S569		507	3	OAR3_179244393.1	OAR3_211362922_X.1	32118.5	604
S569	CON	5	3	s09050.1	OAR3_192440268.1	551.943	13

S569	UNION	5	3	OAR3_179244393.1	OAR3_211362922_X.1	32118.5	604
S570		627	3	OAR3_155236610.1	s75549.1	19415.4	375
S570		629	3	OAR3_155236610.1	s75549.1	19415.4	375
S570		126	3	OAR3_152741614.1	OAR3_163942175.1	11200.6	212
S570		27	3	OAR3_156830410.1	OAR3_158745008_X.1	1914.6	36
S570		507	3	OAR3_156766007_X.1	OAR3_158584850_X.1	1818.84	33
S570	CON	5	3	OAR3_156830410.1	OAR3_158584850_X.1	1754.44	32
S570	UNION	5	3	OAR3_152741614.1	s75549.1	21910.4	419
S580		45	3	OAR3_134591031.1	OAR3_135863920.1	1272.89	30
S580		616	3	OAR3_134547477.1	OAR3_136948456.1	2400.98	51
S580		152	3	OAR3_134370389.1	OAR3_137497257.1	3126.87	67
S580		90	3	OAR3_129044189.1	OAR3_134747538.1	5703.35	108
S580		61	3	OAR3_134416842.1	OAR3_136398703.1	1981.86	43
S580	CON	5	3	OAR3_134591031.1	OAR3_134747538.1	156.507	3
S580	UNION	5	3	OAR3_129044189.1	OAR3_137497257.1	8453.07	166
S581		801	3	OAR3_115651452.1	OAR3_118053045.1	2401.59	50
S581		702	3	OAR3_115651452.1	OAR3_117843556.1	2192.1	44
S581		629	3	OAR3_112096089.1	OAR3_121762751.1	9666.66	172
S581		152	3	OAR3_115651452.1	s09346.1	3379.64	69
S581		616	3	OAR3_115651452.1	OAR3_118690316.1	3038.86	63
S581	CON	5	3	OAR3_115651452.1	OAR3_117843556.1	2192.1	44
S581	UNION	5	3	OAR3_112096089.1	OAR3_121762751.1	9666.66	172
S582		801	3	s65769.1	s18106.1	5900.44	108
S582		702	3	s04344.1	s28807.1	1280.72	33
S582		27	3	OAR3_108120929.1	s42653.1	2413.72	44
S582		79	3	s00521.1	s51141.1	3995.25	72
S582		29	3	s29822.1	s42653.1	3721.94	77
S582	CON	5	3	OAR3_108120929.1	s28807.1	169.787	4
S582	UNION	5	3	s29822.1	s18106.1	6131.25	113
S583		152	3	OAR3_78511530.1	OAR3_80763380.1	2251.85	51
S583		507	3	OAR3_77718162.1	OAR3_82856188.1	5138.03	112
S583		702	3	OAR3_78338656.1	s45788.1	2098.32	48
S583		123	3	OAR3_78338656.1	s45788.1	2098.32	48
S583		49	3	OAR3_79159004.1	OAR3_80722364.1	1563.36	38
S583	CON	5	3	OAR3_79159004.1	s45788.1	1277.97	32
S583	UNION	5	3	OAR3_77718162.1	OAR3_82856188.1	5138.03	112
S585		702	3	s57378.1	OAR3_74933537.1	1989.28	43
S585		45	3	OAR3_70204674.1	s57378.1	2739.58	51
S585		829	3	OAR3_67058161.1	OAR3_74604120.1	7545.96	148
S585		157	3	OAR3_70578986.1	OAR3_76536013.1	5957.03	117
S585		801	3	OAR3_72317520.1	OAR3_74181493.1	1863.97	40
S585	CON	5	3	s57378.1	s57378.1	0	1
S585	UNION	5	3	OAR3_67058161.1	OAR3_76536013.1	9477.85	187
S588		829	3	OAR3_67058161.1	OAR3_74604120.1	7545.96	148
S588		27	3	OAR3_64474656.1	OAR3_67137932.1	2663.28	53
S588		49	3	OAR3_66440567_X.1	OAR3_68372978.1	1932.41	42
S588		61	3	s40974.1	OAR3_68372978.1	2366.7	50
S588		123	3	s24884.1	OAR3_67751047.1	1949.75	42

S588	CON	5	3	OAR3_67058161.1	OAR3_67137932.1	79.771	3
S588	UNION	5	3	OAR3_64474656.1	OAR3_74604120.1	10129.5	198
S592		27	3	OAR3_64474656.1	OAR3_67137932.1	2663.28	53
S592		40	3	OAR3_64474656.1	OAR3_66798124.1	2323.47	45
S592		49	3	OAR3_66440567_X.1	OAR3_68372978.1	1932.41	42
S592		61	3	s40974.1	OAR3_68372978.1	2366.7	50
S592		123	3	s24884.1	OAR3_67751047.1	1949.75	42
S592	CON	5	3	OAR3_66440567_X.1	OAR3_66798124.1	357.557	7
S592	UNION	5	3	OAR3_64474656.1	OAR3_68372978.1	3898.32	80
S593		27	3	OAR3_64474656.1	OAR3_67137932.1	2663.28	53
S593		40	3	OAR3_64474656.1	OAR3_66798124.1	2323.47	45
S593		627	3	OAR3_64474656.1	OAR3_66317995.1	1843.34	37
S593		45	3	s45049.1	OAR3_66317995.1	4167.71	82
S593		702	3	s50340.1	s35506.1	2560.33	53
S593	CON	5	3	OAR3_64474656.1	s35506.1	299.148	9
S593	UNION	5	3	s45049.1	OAR3_67137932.1	4987.65	98
S595		27	3	OAR3_18901084_X.1	OAR3_22495716.1	3594.63	81
S595		61	3	s65199.1	s62291.1	2512.95	62
S595		702	3	DU259120_464.1	OAR3_59494261.1	59494.3	1111
S595		79	3	OAR3_17718190.1	OAR3_24559969.1	6841.78	153
S595		507	3	s49768.1	OAR3_28567790.1	9061.66	193
S595	CON	5	3	s49768.1	s62291.1	789.164	20
S595	UNION	5	3	DU259120_464.1	OAR3_59494261.1	59494.3	1111
S598		123	3	s36039.1	OAR3_18087923.1	2271.09	47
S598		629	3	s36039.1	OAR3_18087923.1	2271.09	47
S598		702	3	DU259120_464.1	OAR3_59494261.1	59494.3	1111
S598		79	3	OAR3_17718190.1	OAR3_24559969.1	6841.78	153
S598		61	3	s65199.1	s62291.1	2512.95	62
S598	CON	5	3	s65199.1	OAR3_18087923.1	305.577	9
S598	UNION	5	3	DU259120_464.1	OAR3_59494261.1	59494.3	1111
S603		79	2	s71223.1	s55376.1	1830.55	40
S603		627	2	OAR2_257231205.1	OAR2_260008602.1	2777.4	52
S603		629	2	OAR2_257231205.1	OAR2_260008602.1	2777.4	52
S603		123	2	OAR2_258146403_X.1	s71223.1	1445.95	30
S603		702	2	OAR2_252278273.1	s29579.1	10830.2	215
S603	CON	5	2	s71223.1	s71223.1	0	1
S603	UNION	5	2	OAR2_252278273.1	s29579.1	10830.2	215
S611		702	2	OAR2_252278273.1	s29579.1	10830.2	215
S611		29	2	OAR2_238444943.1	s43939.1	16224.6	314
S611		606	2	OAR2_250716720.1	OAR2_253423292_X.1	2706.57	51
S611		801	2	OAR2_251639875.1	s61009.1	1651.34	32
S611		829	2	s19317.1	OAR2_253758268_X.1	1765.93	31
S611	CON	5	2	OAR2_252278273.1	s61009.1	1012.95	21
S611	UNION	5	2	OAR2_238444943.1	s29579.1	24663.6	487
S612		629	2	OAR2_242229131.1	DU503161_123.1	7148.91	146
S612		627	2	OAR2_238165550.1	OAR2_246045571.1	7880.02	156
S612		123	2	OAR2_235417778.1	OAR2_244336911.1	8919.13	185
S612		29	2	OAR2_238444943.1	s43939.1	16224.6	314

S612		844	2	OAR2_243206127.1	OAR2_245289710.1	2083.58	41
S612	CON	5	2	OAR2_243206127.1	OAR2_244336911.1	1130.78	24
S612	UNION	5	2	OAR2_235417778.1	s43939.1	19251.7	381
S615		90	2	OAR2_170073165.1	OAR2_173940252.1	3867.09	87
S615		140	2	OAR2_169232830.1	OAR2_172379160.1	3146.33	68
S615		606	2	OAR2_169232830.1	s72026.1	1949.14	41
S615		507	2	OAR2_167852063.1	OAR2_173821801.1	5969.74	125
S615		123	2	OAR2_167959232.1	OAR2_176091475.1	8132.24	171
S615	CON	5	2	OAR2_170073165.1	s72026.1	1108.81	26
S615	UNION	5	2	OAR2_167852063.1	OAR2_176091475.1	8239.41	174
S616		152	2	s39150.1	OAR2_166756467.1	5092.89	98
S616		507	2	OAR2_158519928.1	OAR2_163536981.1	5017.05	97
S616		702	2	s54108.1	OAR2_162167662.1	23623.2	482
S616		90	2	OAR2_160912478.1	s22217.1	3583.91	72
S616		40	2	s39150.1	s70789.1	3202.21	62
S616	CON	5	2	s39150.1	OAR2_162167662.1	504.088	11
S616	UNION	5	2	s54108.1	OAR2_166756467.1	28212	569
S617		507	2	OAR2_158519928.1	OAR2_163536981.1	5017.05	97
S617		152	2	OAR2_157048544.1	OAR2_159191106.1	2142.56	43
S617		627	2	OAR2_155425432.1	OAR2_160296103.1	4870.67	100
S617		606	2	s01065.1	s72813.1	6497.5	138
S617		702	2	s54108.1	OAR2_162167662.1	23623.2	482
S617	CON	5	2	OAR2_158519928.1	OAR2_159191106.1	671.178	13
S617	UNION	5	2	s54108.1	OAR2_163536981.1	24992.5	505
S619		45	2	s52793.1	OAR2_74821030.1	1573.02	33
S619		29	2	OAR2_73146917.1	OAR2_74924055.1	1777.14	37
S619		49	2	OAR2_61907594.1	OAR2_74652390.1	12744.8	265
S619		61	2	OAR2_72802015.1	OAR2_80118756.1	7316.74	138
S619		606	2	OAR2_74253113.1	OAR2_82226321.1	7973.21	148
S619	CON	5	2	OAR2_74253113.1	OAR2_74652390.1	399.277	10
S619	UNION	5	2	OAR2_61907594.1	OAR2_82226321.1	20318.7	403
S620		45	2	OAR2_65431232.1	OAR2_67968106.1	2536.87	50
S620		40	2	OAR2_63943896.1	s59858.1	2994.2	62
S620		29	2	OAR2_63911376.1	OAR2_66880170.1	2968.79	62
S620		507	2	OAR2_63911376.1	OAR2_65914681.1	2003.31	43
S620		49	2	OAR2_61907594.1	OAR2_74652390.1	12744.8	265
S620	CON	5	2	OAR2_65431232.1	OAR2_65914681.1	483.449	10
S620	UNION	5	2	OAR2_61907594.1	OAR2_74652390.1	12744.8	265
S621		152	2	OAR2_52795503.1	OAR2_54554938.1	1759.43	38
S621		45	2	OAR2_52795503.1	OAR2_54554938.1	1759.43	38
S621		507	2	s37403.1	s36747.1	4416.51	74
S621		79	2	OAR2_49013850.1	s36747.1	5342.23	88
S621		29	2	s05611.1	OAR2_55308142.1	7693.97	128
S621	CON	5	2	OAR2_52795503.1	s36747.1	1560.57	35
S621	UNION	5	2	s05611.1	OAR2_55308142.1	7693.97	128
S623		507	1	s22571.1	s56772.1	15398.3	291
S623		702	1	OAR1_282017475.1	s67262.1	9551.48	171
S623		45	1	s00865.1	OAR1_292547071.1	1801.06	41

S623		844	1	s30252.1	s15782.1	2657.67	55
S623		61	1	OAR1_291297885.1	s65284.1	7463.92	143
S623	CON	5	1	OAR1_291297885.1	s67262.1	271.067	7
S623	UNION	5	1	s22571.1	s65284.1	17454.1	322
S629		29	1	OAR1_283569518.1	s39199.1	5956.3	95
S629		844	1	OAR1_281552674.1	s62624.1	2326.12	51
S629		507	1	s22571.1	s56772.1	15398.3	291
S629		90	1	OAR1_280918505.1	s59682.1	4159.05	86
S629		702	1	OAR1_282017475.1	s67262.1	9551.48	171
S629	CON	5	1	OAR1_283569518.1	s62624.1	309.272	7
S629	UNION	5	1	OAR1_280918505.1	s56772.1	15787.5	299
S630		844	1	OAR1_281552674.1	s62624.1	2326.12	51
S630		507	1	s22571.1	s56772.1	15398.3	291
S630		90	1	OAR1_280918505.1	s59682.1	4159.05	86
S630		29	1	OAR1_280132963.1	s44037.1	2616.65	54
S630		702	1	OAR1_282017475.1	s67262.1	9551.48	171
S630	CON	5	1	OAR1_282017475.1	s44037.1	732.14	15
S630	UNION	5	1	OAR1_280132963.1	s56772.1	16573	315
S631		126	1	OAR1_253349819.1	s70277.1	12807	217
S631		829	1	OAR1_257557854.1	s49280.1	5797.95	97
S631		606	1	OAR1_262265433.1	OAR1_264697118.1	2431.68	44
S631		152	1	OAR1_262561308.1	OAR1_266535457.1	3974.15	71
S631		507	1	s27023.1	s70277.1	2887.16	50
S631	CON	5	1	s27023.1	s49280.1	86.116	3
S631	UNION	5	1	OAR1_253349819.1	OAR1_266535457.1	13185.6	224
S632		126	1	s40188.1	OAR1_241703558.1	8537.63	145
S632		152	1	OAR1_230994116.1	OAR1_233249488.1	2255.37	43
S632		40	1	OAR1_230374318.1	OAR1_234437646.1	4063.33	74
S632		829	1	OAR1_219136030.1	OAR1_236103073.1	16967	322
S632		702	1	OAR1_228131256.1	OAR1_233756581.1	5625.32	96
S632	CON	5	1	s40188.1	OAR1_233249488.1	83.56	2
S632	UNION	5	1	OAR1_219136030.1	OAR1_241703558.1	22567.5	406
S636		123	1	OAR1_221370736.1	OAR1_222987599.1	1616.86	31
S636		829	1	OAR1_219136030.1	OAR1_236103073.1	16967	322
S636		629	1	OAR1_214417389.1	OAR1_233116174.1	18698.8	341
S636		702	1	OAR1_201628760.1	OAR1_224818104.1	23189.3	424
S636		90	1	OAR1_222537672.1	OAR1_230460155.1	7922.48	157
S636	CON	5	1	OAR1_222537672.1	OAR1_222987599.1	449.927	10
S636	UNION	5	1	OAR1_201628760.1	OAR1_236103073.1	34474.3	639
S642		123	1	OAR1_221370736.1	OAR1_222987599.1	1616.86	31
S642		829	1	OAR1_219136030.1	OAR1_236103073.1	16967	322
S642		629	1	OAR1_214417389.1	OAR1_233116174.1	18698.8	341
S642		702	1	OAR1_201628760.1	OAR1_224818104.1	23189.3	424
S642		627	1	s00549.1	OAR1_221878496.1	2683.75	44
S642	CON	5	1	OAR1_221370736.1	OAR1_221878496.1	507.76	8
S642	UNION	5	1	OAR1_201628760.1	OAR1_236103073.1	34474.3	639
S643		829	1	OAR1_219136030.1	OAR1_236103073.1	16967	322
S643		629	1	OAR1_214417389.1	OAR1_233116174.1	18698.8	341

S643		702	1	OAR1_201628760.1	OAR1_224818104.1	23189.3	424
S643		126	1	OAR1_217543314.1	OAR1_219952416.1	2409.1	36
S643		627	1	s00549.1	OAR1_221878496.1	2683.75	44
S643	CON	5	1	s00549.1	OAR1_219952416.1	757.672	15
S643	UNION	5	1	OAR1_201628760.1	OAR1_236103073.1	34474.3	639
S644		829	1	s66109.1	s65811.1	2617.61	35
S644		123	1	s30590.1	OAR1_201878358.1	3641.45	75
S644		801	1	OAR1_198827329.1	s56333.1	6897.16	121
S644		157	1	DU287626_225.1 250	506CS3900283200001_442.1	2218.24	32
S644		702	1	OAR1_201628760.1	OAR1_224818104.1	23189.3	424
S644	CON	5	1	s66109.1	OAR1_201878358.1	59.136	3
S644	UNION	5	1	s30590.1	OAR1_224818104.1	26581.2	493
S651		606	1	s12579.1	OAR1_193526104.1	9327.73	173
S651		627	1	s12579.1	OAR1_190617487.1	6419.11	121
S651		629	1	s12579.1	OAR1_190617487.1	6419.11	121
S651		157	1	OAR1_185953850.1	s57353.1	2652.55	54
S651		45	1	s00576.1	s17408.1	6283.1	119
S651	CON	5	1	OAR1_185953850.1	s57353.1	2652.55	54
S651	UNION	5	1	s00576.1	OAR1_193526104.1	9394.42	175
S652		606	1	s12579.1	OAR1_193526104.1	9327.73	173
S652		627	1	s12579.1	OAR1_190617487.1	6419.11	121
S652		629	1	s12579.1	OAR1_190617487.1	6419.11	121
S652		79	1	OAR1_182020254.1	OAR1_185615587.1	3595.33	73
S652		45	1	s00576.1	s17408.1	6283.1	119
S652	CON	5	1	s12579.1	OAR1_185615587.1	1417.21	28
S652	UNION	5	1	OAR1_182020254.1	OAR1_193526104.1	11505.9	218
S654		79	1	OAR1_149565545.1	OAR1_151312088.1	1746.54	42
S654		61	1	OAR1_148262943.1	OAR1_151312088.1	3049.14	67
S654		123	1	s53597.1	OAR1_153662211.1	35798.6	631
S654		702	1	s14189.1	s40751.1	42088.8	712
S654		90	1	OAR1_148901159.1	OAR1_151247138.1	2345.98	54
S654	CON	5	1	OAR1_149565545.1	s40751.1	247.384	7
S654	UNION	5	1	s14189.1	OAR1_153662211.1	45938.1	791
S655		702	1	s14189.1	s40751.1	42088.8	712
S655		123	1	s53597.1	OAR1_153662211.1	35798.6	631
S655		829	1	OAR1_145497863.1	OAR1_149463910.1	3966.05	64
S655		61	1	OAR1_148262943.1	OAR1_151312088.1	3049.14	67
S655		90	1	OAR1_148901159.1	OAR1_151247138.1	2345.98	54
S655	CON	5	1	OAR1_148901159.1	OAR1_149463910.1	562.751	14
S655	UNION	5	1	s14189.1	OAR1_153662211.1	45938.1	791
S658		61	1	OAR1_148262943.1	OAR1_151312088.1	3049.14	67
S658		79	1	OAR1_143829113.1	OAR1_148557968.1	4728.85	72
S658		123	1	s53597.1	OAR1_153662211.1	35798.6	631
S658		702	1	s14189.1	s40751.1	42088.8	712
S658		829	1	OAR1_145497863.1	OAR1_149463910.1	3966.05	64
S658	CON	5	1	OAR1_148262943.1	OAR1_148557968.1	295.025	5
S658	UNION	5	1	s14189.1	OAR1_153662211.1	45938.1	791
S659		79	1	OAR1_143829113.1	OAR1_148557968.1	4728.85	72

S659		123	1	s53597.1	OAR1_153662211.1	35798.6	631
S659		702	1	s14189.1	s40751.1	42088.8	712
S659		606	1	DU354639_611.1	OAR1_145810797.1	6263.2	106
S659		829	1	OAR1_145497863.1	OAR1_149463910.1	3966.05	64
S659	CON	5	1	OAR1_145497863.1	OAR1_145810797.1	312.934	5
S659	UNION	5	1	s14189.1	OAR1_153662211.1	45938.1	791
S660		606	1	DU354639_611.1	OAR1_145810797.1	6263.2	106
S660		27	1	s63741.1	OAR1_143333465.1	4008.69	70
S660		627	1	s03118.1	OAR1_141731333.1	16512	283
S660		702	1	s14189.1	s40751.1	42088.8	712
S660		123	1	s53597.1	OAR1_153662211.1	35798.6	631
S660	CON	5	1	DU354639_611.1	OAR1_141731333.1	2183.74	37
S660	UNION	5	1	s14189.1	OAR1_153662211.1	45938.1	791
S661		45	1	s37607.1	s04671.1	16346.9	247
S661		49	1	OAR1_113402761.1	s75030.1	4288.36	49
S661		702	1	s14189.1	s40751.1	42088.8	712
S661		801	1	OAR1_110989185.1	s10939.1	7522.21	114
S661		556	1	OAR1_112929344.1	OAR1_117762259.1	4832.91	61
S661	CON	5	1	s37607.1	s75030.1	2661.79	32
S661	UNION	5	1	s14189.1	s40751.1	42088.8	712
S667		616	1	s16707.1	OAR1_74015424.1	3018.78	60
S667		507	1	OAR1_67421568.1	OAR1_71254279.1	3832.71	57
S667		702	1	OAR1_65265541_X.1	OAR1_73281352.1	8015.81	136
S667		90	1	OAR1_66156513.1	OAR1_74623022.1	8466.51	147
S667		61	1	s58781.1	s29884.1	9573.23	170
S667	CON	5	1	s16707.1	OAR1_71254279.1	257.632	6
S667	UNION	5	1	OAR1_65265541_X.1	s29884.1	12598.8	227
S668		606	1	OAR1_44090814.1	OAR1_51307088.1	7216.27	123
S668		45	1	OAR1_44090814.1	OAR1_47087569.1	2996.76	55
S668		507	1	OAR1_43083702.1	s14904.1	5678.21	99
S668		702	1	OAR1_7717464.1	OAR1_58938560.1	51221.1	934
S668		844	1	OAR1_44241542.1	OAR1_49456350.1	5214.81	94
S668	CON	5	1	OAR1_44241542.1	OAR1_47087569.1	2846.03	52
S668	UNION	5	1	OAR1_7717464.1	OAR1_58938560.1	51221.1	934
S669		90	1	OAR1_16750809.1	OAR1_19152280.1	2401.47	43
S669		79	1	s73231.1	OAR1_16795469.1	3934.26	80
S669		606	1	OAR1_15578534.1	s69885.1	12559.2	218
S669		702	1	OAR1_7717464.1	OAR1_58938560.1	51221.1	934
S669		627	1	OAR1_15256499.1	OAR1_18052854.1	2796.36	53
S669	CON	5	1	OAR1_16750809.1	OAR1_16795469.1	44.66	3
S669	UNION	5	1	OAR1_7717464.1	OAR1_58938560.1	51221.1	934
S672		606	26	OAR26_42480765.1	OAR26_44441243.1	1960.48	34
S672		627	26	s72956.1	OAR26_44089889.1	1679.27	32
S672		45	26	s70488.1	OAR26_42514266.1	6953.15	122
S672		61	26	s63463.1	OAR26_46995252.1	7812.86	140
S672	CON	4	26	OAR26_42480765.1	OAR26_42514266.1	33.501	2
S672	UNION	4	26	s70488.1	OAR26_46995252.1	11434.1	195
S673		27	26	OAR26_19029894.1	s08067.1	9710.75	157

S673		29	26	OAR26_19029894.1	OAR26_25594436.1	6564.54	94
S673		606	26	OAR26_16835791.1	OAR26_25248695.1	8412.9	137
S673		152	26	s66187.1	OAR26_20847538.1	4834.37	90
S673	CON	4	26	OAR26_19029894.1	OAR26_20847538.1	1817.64	29
S673	UNION	4	26	s66187.1	s08067.1	12727.5	218
S674		829	26	OAR26_8625199.1	OAR26_10403629.1	1778.43	34
S674		90	26	OAR26_6073159.1	OAR26_10130406.1	4057.25	46
S674		844	26	OAR26_8709366.1	OAR26_10981172.1	2271.81	41
S674		126	26	OAR26_8625199.1	OAR26_13494685.1	4869.49	83
S674	CON	4	26	OAR26_8709366.1	OAR26_10130406.1	1421.04	29
S674	UNION	4	26	OAR26_6073159.1	OAR26_13494685.1	7421.53	99
S679		629	25	s32866.1	OAR25_31259191.1	2423.01	48
S679		126	25	OAR25_24120855.1	OAR25_29970035.1	5849.18	104
S679		507	25	s10320.1	s12031.1	20755.8	401
S679		45	25	OAR25_24491716.1	OAR25_29853310.1	5361.59	95
S679	CON	4	25	s32866.1	s12031.1	536.013	15
S679	UNION	4	25	s10320.1	OAR25_31259191.1	22642.8	434
S680		507	25	s10320.1	s12031.1	20755.8	401
S680		844	25	OAR25_19516269.1	OAR25_25861480.1	6345.21	122
S680		126	25	OAR25_24120855.1	OAR25_29970035.1	5849.18	104
S680		45	25	OAR25_24491716.1	OAR25_29853310.1	5361.59	95
S680	CON	4	25	OAR25_24491716.1	OAR25_25861480.1	1369.76	26
S680	UNION	4	25	s10320.1	OAR25_29970035.1	21353.7	409
S681		507	25	s10320.1	s12031.1	20755.8	401
S681		61	25	OAR25_12492866.1	OAR25_21044148.1	8551.28	175
S681		616	25	OAR25_14447424.1	OAR25_15709970.1	1262.55	30
S681		152	25	OAR25_15029968.1	OAR25_16672918.1	1642.95	34
S681	CON	4	25	OAR25_15029968.1	OAR25_15709970.1	680.002	16
S681	UNION	4	25	s10320.1	s12031.1	20755.8	401
S682		90	25	OAR25_10160789.1	s21640.1	3276.28	60
S682		844	25	OAR25_9879901.1	OAR25_11438585.1	1558.68	31
S682		507	25	s10320.1	s12031.1	20755.8	401
S682		152	25	OAR25_10480244.1	OAR25_14279951.1	3799.71	71
S682	CON	4	25	OAR25_10480244.1	OAR25_11438585.1	958.341	19
S682	UNION	4	25	s10320.1	s12031.1	20755.8	401
S683		79	25	s21107.1	s10114.1	2215.18	43
S683		627	25	s52797.1	s09722.1	2190.12	43
S683		49	25	OAR25_5894736.1	OAR25_7752816.1	1858.08	35
S683		45	25	s58408.1	s21107.1	1832.56	31
S683	CON	4	25	s21107.1	s21107.1	0	1
S683	UNION	4	25	s58408.1	s10114.1	4047.74	73
S688		844	25	s42361.1	s39664.1	1600.34	35
S688		61	25	s42361.1	s41841.1	1784.14	38
S688		29	25	s42361.1	OAR25_2252731.1	2252.73	47
S688		90	25	s12866.1	s21472.1	4810.44	79
S688	CON	4	25	s12866.1	s39664.1	1600.34	34
S688	UNION	4	25	s42361.1	s21472.1	4810.44	80

S694		126	24	s31084.1	OAR24_7741373.1	1842.11	35
S694		157	24	s68231.1	DU281388_299.1	1742.96	30
S694		844	24	s53625.1	s04817.1	6789.77	105
S694		702	24	s35472.1	OAR24_22201078.1	16855.5	259
S694	CON	4	24	s31084.1	DU281388_299.1	347.164	6
S694	UNION	4	24	s53625.1	OAR24_22201078.1	21083.7	319
S698		29	24	s69582.1	OAR24_4058568.1	4058.57	50
S698		140	24	s41842.1	OAR24_4058568.1	4058.57	51
S698		61	24	s45142.1	s74400.1	3026.07	36
S698		844	24	s53625.1	s04817.1	6789.77	105
S698	CON	4	24	s53625.1	s74400.1	1908.73	23
S698	UNION	4	24	s45142.1	s04817.1	7907.1	118
S703		27	23	s58136.1	OAR23_60124720.1	9675.72	139
S703		29	23	s58136.1	OAR23_60124720.1	9675.72	139
S703		123	23	OAR23_55806264.1	OAR23_59401394.1	3595.13	53
S703		844	23	s17206.1	OAR23_60961233.1	2559.8	38
S703	CON	4	23	s17206.1	OAR23_59401394.1	999.966	14
S703	UNION	4	23	s58136.1	OAR23_60961233.1	10512.2	153
S704		61	23	s40890.1	s23488.1	2039.45	34
S704		45	23	OAR23_32717464.1	s03900.1	8953.01	139
S704		702	23	OAR23_32970524.1	s47536.1	10557	158
S704		140	23	OAR23_33045102.1	s30665.1	2503.87	39
S704	CON	4	23	OAR23_33045102.1	s23488.1	871.498	14
S704	UNION	4	23	s40890.1	s47536.1	11650.4	176
S712		702	23	OAR23_32970524.1	s47536.1	10557	158
S712		140	23	OAR23_30906493.1	OAR23_32970524.1	2064.03	32
S712		61	23	s40890.1	s23488.1	2039.45	34
S712		45	23	OAR23_32717464.1	s03900.1	8953.01	139
S712	CON	4	23	OAR23_32970524.1	OAR23_32970524.1	0	1
S712	UNION	4	23	OAR23_30906493.1	s47536.1	12621.1	189
S713		45	23	OAR23_32717464.1	s03900.1	8953.01	139
S713		616	23	OAR23_30686953.1	OAR23_32932392_X.1	2245.44	33
S713		140	23	OAR23_30906493.1	OAR23_32970524.1	2064.03	32
S713		61	23	s40890.1	s23488.1	2039.45	34
S713	CON	4	23	OAR23_32717464.1	OAR23_32932392_X.1	214.929	5
S713	UNION	4	23	OAR23_30686953.1	s03900.1	10983.5	167
S715		844	22	s75260.1	s49093.1	2055.87	43
S715		507	22	s29080.1	OAR22_49444571.1	3407.59	66
S715		61	22	s24975.1	OAR22_48881341.1	1818.67	34
S715		556	22	s41217.1	OAR22_49529205.1	1894.93	40
S715	CON	4	22	s41217.1	OAR22_48881341.1	1247.07	24
S715	UNION	4	22	s29080.1	OAR22_49529205.1	3492.22	68
S716		507	22	s06758.1	OAR22_42355166.1	24436.4	466
S716		40	22	OAR22_39226599.1	s63122.1	3096.53	64
S716		140	22	OAR22_39672861.1	s60513.1	2063.93	43
S716		61	22	s13679.1	OAR22_41976427.1	1582.02	36
S716	CON	4	22	s13679.1	s60513.1	1342.38	31
S716	UNION	4	22	s06758.1	OAR22_42355166.1	24436.4	466

S719		49	21	s74518.1	s38134.1	13941.9	212
S719		606	21	OAR21_21865772.1	OAR21_36571389.1	14705.6	232
S719		844	21	OAR21_23862060.1	s47821.1	8308.09	126
S719		152	21	OAR21_25759271.1	OAR21_27500331.1	1741.06	31
S719	CON	4	21	OAR21_25759271.1	OAR21_27500331.1	1741.06	31
S719	UNION	4	21	s74518.1	OAR21_36571389.1	17643.3	276
S722		45	21	OAR21_19280450.1	OAR21_24102988.1	4822.54	78
S722		49	21	s74518.1	s38134.1	13941.9	212
S722		801	21	OAR21_17659120.1	s64572.1	2030.63	35
S722		79	21	OAR21_16500504.1	OAR21_24084777.1	7584.27	125
S722	CON	4	21	OAR21_19280450.1	s64572.1	409.299	4
S722	UNION	4	21	OAR21_16500504.1	s38134.1	16369.5	255
S723		616	21	s07207.1	OAR21_11371256.1	2929.72	51
S723		27	21	OAR21_6702302.1	s41655.1	5418.27	94
S723		702	21	s74845.1	OAR21_17189778.1	17189.8	241
S723		123	21	OAR21_7901164.1	OAR21_11513066.1	3611.9	63
S723	CON	4	21	s07207.1	OAR21_11371256.1	2929.72	51
S723	UNION	4	21	s74845.1	OAR21_17189778.1	17189.8	241
S724		29	20	OAR20_45542235.1	s37556.1	1477.59	36
S724		606	20	s40462.1	s37556.1	1520.29	37
S724		844	20	OAR20_42071000_X.1	s25696.1	3826.95	84
S724		61	20	s64834.1	s49617.1	1868.62	41
S724	CON	4	20	OAR20_45542235.1	s25696.1	355.715	8
S724	UNION	4	20	OAR20_42071000_X.1	s37556.1	4948.82	112
S727		27	20	OAR20_42482053.1	s54536.1	2596.59	58
S727		844	20	OAR20_42071000_X.1	s25696.1	3826.95	84
S727		616	20	s09155.1	s64274.1	2115.44	47
S727		61	20	s64834.1	s49617.1	1868.62	41
S727	CON	4	20	s64834.1	s64274.1	779.162	18
S727	UNION	4	20	OAR20_42071000_X.1	s49617.1	3991.17	88
S728		61	20	s28948.1	OAR20_35185147.1	4569.52	68
S728		27	20	s28948.1	OAR20_34756048.1	4140.42	61
S728		45	20	s72198.1	OAR20_34436224.1	7964.15	94
S728		140	20	OAR20_26794433.1	OAR20_33616292.1	6821.86	77
S728	CON	4	20	s28948.1	OAR20_33616292.1	3000.66	41
S728	UNION	4	20	s72198.1	OAR20_35185147.1	8713.08	109
S729		627	20	s44306.1	s06051.1	3639.25	77
S729		140	20	s16031.1	s12267.1	1880.87	40
S729		27	20	s58254.1	s12267.1	2049.4	44
S729		29	20	s38719.1	OAR20_17775022.1	4200.7	84
S729	CON	4	20	s44306.1	s12267.1	852.561	21
S729	UNION	4	20	s38719.1	s06051.1	6250.08	128
S735		27	20	OAR20_11442511.1	s59954.1	2223.77	40
S735		29	20	OAR20_10918132_X.1	s54709.1	2576.65	50
S735		702	20	OAR20_27119.1	OAR20_14072832.1	14045.7	264
S735		90	20	s34560.1	s74774.1	5360.26	107
S735	CON	4	20	OAR20_11442511.1	s74774.1	1915.04	36

S735	UNION	4	20	OAR20_27119.1	OAR20_14072832.1	14045.7	264
S736		61	19	s68645.1	s10765.1	3926.28	59
S736		829	19	s63765.1	s58019.1	1368.46	30
S736		123	19	s06628.1	s48005.1	14962.5	245
S736		616	19	OAR19_49208953.1	s75263.1	1971.38	34
S736	CON	4	19	OAR19_49208953.1	s58019.1	62.386	2
S736	UNION	4	19	s06628.1	s48005.1	14962.5	245
S737		61	19	s68645.1	s10765.1	3926.28	59
S737		829	19	s63765.1	s58019.1	1368.46	30
S737		29	19	OAR19_45775486.1	s68645.1	3399.83	67
S737		123	19	s06628.1	s48005.1	14962.5	245
S737	CON	4	19	s68645.1	s68645.1	0	1
S737	UNION	4	19	s06628.1	s48005.1	14962.5	245
S741		702	19	OAR19_41101734.1	OAR19_48025918.1	6924.18	121
S741		123	19	s06628.1	s48005.1	14962.5	245
S741		29	19	OAR19_45775486.1	s68645.1	3399.83	67
S741		829	19	s63765.1	s58019.1	1368.46	30
S741	CON	4	19	s63765.1	OAR19_48025918.1	123.044	4
S741	UNION	4	19	OAR19_41101734.1	s48005.1	15700.5	260
S742		702	19	OAR19_41101734.1	OAR19_48025918.1	6924.18	121
S742		123	19	s06628.1	s48005.1	14962.5	245
S742		152	19	OAR19_42964158.1	OAR19_46545362.1	3581.2	57
S742		29	19	OAR19_45775486.1	s68645.1	3399.83	67
S742	CON	4	19	OAR19_45775486.1	OAR19_46545362.1	769.876	13
S742	UNION	4	19	OAR19_41101734.1	s48005.1	15700.5	260
S756		123	19	s06628.1	s48005.1	14962.5	245
S756		61	19	s43852.1	OAR19_43892533.1	2709.91	48
S756		702	19	OAR19_41101734.1	OAR19_48025918.1	6924.18	121
S756		152	19	OAR19_42964158.1	OAR19_46545362.1	3581.2	57
S756	CON	4	19	OAR19_42964158.1	OAR19_43892533.1	928.375	16
S756	UNION	4	19	OAR19_41101734.1	s48005.1	15700.5	260
S759		507	19	s25457.1	OAR19_41558943.1	6509.38	133
S759		90	19	OAR19_39869628.1	OAR19_41692151.1	1822.52	41
S759		702	19	OAR19_41101734.1	OAR19_48025918.1	6924.18	121
S759		61	19	s43852.1	OAR19_43892533.1	2709.91	48
S759	CON	4	19	s43852.1	OAR19_41558943.1	376.319	10
S759	UNION	4	19	s25457.1	OAR19_48025918.1	12976.4	242
S760		152	19	s09913.1	OAR19_37223995.1	2230.49	46
S760		844	19	OAR19_32284446.1	OAR19_37764352.1	5479.91	109
S760		27	19	OAR19_34533438.1	OAR19_36976172.1	2442.73	46
S760		507	19	s25457.1	OAR19_41558943.1	6509.38	133
S760	CON	4	19	s25457.1	OAR19_36976172.1	1926.61	38
S760	UNION	4	19	OAR19_32284446.1	OAR19_41558943.1	9274.5	184
S761		27	19	OAR19_20140394.1	OAR19_23298665.1	3158.27	60
S761		29	19	OAR19_19784946.1	s01850.1	3302.34	63
S761		702	19	OAR19_7253713.1	s27711.1	16961.5	313
S761		49	19	OAR19_21684191.1	s55682.1	9977.43	163

S761	CON	4	19	OAR19_21684191.1	s01850.1	1403.09	23
S761	UNION	4	19	OAR19_7253713.1	s55682.1	24407.9	439
S762		27	19	OAR19_20140394.1	OAR19_23298665.1	3158.27	60
S762		29	19	OAR19_19784946.1	s01850.1	3302.34	63
S762		702	19	OAR19_7253713.1	s27711.1	16961.5	313
S762		79	19	OAR19_19497555.1	OAR19_21580517.1	2082.96	43
S762	CON	4	19	OAR19_20140394.1	OAR19_21580517.1	1440.12	30
S762	UNION	4	19	OAR19_7253713.1	s27711.1	16961.5	313
S763		126	19	OAR19_7959420.1	OAR19_9490656.1	1531.24	34
S763		79	19	OAR19_6316471.1	s23781.1	2364.11	40
S763		45	19	OAR19_3773707.1	OAR19_13260250.1	9486.54	172
S763		702	19	OAR19_7253713.1	s27711.1	16961.5	313
S763	CON	4	19	OAR19_7959420.1	s23781.1	721.16	13
S763	UNION	4	19	OAR19_3773707.1	s27711.1	20441.5	368
S764		140	18	OAR18_54263457.1	OAR18_57166462.1	2903.01	35
S764		507	18	OAR18_52361774.1	OAR18_56351698.1	3989.92	47
S764		152	18	OAR18_51594268.1	OAR18_55121773.1	3527.51	53
S764		606	18	OAR18_52219091.1	s59424.1	5545.32	70
S764	CON	4	18	OAR18_54263457.1	OAR18_55121773.1	858.316	14
S764	UNION	4	18	OAR18_51594268.1	s59424.1	6170.15	84
S767		40	17	s25119.1	OAR17_52473999.1	2202.59	36
S767		79	17	OAR17_44276820.1	OAR17_50838067.1	6561.25	100
S767		702	17	OAR17_36966397.1	OAR17_74039265.1	37072.9	600
S767		507	17	s47176.1	s49020.1	26754.9	433
S767	CON	4	17	s25119.1	OAR17_50838067.1	566.657	11
S767	UNION	4	17	OAR17_36966397.1	OAR17_74039265.1	37072.9	600
S770		702	17	OAR17_36966397.1	OAR17_74039265.1	37072.9	600
S770		40	17	OAR17_38599721.1	OAR17_42515780.1	3916.06	64
S770		507	17	OAR17_40830954.1	OAR17_43503660.1	2672.71	51
S770		140	17	s32577.1	s24812.1	4527.89	80
S770	CON	4	17	s32577.1	OAR17_42515780.1	261.423	8
S770	UNION	4	17	OAR17_36966397.1	OAR17_74039265.1	37072.9	600
S771		507	17	OAR17_40830954.1	OAR17_43503660.1	2672.71	51
S771		844	17	OAR17_39208742.1	s35423.1	1819.5	31
S771		702	17	OAR17_36966397.1	OAR17_74039265.1	37072.9	600
S771		40	17	OAR17_38599721.1	OAR17_42515780.1	3916.06	64
S771	CON	4	17	OAR17_40830954.1	s35423.1	197.291	5
S771	UNION	4	17	OAR17_36966397.1	OAR17_74039265.1	37072.9	600
S772		29	16	OAR16_19414529.1	OAR16_21237906.1	1823.38	32
S772		507	16	OAR16_18182900.1	s47010.1	1410.34	34
S772		140	16	OAR16_18125193.1	s55433.1	1342.03	32
S772		702	16	OAR16_11050320.1	OAR16_20062365.1	9012.05	173
S772	CON	4	16	OAR16_19414529.1	s55433.1	52.695	2
S772	UNION	4	16	OAR16_11050320.1	OAR16_21237906.1	10187.6	193
S773		629	15	s05399.1	s61861.1	1488.93	30
S773		126	15	s60771.1	s31640.1	1782.1	38
S773		801	15	s60771.1	OAR15_41436474.1	1502.15	34

S773		40	15	OAR15_39858472.1	s31640.1	1857.95	39
S773	CON	4	15	s05399.1	OAR15_41436474.1	1069.27	24
S773	UNION	4	15	OAR15_39858472.1	s61861.1	1997.66	41
S774		629	15	s19289.1	OAR15_33315132.1	6603.26	137
S774		45	15	s70134.1	OAR15_33211632.1	5359.66	108
S774		61	15	s35266.1	s02747.1	1983.57	37
S774		123	15	OAR15_31347502.1	OAR15_32984442.1	1636.94	40
S774	CON	4	15	OAR15_31347502.1	s02747.1	443.91	11
S774	UNION	4	15	s19289.1	OAR15_33315132.1	6603.26	137
S775		123	15	OAR15_26646294.1	s74508.1	4322.48	83
S775		629	15	s19289.1	OAR15_33315132.1	6603.26	137
S775		45	15	s70134.1	OAR15_33211632.1	5359.66	108
S775		61	15	s35266.1	s02747.1	1983.57	37
S775	CON	4	15	s35266.1	s74508.1	1160.93	18
S775	UNION	4	15	OAR15_26646294.1	OAR15_33315132.1	6668.84	138
S776		45	15	s70134.1	OAR15_33211632.1	5359.66	108
S776		629	15	s19289.1	OAR15_33315132.1	6603.26	137
S776		627	15	s19289.1	s49846.1	1306.64	30
S776		123	15	OAR15_26646294.1	s74508.1	4322.48	83
S776	CON	4	15	s70134.1	s49846.1	166.542	5
S776	UNION	4	15	OAR15_26646294.1	OAR15_33315132.1	6668.84	138
S777		629	15	s19289.1	OAR15_33315132.1	6603.26	137
S777		627	15	s19289.1	s49846.1	1306.64	30
S777		123	15	OAR15_26646294.1	s74508.1	4322.48	83
S777		507	15	OAR15_24033751.1	s73229.1	2732.28	57
S777	CON	4	15	s19289.1	s73229.1	54.154	2
S777	UNION	4	15	OAR15_24033751.1	OAR15_33315132.1	9281.38	192
S778		702	15	s33423.1	s34566.1	24919.4	406
S778		126	15	OAR15_16562183.1	OAR15_22081912.1	5519.73	79
S778		45	15	s57471.1	s74813.1	2367.54	41
S778		29	15	s45138.1	s54251.1	1932.01	35
S778	CON	4	15	s45138.1	OAR15_22081912.1	135.81	4
S778	UNION	4	15	s33423.1	s34566.1	24919.4	406
S782		702	15	s33423.1	s34566.1	24919.4	406
S782		157	15	OAR15_2886961.1	s14178.1	2148.25	37
S782		27	15	OAR15_3892898.1	s07134.1	2095.32	35
S782		49	15	OAR15_4725093.1	OAR15_14317931.1	9592.84	155
S782	CON	4	15	OAR15_4725093.1	s14178.1	310.12	5
S782	UNION	4	15	s33423.1	s34566.1	24919.4	406
S786		627	14	s52668.1	s53928.1	1830.97	35
S786		629	14	s52668.1	s53928.1	1830.97	35
S786		606	14	s06052.1	s53928.1	4103.06	57
S786		702	14	OAR14_63676213.1	s42046.1	4180.8	44
S786	CON	4	14	s52668.1	s42046.1	385.002	9
S786	UNION	4	14	OAR14_63676213.1	s53928.1	5626.77	70
S792		606	14	s06052.1	s53928.1	4103.06	57
S792		123	14	s09739.1	s73257.1	7402.46	63

S792		49	14	OAR14_63676213.1	OAR14_66990507.1	3314.29	31
S792		702	14	OAR14_63676213.1	s42046.1	4180.8	44
S792	CON	4	14	s06052.1	s73257.1	74.091	3
S792	UNION	4	14	s09739.1	s53928.1	11431.4	117
S796		126	14	OAR14_58104354.1	s36676.1	6513.92	50
S796		123	14	s09739.1	s73257.1	7402.46	63
S796		49	14	OAR14_63676213.1	OAR14_66990507.1	3314.29	31
S796		702	14	OAR14_63676213.1	s42046.1	4180.8	44
S796	CON	4	14	OAR14_63676213.1	s36676.1	942.065	7
S796	UNION	4	14	s09739.1	s42046.1	9985.46	91
S797		126	14	OAR14_58104354.1	s36676.1	6513.92	50
S797		123	14	s09739.1	s73257.1	7402.46	63
S797		702	14	s52270.1	s56709.1	2277.8	36
S797		90	14	OAR14_55447588.1	OAR14_58537282.1	3089.69	43
S797	CON	4	14	OAR14_58104354.1	OAR14_58537282.1	432.928	9
S797	UNION	4	14	OAR14_55447588.1	s73257.1	9826.42	93
S803		152	14	s40481.1	OAR14_47633470.1	2753	41
S803		507	14	DU411145_363.1	s54719.1	2325.47	34
S803		157	14	s68508.1	s07549.1	2905.99	44
S803		606	14	s00394.1	s17905.1	3115.89	43
S803	CON	4	14	s68508.1	s54719.1	237.245	4
S803	UNION	4	14	DU411145_363.1	s07549.1	4994.21	74
S814		152	14	s40481.1	OAR14_47633470.1	2753	41
S814		507	14	DU411145_363.1	s54719.1	2325.47	34
S814		556	14	OAR14_41540680_X.1	OAR14_44901131.1	3360.45	52
S814		606	14	s00394.1	s17905.1	3115.89	43
S814	CON	4	14	s40481.1	OAR14_44901131.1	20.664	2
S814	UNION	4	14	OAR14_41540680_X.1	OAR14_47633470.1	6092.79	91
S815		507	14	DU411145_363.1	s54719.1	2325.47	34
S815		90	14	s10566.1	s18934.1	2141.14	32
S815		556	14	OAR14_41540680_X.1	OAR14_44901131.1	3360.45	52
S815		606	14	s00394.1	s17905.1	3115.89	43
S815	CON	4	14	s00394.1	s18934.1	670.357	8
S815	UNION	4	14	OAR14_41540680_X.1	s17905.1	5668.96	83
S816		90	14	s10566.1	s18934.1	2141.14	32
S816		61	14	s31260.1	OAR14_42706372.1	1673.04	30
S816		801	14	OAR14_38675883.1	s10566.1	3947.09	73
S816		556	14	OAR14_41540680_X.1	OAR14_44901131.1	3360.45	52
S816	CON	4	14	s10566.1	s10566.1	0	1
S816	UNION	4	14	OAR14_38675883.1	OAR14_44901131.1	6225.25	108
S817		627	14	OAR14_18935490.1	s63952.1	3002.98	58
S817		629	14	OAR14_17682700.1	OAR14_19525237.1	1842.54	32
S817		45	14	s57041.1	OAR14_19869404.1	8348.89	136
S817		702	14	s22016.1	s49153.1	8137.25	142
S817	CON	4	14	OAR14_18935490.1	OAR14_19525237.1	589.747	10
S817	UNION	4	14	s57041.1	s49153.1	14518.7	245
S818		629	14	OAR14_17682700.1	OAR14_19525237.1	1842.54	32

S818		45	14	s57041.1	OAR14_19869404.1	8348.89	136
S818		702	14	s22016.1	s49153.1	8137.25	142
S818		507	14	s19019.1	s31525.1	5170.03	82
S818	CON	4	14	s22016.1	s31525.1	118.123	3
S818	UNION	4	14	s57041.1	s49153.1	14518.7	245
S819		627	14	s56240.1	OAR14_16764006.1	4385.77	69
S819		45	14	s57041.1	OAR14_19869404.1	8348.89	136
S819		629	14	s75886.1	OAR14_16276596.1	6006.72	97
S819		507	14	s19019.1	s31525.1	5170.03	82
S819	CON	4	14	s19019.1	OAR14_16276596.1	3426.52	55
S819	UNION	4	14	s75886.1	OAR14_19869404.1	9599.53	154
S820		702	13	s61419.1	OAR13_89063022.1	2424.17	48
S820		27	13	s57248.1	OAR13_87964841.1	3134.51	57
S820		507	13	OAR13_82659110.1	OAR13_89063022.1	6403.91	118
S820		140	13	OAR13_85176472.1	OAR13_89063022.1	3886.55	70
S820	CON	4	13	s61419.1	OAR13_87964841.1	1325.99	28
S820	UNION	4	13	OAR13_82659110.1	OAR13_89063022.1	6403.91	118
S821		606	13	OAR13_60423450.1	s33005.1	3263.21	60
S821		702	13	s43103.1	s69997.1	32663.3	559
S821		40	13	s32476.1	s31201.1	1232.63	30
S821		79	13	s16241.1	s70884.1	1871.22	43
S821	CON	4	13	OAR13_60423450.1	s70884.1	586.444	15
S821	UNION	4	13	s43103.1	s69997.1	32663.3	559
S822		556	13	s18674.1	OAR13_44631782.1	5987.19	98
S822		49	13	OAR13_37697946.1	OAR13_39146165.1	1448.22	32
S822		29	13	OAR13_39146165.1	OAR13_41484865.1	2338.7	37
S822		629	13	OAR13_34785098.1	s67974.1	5171.07	99
S822	CON	4	13	OAR13_39146165.1	OAR13_39146165.1	0	1
S822	UNION	4	13	OAR13_34785098.1	OAR13_44631782.1	9846.68	171
S823		90	13	OAR13_35648372.1	s26568.1	2163.61	45
S823		629	13	OAR13_34785098.1	s67974.1	5171.07	99
S823		49	13	OAR13_37697946.1	OAR13_39146165.1	1448.22	32
S823		829	13	s29601.1	OAR13_37876562.1	14843.5	275
S823	CON	4	13	OAR13_37697946.1	s26568.1	114.038	3
S823	UNION	4	13	s29601.1	s67974.1	16923.1	318
S824		61	13	OAR13_13641626.1	OAR13_15911703.1	2270.08	44
S824		49	13	OAR13_13577766.1	OAR13_17982125.1	4404.36	89
S824		152	13	OAR13_12044615.1	OAR13_13987202.1	1942.59	36
S824		829	13	s65179.1	OAR13_13952314.1	3280.8	41
S824	CON	4	13	OAR13_13641626.1	OAR13_13952314.1	310.688	9
S824	UNION	4	13	s65179.1	OAR13_17982125.1	7310.61	120
S827		29	12	OAR12_70366176.1	OAR12_73124271.1	2758.09	61
S827		40	12	OAR12_72598226.1	OAR12_74743841.1	2145.61	46
S827		90	12	OAR12_72461244.1	s65734.1	2576.88	55
S827		152	12	OAR12_71058743.1	s72894.1	1825.25	41
S827	CON	4	12	OAR12_72598226.1	s72894.1	285.766	7
S827	UNION	4	12	OAR12_70366176.1	s65734.1	4671.94	102

S828		152	12	OAR12_71058743.1	s72894.1	1825.25	41
S828		123	12	OAR12_70188998.1	OAR12_72385165.1	2196.17	47
S828		702	12	s22960.1	s60710.1	58755.9	1088
S828		29	12	OAR12_70366176.1	OAR12_73124271.1	2758.09	61
S828	CON	4	12	OAR12_71058743.1	s60710.1	842.053	19
S828	UNION	4	12	s22960.1	OAR12_73124271.1	59979.4	1114
S829		702	12	s22960.1	s60710.1	58755.9	1088
S829		79	12	OAR12_12066172.1	OAR12_16134990.1	4068.82	79
S829		140	12	OAR12_11773176.1	OAR12_13334889.1	1561.71	30
S829		507	12	s13792.1	s57817.1	31188.2	514
S829	CON	4	12	s22960.1	OAR12_13334889.1	189.999	6
S829	UNION	4	12	s13792.1	s60710.1	71900.8	1272
S830		556	11	s59845.1	OAR11_63406239.1	2453.91	35
S830		152	11	OAR11_57189920.1	OAR11_61713996.1	4524.08	72
S830		629	11	s13742.1	OAR11_61687914.1	12951	212
S830		702	11	OAR11_57135966.1	OAR11_63846580.1	6710.61	109
S830	CON	4	11	s59845.1	OAR11_61687914.1	735.581	7
S830	UNION	4	11	s13742.1	OAR11_63846580.1	15109.6	249
S833		629	11	s13742.1	OAR11_61687914.1	12951	212
S833		702	11	OAR11_57135966.1	OAR11_63846580.1	6710.61	109
S833		152	11	OAR11_57189920.1	OAR11_61713996.1	4524.08	72
S833		140	11	s19721.1	s56411.1	2390.11	37
S833	CON	4	11	s19721.1	s56411.1	2390.11	37
S833	UNION	4	11	s13742.1	OAR11_63846580.1	15109.6	249
S834		90	11	s07556.1	OAR11_55421823.1	8158.13	135
S834		702	11	OAR11_45618835.1	OAR11_53479950.1	7861.11	136
S834		627	11	s53130.1	OAR11_47522525.1	5053.14	79
S834		556	11	s45011.1	OAR11_47646066_X.1	5597.73	89
S834	CON	4	11	s07556.1	OAR11_47522525.1	258.834	3
S834	UNION	4	11	s45011.1	OAR11_55421823.1	13373.5	218
S835		79	11	s73325.1	s62452.1	1651.72	35
S835		27	11	s04627.1	s73325.1	5587.46	73
S835		702	11	OAR11_17172323.1	OAR11_33359712.1	16187.4	260
S835		90	11	s08572.1	OAR11_30551868.1	7951.23	122
S835	CON	4	11	s73325.1	s73325.1	0	1
S835	UNION	4	11	OAR11_17172323.1	OAR11_33359712.1	16187.4	260
S836		45	10	OAR10_79145098.1	OAR10_87563877.1	8418.78	181
S836		79	10	OAR10_79145098.1	s67060.1	9854.04	210
S836		629	10	OAR10_81430826_X.1	OAR10_83541915.1	2111.09	43
S836		140	10	s75182.1	OAR10_84250273.1	1485.41	36
S836	CON	4	10	s75182.1	OAR10_83541915.1	777.05	19
S836	UNION	4	10	OAR10_79145098.1	s67060.1	9854.04	210
S842		27	10	OAR10_52097098.1	OAR10_55148103.1	3051.01	65
S842		45	10	OAR10_35722333.1	OAR10_57396545.1	21674.2	405
S842		157	10	OAR10_47747943.1	OAR10_53208154_X.1	5460.21	117
S842		606	10	OAR10_51406054.1	OAR10_52777291.1	1371.24	30
S842	CON	4	10	OAR10_52097098.1	OAR10_52777291.1	680.193	16
S842	UNION	4	10	OAR10_35722333.1	OAR10_57396545.1	21674.2	405

S846		829	10	OAR10_33047233.1	OAR10_51969493.1	18922.3	352
S846		45	10	OAR10_35722333.1	OAR10_57396545.1	21674.2	405
S846		157	10	OAR10_47747943.1	OAR10_53208154_X.1	5460.21	117
S846		606	10	OAR10_51406054.1	OAR10_52777291.1	1371.24	30
S846	CON	4	10	OAR10_51406054.1	OAR10_51969493.1	563.439	13
S846	UNION	4	10	OAR10_33047233.1	OAR10_57396545.1	24349.3	459
S847		702	10	s49567.1	OAR10_48462141.1	48462.1	914
S847		829	10	OAR10_33047233.1	OAR10_51969493.1	18922.3	352
S847		45	10	OAR10_35722333.1	OAR10_57396545.1	21674.2	405
S847		157	10	OAR10_47747943.1	OAR10_53208154_X.1	5460.21	117
S847	CON	4	10	OAR10_47747943.1	OAR10_48462141.1	714.198	15
S847	UNION	4	10	s49567.1	OAR10_57396545.1	57396.5	1095
S854		829	10	OAR10_33047233.1	OAR10_51969493.1	18922.3	352
S854		606	10	s44276.1	s64007.1	1390.84	30
S854		702	10	s49567.1	OAR10_48462141.1	48462.1	914
S854		126	10	OAR10_33047233.1	s27070.1	1834.38	36
S854	CON	4	10	OAR10_33047233.1	s64007.1	1368.31	29
S854	UNION	4	10	s49567.1	OAR10_51969493.1	51969.5	988
S855		702	10	s49567.1	OAR10_48462141.1	48462.1	914
S855		844	10	OAR10_17703888.1	s09622.1	13279.3	268
S855		49	10	OAR10_29907137.1	s34530.1	1490.58	33
S855		801	10	OAR10_30059832.1	s26724.1	2707.32	56
S855	CON	4	10	OAR10_30059832.1	s09622.1	923.343	19
S855	UNION	4	10	s49567.1	OAR10_48462141.1	48462.1	914
S856		702	10	s49567.1	OAR10_48462141.1	48462.1	914
S856		844	10	OAR10_11311566.1	OAR10_15972516.1	4660.95	92
S856		123	10	OAR10_12900328.1	OAR10_15258330.1	2358	47
S856		40	10	s10371.1	s16574.1	1926.17	41
S856	CON	4	10	s10371.1	OAR10_15258330.1	1670.43	36
S856	UNION	4	10	s49567.1	OAR10_48462141.1	48462.1	914
S870		126	10	OAR10_10194483.1	OAR10_12381159_X.1	2186.68	41
S870		61	10	OAR10_9852956.1	s23854.1	2137.39	41
S870		702	10	s49567.1	OAR10_48462141.1	48462.1	914
S870		844	10	OAR10_11311566.1	OAR10_15972516.1	4660.95	92
S870	CON	4	10	OAR10_11311566.1	s23854.1	678.78	15
S870	UNION	4	10	s49567.1	OAR10_48462141.1	48462.1	914
S871		606	10	s62543.1	OAR10_9807313.1	1736.97	33
S871		126	10	s49567.1	OAR10_9585529.1	9585.53	173
S871		702	10	s49567.1	OAR10_48462141.1	48462.1	914
S871		29	10	s62543.1	s72120.1	1935.26	37
S871	CON	4	10	s62543.1	OAR10_9585529.1	1515.19	28
S871	UNION	4	10	s49567.1	OAR10_48462141.1	48462.1	914
S872		616	10	OAR10_1660541.1	OAR10_5599088.1	3938.55	71
S872		126	10	s49567.1	OAR10_9585529.1	9585.53	173
S872		629	10	OAR10_5271954.1	OAR10_7507728.1	2235.77	34
S872		702	10	s49567.1	OAR10_48462141.1	48462.1	914
S872	CON	4	10	OAR10_5271954.1	OAR10_5599088.1	327.134	5

S872	UNION	4	10	s49567.1	OAR10_48462141.1	48462.1	914
S876		616	10	OAR10_1660541.1	OAR10_5599088.1	3938.55	71
S876		126	10	s49567.1	OAR10_9585529.1	9585.53	173
S876		507	10	OARUn.1971_37990.1	OAR10_1895343.1	1895.34	48
S876		702	10	s49567.1	OAR10_48462141.1	48462.1	914
S876	CON	4	10	OAR10_1660541.1	OAR10_1895343.1	234.802	7
S876	UNION	4	10	OARUn.1971_37990.1	OAR10_48462141.1	48462.1	919
S877		152	9	s72980.1	s29382.1	1444.6	30
S877		27	9	s28418.1	s60249.1	1605.72	34
S877		616	9	OAR9_85044633.1	s64103.1	2852.17	62
S877		702	9	OAR9_38323108.1	OAR9_96544082.1	58221	1087
S877	CON	4	9	s72980.1	s60249.1	297.948	7
S877	UNION	4	9	OAR9_38323108.1	OAR9_96544082.1	58221	1087
S881		152	9	OAR9_77508470.1	s62001.1	3135.15	63
S881		507	9	OAR9_72979014.1	OAR9_79627675.1	6648.66	130
S881		702	9	OAR9_38323108.1	OAR9_96544082.1	58221	1087
S881		29	9	OAR9_75929101.1	OAR9_77728428.1	1799.33	33
S881	CON	4	9	OAR9_77508470.1	OAR9_77728428.1	219.958	4
S881	UNION	4	9	OAR9_38323108.1	OAR9_96544082.1	58221	1087
S885		507	9	OAR9_72979014.1	OAR9_79627675.1	6648.66	130
S885		27	9	OAR9_71313922.1	OAR9_75600035_X.1	4286.11	88
S885		29	9	OAR9_69054720.1	OAR9_73056873.1	4002.15	80
S885		702	9	OAR9_38323108.1	OAR9_96544082.1	58221	1087
S885	CON	4	9	OAR9_72979014.1	OAR9_73056873.1	77.859	3
S885	UNION	4	9	OAR9_38323108.1	OAR9_96544082.1	58221	1087
S886		45	9	s68634.1	s50783.1	21048.3	358
S886		702	9	OAR9_38323108.1	OAR9_96544082.1	58221	1087
S886		801	9	s24048.1	OAR9_55350197.1	4983.18	48
S886		629	9	OAR9_55140044.1	OAR9_63193973.1	8053.93	166
S886	CON	4	9	OAR9_55140044.1	OAR9_55350197.1	210.153	5
S886	UNION	4	9	s68634.1	OAR9_96544082.1	60605.7	1136
S887		45	9	s68634.1	s50783.1	21048.3	358
S887		702	9	OAR9_38323108.1	OAR9_96544082.1	58221	1087
S887		507	9	OAR9_42661858.1	OAR9_44747972.1	2086.11	49
S887		49	9	OAR9_43772610.1	s14105.1	3346.05	63
S887	CON	4	9	OAR9_43772610.1	OAR9_44747972.1	975.362	22
S887	UNION	4	9	s68634.1	OAR9_96544082.1	60605.7	1136
S892		61	9	OAR9_23382060.1	s56435.1	7434.21	145
S892		45	9	OAR9_26972939.1	OAR9_35203437.1	8230.5	170
S892		152	9	OAR9_28346970.1	OAR9_30909060.1	2562.09	52
S892		27	9	s30783.1	OAR9_32605349.1	3103.89	69
S892	CON	4	9	s30783.1	s56435.1	1314.82	30
S892	UNION	4	9	OAR9_23382060.1	OAR9_35203437.1	11821.4	246
S893		61	9	OAR9_23382060.1	s56435.1	7434.21	145
S893		49	9	OAR9_26114366.1	s24569.1	2421.05	37
S893		45	9	OAR9_26972939.1	OAR9_35203437.1	8230.5	170
S893		152	9	OAR9_28346970.1	OAR9_30909060.1	2562.09	52

S893	CON	4	9	OAR9_28346970.1	s24569.1	188.443	5
S893	UNION	4	9	OAR9_23382060.1	OAR9_35203437.1	11821.4	246
S896		45	9	OAR9_26972939.1	OAR9_35203437.1	8230.5	170
S896		90	9	s19462.1	s01999.1	3928.4	85
S896		61	9	OAR9_23382060.1	s56435.1	7434.21	145
S896		49	9	OAR9_26114366.1	s24569.1	2421.05	37
S896	CON	4	9	OAR9_26972939.1	s01999.1	116.369	2
S896	UNION	4	9	s19462.1	OAR9_35203437.1	12042.5	253
S897		49	9	OAR9_26114366.1	s24569.1	2421.05	37
S897		507	9	s75518.1	OAR9_26587263.1	2962.39	66
S897		61	9	OAR9_23382060.1	s56435.1	7434.21	145
S897		90	9	s19462.1	s01999.1	3928.4	85
S897	CON	4	9	OAR9_26114366.1	OAR9_26587263.1	472.897	8
S897	UNION	4	9	s19462.1	s56435.1	7655.36	152
S898		152	9	OAR9_8876882.1	s21274.1	4522.12	76
S898		79	9	OAR9_8757504.1	OAR9_10494263.1	1736.76	34
S898		123	9	OAR9_8757504.1	OAR9_10494263.1	1736.76	34
S898		844	9	OAR9_5773857.1	s10011.1	10109	172
S898	CON	4	9	OAR9_8876882.1	OAR9_10494263.1	1617.38	33
S898	UNION	4	9	OAR9_5773857.1	s10011.1	10109	172
S901		829	8	OAR8_82151383.1	OAR8_84067219.1	1915.84	39
S901		79	8	OAR8_81635650.1	OAR8_87540028.1	5904.38	127
S901		49	8	OAR8_77578890.1	s01826.1	8664.11	175
S901		844	8	OAR8_82151383.1	OAR8_84032183.1	1880.8	38
S901	CON	4	8	OAR8_82151383.1	OAR8_84032183.1	1880.8	38
S901	UNION	4	8	OAR8_77578890.1	OAR8_87540028.1	9961.14	204
S902		27	8	OAR8_73702430.1	s22687.1	2779.43	58
S902		140	8	OAR8_73355352.1	s22687.1	3126.51	64
S902		152	8	OAR8_72707636.1	OAR8_74991269.1	2283.63	44
S902		507	8	s24061.1	OAR8_74194992.1	1639.28	32
S902	CON	4	8	OAR8_73702430.1	OAR8_74194992.1	492.562	9
S902	UNION	4	8	s24061.1	s22687.1	3926.15	81
S903		61	8	OAR8_69411239.1	OAR8_73226726.1	3815.49	73
S903		45	8	OAR8_68565079.1	OAR8_70323257.1	1758.18	37
S903		829	8	s72483.1	OAR8_69681597.1	2268.01	50
S903		126	8	OAR8_69411239.1	OAR8_72238134.1	2826.89	57
S903	CON	4	8	OAR8_69411239.1	OAR8_69681597.1	270.358	7
S903	UNION	4	8	s72483.1	OAR8_73226726.1	5813.14	116
S906		627	8	OAR8_62742101.1	OAR8_65291492.1	2549.39	49
S906		629	8	OAR8_62742101.1	OAR8_65291492.1	2549.39	49
S906		126	8	OAR8_62481318.1	OAR8_64247515.1	1766.2	33
S906		45	8	OAR8_62748427.1	OAR8_67327419.1	4578.99	91
S906	CON	4	8	OAR8_62748427.1	OAR8_64247515.1	1499.09	27
S906	UNION	4	8	OAR8_62481318.1	OAR8_67327419.1	4846.1	97
S907		49	8	OAR8_45873570.1	OAR8_48463824.1	2590.25	47
S907		140	8	s61158.1	OAR8_52791160.1	9209.77	166
S907		126	8	OAR8_42338337.1	OAR8_45925190.1	3586.85	72

S907		829	8	s66809.1	OAR8_49120824.1	9189.29	175
S907	CON	4	8	OAR8_45873570.1	OAR8_45925190.1	51.62	2
S907	UNION	4	8	s66809.1	OAR8_52791160.1	12859.6	240
S910		27	8	OAR8_27562398.1	s58158.1	1805.47	35
S910		140	8	OAR8_26330799.1	OAR8_31515043.1	5184.24	103
S910		702	8	OAR8_26793121.1	OAR8_38710483.1	11917.4	231
S910		126	8	s69453.1	s50983.1	4232.26	88
S910	CON	4	8	s69453.1	s58158.1	1197.75	24
S910	UNION	4	8	OAR8_26330799.1	OAR8_38710483.1	12379.7	239
S911		27	8	OAR8_27562398.1	s58158.1	1805.47	35
S911		140	8	OAR8_26330799.1	OAR8_31515043.1	5184.24	103
S911		45	8	OAR8_23402353.1	OAR8_27562398.1	4160.05	77
S911		702	8	OAR8_26793121.1	OAR8_38710483.1	11917.4	231
S911	CON	4	8	OAR8_27562398.1	OAR8_27562398.1	0	1
S911	UNION	4	8	OAR8_23402353.1	OAR8_38710483.1	15308.1	293
S916		40	8	OAR8_19634473.1	OAR8_21223050.1	1588.58	30
S916		45	8	OAR8_17854216.1	s00683.1	2986.56	63
S916		27	8	OAR8_16197437.1	OAR8_22199299_X.1	6001.86	125
S916		152	8	OAR8_19076673.1	OAR8_21072066.1	1995.39	36
S916	CON	4	8	OAR8_19634473.1	s00683.1	1206.3	22
S916	UNION	4	8	OAR8_16197437.1	OAR8_22199299_X.1	6001.86	125
S917		829	8	OAR8_3622334.1	s46148.1	3360.96	72
S917		79	8	DU438038_308.1	OAR8_12613673.1	9152.14	171
S917		157	8	DU438038_308.1	s74313.1	3885.06	82
S917		507	8	OAR8_5038936.1	OAR8_6495392.1	1456.46	31
S917	CON	4	8	OAR8_5038936.1	OAR8_6495392.1	1456.46	31
S917	UNION	4	8	DU438038_308.1	OAR8_12613673.1	9152.14	171
S918		616	7	OAR7_33605191.1	OAR7_35261620.1	1656.43	32
S918		507	7	OAR7_32708552.1	OAR7_34234525.1	1525.97	30
S918		45	7	OAR7_31172405.1	OAR7_58958307.1	27785.9	542
S918		702	7	OAR7_31685912.1	OAR7_72973471.1	41287.6	769
S918	CON	4	7	OAR7_33605191.1	OAR7_34234525.1	629.334	11
S918	UNION	4	7	OAR7_31172405.1	OAR7_72973471.1	41801.1	782
S919		126	7	OAR7_31134779.1	OAR7_32846682.1	1711.9	37
S919		45	7	OAR7_31172405.1	OAR7_58958307.1	27785.9	542
S919		702	7	OAR7_31685912.1	OAR7_72973471.1	41287.6	769
S919		507	7	OAR7_32708552.1	OAR7_34234525.1	1525.97	30
S919	CON	4	7	OAR7_32708552.1	OAR7_32846682.1	138.13	3
S919	UNION	4	7	OAR7_31134779.1	OAR7_72973471.1	41838.7	783
S925		45	6	OAR6_96497355.1	OAR6_107118527.1	10621.2	208
S925		844	6	OAR6_101563461.1	OAR6_104016857_X.1	2453.4	49
S925		507	6	OAR6_103085812.1	OAR6_104904339.1	1818.53	32
S925		123	6	s52502.1	OAR6_105661466.1	2254.54	35
S925	CON	4	6	s52502.1	OAR6_104016857_X.1	609.929	6
S925	UNION	4	6	OAR6_96497355.1	OAR6_107118527.1	10621.2	208
S930		140	6	s69231.1	OAR6_79639233.1	3393.23	59
S930		507	6	OAR6_74631720.1	OAR6_76321832.1	1690.11	34

S930		556	6	OAR6_74224903.1	OAR6_76377079.1	2152.18	45
S930		627	6	OAR6_66400799.1	OAR6_90538374.1	24137.6	431
S930	CON	4	6	s69231.1	OAR6_76321832.1	75.832	3
S930	UNION	4	6	OAR6_66400799.1	OAR6_90538374.1	24137.6	431
S931		844	6	OAR6_66669683.1	OAR6_69415746.1	2746.06	46
S931		627	6	OAR6_66400799.1	OAR6_90538374.1	24137.6	431
S931		90	6	OAR6_67781585.1	s33521.1	4904.72	95
S931		702	6	OAR6_68249537.1	OAR6_70866677.1	2617.14	47
S931	CON	4	6	OAR6_68249537.1	OAR6_69415746.1	1166.21	17
S931	UNION	4	6	OAR6_66400799.1	OAR6_90538374.1	24137.6	431
S932		123	6	OAR6_62227590.1	OAR6_64593905.1	2366.32	47
S932		40	6	OAR6_62471770.1	OAR6_65237136.1	2765.37	57
S932		29	6	OAR6_63553726_X.1	OAR6_65237136.1	1683.41	37
S932		606	6	OAR6_64284257.1	OAR6_65763661.1	1479.4	31
S932	CON	4	6	OAR6_64284257.1	OAR6_64593905.1	309.648	10
S932	UNION	4	6	OAR6_62227590.1	OAR6_65763661.1	3536.07	68
S933		629	6	OAR6_58103750.1	OAR6_60012668.1	1908.92	31
S933		61	6	OAR6_57881110.1	OAR6_62074943.1	4193.83	74
S933		29	6	OAR6_55784046.1	OAR6_58211072.1	2427.03	48
S933		79	6	OAR6_51660421.1	s21191.1	10134.5	185
S933	CON	4	6	OAR6_58103750.1	OAR6_58211072.1	107.322	3
S933	UNION	4	6	OAR6_51660421.1	OAR6_62074943.1	10414.5	191
S935		61	6	OAR6_36486409_X.1	s43499.1	3721.22	57
S935		152	6	OAR6_35293995.1	OAR6_38284795.1	2990.8	55
S935		702	6	OAR6_6278702.1	OAR6_45273992.1	38995.3	741
S935		140	6	s73122.1	OAR6_49463357.1	26607.7	513
S935	CON	4	6	OAR6_36486409_X.1	OAR6_38284795.1	1798.38	31
S935	UNION	4	6	OAR6_6278702.1	OAR6_49463357.1	43184.7	833
S941		702	6	OAR6_6278702.1	OAR6_45273992.1	38995.3	741
S941		140	6	s73122.1	OAR6_49463357.1	26607.7	513
S941		123	6	s65803.1	OAR6_35548649.1	7909.9	153
S941		152	6	OAR6_35293995.1	OAR6_38284795.1	2990.8	55
S941	CON	4	6	OAR6_35293995.1	OAR6_35548649.1	254.654	6
S941	UNION	4	6	OAR6_6278702.1	OAR6_49463357.1	43184.7	833
S942		79	6	s40472.1	s30534.1	2475.33	38
S942		507	6	OAR6_4458962.1	OAR6_6482986_X.1	2024.03	38
S942		152	6	OAR6_4458962.1	OAR6_6157042.1	1698.08	30
S942		90	6	OAR6_4458962.1	s44871.1	4179.72	70
S942	CON	4	6	s40472.1	OAR6_6157042.1	125.211	2
S942	UNION	4	6	OAR6_4458962.1	s44871.1	4179.72	70
S943		152	5	OAR5_92367367_X.1	OAR5_94123834.1	1756.47	38
S943		606	5	OAR5_92205253.1	OAR5_94598485.1	2393.23	53
S943		702	5	OAR5_77977258.1	OAR5_100127181.1	22149.9	428
S943		627	5	s09048.1	OAR5_100090515.1	6457.02	134
S943	CON	4	5	s09048.1	OAR5_94123834.1	490.339	11
S943	UNION	4	5	OAR5_77977258.1	OAR5_100127181.1	22149.9	428
S944		152	5	OAR5_92367367_X.1	OAR5_94123834.1	1756.47	38

S944		606	5	OAR5_92205253.1	OAR5_94598485.1	2393.23	53
S944		140	5	DU246484_173.1	OAR5_93172312.1	2645.81	43
S944		702	5	OAR5_77977258.1	OAR5_100127181.1	22149.9	428
S944	CON	4	5	OAR5_92367367_X.1	OAR5_93172312.1	804.944	18
S944	UNION	4	5	OAR5_77977258.1	OAR5_100127181.1	22149.9	428
S967		702	5	OAR5_77977258.1	OAR5_100127181.1	22149.9	428
S967		40	5	s51628.1	OAR5_91610742.1	2115.86	31
S967		123	5	OAR5_89563589.1	OAR5_91733625.1	2170.04	31
S967		140	5	DU246484_173.1	OAR5_93172312.1	2645.81	43
S967	CON	4	5	DU246484_173.1	OAR5_91610742.1	1084.24	11
S967	UNION	4	5	OAR5_77977258.1	OAR5_100127181.1	22149.9	428
S968		702	5	OAR5_77977258.1	OAR5_100127181.1	22149.9	428
S968		27	5	OAR5_73707380.1	OAR5_78020462.1	4313.08	92
S968		606	5	OAR5_70256606.1	OAR5_82901477.1	12644.9	245
S968		629	5	OAR5_76098058.1	OAR5_81196810.1	5098.75	99
S968	CON	4	5	OAR5_77977258.1	OAR5_78020462.1	43.204	2
S968	UNION	4	5	OAR5_70256606.1	OAR5_100127181.1	29870.6	580
S972		627	5	OAR5_69570106.1	OAR5_71156734.1	1586.63	30
S972		126	5	s52618.1	OAR5_72565538.1	7244.72	139
S972		629	5	OAR5_57983508.1	OAR5_71824766.1	13841.3	230
S972		606	5	OAR5_70256606.1	OAR5_82901477.1	12644.9	245
S972	CON	4	5	OAR5_70256606.1	OAR5_71156734.1	900.128	19
S972	UNION	4	5	OAR5_57983508.1	OAR5_82901477.1	24918	441
S973		507	5	OAR5_49532219.1	s07760.1	3268.6	64
S973		152	5	s12940.1	OAR5_61924626.1	14110.5	253
S973		801	5	OAR5_47774570.1	s54250.1	7553.49	151
S973		157	5	s37412.1	s21988.1	1297.99	34
S973	CON	4	5	OAR5_49532219.1	s21988.1	240.912	7
S973	UNION	4	5	OAR5_47774570.1	OAR5_61924626.1	14150.1	254
S974		152	5	s23065.1	OAR5_47736089.1	2786.7	45
S974		627	5	s71007.1	s04774.1	5048.15	85
S974		629	5	s71007.1	s04774.1	5048.15	85
S974		45	5	s71007.1	s06750.1	4481.91	70
S974	CON	4	5	s23065.1	OAR5_47736089.1	2786.7	45
S974	UNION	4	5	s71007.1	s04774.1	5048.15	85
S975		616	5	OAR5_27410982.1	OAR5_29375239.1	1964.26	41
S975		140	5	s53281.1	OAR5_27751113.1	1632.65	36
S975		507	5	s16895.1	s49973.1	1857.27	42
S975		27	5	s51103.1	OAR5_28164690.1	1648.86	34
S975	CON	4	5	OAR5_27410982.1	OAR5_27751113.1	340.131	7
S975	UNION	4	5	s16895.1	OAR5_29375239.1	3412.13	74
S976		629	5	OAR5_17235395.1	s17924.1	1949.73	32
S976		801	5	s44978.1	OAR5_17455332.1	1644.58	33
S976		61	5	OAR5_12770106.1	OAR5_22276454.1	9506.35	152
S976		45	5	OAR5_10884558.1	s06935.1	11513.7	188
S976	CON	4	5	OAR5_17235395.1	OAR5_17455332.1	219.937	2
S976	UNION	4	5	OAR5_10884558.1	s06935.1	11513.7	188

S984		45	5	OAR5_10884558.1	s06935.1	11513.7	188
S984		40	5	s57898.1	s26328.1	2181.44	31
S984		61	5	OAR5_12770106.1	OAR5_22276454.1	9506.35	152
S984		29	5	s08877.1	s18219.1	2077.76	31
S984	CON	4	5	s08877.1	s26328.1	1535.21	21
S984	UNION	4	5	OAR5_10884558.1	s06935.1	11513.7	188
S985		90	4	s49126.1	OAR4_121393593.1	2251.08	32
S985		844	4	s49126.1	OAR4_121393593.1	2251.08	32
S985		507	4	OAR4_117802819.1	OAR4_121393593.1	3590.77	62
S985		616	4	s09629.1	s64459.1	4989.46	82
S985	CON	4	4	s09629.1	OAR4_121393593.1	2237.47	31
S985	UNION	4	4	OAR4_117802819.1	s64459.1	6342.77	113
S992		126	4	s31432.1	OAR4_116953207.1	2171.45	50
S992		27	4	s16058.1	OAR4_115829682.1	8547.19	152
S992		844	4	OAR4_115675953.1	s65922.1	1382.7	35
S992		507	4	OAR4_111473821.1	OAR4_115829682.1	4355.86	71
S992	CON	4	4	OAR4_115675953.1	OAR4_115829682.1	153.729	3
S992	UNION	4	4	s16058.1	s65922.1	9776.17	184
S995		627	4	OAR4_83576387.1	OAR4_89216253.1	5639.87	112
S995		507	4	OAR4_76898220.1	OAR4_83911244.1	7013.02	135
S995		702	4	s58367.1	OAR4_85483475.1	25992.5	514
S995		61	4	OAR4_83231101.1	s24964.1	1857.88	38
S995	CON	4	4	OAR4_83576387.1	OAR4_83911244.1	334.857	6
S995	UNION	4	4	s58367.1	OAR4_89216253.1	29725.3	589
S996		606	4	s58039.1	s49979.1	1493.77	36
S996		507	4	OAR4_76898220.1	OAR4_83911244.1	7013.02	135
S996		702	4	s58367.1	OAR4_85483475.1	25992.5	514
S996		627	4	s56367.1	OAR4_80229268.1	3866.18	75
S996	CON	4	4	s58039.1	OAR4_80229268.1	429.749	9
S996	UNION	4	4	s58367.1	OAR4_85483475.1	25992.5	514
S997		702	4	s58367.1	OAR4_85483475.1	25992.5	514
S997		627	4	OAR4_70656759_X.1	s13803.1	5088.69	101
S997		616	4	OAR4_72367529.1	OAR4_78991289.1	6623.76	132
S997		29	4	OAR4_74705318.1	OAR4_78216552.1	3511.23	72
S997	CON	4	4	OAR4_74705318.1	s13803.1	1040.13	22
S997	UNION	4	4	s58367.1	OAR4_85483475.1	25992.5	514
S1000		702	4	s58367.1	OAR4_85483475.1	25992.5	514
S1000		616	4	OAR4_63468173.1	s75934.1	1686.15	32
S1000		152	4	OAR4_63706459.1	OAR4_69891218.1	6184.76	115
S1000		606	4	OAR4_64327263.1	OAR4_67621451.1	3294.19	61
S1000	CON	4	4	OAR4_64327263.1	s75934.1	827.056	15
S1000	UNION	4	4	s58367.1	OAR4_85483475.1	25992.5	514
S1001		606	4	OAR4_15211722_X.1	OAR4_24841202.1	9629.48	193
S1001		702	4	OARUn.284_293028.1	OAR4_24251309.1	24251.3	470
S1001		126	4	s29747.1	OAR4_16701883.1	4730.1	103
S1001		29	4	s70520.1	OAR4_21300620.1	4951.68	88
S1001	CON	4	4	s70520.1	OAR4_16701883.1	352.943	9
S1001	UNION	4	4	OARUn.284_293028.1	OAR4_24841202.1	24841.2	483

S1008		29	3	OAR3_210514349.1	s62716.1	4058.72	71
S1008		606	3	OAR3_208666709.1	s05793.1	2048.02	40
S1008		507	3	OAR3_179244393.1	OAR3_211362922_X.1	32118.5	604
S1008		616	3	OAR3_209072421.1	OAR3_222052522.1	12980.1	230
S1008	CON	4	3	OAR3_210514349.1	s05793.1	200.379	5
S1008	UNION	4	3	OAR3_179244393.1	OAR3_222052522.1	42808.1	792
S1011		507	3	OAR3_179244393.1	OAR3_211362922_X.1	32118.5	604
S1011		157	3	OAR3_206856083.1	OAR3_209162669.1	2306.59	49
S1011		606	3	OAR3_208666709.1	s05793.1	2048.02	40
S1011		616	3	OAR3_209072421.1	OAR3_222052522.1	12980.1	230
S1011	CON	4	3	OAR3_209072421.1	OAR3_209162669.1	90.248	2
S1011	UNION	4	3	OAR3_179244393.1	OAR3_222052522.1	42808.1	792
S1012		507	3	OAR3_179244393.1	OAR3_211362922_X.1	32118.5	604
S1012		123	3	s09050.1	OAR3_202943170.1	11054.8	191
S1012		702	3	OAR3_196157112.1	OAR3_199132095.1	2974.98	56
S1012		157	3	OAR3_197414907.1	OAR3_199052614.1	1637.71	31
S1012	CON	4	3	OAR3_197414907.1	OAR3_199052614.1	1637.71	31
S1012	UNION	4	3	OAR3_179244393.1	OAR3_211362922_X.1	32118.5	604
S1014		507	3	OAR3_179244393.1	OAR3_211362922_X.1	32118.5	604
S1014		123	3	s09050.1	OAR3_202943170.1	11054.8	191
S1014		140	3	s01490.1	s39041.1	1666.11	31
S1014		702	3	OAR3_196157112.1	OAR3_199132095.1	2974.98	56
S1014	CON	4	3	OAR3_196157112.1	s39041.1	151.885	4
S1014	UNION	4	3	OAR3_179244393.1	OAR3_211362922_X.1	32118.5	604
S1015		123	3	s09050.1	OAR3_202943170.1	11054.8	191
S1015		702	3	OAR3_190128314.1	OAR3_194730311.1	4602	79
S1015		507	3	OAR3_179244393.1	OAR3_211362922_X.1	32118.5	604
S1015		140	3	s01490.1	s39041.1	1666.11	31
S1015	CON	4	3	s01490.1	OAR3_194730311.1	87.424	2
S1015	UNION	4	3	OAR3_179244393.1	OAR3_211362922_X.1	32118.5	604
S1016		140	3	OAR3_188369387.1	s01421.1	2146.03	47
S1016		123	3	s55427.1	s32545.1	2793.06	53
S1016		702	3	OAR3_178121896.1	s16918.1	11927	230
S1016		507	3	OAR3_179244393.1	OAR3_211362922_X.1	32118.5	604
S1016	CON	4	3	OAR3_188369387.1	s32545.1	338.408	10
S1016	UNION	4	3	OAR3_178121896.1	OAR3_211362922_X.1	33241	624
S1017		627	3	OAR3_155236610.1	s75549.1	19415.4	375
S1017		629	3	OAR3_155236610.1	s75549.1	19415.4	375
S1017		126	3	OAR3_164029380.1	s12237.1	3624.48	67
S1017		61	3	OAR3_164422954.1	s12237.1	3230.91	60
S1017	CON	4	3	OAR3_164422954.1	s12237.1	3230.91	60
S1017	UNION	4	3	OAR3_155236610.1	s75549.1	19415.4	375
S1018		702	3	OAR3_151694139.1	s22496.1	2159.1	45
S1018		507	3	OAR3_151463131.1	s47510.1	2199.78	44
S1018		61	3	OAR3_149613458.1	OAR3_152083446.1	2469.99	51
S1018		90	3	OAR3_149080267.1	OAR3_151694139.1	2613.87	57
S1018	CON	4	3	OAR3_151694139.1	OAR3_151694139.1	0	1

S1018	UNION	4	3	OAR3_149080267.1	s22496.1	4772.97	101
S1019		140	3	OAR3_118738718.1	OAR3_121938058.1	3199.34	65
S1019		123	3	OAR3_118383093.1	OAR3_120348297.1	1965.2	39
S1019		152	3	OAR3_115651452.1	s09346.1	3379.64	69
S1019		629	3	OAR3_112096089.1	OAR3_121762751.1	9666.66	172
S1019	CON	4	3	OAR3_118738718.1	s09346.1	292.373	6
S1019	UNION	4	3	OAR3_112096089.1	OAR3_121938058.1	9841.97	175
S1022		123	3	OAR3_118383093.1	OAR3_120348297.1	1965.2	39
S1022		152	3	OAR3_115651452.1	s09346.1	3379.64	69
S1022		616	3	OAR3_115651452.1	OAR3_118690316.1	3038.86	63
S1022		629	3	OAR3_112096089.1	OAR3_121762751.1	9666.66	172
S1022	CON	4	3	OAR3_118383093.1	OAR3_118690316.1	307.223	6
S1022	UNION	4	3	OAR3_112096089.1	OAR3_121762751.1	9666.66	172
S1024		123	3	s26313.1	OAR3_86263758.1	2301.95	49
S1024		45	3	s30085.1	OAR3_88649034.1	3880.06	72
S1024		829	3	OAR3_84830738_X.1	OAR3_87055108.1	2224.37	45
S1024		140	3	OAR3_84830738_X.1	s18949.1	13571.1	269
S1024	CON	4	3	OAR3_84830738_X.1	OAR3_86263758.1	1433.02	28
S1024	UNION	4	3	s26313.1	s18949.1	14440.1	290
S1035		29	3	OAR3_68985625.1	OAR3_71509142.1	2523.52	46
S1035		829	3	OAR3_67058161.1	OAR3_74604120.1	7545.96	148
S1035		45	3	OAR3_70204674.1	s57378.1	2739.58	51
S1035		157	3	OAR3_70578986.1	OAR3_76536013.1	5957.03	117
S1035	CON	4	3	OAR3_70578986.1	OAR3_71509142.1	930.156	17
S1035	UNION	4	3	OAR3_67058161.1	OAR3_76536013.1	9477.85	187
S1039		29	3	OAR3_68985625.1	OAR3_71509142.1	2523.52	46
S1039		829	3	OAR3_67058161.1	OAR3_74604120.1	7545.96	148
S1039		45	3	OAR3_70204674.1	s57378.1	2739.58	51
S1039		123	3	OAR3_68391345.1	OAR3_70204674.1	1813.33	34
S1039	CON	4	3	OAR3_70204674.1	OAR3_70204674.1	0	1
S1039	UNION	4	3	OAR3_67058161.1	OAR3_74604120.1	7545.96	148
S1040		702	3	s50340.1	s35506.1	2560.33	53
S1040		556	3	s12121.1	OAR3_62555400.1	2127.51	34
S1040		45	3	s45049.1	OAR3_66317995.1	4167.71	82
S1040		157	3	OAR3_59779674.1	s51327.1	4543.97	78
S1040	CON	4	3	s50340.1	OAR3_62555400.1	341.922	6
S1040	UNION	4	3	OAR3_59779674.1	OAR3_66317995.1	6538.32	118
S1041		627	3	OAR3_35868525.1	s49084.1	3139.94	61
S1041		629	3	OAR3_35868525.1	s49084.1	3139.94	61
S1041		616	3	OAR3_35747405.1	OAR3_37420977.1	1673.57	33
S1041		702	3	DU259120_464.1	OAR3_59494261.1	59494.3	1111
S1041	CON	4	3	OAR3_35868525.1	OAR3_37420977.1	1552.45	30
S1041	UNION	4	3	DU259120_464.1	OAR3_59494261.1	59494.3	1111
S1042		606	3	s11352.1	OAR3_30766484.1	1487.15	32
S1042		27	3	OAR3_28398665.1	OAR3_32271305.1	3872.64	80
S1042		702	3	DU259120_464.1	OAR3_59494261.1	59494.3	1111
S1042		556	3	OAR3_29169436.1	OAR3_31172991.1	2003.56	43

S1042	CON	4	3	s11352.1	OAR3_30766484.1	1487.15	32
S1042	UNION	4	3	DU259120_464.1	OAR3_59494261.1	59494.3	1111
S1043		27	3	OAR3_28398665.1	OAR3_32271305.1	3872.64	80
S1043		61	3	OAR3_27099890.1	s60002.1	1720.22	39
S1043		507	3	s49768.1	OAR3_28567790.1	9061.66	193
S1043		702	3	DU259120_464.1	OAR3_59494261.1	59494.3	1111
S1043	CON	4	3	OAR3_28398665.1	OAR3_28567790.1	169.125	4
S1043	UNION	4	3	DU259120_464.1	OAR3_59494261.1	59494.3	1111
S1045		702	3	DU259120_464.1	OAR3_59494261.1	59494.3	1111
S1045		507	3	s49768.1	OAR3_28567790.1	9061.66	193
S1045		152	3	OAR3_24047322.1	OAR3_27322763.1	3275.44	68
S1045		61	3	OAR3_27099890.1	s60002.1	1720.22	39
S1045	CON	4	3	OAR3_27099890.1	OAR3_27322763.1	222.873	5
S1045	UNION	4	3	DU259120_464.1	OAR3_59494261.1	59494.3	1111
S1048		507	3	s62006.1	s69257.1	1928.36	38
S1048		629	3	s03864.1	s69257.1	2609.38	55
S1048		27	3	OAR3_2505765.1	s46775.1	3060.4	58
S1048		702	3	DU259120_464.1	OAR3_59494261.1	59494.3	1111
S1048	CON	4	3	s62006.1	s46775.1	1180.8	22
S1048	UNION	4	3	DU259120_464.1	OAR3_59494261.1	59494.3	1111
S1053		844	2	OAR2_261042542.1	s29579.1	2065.98	50
S1053		507	2	s28022.1	s74961.1	1873.61	46
S1053		79	2	s71223.1	s55376.1	1830.55	40
S1053		702	2	OAR2_252278273.1	s29579.1	10830.2	215
S1053	CON	4	2	OAR2_261042542.1	s55376.1	380.358	9
S1053	UNION	4	2	OAR2_252278273.1	s29579.1	10830.2	215
S1058		627	2	OAR2_257231205.1	OAR2_260008602.1	2777.4	52
S1058		629	2	OAR2_257231205.1	OAR2_260008602.1	2777.4	52
S1058		123	2	OAR2_255186614.1	s38715.1	2347.14	47
S1058		702	2	OAR2_252278273.1	s29579.1	10830.2	215
S1058	CON	4	2	OAR2_257231205.1	s38715.1	302.554	7
S1058	UNION	4	2	OAR2_252278273.1	s29579.1	10830.2	215
S1059		45	2	s55941.1	s46164.1	2104	34
S1059		507	2	OAR2_224687649.1	s08704.1	6568.89	90
S1059		616	2	OAR2_229781196.1	s00795.1	3081.64	50
S1059		40	2	OAR2_227627755.1	s54339.1	2648.12	33
S1059	CON	4	2	OAR2_229781196.1	s54339.1	494.676	8
S1059	UNION	4	2	OAR2_224687649.1	s00795.1	8175.18	117
S1063		29	2	OAR2_223862860_X.1	OAR2_226636096.1	2773.24	53
S1063		140	2	OAR2_221746917.1	OAR2_225636672.1	3889.76	77
S1063		702	2	OAR2_212387600.1	OAR2_223862860_X.1	11475.3	237
S1063		79	2	OAR2_222545307.1	OAR2_224219290.1	1673.98	33
S1063	CON	4	2	OAR2_223862860_X.1	OAR2_223862860_X.1	0	1
S1063	UNION	4	2	OAR2_212387600.1	OAR2_226636096.1	14248.5	289
S1067		556	2	OAR2_194667510.1	OAR2_196843824.1	2176.31	36
S1067		627	2	OAR2_192100658.1	OAR2_194917758.1	2817.1	55
S1067		140	2	OAR2_191308232.1	OAR2_203356427.1	12048.2	217

S1067		79	2	OAR2_181847398.1	s12968.1	13971.1	260
S1067	CON	4	2	OAR2_194667510.1	OAR2_194917758.1	250.248	6
S1067	UNION	4	2	OAR2_181847398.1	OAR2_203356427.1	21509	395
S1068		507	2	OAR2_193075643.1	OAR2_194594406.1	1518.76	30
S1068		627	2	OAR2_192100658.1	OAR2_194917758.1	2817.1	55
S1068		79	2	OAR2_181847398.1	s12968.1	13971.1	260
S1068		140	2	OAR2_191308232.1	OAR2_203356427.1	12048.2	217
S1068	CON	4	2	OAR2_193075643.1	OAR2_194594406.1	1518.76	30
S1068	UNION	4	2	OAR2_181847398.1	OAR2_203356427.1	21509	395
S1073		140	2	OAR2_187991993.1	OAR2_190420269.1	2428.28	49
S1073		152	2	s61174.1	s26570.1	7267.15	138
S1073		90	2	OAR2_181512709.1	OAR2_190616592.1	9103.88	175
S1073		79	2	OAR2_181847398.1	s12968.1	13971.1	260
S1073	CON	4	2	OAR2_187991993.1	s26570.1	347.543	6
S1073	UNION	4	2	s61174.1	s12968.1	14746.1	278
S1076		627	2	OAR2_155425432.1	OAR2_160296103.1	4870.67	100
S1076		606	2	s01065.1	s72813.1	6497.5	138
S1076		702	2	s54108.1	OAR2_162167662.1	23623.2	482
S1076		45	2	OAR2_154645235.1	OAR2_156250273.1	1605.04	39
S1076	CON	4	2	OAR2_155425432.1	OAR2_156250273.1	824.841	18
S1076	UNION	4	2	s54108.1	OAR2_162167662.1	23623.2	482
S1077		123	2	OAR2_93160890.1	s59269.1	3203.27	47
S1077		49	2	OAR2_90239503.1	OAR2_96436987_X.1	6197.48	106
S1077		606	2	s14873.1	OAR2_96066817.1	6724.02	118
S1077		79	2	OAR2_93935779.1	OAR2_96537414.1	2601.64	32
S1077	CON	4	2	OAR2_93935779.1	OAR2_96066817.1	2131.04	26
S1077	UNION	4	2	s14873.1	OAR2_96537414.1	7194.62	124
S1078		49	2	OAR2_90239503.1	OAR2_96436987_X.1	6197.48	106
S1078		90	2	OAR2_90239503.1	s66693.1	3509.49	72
S1078		123	2	OAR2_93160890.1	s59269.1	3203.27	47
S1078		606	2	s14873.1	OAR2_96066817.1	6724.02	118
S1078	CON	4	2	OAR2_93160890.1	s66693.1	588.102	15
S1078	UNION	4	2	s14873.1	OAR2_96436987_X.1	7094.19	123
S1079		507	2	s37403.1	s36747.1	4416.51	74
S1079		79	2	OAR2_49013850.1	s36747.1	5342.23	88
S1079		45	2	OAR2_17890079.1	s50931.1	32810.2	566
S1079		29	2	s05611.1	OAR2_55308142.1	7693.97	128
S1079	CON	4	2	s37403.1	s50931.1	760.761	5
S1079	UNION	4	2	OAR2_17890079.1	OAR2_55308142.1	37418.1	649
S1085		27	2	OAR2_21576835.1	s72217.1	1514.78	31
S1085		45	2	OAR2_17890079.1	s50931.1	32810.2	566
S1085		140	2	s43106.1	OAR2_21997773.1	5865.81	99
S1085		702	2	s55237.1	OAR2_24281639.1	13422.7	255
S1085	CON	4	2	OAR2_21576835.1	OAR2_21997773.1	420.938	8
S1085	UNION	4	2	s55237.1	s50931.1	39841.4	703
S1086		140	2	s43106.1	OAR2_21997773.1	5865.81	99
S1086		40	2	OAR2_15830990.1	s33375.1	2008.94	36

S1086		702	2	s55237.1	OAR2_24281639.1	13422.7	255
S1086		606	2	OAR2_8977613.1	OAR2_16538077.1	7560.46	153
S1086	CON	4	2	s43106.1	OAR2_16538077.1	406.112	8
S1086	UNION	4	2	OAR2_8977613.1	OAR2_24281639.1	15304	294
S1089		629	2	OAR2_9828035.1	OAR2_12469561.1	2641.53	51
S1089		627	2	s00957.1	OAR2_12469561.1	3947.37	80
S1089		702	2	s55237.1	OAR2_24281639.1	13422.7	255
S1089		606	2	OAR2_8977613.1	OAR2_16538077.1	7560.46	153
S1089	CON	4	2	s55237.1	OAR2_12469561.1	1610.6	30
S1089	UNION	4	2	s00957.1	OAR2_24281639.1	15759.4	305
S1098		606	1	OAR1_268122939.1	s72986.1	4302.03	61
S1098		844	1	s08603.1	OAR1_268690065.1	1941.63	33
S1098		507	1	OAR1_266243405.1	OAR1_270405323.1	4161.92	75
S1098		801	1	s09883.1	s73870.1	6372.31	95
S1098	CON	4	1	OAR1_268122939.1	OAR1_268690065.1	567.126	11
S1098	UNION	4	1	OAR1_266243405.1	s73870.1	6987.71	106
S1099		556	1	OAR1_256940663.1	OAR1_259109428.1	2168.76	39
S1099		844	1	OAR1_256339018.1	OAR1_260270011.1	3930.99	65
S1099		126	1	OAR1_253349819.1	s70277.1	12807	217
S1099		829	1	OAR1_257557854.1	s49280.1	5797.95	97
S1099	CON	4	1	OAR1_257557854.1	OAR1_259109428.1	1551.57	25
S1099	UNION	4	1	OAR1_253349819.1	s70277.1	12807	217
S1100		507	1	OAR1_237623635.1	OAR1_241663250.1	4039.61	56
S1100		126	1	s40188.1	OAR1_241703558.1	8537.63	145
S1100		79	1	OAR1_235621637.1	OAR1_242295786.1	6674.15	108
S1100		606	1	OAR1_236964370.1	s30975.1	1929.5	38
S1100	CON	4	1	OAR1_237623635.1	s30975.1	1270.23	25
S1100	UNION	4	1	s40188.1	OAR1_242295786.1	9129.86	158
S1101		606	1	OAR1_236964370.1	s30975.1	1929.5	38
S1101		507	1	s33272.1	OAR1_237198444.1	2871.7	58
S1101		126	1	s40188.1	OAR1_241703558.1	8537.63	145
S1101		79	1	OAR1_235621637.1	OAR1_242295786.1	6674.15	108
S1101	CON	4	1	OAR1_236964370.1	OAR1_237198444.1	234.074	4
S1101	UNION	4	1	s40188.1	OAR1_242295786.1	9129.86	158
S1102		79	1	OAR1_235621637.1	OAR1_242295786.1	6674.15	108
S1102		829	1	OAR1_219136030.1	OAR1_236103073.1	16967	322
S1102		507	1	s33272.1	OAR1_237198444.1	2871.7	58
S1102		126	1	s40188.1	OAR1_241703558.1	8537.63	145
S1102	CON	4	1	OAR1_235621637.1	OAR1_236103073.1	481.436	11
S1102	UNION	4	1	OAR1_219136030.1	OAR1_242295786.1	23159.8	419
S1110		507	1	s33272.1	OAR1_237198444.1	2871.7	58
S1110		126	1	s40188.1	OAR1_241703558.1	8537.63	145
S1110		40	1	OAR1_230374318.1	OAR1_234437646.1	4063.33	74
S1110		829	1	OAR1_219136030.1	OAR1_236103073.1	16967	322
S1110	CON	4	1	s33272.1	OAR1_234437646.1	110.907	4
S1110	UNION	4	1	OAR1_219136030.1	OAR1_241703558.1	22567.5	406
S1116		126	1	OAR1_217543314.1	OAR1_219952416.1	2409.1	36

S1116		616	1	OAR1_215364467.1	OAR1_219065429.1	3700.96	59
S1116		702	1	OAR1_201628760.1	OAR1_224818104.1	23189.3	424
S1116		629	1	OAR1_214417389.1	OAR1_233116174.1	18698.8	341
S1116	CON	4	1	OAR1_217543314.1	OAR1_219065429.1	1522.12	19
S1116	UNION	4	1	OAR1_201628760.1	OAR1_233116174.1	31487.4	578
S1117		61	1	OAR1_214424851.1	s12243.1	2026.25	37
S1117		629	1	OAR1_214417389.1	OAR1_233116174.1	18698.8	341
S1117		702	1	OAR1_201628760.1	OAR1_224818104.1	23189.3	424
S1117		616	1	OAR1_215364467.1	OAR1_219065429.1	3700.96	59
S1117	CON	4	1	OAR1_215364467.1	s12243.1	1086.63	18
S1117	UNION	4	1	OAR1_201628760.1	OAR1_233116174.1	31487.4	578
S1124		702	1	OAR1_201628760.1	OAR1_224818104.1	23189.3	424
S1124		45	1	s68692.1	OAR1_208344041_X.1	2497.93	43
S1124		61	1	OAR1_206794718.1	OAR1_210786386.1	3991.67	81
S1124		29	1	OAR1_207403921.1	OAR1_209169552_X.1	1765.63	31
S1124	CON	4	1	OAR1_207403921.1	OAR1_208344041_X.1	940.121	14
S1124	UNION	4	1	OAR1_201628760.1	OAR1_224818104.1	23189.3	424
S1125		61	1	OAR1_206794718.1	OAR1_210786386.1	3991.67	81
S1125		629	1	DU316524_436.1	OAR1_207345817.1	5355.54	90
S1125		702	1	OAR1_201628760.1	OAR1_224818104.1	23189.3	424
S1125		45	1	s68692.1	OAR1_208344041_X.1	2497.93	43
S1125	CON	4	1	OAR1_206794718.1	OAR1_207345817.1	551.099	13
S1125	UNION	4	1	OAR1_201628760.1	OAR1_224818104.1	23189.3	424
S1126		45	1	s68692.1	OAR1_208344041_X.1	2497.93	43
S1126		507	1	s71677.1	OAR1_206274474.1	4131.31	65
S1126		629	1	DU316524_436.1	OAR1_207345817.1	5355.54	90
S1126		702	1	OAR1_201628760.1	OAR1_224818104.1	23189.3	424
S1126	CON	4	1	s68692.1	OAR1_206274474.1	428.364	7
S1126	UNION	4	1	OAR1_201628760.1	OAR1_224818104.1	23189.3	424
S1127		157	1	DU287626_225.1 250	506CS3900283200001_442.1	2218.24	32
S1127		507	1	OAR1_194627962.1	OAR1_201343673.1	6715.71	128
S1127		123	1	s30590.1	OAR1_201878358.1	3641.45	75
S1127		801	1	OAR1_198827329.1	s56333.1	6897.16	121
S1127	CON	4	1	DU287626_225.1	OAR1_201343673.1	272.282	7
S1127	UNION	4	1	OAR1_194627962.1	s56333.1	11096.5	199
S1133		829	1	s65978.1	OAR1_199615737.1	2744.25	51
S1133		507	1	OAR1_194627962.1	OAR1_201343673.1	6715.71	128
S1133		123	1	s30590.1	OAR1_201878358.1	3641.45	75
S1133		801	1	OAR1_198827329.1	s56333.1	6897.16	121
S1133	CON	4	1	OAR1_198827329.1	OAR1_199615737.1	788.408	14
S1133	UNION	4	1	OAR1_194627962.1	s56333.1	11096.5	199
S1136		123	1	s73461.1	s51810.1	2007.14	39
S1136		801	1	OAR1_191797121.1	OAR1_193664187.1	1867.07	37
S1136		40	1	OAR1_190668511.1	OAR1_193623565.1	2955.05	54
S1136		606	1	s12579.1	OAR1_193526104.1	9327.73	173
S1136	CON	4	1	s73461.1	OAR1_193526104.1	1308.63	25
S1136	UNION	4	1	s12579.1	s51810.1	10026.2	187

S1137		702	1	OAR1_7717464.1	OAR1_58938560.1	51221.1	934
S1137		606	1	OAR1_44090814.1	OAR1_51307088.1	7216.27	123
S1137		844	1	OAR1_44241542.1	OAR1_49456350.1	5214.81	94
S1137		123	1	s25087.1	OAR1_52085279.1	3294.22	54
S1137	CON	4	1	s25087.1	OAR1_49456350.1	665.292	14
S1137	UNION	4	1	OAR1_7717464.1	OAR1_58938560.1	51221.1	934
S1138		61	1	OAR1_32040848.1	OAR1_37282541.1	5241.69	99
S1138		79	1	s75178.1	s57223.1	2875.75	56
S1138		702	1	OAR1_7717464.1	OAR1_58938560.1	51221.1	934
S1138		123	1	s67590.1	s45674.1	11170	204
S1138	CON	4	1	OAR1_32040848.1	s57223.1	228.114	6
S1138	UNION	4	1	OAR1_7717464.1	OAR1_58938560.1	51221.1	934
S1139		61	1	OAR1_4303667.1	s49872.1	3986.89	79
S1139		606	1	s65509.1	OAR1_9881817.1	5706.07	114
S1139		152	1	OAR1_2921089.1	s05524.1	1595.06	31
S1139		90	1	OAR1_2921089.1	s05524.1	1595.06	31
S1139	CON	4	1	OAR1_4303667.1	s05524.1	212.486	4
S1139	UNION	4	1	OAR1_2921089.1	OAR1_9881817.1	6960.73	138
S1140		829	26	OAR26_46921474.1	OAR26_50043613.1	3122.14	60
S1140		606	26	OAR26_46747403.1	s46142.1	1645.34	32
S1140		61	26	s63463.1	OAR26_46995252.1	7812.86	140
S1140	CON	3	26	OAR26_46921474.1	OAR26_46995252.1	73.778	3
S1140	UNION	3	26	s63463.1	OAR26_50043613.1	10861.2	197
S1146		29	26	OAR26_26341151.1	OAR26_31513764.1	5172.61	112
S1146		27	26	OAR26_19029894.1	s08067.1	9710.75	157
S1146		61	26	OAR26_25273391.1	s54702.1	1825.22	33
S1146	CON	3	26	OAR26_26341151.1	s54702.1	757.461	18
S1146	UNION	3	26	OAR26_19029894.1	OAR26_31513764.1	12483.9	219
S1147		27	26	OAR26_19029894.1	s08067.1	9710.75	157
S1147		29	26	OAR26_19029894.1	OAR26_25594436.1	6564.54	94
S1147		61	26	OAR26_25273391.1	s54702.1	1825.22	33
S1147	CON	3	26	OAR26_25273391.1	OAR26_25594436.1	321.045	2
S1147	UNION	3	26	OAR26_19029894.1	s08067.1	9710.75	157
S1148		606	26	OAR26_11939982.1	OAR26_14981321.1	3041.34	49
S1148		126	26	OAR26_8625199.1	OAR26_13494685.1	4869.49	83
S1148		90	26	OAR26_10633898.1	OAR26_12884989_X.1	2251.09	33
S1148	CON	3	26	OAR26_11939982.1	OAR26_12884989_X.1	945.008	16
S1148	UNION	3	26	OAR26_8625199.1	OAR26_14981321.1	6356.12	104
S1149		844	26	OAR26_8709366.1	OAR26_10981172.1	2271.81	41
S1149		126	26	OAR26_8625199.1	OAR26_13494685.1	4869.49	83
S1149		90	26	OAR26_10633898.1	OAR26_12884989_X.1	2251.09	33
S1149	CON	3	26	OAR26_10633898.1	OAR26_10981172.1	347.274	4
S1149	UNION	3	26	OAR26_8625199.1	OAR26_13494685.1	4869.49	83
S1150		507	25	s10320.1	s12031.1	20755.8	401
S1150		79	25	s21107.1	s10114.1	2215.18	43
S1150		627	25	s52797.1	s09722.1	2190.12	43
S1150	CON	3	25	s10320.1	s09722.1	389.054	8

S1150	UNION	3	25	s52797.1	s12031.1	22556.9	436
S1153		45	25	s58408.1	s21107.1	1832.56	31
S1153		123	25	OAR25_4272552.1	OAR25_6357231.1	2084.68	37
S1153		49	25	OAR25_5894736.1	OAR25_7752816.1	1858.08	35
S1153	CON	3	25	OAR25_5894736.1	OAR25_6357231.1	462.495	8
S1153	UNION	3	25	OAR25_4272552.1	OAR25_7752816.1	3480.26	64
S1154		123	25	OAR25_4272552.1	OAR25_6357231.1	2084.68	37
S1154		90	25	s12866.1	s21472.1	4810.44	79
S1154		606	25	OAR25_3074074.1	s75010.1	2248.45	30
S1154	CON	3	25	OAR25_4272552.1	s21472.1	537.884	13
S1154	UNION	3	25	s12866.1	OAR25_6357231.1	6357.23	103
S1155		152	24	s59335.1	s03728.1	3687.79	56
S1155		801	24	DU442221_261.1	OAR24_39340682.1	2923.64	44
S1155		79	24	OAR24_37150973.1	s52435.1	2732.57	31
S1155	CON	3	24	OAR24_37150973.1	s03728.1	1912.12	21
S1155	UNION	3	24	s59335.1	s52435.1	4508.24	66
S1174		844	24	OAR24_27265846.1	s16047.1	2492.21	39
S1174		801	24	OAR24_26497561.1	OAR24_30209224.1	3711.66	59
S1174		45	24	OAR24_25044819.1	s31442.1	2930.96	39
S1174	CON	3	24	OAR24_27265846.1	s31442.1	709.929	11
S1174	UNION	3	24	OAR24_25044819.1	OAR24_30209224.1	5164.4	75
S1179		140	24	s62631.1	s39918.1	2861.66	39
S1179		90	24	DU452167_477.1	s12637.1	3545.74	51
S1179		45	24	OAR24_25044819.1	s31442.1	2930.96	39
S1179	CON	3	24	OAR24_25044819.1	s12637.1	959.86	12
S1179	UNION	3	24	DU452167_477.1	s31442.1	5516.83	78
S1183		140	24	s62631.1	s39918.1	2861.66	39
S1183		90	24	DU452167_477.1	s12637.1	3545.74	51
S1183		29	24	OAR24_20737428.1	OAR24_24557331.1	3819.9	58
S1183	CON	3	24	s62631.1	OAR24_24557331.1	1354.18	20
S1183	UNION	3	24	OAR24_20737428.1	s39918.1	5327.38	77
S1184		27	23	s58136.1	OAR23_60124720.1	9675.72	139
S1184		29	23	s58136.1	OAR23_60124720.1	9675.72	139
S1184		606	23	OAR23_47138977.1	s72429.1	3767.44	67
S1184	CON	3	23	s58136.1	s72429.1	457.415	7
S1184	UNION	3	23	OAR23_47138977.1	OAR23_60124720.1	12985.7	199
S1191		29	23	s58159.1	OAR23_31443579.1	5396.2	77
S1191		79	23	OAR23_25154247.1	OAR23_31443579.1	6289.33	95
S1191		61	23	OAR23_25624860.1	s11742.1	2147.4	31
S1191	CON	3	23	s58159.1	s11742.1	1724.89	22
S1191	UNION	3	23	OAR23_25154247.1	OAR23_31443579.1	6289.33	95
S1192		45	23	OAR23_11847614.1	OAR23_14163654.1	2316.04	37
S1192		79	23	s35508.1	OAR23_11959774.1	4010.75	66
S1192		702	23	s07716.1	OAR23_12771349.1	4783.72	80
S1192	CON	3	23	OAR23_11847614.1	OAR23_11959774.1	112.16	2
S1192	UNION	3	23	s35508.1	OAR23_14163654.1	6214.63	101

S1194		45	22	s74001.1	s40749.1	2372.79	52
S1194		616	22	DU292797_377.1	s40749.1	1663.13	36
S1194		157	22	OAR22_51968251.1	s22848.1	1895.85	43
S1194	CON	3	22	OAR22_51968251.1	s40749.1	1636.94	35
S1194	UNION	3	22	s74001.1	s22848.1	2631.7	60
S1195		61	22	OAR22_44931867.1	s72791.1	1996.46	44
S1195		123	22	OAR22_44931867.1	OAR22_46715650.1	1783.78	40
S1195		507	22	s29080.1	OAR22_49444571.1	3407.59	66
S1195	CON	3	22	s29080.1	OAR22_46715650.1	678.668	12
S1195	UNION	3	22	OAR22_44931867.1	OAR22_49444571.1	4512.7	94
S1209		61	22	OAR22_44931867.1	s72791.1	1996.46	44
S1209		123	22	OAR22_44931867.1	OAR22_46715650.1	1783.78	40
S1209		79	22	OAR22_44471234.1	OAR22_45832317.1	1361.08	33
S1209	CON	3	22	OAR22_44931867.1	OAR22_45832317.1	900.45	23
S1209	UNION	3	22	OAR22_44471234.1	s72791.1	2457.09	54
S1212		27	21	s25809.1	s13923.1	11828.3	168
S1212		49	21	s74518.1	s38134.1	13941.9	212
S1212		606	21	OAR21_21865772.1	OAR21_36571389.1	14705.6	232
S1212	CON	3	21	s25809.1	s38134.1	446.814	8
S1212	UNION	3	21	s74518.1	s13923.1	25323.4	372
S1213		801	21	OAR21_17659120.1	s64572.1	2030.63	35
S1213		123	21	OAR21_15046774.1	OAR21_17819384.1	2772.61	42
S1213		79	21	OAR21_16500504.1	OAR21_24084777.1	7584.27	125
S1213	CON	3	21	OAR21_17659120.1	OAR21_17819384.1	160.264	6
S1213	UNION	3	21	OAR21_15046774.1	OAR21_24084777.1	9038	144
S1216		702	21	s74845.1	OAR21_17189778.1	17189.8	241
S1216		123	21	OAR21_15046774.1	OAR21_17819384.1	2772.61	42
S1216		79	21	OAR21_16500504.1	OAR21_24084777.1	7584.27	125
S1216	CON	3	21	OAR21_16500504.1	OAR21_17189778.1	689.274	10
S1216	UNION	3	21	s74845.1	OAR21_24084777.1	24084.8	356
S1217		27	21	OAR21_6702302.1	s41655.1	5418.27	94
S1217		140	21	OAR21_2317339.1	OAR21_6702302.1	4384.96	47
S1217		702	21	s74845.1	OAR21_17189778.1	17189.8	241
S1217	CON	3	21	OAR21_6702302.1	OAR21_6702302.1	0	1
S1217	UNION	3	21	s74845.1	OAR21_17189778.1	17189.8	241
S1218		606	20	OAR20_50867736.1	OAR20_55563675.1	4695.94	69
S1218		40	20	s14624.1	OAR20_52019528.1	1930.35	35
S1218		829	20	OAR20_49963583.1	s46244.1	1975.9	36
S1218	CON	3	20	OAR20_50867736.1	s46244.1	1071.75	17
S1218	UNION	3	20	OAR20_49963583.1	OAR20_55563675.1	5600.09	88
S1219		606	20	s40462.1	s37556.1	1520.29	37
S1219		29	20	OAR20_45542235.1	s37556.1	1477.59	36
S1219		123	20	OAR20_46544221.1	OAR20_49453726.1	2909.51	63
S1219	CON	3	20	OAR20_46544221.1	s37556.1	475.603	13
S1219	UNION	3	20	s40462.1	OAR20_49453726.1	3954.19	87

S1229		27	20	OAR20_42482053.1	s54536.1	2596.59	58
S1229		844	20	OAR20_42071000_X.1	s25696.1	3826.95	84
S1229		29	20	OAR20_41289146.1	OAR20_42731904.1	1442.76	36
S1229	CON	3	20	OAR20_42482053.1	OAR20_42731904.1	249.851	8
S1229	UNION	3	20	OAR20_41289146.1	s25696.1	4608.8	103
S1236		140	20	OAR20_17174254.1	s16638.1	1783.83	32
S1236		627	20	s44306.1	s06051.1	3639.25	77
S1236		29	20	s38719.1	OAR20_17775022.1	4200.7	84
S1236	CON	3	20	OAR20_17174254.1	OAR20_17775022.1	600.768	9
S1236	UNION	3	20	s38719.1	s06051.1	6250.08	128
S1237		29	20	s38719.1	OAR20_17775022.1	4200.7	84
S1237		27	20	OAR20_11442511.1	s59954.1	2223.77	40
S1237		702	20	OAR20_27119.1	OAR20_14072832.1	14045.7	264
S1237	CON	3	20	s38719.1	s59954.1	91.957	2
S1237	UNION	3	20	OAR20_27119.1	OAR20_17775022.1	17747.9	335
S1240		702	20	OAR20_27119.1	OAR20_14072832.1	14045.7	264
S1240		152	20	OAR20_7626319.1	s15774.1	2135.34	43
S1240		90	20	s34560.1	s74774.1	5360.26	107
S1240	CON	3	20	s34560.1	s15774.1	1764.37	38
S1240	UNION	3	20	OAR20_27119.1	OAR20_14072832.1	14045.7	264
S1243		123	19	s06628.1	s48005.1	14962.5	245
S1243		29	19	s08949.1	s26710.1	3068.64	46
S1243		27	19	s21070.1	OAR19_58095077.1	2593.53	49
S1243	CON	3	19	s21070.1	s26710.1	1202.82	22
S1243	UNION	3	19	s06628.1	OAR19_58095077.1	16255.3	271
S1244		123	19	s06628.1	s48005.1	14962.5	245
S1244		140	19	s45366.1	s34745.1	3419.3	49
S1244		29	19	s08949.1	s26710.1	3068.64	46
S1244	CON	3	19	s08949.1	s34745.1	410.491	5
S1244	UNION	3	19	s06628.1	s48005.1	14962.5	245
S1257		79	19	s55682.1	OAR19_34354181.1	2692.56	49
S1257		45	19	OAR19_31737971_X.1	s23858.1	2050.04	36
S1257		844	19	OAR19_32284446.1	OAR19_37764352.1	5479.91	109
S1257	CON	3	19	OAR19_32284446.1	s23858.1	1503.57	28
S1257	UNION	3	19	s55682.1	OAR19_37764352.1	6102.73	119
S1261		507	19	OAR19_25643551.1	OAR19_29244492.1	3600.94	62
S1261		616	19	OAR19_24079470.1	OAR19_29193780.1	5114.31	84
S1261		49	19	OAR19_21684191.1	s55682.1	9977.43	163
S1261	CON	3	19	OAR19_25643551.1	OAR19_29193780.1	3550.23	60
S1261	UNION	3	19	OAR19_21684191.1	s55682.1	9977.43	163
S1262		616	19	OAR19_24079470.1	OAR19_29193780.1	5114.31	84
S1262		49	19	OAR19_21684191.1	s55682.1	9977.43	163
S1262		702	19	OAR19_7253713.1	s27711.1	16961.5	313
S1262	CON	3	19	OAR19_24079470.1	s27711.1	135.761	3
S1262	UNION	3	19	OAR19_7253713.1	s55682.1	24407.9	439
S1264		507	19	s21945.1	s40021.1	5779.07	97

S1264		27	19	s09766.1	OAR19_2409205.1	2409.2	39
S1264		79	19	OAR19_1987551.1	OAR19_3684221.1	1696.67	33
S1264	CON	3	19	OAR19_1987551.1	OAR19_2409205.1	421.654	8
S1264	UNION	3	19	s21945.1	s40021.1	5779.07	97
S1265		606	18	OAR18_52219091.1	s59424.1	5545.32	70
S1265		829	18	OAR18_56445082.1	s36667.1	4906.48	93
S1265		507	18	s14692.1	OAR18_59623348.1	2295.67	44
S1265	CON	3	18	s14692.1	s59424.1	436.737	7
S1265	UNION	3	18	OAR18_52219091.1	s36667.1	9132.47	145
S1266		606	18	OAR18_52219091.1	s59424.1	5545.32	70
S1266		140	18	OAR18_54263457.1	OAR18_57166462.1	2903.01	35
S1266		829	18	OAR18_56445082.1	s36667.1	4906.48	93
S1266	CON	3	18	OAR18_56445082.1	OAR18_57166462.1	721.38	8
S1266	UNION	3	18	OAR18_52219091.1	s36667.1	9132.47	145
S1271		140	18	s31152.1	OAR18_21577929.1	1843.36	37
S1271		45	18	s66371.1	OAR18_24036473.1	4407.12	76
S1271		507	18	s44433.1	OAR18_29143813.1	9489.05	160
S1271	CON	3	18	s31152.1	OAR18_21577929.1	1843.36	37
S1271	UNION	3	18	s66371.1	OAR18_29143813.1	9514.46	161
S1273		40	18	OAR18_12013453.1	s73019.1	2210.28	40
S1273		629	18	OAR18_12872464.1	OAR18_14500139.1	1627.67	33
S1273		140	18	s24860.1	OAR18_16428067.1	2788.24	58
S1273	CON	3	18	s24860.1	s73019.1	583.902	8
S1273	UNION	3	18	OAR18_12013453.1	OAR18_16428067.1	4414.61	90
S1276		629	18	OAR18_2457937_X.1	OAR18_9257846.1	6799.91	133
S1276		606	18	s07816.1	OAR18_9083749.1	9083.75	161
S1276		123	18	OARUn.992_53657_X.1	s30059.1	7868.3	144
S1276	CON	3	18	OAR18_2457937_X.1	s30059.1	5410.36	102
S1276	UNION	3	18	OARUn.992_53657_X.1	OAR18_9257846.1	9257.85	175
S1279		616	17	s68769.1	s27077.1	2265.51	34
S1279		844	17	s06760.1	s72479.1	2205.58	38
S1279		45	17	s15206.1	s22205.1	6445.19	96
S1279	CON	3	17	s68769.1	s72479.1	89.484	3
S1279	UNION	3	17	s15206.1	s22205.1	6445.19	96
S1280		844	17	s06760.1	s72479.1	2205.58	38
S1280		49	17	s47858.1	s22875.1	2512.56	32
S1280		45	17	s15206.1	s22205.1	6445.19	96
S1280	CON	3	17	s06760.1	s22875.1	118.536	4
S1280	UNION	3	17	s47858.1	s22205.1	6864.9	99
S1281		49	17	s47858.1	s22875.1	2512.56	32
S1281		702	17	OAR17_36966397.1	OAR17_74039265.1	37072.9	600
S1281		45	17	s15206.1	s22205.1	6445.19	96
S1281	CON	3	17	s15206.1	OAR17_74039265.1	1870.06	24
S1281	UNION	3	17	OAR17_36966397.1	s22205.1	41648	672
S1292		49	17	OAR17_15114925.1	OAR17_33024300.1	17909.4	313
S1292		61	17	OAR17_31146799.1	OAR17_33487124.1	2340.32	46

S1292		629	17	OAR17_32056831.1	OAR17_34275919.1	2219.09	43
S1292	CON	3	17	OAR17_32056831.1	OAR17_33024300.1	967.469	18
S1292	UNION	3	17	OAR17_15114925.1	OAR17_34275919.1	19161	338
S1293		61	17	OAR17_31146799.1	OAR17_33487124.1	2340.32	46
S1293		829	17	OAR17_29292591.1	OAR17_31561091.1	2268.5	41
S1293		49	17	OAR17_15114925.1	OAR17_33024300.1	17909.4	313
S1293	CON	3	17	OAR17_31146799.1	OAR17_31561091.1	414.292	10
S1293	UNION	3	17	OAR17_15114925.1	OAR17_33487124.1	18372.2	322
S1294		829	17	OAR17_29292591.1	OAR17_31561091.1	2268.5	41
S1294		49	17	OAR17_15114925.1	OAR17_33024300.1	17909.4	313
S1294		140	17	OAR17_28121648.1	s05512.1	1821.91	32
S1294	CON	3	17	OAR17_29292591.1	s05512.1	650.972	11
S1294	UNION	3	17	OAR17_15114925.1	OAR17_33024300.1	17909.4	313
S1302		140	17	OAR17_28121648.1	s05512.1	1821.91	32
S1302		801	17	OAR17_27620209.1	OAR17_29260298.1	1640.09	32
S1302		49	17	OAR17_15114925.1	OAR17_33024300.1	17909.4	313
S1302	CON	3	17	OAR17_28121648.1	OAR17_29260298.1	1138.65	20
S1302	UNION	3	17	OAR17_15114925.1	OAR17_33024300.1	17909.4	313
S1305		702	16	OAR16_63959301.1	s59627.1	11378.9	220
S1305		126	16	OAR16_66186381.1	OAR16_72265538.1	6079.16	118
S1305		829	16	s32243.1	OAR16_74018348_X.1	2115.33	47
S1305	CON	3	16	s32243.1	OAR16_72265538.1	362.518	11
S1305	UNION	3	16	OAR16_63959301.1	s59627.1	11378.9	220
S1306		702	16	OAR16_63959301.1	s59627.1	11378.9	220
S1306		126	16	OAR16_66186381.1	OAR16_72265538.1	6079.16	118
S1306		123	16	OAR16_69472743.1	OAR16_71364480.1	1891.74	32
S1306	CON	3	16	OAR16_69472743.1	OAR16_71364480.1	1891.74	32
S1306	UNION	3	16	OAR16_63959301.1	s59627.1	11378.9	220
S1311		702	16	OAR16_63959301.1	s59627.1	11378.9	220
S1311		629	16	s07759.1	OAR16_64570335.1	1827.46	36
S1311		507	16	OAR16_63959301.1	OAR16_65917082.1	1957.78	37
S1311	CON	3	16	OAR16_63959301.1	OAR16_64570335.1	611.034	13
S1311	UNION	3	16	s07759.1	s59627.1	12595.3	243
S1312		61	16	OAR16_32465649.1	OAR16_50643262_X.1	18177.6	326
S1312		616	16	OAR16_40749182.1	OAR16_55771610.1	15022.4	264
S1312		123	16	OAR16_41066473.1	OAR16_43133409.1	2066.94	41
S1312	CON	3	16	OAR16_41066473.1	OAR16_43133409.1	2066.94	41
S1312	UNION	3	16	OAR16_32465649.1	OAR16_55771610.1	23306	414
S1313		606	16	s61872.1	OAR16_32278875.1	10181.3	193
S1313		126	16	s55728.1	OAR16_31959917.1	3660.62	71
S1313		40	16	OAR16_31845248.1	OAR16_33763548.1	1918.3	42
S1313	CON	3	16	OAR16_31845248.1	OAR16_31959917.1	114.669	3
S1313	UNION	3	16	s61872.1	OAR16_33763548.1	11666	227
S1314		702	16	s52990.1	OAR16_10814090.1	10538	191
S1314		616	16	s09833.1	OAR16_6409309.1	1891.74	37
S1314		79	16	s09833.1	OAR16_7116193.1	2598.62	50

S1314	CON	3	16	s09833.1	OAR16_6409309.1	1891.74	37
S1314	UNION	3	16	s52990.1	OAR16_10814090.1	10538	191
S1315		61	15	OAR15_62680174.1	OAR15_65584079.1	2903.91	67
S1315		702	15	OAR15_63768341.1	OAR15_66653722.1	2885.38	65
S1315		844	15	s45839.1	OAR15_65710884_X.1	1482.69	35
S1315	CON	3	15	s45839.1	OAR15_65584079.1	1355.89	33
S1315	UNION	3	15	OAR15_62680174.1	OAR15_66653722.1	3973.55	87
S1316		126	15	s25555.1	OAR15_39421343.1	3025.36	55
S1316		49	15	OAR15_35705338.1	OAR15_38181495.1	2476.16	47
S1316		606	15	OAR15_35392874.1	OAR15_37160581.1	1767.71	36
S1316	CON	3	15	s25555.1	OAR15_37160581.1	764.599	16
S1316	UNION	3	15	OAR15_35392874.1	OAR15_39421343.1	4028.47	75
S1326		702	15	s33423.1	s34566.1	24919.4	406
S1326		49	15	OAR15_4725093.1	OAR15_14317931.1	9592.84	155
S1326		27	15	OAR15_12128190.1	s35677.1	1460.32	31
S1326	CON	3	15	OAR15_12128190.1	s35677.1	1460.32	31
S1326	UNION	3	15	s33423.1	s34566.1	24919.4	406
S1327		507	15	OAR15_8447146.1	OAR15_11973263.1	3526.12	57
S1327		49	15	OAR15_4725093.1	OAR15_14317931.1	9592.84	155
S1327		702	15	s33423.1	s34566.1	24919.4	406
S1327	CON	3	15	OAR15_8447146.1	OAR15_11973263.1	3526.12	57
S1327	UNION	3	15	s33423.1	s34566.1	24919.4	406
S1328		152	15	s74830.1	OAR15_3794544.1	3794.54	65
S1328		702	15	s33423.1	s34566.1	24919.4	406
S1328		157	15	OAR15_2886961.1	s14178.1	2148.25	37
S1328	CON	3	15	OAR15_2886961.1	OAR15_3794544.1	907.583	14
S1328	UNION	3	15	s74830.1	s34566.1	24919.4	409
S1329		152	14	s70225.1	s63574.1	3879.52	30
S1329		126	14	OAR14_58104354.1	s36676.1	6513.92	50
S1329		123	14	s09739.1	s73257.1	7402.46	63
S1329	CON	3	14	s70225.1	s63574.1	3879.52	30
S1329	UNION	3	14	s09739.1	s73257.1	7402.46	63
S1332		702	14	s22016.1	s49153.1	8137.25	142
S1332		627	14	OAR14_18935490.1	s63952.1	3002.98	58
S1332		616	14	s39735.1	OAR14_21824579.1	1800.95	39
S1332	CON	3	14	s39735.1	OAR14_21824579.1	1800.95	39
S1332	UNION	3	14	s22016.1	s49153.1	8137.25	142
S1333		702	14	s54311.1	s51612.1	6481.31	111
S1333		606	14	OAR14_1720811.1	OAR14_4424829.1	2704.02	50
S1333		801	14	DU206491_155.1	OAR14_4424829.1	2428.84	44
S1333	CON	3	14	DU206491_155.1	OAR14_4424829.1	2428.84	44
S1333	UNION	3	14	s54311.1	s51612.1	6481.31	111
S1334		507	13	s28658.1	OAR13_82500579.1	3148.43	62
S1334		29	13	OAR13_80012567.1	s64909.1	2537.79	56
S1334		616	13	OAR13_81897604.1	s03117.1	1965.48	36
S1334	CON	3	13	OAR13_81897604.1	OAR13_82500579.1	602.975	11

S1334	UNION	3	13	s28658.1	s03117.1	4510.93	87
S1336		702	13	s43103.1	s69997.1	32663.3	559
S1336		79	13	s13366.1	s49999.1	5945.38	115
S1336		157	13	s67695.1	OAR13_74948343.1	2275.72	43
S1336	CON	3	13	s67695.1	OAR13_74948343.1	2275.72	43
S1336	UNION	3	13	s43103.1	s69997.1	32663.3	559
S1337		844	13	s17883.1	OAR13_68528151.1	4897.41	78
S1337		606	13	OAR13_60423450.1	s33005.1	3263.21	60
S1337		702	13	s43103.1	s69997.1	32663.3	559
S1337	CON	3	13	s17883.1	s33005.1	55.923	2
S1337	UNION	3	13	s43103.1	s69997.1	32663.3	559
S1345		702	13	s43103.1	s69997.1	32663.3	559
S1345		79	13	OAR13_53748496.1	s31619.1	2561.29	33
S1345		616	13	OAR13_54850095.1	OAR13_57402806.1	2552.71	39
S1345	CON	3	13	OAR13_54850095.1	s31619.1	1459.69	18
S1345	UNION	3	13	s43103.1	s69997.1	32663.3	559
S1349		79	13	OAR13_53748496.1	s31619.1	2561.29	33
S1349		844	13	OAR13_47720904.1	s20479.1	6338.95	104
S1349		702	13	s43103.1	s69997.1	32663.3	559
S1349	CON	3	13	OAR13_53748496.1	s20479.1	311.354	4
S1349	UNION	3	13	s43103.1	s69997.1	32663.3	559
S1355		829	13	s12849.1	OAR13_20578061.1	2410.3	38
S1355		616	13	OAR13_19366874.1	s47163.1	7083.23	139
S1355		123	13	s43905.1	OAR13_26635884.1	6436.53	127
S1355	CON	3	13	s43905.1	OAR13_20578061.1	378.708	10
S1355	UNION	3	13	s12849.1	OAR13_26635884.1	8468.12	155
S1362		49	13	OAR13_13577766.1	OAR13_17982125.1	4404.36	89
S1362		829	13	OAR13_16575159.1	s35531.1	1525.75	32
S1362		702	13	s44144.1	OAR13_19332393.1	2130.3	31
S1362	CON	3	13	s44144.1	OAR13_17982125.1	780.029	17
S1362	UNION	3	13	OAR13_13577766.1	OAR13_19332393.1	5754.63	103
S1363		829	13	s65179.1	OAR13_13952314.1	3280.8	41
S1363		702	13	OAR13_4448234.1	OAR13_11941763.1	7493.53	117
S1363		126	13	OAR13_2980585_X.1	s00832.1	9013.02	139
S1363	CON	3	13	s65179.1	OAR13_11941763.1	1270.25	5
S1363	UNION	3	13	OAR13_2980585_X.1	OAR13_13952314.1	10971.7	174
S1366		627	12	OAR12_82122841.1	OAR12_83955714.1	1832.87	34
S1366		616	12	s52578.1	s09138.1	2230.84	40
S1366		61	12	s51577.1	s33621.1	2613.36	41
S1366	CON	3	12	s51577.1	OAR12_83955714.1	167.025	5
S1366	UNION	3	12	OAR12_82122841.1	s33621.1	4279.2	70
S1370		702	12	OAR12_80045456.1	s38430.1	2801.48	51
S1370		627	12	OAR12_82122841.1	OAR12_83955714.1	1832.87	34
S1370		616	12	s52578.1	s09138.1	2230.84	40
S1370	CON	3	12	s52578.1	s38430.1	375.609	7
S1370	UNION	3	12	OAR12_80045456.1	s09138.1	4656.71	84

S1382		90	12	OAR12_72461244.1	s65734.1	2576.88	55
S1382		40	12	OAR12_72598226.1	OAR12_74743841.1	2145.61	46
S1382		629	12	s04331.1	OAR12_74743841.1	1367.59	30
S1382	CON	3	12	s04331.1	OAR12_74743841.1	1367.59	30
S1382	UNION	3	12	OAR12_72461244.1	s65734.1	2576.88	55
S1388		507	12	s13792.1	s57817.1	31188.2	514
S1388		702	12	s66672.1	s60216.1	3126.9	62
S1388		606	12	s61648.1	OAR12_10619267.1	6012.46	53
S1388	CON	3	12	s61648.1	s60216.1	585.336	8
S1388	UNION	3	12	s13792.1	s57817.1	31188.2	514
S1392		507	12	s13792.1	s57817.1	31188.2	514
S1392		702	12	s66672.1	s60216.1	3126.9	62
S1392		157	12	OAR12_2860767.1	OAR12_4247318.1	1386.55	30
S1392	CON	3	12	OAR12_2860767.1	OAR12_4247318.1	1386.55	30
S1392	UNION	3	12	s13792.1	s57817.1	31188.2	514
S1393		702	11	OAR11_45618835.1	OAR11_53479950.1	7861.11	136
S1393		90	11	s07556.1	OAR11_55421823.1	8158.13	135
S1393		629	11	s13742.1	OAR11_61687914.1	12951	212
S1393	CON	3	11	s13742.1	OAR11_53479950.1	4742.99	78
S1393	UNION	3	11	OAR11_45618835.1	OAR11_61687914.1	16069.1	270
S1396		629	11	OAR11_34446037.1	OAR11_37253436.1	2807.4	52
S1396		49	11	OAR11_34446037.1	s50883.1	3076.39	57
S1396		29	11	s12433.1	s70389.1	1686.29	30
S1396	CON	3	11	s12433.1	OAR11_37253436.1	996.198	19
S1396	UNION	3	11	OAR11_34446037.1	s70389.1	3497.49	63
S1399		702	11	OAR11_17172323.1	OAR11_33359712.1	16187.4	260
S1399		629	11	s30225.1	OAR11_32882457.1	2081.37	35
S1399		152	11	OAR11_32301525.1	OAR11_34348656.1	2047.13	32
S1399	CON	3	11	OAR11_32301525.1	OAR11_32882457.1	580.932	9
S1399	UNION	3	11	OAR11_17172323.1	OAR11_34348656.1	17176.3	277
S1400		702	11	OAR11_17172323.1	OAR11_33359712.1	16187.4	260
S1400		79	11	s73325.1	s62452.1	1651.72	35
S1400		629	11	s30225.1	OAR11_32882457.1	2081.37	35
S1400	CON	3	11	s30225.1	s62452.1	398.717	9
S1400	UNION	3	11	OAR11_17172323.1	OAR11_33359712.1	16187.4	260
S1412		45	10	OAR10_79145098.1	OAR10_87563877.1	8418.78	181
S1412		79	10	OAR10_79145098.1	s67060.1	9854.04	210
S1412		152	10	OAR10_84897197.1	OAR10_88168947.1	3271.75	67
S1412	CON	3	10	OAR10_84897197.1	OAR10_87563877.1	2666.68	56
S1412	UNION	3	10	OAR10_79145098.1	s67060.1	9854.04	210
S1413		507	10	OAR10_54127756.1	s31288.1	7114.03	124
S1413		27	10	OAR10_52097098.1	OAR10_55148103.1	3051.01	65
S1413		45	10	OAR10_35722333.1	OAR10_57396545.1	21674.2	405
S1413	CON	3	10	OAR10_54127756.1	OAR10_55148103.1	1020.35	20
S1413	UNION	3	10	OAR10_35722333.1	s31288.1	25519.4	468

S1416		507	10	OAR10_15620012.1	OAR10_19558015.1	3938	70
S1416		844	10	OAR10_11311566.1	OAR10_15972516.1	4660.95	92
S1416		702	10	s49567.1	OAR10_48462141.1	48462.1	914
S1416	CON	3	10	OAR10_15620012.1	OAR10_15972516.1	352.504	7
S1416	UNION	3	10	s49567.1	OAR10_48462141.1	48462.1	914
S1417		61	10	OAR10_9852956.1	s23854.1	2137.39	41
S1417		29	10	s62543.1	s72120.1	1935.26	37
S1417		702	10	s49567.1	OAR10_48462141.1	48462.1	914
S1417	CON	3	10	OAR10_9852956.1	s72120.1	152.651	4
S1417	UNION	3	10	s49567.1	OAR10_48462141.1	48462.1	914
S1418		616	9	OAR9_94565556_X.1	OAR9_96141857.1	1576.3	30
S1418		702	9	OAR9_38323108.1	OAR9_96544082.1	58221	1087
S1418		29	9	OAR9_94742308.1	s44758.1	2123.59	44
S1418	CON	3	9	OAR9_94742308.1	OAR9_96141857.1	1399.55	28
S1418	UNION	3	9	OAR9_38323108.1	s44758.1	58542.8	1094
S1419		702	9	OAR9_38323108.1	OAR9_96544082.1	58221	1087
S1419		152	9	OAR9_77508470.1	s62001.1	3135.15	63
S1419		140	9	s10879.1	s05406.1	2485.9	47
S1419	CON	3	9	s10879.1	s62001.1	445.803	8
S1419	UNION	3	9	OAR9_38323108.1	OAR9_96544082.1	58221	1087
S1433		29	9	OAR9_69054720.1	OAR9_73056873.1	4002.15	80
S1433		507	9	OAR9_66516428.1	OAR9_70808263.1	4291.84	72
S1433		702	9	OAR9_38323108.1	OAR9_96544082.1	58221	1087
S1433	CON	3	9	OAR9_69054720.1	OAR9_70808263.1	1753.54	30
S1433	UNION	3	9	OAR9_38323108.1	OAR9_96544082.1	58221	1087
S1434		79	9	s73465.1	OAR9_40771150.1	8319.46	179
S1434		45	9	s68634.1	s50783.1	21048.3	358
S1434		702	9	OAR9_38323108.1	OAR9_96544082.1	58221	1087
S1434	CON	3	9	OAR9_38323108.1	OAR9_40771150.1	2448.04	49
S1434	UNION	3	9	s73465.1	OAR9_96544082.1	64092.4	1217
S1439		126	9	s59135.1	OAR9_20804687.1	2341.2	49
S1439		606	9	s18791.1	s12376.1	1409.01	30
S1439		45	9	OAR9_18493683.1	s19052.1	5564.34	125
S1439	CON	3	9	OAR9_18493683.1	s12376.1	103.495	3
S1439	UNION	3	9	s18791.1	s19052.1	6869.85	152
S1442		844	9	OAR9_5773857.1	s10011.1	10109	172
S1442		152	9	OAR9_8876882.1	s21274.1	4522.12	76
S1442		27	9	OAR9_11283827.1	s21274.1	2115.18	31
S1442	CON	3	9	OAR9_11283827.1	s21274.1	2115.18	31
S1442	UNION	3	9	OAR9_5773857.1	s10011.1	10109	172
S1443		844	9	OAR9_5773857.1	s10011.1	10109	172
S1443		629	9	OAR9_3164827.1	OAR9_5842029.1	2677.2	53
S1443		123	9	OAR9_1203135.1	s25647.1	4723.76	95
S1443	CON	3	9	OAR9_5773857.1	OAR9_5842029.1	68.172	3
S1443	UNION	3	9	OAR9_1203135.1	s10011.1	14679.7	263
S1444		629	9	OAR9_3164827.1	OAR9_5842029.1	2677.2	53

S1444		79	9	OAR9_2798433.1	OAR9_4459408.1	1660.97	32
S1444		123	9	OAR9_1203135.1	s25647.1	4723.76	95
S1444	CON	3	9	OAR9_3164827.1	OAR9_4459408.1	1294.58	27
S1444	UNION	3	9	OAR9_1203135.1	s25647.1	4723.76	95
S1450		61	8	OAR8_91007371.1	OAR8_93131673.1	2124.3	53
S1450		49	8	OAR8_91293224.1	s52430.1	1238.58	33
S1450		606	8	OAR8_91701722.1	s53145.1	2751.4	60
S1450	CON	3	8	OAR8_91701722.1	s52430.1	830.078	22
S1450	UNION	3	8	OAR8_91007371.1	s53145.1	3445.75	78
S1455		507	8	OAR8_85811057.1	s41250.1	2810.84	53
S1455		79	8	OAR8_81635650.1	OAR8_87540028.1	5904.38	127
S1455		606	8	OAR8_86919224.1	OAR8_89769401.1	2850.18	48
S1455	CON	3	8	OAR8_86919224.1	OAR8_87540028.1	620.804	14
S1455	UNION	3	8	OAR8_81635650.1	OAR8_89769401.1	8133.75	161
S1466		49	8	OAR8_77578890.1	s01826.1	8664.11	175
S1466		126	8	s00802.1	OAR8_82107007.1	3531.36	62
S1466		79	8	OAR8_81635650.1	OAR8_87540028.1	5904.38	127
S1466	CON	3	8	OAR8_81635650.1	OAR8_82107007.1	471.357	10
S1466	UNION	3	8	OAR8_77578890.1	OAR8_87540028.1	9961.14	204
S1471		556	8	OAR8_76403936.1	OAR8_78079638.1	1675.7	31
S1471		27	8	OAR8_73702430.1	s22687.1	2779.43	58
S1471		140	8	OAR8_73355352.1	s22687.1	3126.51	64
S1471	CON	3	8	OAR8_76403936.1	s22687.1	77.926	3
S1471	UNION	3	8	OAR8_73355352.1	OAR8_78079638.1	4724.29	92
S1476		507	8	s24061.1	OAR8_74194992.1	1639.28	32
S1476		61	8	OAR8_69411239.1	OAR8_73226726.1	3815.49	73
S1476		152	8	OAR8_72707636.1	OAR8_74991269.1	2283.63	44
S1476	CON	3	8	OAR8_72707636.1	OAR8_73226726.1	519.09	11
S1476	UNION	3	8	OAR8_69411239.1	OAR8_74991269.1	5580.03	106
S1480		140	8	s61158.1	OAR8_52791160.1	9209.77	166
S1480		27	8	OAR8_49458688.1	OAR8_51041603.1	1582.91	36
S1480		507	8	OAR8_50006699.1	OAR8_52500656.1	2493.96	41
S1480	CON	3	8	OAR8_50006699.1	OAR8_51041603.1	1034.9	25
S1480	UNION	3	8	s61158.1	OAR8_52791160.1	9209.77	166
S1485		45	8	OAR8_17854216.1	s00683.1	2986.56	63
S1485		126	8	OAR8_16617875.1	OAR8_18017904.1	1400.03	33
S1485		27	8	OAR8_16197437.1	OAR8_22199299_X.1	6001.86	125
S1485	CON	3	8	OAR8_17854216.1	OAR8_18017904.1	163.688	5
S1485	UNION	3	8	OAR8_16197437.1	OAR8_22199299_X.1	6001.86	125
S1492		79	8	DU438038_308.1	OAR8_12613673.1	9152.14	171
S1492		90	8	OAR8_7630333.1	s19466.1	2567.86	35
S1492		801	8	OAR8_9152520.1	OAR8_11530695.1	2378.18	44
S1492	CON	3	8	OAR8_9152520.1	s19466.1	1045.68	18
S1492	UNION	3	8	DU438038_308.1	OAR8_12613673.1	9152.14	171
S1504		629	7	OAR7_106304514.1	OAR7_108923470.1	2618.96	49
S1504		627	7	OAR7_106304514.1	OAR7_108844976.1	2540.46	47

S1504		49	7	OAR7_107340259.1	OAR7_108923470.1	1583.21	31
S1504	CON	3	7	OAR7_107340259.1	OAR7_108844976.1	1504.72	29
S1504	UNION	3	7	OAR7_106304514.1	OAR7_108923470.1	2618.96	49
S1505		629	7	OAR7_106304514.1	OAR7_108923470.1	2618.96	49
S1505		627	7	OAR7_106304514.1	OAR7_108844976.1	2540.46	47
S1505		45	7	s37625.1	s69049.1	1451.29	31
S1505	CON	3	7	OAR7_106304514.1	s69049.1	157.402	4
S1505	UNION	3	7	s37625.1	OAR7_108923470.1	3912.84	76
S1507		45	7	s37625.1	s69049.1	1451.29	31
S1507		616	7	OAR7_104475739.1	s16852.1	1555.5	31
S1507		702	7	OAR7_97739969.1	OAR7_105267200.1	7527.23	129
S1507	CON	3	7	s37625.1	OAR7_105267200.1	256.57	6
S1507	UNION	3	7	OAR7_97739969.1	s69049.1	8721.95	154
S1508		616	7	OAR7_104475739.1	s16852.1	1555.5	31
S1508		29	7	OAR7_102949447.1	s58900.1	2012.6	32
S1508		702	7	OAR7_97739969.1	OAR7_105267200.1	7527.23	129
S1508	CON	3	7	OAR7_104475739.1	s58900.1	486.305	9
S1508	UNION	3	7	OAR7_97739969.1	s16852.1	8291.27	145
S1510		702	7	OAR7_97739969.1	OAR7_105267200.1	7527.23	129
S1510		606	7	OAR7_100406183.1	s46899.1	3556.11	59
S1510		29	7	OAR7_102949447.1	s58900.1	2012.6	32
S1510	CON	3	7	OAR7_102949447.1	s46899.1	1012.84	17
S1510	UNION	3	7	OAR7_97739969.1	OAR7_105267200.1	7527.23	129
S1511		702	7	OAR7_97739969.1	OAR7_105267200.1	7527.23	129
S1511		126	7	OAR7_94733688.1	OAR7_99255226.1	4521.54	85
S1511		152	7	OAR7_97719696.1	OAR7_99836613.1	2116.92	37
S1511	CON	3	7	OAR7_97739969.1	OAR7_99255226.1	1515.26	25
S1511	UNION	3	7	OAR7_94733688.1	OAR7_105267200.1	10533.5	189
S1515		844	7	s03357.1	s61284.1	4208.81	84
S1515		61	7	OAR7_79189143.1	s15556.1	5289.18	104
S1515		627	7	OAR7_78268516.1	s03357.1	1902.67	35
S1515	CON	3	7	s03357.1	s03357.1	0	1
S1515	UNION	3	7	OAR7_78268516.1	s15556.1	6209.8	120
S1516		616	7	s56266.1	OAR7_31373399.1	1556.8	35
S1516		126	7	OAR7_31134779.1	OAR7_32846682.1	1711.9	37
S1516		45	7	OAR7_31172405.1	OAR7_58958307.1	27785.9	542
S1516	CON	3	7	OAR7_31172405.1	OAR7_31373399.1	200.994	6
S1516	UNION	3	7	s56266.1	OAR7_58958307.1	29141.7	571
S1519		123	7	s13188.1	s71719.1	12317	181
S1519		126	7	OAR7_21170284.1	OAR7_24149695.1	2979.41	39
S1519		157	7	OAR7_22306460.1	OAR7_29584778.1	7278.32	78
S1519	CON	3	7	OAR7_22306460.1	OAR7_24149695.1	1843.23	19
S1519	UNION	3	7	s13188.1	OAR7_29584778.1	12352.9	182
S1525		45	7	s23542.1	s45087.1	5861.48	112
S1525		90	7	s32513.1	OAR7_10343956.1	2617.24	53
S1525		123	7	OAR7_9376658.1	s19744.1	3586.07	67

S1525	CON	3	7	OAR7_9376658.1	OAR7_10343956.1	967.298	18
S1525	UNION	3	7	s32513.1	s45087.1	6405.8	126
S1536		702	6	OAR6_124592433.1	OAR6_129053557.1	4461.12	86
S1536		79	6	OAR6_125967269.1	s55582.1	2969.33	57
S1536		629	6	OAR6_127050597.1	OAR6_129053557.1	2002.96	40
S1536	CON	3	6	OAR6_127050597.1	s55582.1	1886	37
S1536	UNION	3	6	OAR6_124592433.1	OAR6_129053557.1	4461.12	86
S1537		616	6	OAR6_124394473.1	s14915.1	1746.14	35
S1537		702	6	OAR6_124592433.1	OAR6_129053557.1	4461.12	86
S1537		79	6	OAR6_125967269.1	s55582.1	2969.33	57
S1537	CON	3	6	OAR6_125967269.1	s14915.1	173.343	5
S1537	UNION	3	6	OAR6_124394473.1	OAR6_129053557.1	4659.08	90
S1539		702	6	OAR6_124592433.1	OAR6_129053557.1	4461.12	86
S1539		29	6	OAR6_121651498.1	OAR6_124727445.1	3075.95	59
S1539		616	6	OAR6_124394473.1	s14915.1	1746.14	35
S1539	CON	3	6	OAR6_124592433.1	OAR6_124727445.1	135.012	5
S1539	UNION	3	6	OAR6_121651498.1	OAR6_129053557.1	7402.06	140
S1540		606	6	OAR6_119125205.1	OAR6_121181023.1	2055.82	46
S1540		844	6	s59122.1	OAR6_119125205.1	3781.3	51
S1540		702	6	s03175.1	s49558.1	3896.24	53
S1540	CON	3	6	OAR6_119125205.1	OAR6_119125205.1	0	1
S1540	UNION	3	6	s03175.1	OAR6_121181023.1	5895.75	97
S1541		27	6	OAR6_110447914.1	s05385.1	1913.1	41
S1541		29	6	OAR6_109808350.1	s05385.1	2552.67	56
S1541		140	6	OAR6_109808350.1	OAR6_111646056.1	1837.71	44
S1541	CON	3	6	OAR6_110447914.1	OAR6_111646056.1	1198.14	29
S1541	UNION	3	6	OAR6_109808350.1	s05385.1	2552.67	56
S1542		45	6	OAR6_96497355.1	OAR6_107118527.1	10621.2	208
S1542		123	6	s52502.1	OAR6_105661466.1	2254.54	35
S1542		90	6	OAR6_105017216.1	OAR6_108898159.1	3880.94	72
S1542	CON	3	6	OAR6_105017216.1	OAR6_105661466.1	644.25	9
S1542	UNION	3	6	OAR6_96497355.1	OAR6_108898159.1	12400.8	243
S1543		90	6	OAR6_101640082.1	OAR6_103074951.1	1434.87	33
S1543		45	6	OAR6_96497355.1	OAR6_107118527.1	10621.2	208
S1543		844	6	OAR6_101563461.1	OAR6_104016857_X.1	2453.4	49
S1543	CON	3	6	OAR6_101640082.1	OAR6_103074951.1	1434.87	33
S1543	UNION	3	6	OAR6_96497355.1	OAR6_107118527.1	10621.2	208
S1544		507	6	OAR6_95218086.1	OAR6_99241952.1	4023.87	79
S1544		629	6	s49331.1	OAR6_97850334.1	7126.43	128
S1544		45	6	OAR6_96497355.1	OAR6_107118527.1	10621.2	208
S1544	CON	3	6	OAR6_96497355.1	OAR6_97850334.1	1352.98	28
S1544	UNION	3	6	s49331.1	OAR6_107118527.1	16394.6	308
S1547		152	6	DU430803_572.1	OAR6_94868641.1	1361.57	33
S1547		140	6	OAR6_91920640.1	OAR6_94508578.1	2587.94	36
S1547		629	6	s49331.1	OAR6_97850334.1	7126.43	128
S1547	CON	3	6	DU430803_572.1	OAR6_94508578.1	1001.5	23

S1547	UNION	3	6	s49331.1	OAR6_97850334.1	7126.43	128
S1560		627	6	OAR6_66400799.1	OAR6_90538374.1	24137.6	431
S1560		90	6	s51667.1	OAR6_90538374.1	11120.6	185
S1560		616	6	OAR6_85367529.1	OAR6_90479997.1	5112.47	77
S1560	CON	3	6	OAR6_85367529.1	OAR6_90479997.1	5112.47	77
S1560	UNION	3	6	OAR6_66400799.1	OAR6_90538374.1	24137.6	431
S1561		90	6	s51667.1	OAR6_90538374.1	11120.6	185
S1561		140	6	s69231.1	OAR6_79639233.1	3393.23	59
S1561		627	6	OAR6_66400799.1	OAR6_90538374.1	24137.6	431
S1561	CON	3	6	s51667.1	OAR6_79639233.1	221.413	4
S1561	UNION	3	6	OAR6_66400799.1	OAR6_90538374.1	24137.6	431
S1563		556	6	OAR6_74224903.1	OAR6_76377079.1	2152.18	45
S1563		152	6	OAR6_71400162.1	OAR6_74224903.1	2824.74	58
S1563		627	6	OAR6_66400799.1	OAR6_90538374.1	24137.6	431
S1563	CON	3	6	OAR6_74224903.1	OAR6_74224903.1	0	1
S1563	UNION	3	6	OAR6_66400799.1	OAR6_90538374.1	24137.6	431
S1564		627	6	OAR6_66400799.1	OAR6_90538374.1	24137.6	431
S1564		90	6	OAR6_67781585.1	s33521.1	4904.72	95
S1564		152	6	OAR6_71400162.1	OAR6_74224903.1	2824.74	58
S1564	CON	3	6	OAR6_71400162.1	s33521.1	1286.14	28
S1564	UNION	3	6	OAR6_66400799.1	OAR6_90538374.1	24137.6	431
S1565		40	6	OAR6_62471770.1	OAR6_65237136.1	2765.37	57
S1565		627	6	s36343.1	OAR6_62795128.1	1664.66	33
S1565		123	6	OAR6_62227590.1	OAR6_64593905.1	2366.32	47
S1565	CON	3	6	OAR6_62471770.1	OAR6_62795128.1	323.358	5
S1565	UNION	3	6	s36343.1	OAR6_65237136.1	4106.67	85
S1566		61	6	OAR6_57881110.1	OAR6_62074943.1	4193.83	74
S1566		79	6	OAR6_51660421.1	s21191.1	10134.5	185
S1566		627	6	s36343.1	OAR6_62795128.1	1664.66	33
S1566	CON	3	6	s36343.1	s21191.1	664.498	18
S1566	UNION	3	6	OAR6_51660421.1	OAR6_62795128.1	11134.7	200
S1569		29	6	OAR6_55784046.1	OAR6_58211072.1	2427.03	48
S1569		79	6	OAR6_51660421.1	s21191.1	10134.5	185
S1569		629	6	OAR6_55321610.1	OAR6_57182975.1	1861.37	37
S1569	CON	3	6	OAR6_55784046.1	OAR6_57182975.1	1398.93	26
S1569	UNION	3	6	OAR6_51660421.1	s21191.1	10134.5	185
S1572		629	6	OAR6_55321610.1	OAR6_57182975.1	1861.37	37
S1572		702	6	s10352.1	OAR6_55321610.1	5954.38	118
S1572		79	6	OAR6_51660421.1	s21191.1	10134.5	185
S1572	CON	3	6	OAR6_55321610.1	OAR6_55321610.1	0	1
S1572	UNION	3	6	s10352.1	s21191.1	12427.7	236
S1573		61	5	DU254733_336.1	OAR5_114683952_X.1	4203.15	89
S1573		29	5	OAR5_111535489.1	OAR5_113136843.1	1601.35	37
S1573		90	5	OAR5_112354670.1	OAR5_114101380.1	1746.71	38
S1573	CON	3	5	OAR5_112354670.1	OAR5_113136843.1	782.173	18
S1573	UNION	3	5	DU254733_336.1	OAR5_114683952_X.1	4203.15	89

S1576		49	5	OAR5_107618071.1	OAR5_109537332.1	1919.26	42
S1576		90	5	OAR5_105969630.1	OAR5_108700536.1	2730.91	53
S1576		507	5	OAR5_105769947.1	s50506.1	2885.22	56
S1576	CON	3	5	OAR5_107618071.1	s50506.1	1037.1	22
S1576	UNION	3	5	OAR5_105769947.1	OAR5_109537332.1	3767.39	76
S1577		606	5	s01299.1	OAR5_86630257.1	3076.71	57
S1577		27	5	OAR5_79542638.1	s15500.1	5963.32	115
S1577		702	5	OAR5_77977258.1	OAR5_100127181.1	22149.9	428
S1577	CON	3	5	s01299.1	s15500.1	1952.41	36
S1577	UNION	3	5	OAR5_77977258.1	OAR5_100127181.1	22149.9	428
S1578		27	5	OAR5_73707380.1	OAR5_78020462.1	4313.08	92
S1578		606	5	OAR5_70256606.1	OAR5_82901477.1	12644.9	245
S1578		556	5	OAR5_74249563.1	s28640.1	1497.65	36
S1578	CON	3	5	OAR5_74249563.1	s28640.1	1497.65	36
S1578	UNION	3	5	OAR5_70256606.1	OAR5_82901477.1	12644.9	245
S1579		40	5	s36503.1	OAR5_24253861.1	3925.16	69
S1579		152	5	OAR5_22282164.1	s15153.1	2048.29	47
S1579		616	5	OAR5_23613118.1	OAR5_25164869.1	1551.75	33
S1579	CON	3	5	OAR5_23613118.1	OAR5_24253861.1	640.743	16
S1579	UNION	3	5	s36503.1	OAR5_25164869.1	4836.17	86
S1580		152	5	OAR5_22282164.1	s15153.1	2048.29	47
S1580		45	5	OAR5_10884558.1	s06935.1	11513.7	188
S1580		40	5	s36503.1	OAR5_24253861.1	3925.16	69
S1580	CON	3	5	OAR5_22282164.1	s06935.1	116.138	4
S1580	UNION	3	5	OAR5_10884558.1	s15153.1	13445.9	231
S1583		45	5	OAR5_10884558.1	s06935.1	11513.7	188
S1583		61	5	OAR5_12770106.1	OAR5_22276454.1	9506.35	152
S1583		40	5	s36503.1	OAR5_24253861.1	3925.16	69
S1583	CON	3	5	s36503.1	OAR5_22276454.1	1947.75	24
S1583	UNION	3	5	OAR5_10884558.1	OAR5_24253861.1	13369.3	229
S1584		507	4	OAR4_117802819.1	OAR4_121393593.1	3590.77	62
S1584		90	4	DU176899_379.1	s56605.1	1405.36	33
S1584		45	4	OAR4_116517081.1	OAR4_118303004.1	1785.92	44
S1584	CON	3	4	OAR4_117802819.1	OAR4_118303004.1	500.185	12
S1584	UNION	3	4	OAR4_116517081.1	OAR4_121393593.1	4876.51	94
S1589		45	4	OAR4_116517081.1	OAR4_118303004.1	1785.92	44
S1589		844	4	OAR4_115675953.1	s65922.1	1382.7	35
S1589		126	4	s31432.1	OAR4_116953207.1	2171.45	50
S1589	CON	3	4	OAR4_116517081.1	OAR4_116953207.1	436.126	13
S1589	UNION	3	4	s31432.1	OAR4_118303004.1	3521.24	81
S1590		126	4	OAR4_103661998.1	s45195.1	8630.78	167
S1590		27	4	s16058.1	OAR4_115829682.1	8547.19	152
S1590		507	4	OAR4_111473821.1	OAR4_115829682.1	4355.86	71
S1590	CON	3	4	OAR4_111473821.1	s45195.1	818.962	11
S1590	UNION	3	4	OAR4_103661998.1	OAR4_115829682.1	12167.7	227

S1593		126	4	OAR4_103661998.1	s45195.1	8630.78	167
S1593		27	4	s16058.1	OAR4_115829682.1	8547.19	152
S1593		123	4	OAR4_108117513.1	s26707.1	1949.09	35
S1593	CON	3	4	OAR4_108117513.1	s26707.1	1949.09	35
S1593	UNION	3	4	OAR4_103661998.1	OAR4_115829682.1	12167.7	227
S1594		126	4	OAR4_103661998.1	s45195.1	8630.78	167
S1594		61	4	OAR4_106415496.1	OAR4_107786618.1	1371.12	33
S1594		27	4	s16058.1	OAR4_115829682.1	8547.19	152
S1594	CON	3	4	s16058.1	OAR4_107786618.1	504.128	12
S1594	UNION	3	4	OAR4_103661998.1	OAR4_115829682.1	12167.7	227
S1607		507	4	OAR4_90105244.1	OAR4_92447165.1	2341.92	53
S1607		123	4	OAR4_87201676.1	OAR4_92644037.1	5442.36	118
S1607		140	4	OAR4_88648994.1	OAR4_91820024.1	3171.03	67
S1607	CON	3	4	OAR4_90105244.1	OAR4_91820024.1	1714.78	39
S1607	UNION	3	4	OAR4_87201676.1	OAR4_92644037.1	5442.36	118
S1608		140	4	OAR4_88648994.1	OAR4_91820024.1	3171.03	67
S1608		627	4	OAR4_83576387.1	OAR4_89216253.1	5639.87	112
S1608		123	4	OAR4_87201676.1	OAR4_92644037.1	5442.36	118
S1608	CON	3	4	OAR4_88648994.1	OAR4_89216253.1	567.259	12
S1608	UNION	3	4	OAR4_83576387.1	OAR4_92644037.1	9067.65	187
S1616		627	4	OAR4_70656759_X.1	s13803.1	5088.69	101
S1616		29	4	s00796.1	OAR4_70665707.1	1839.11	39
S1616		702	4	s58367.1	OAR4_85483475.1	25992.5	514
S1616	CON	3	4	OAR4_70656759_X.1	OAR4_70665707.1	8.947	2
S1616	UNION	3	4	s58367.1	OAR4_85483475.1	25992.5	514
S1620		702	4	s58367.1	OAR4_85483475.1	25992.5	514
S1620		152	4	OAR4_63706459.1	OAR4_69891218.1	6184.76	115
S1620		29	4	s00796.1	OAR4_70665707.1	1839.11	39
S1620	CON	3	4	s00796.1	OAR4_69891218.1	1064.62	18
S1620	UNION	3	4	s58367.1	OAR4_85483475.1	25992.5	514
S1629		616	4	OAR4_63468173.1	s75934.1	1686.15	32
S1629		157	4	OAR4_61284730.1	s64810.1	2274.83	45
S1629		702	4	s58367.1	OAR4_85483475.1	25992.5	514
S1629	CON	3	4	OAR4_63468173.1	s64810.1	91.386	3
S1629	UNION	3	4	s58367.1	OAR4_85483475.1	25992.5	514
S1630		61	4	OAR4_39813653.1	OAR4_56340637.1	16527	308
S1630		27	4	OAR4_38036161.1	OAR4_40146986.1	2110.82	50
S1630		606	4	OAR4_37940876.1	OAR4_55963926.1	18023	344
S1630	CON	3	4	OAR4_39813653.1	OAR4_40146986.1	333.333	8
S1630	UNION	3	4	OAR4_37940876.1	OAR4_56340637.1	18399.8	352
S1631		61	4	OAR4_27012742.1	OAR4_29317435.1	2304.69	56
S1631		29	4	OAR4_24184274.1	OAR4_27320940_X.1	3136.67	66
S1631		49	4	OAR4_22439455.1	OAR4_29317435.1	6877.98	155
S1631	CON	3	4	OAR4_27012742.1	OAR4_27320940_X.1	308.199	7
S1631	UNION	3	4	OAR4_22439455.1	OAR4_29317435.1	6877.98	155
S1638		702	4	OARUn.284_293028.1	OAR4_24251309.1	24251.3	470

S1638		45	4	OAR4_10748026.1	OAR4_12498072.1	1750.05	39
S1638		126	4	s29747.1	OAR4_16701883.1	4730.1	103
S1638	CON	3	4	s29747.1	OAR4_12498072.1	526.286	12
S1638	UNION	3	4	OARUn.284_293028.1	OAR4_24251309.1	24251.3	470
S1639		79	3	OAR3_229469681_X.1	s43578.1	2300.26	45
S1639		29	3	s65742.1	OAR3_229832073.1	4118	90
S1639		61	3	OAR3_226997236.1	s61647.1	2686.25	58
S1639	CON	3	3	OAR3_229469681_X.1	s61647.1	213.804	4
S1639	UNION	3	3	s65742.1	s43578.1	6055.87	128
S1641		152	3	OAR3_205307357.1	OAR3_207087501_X.1	1780.14	40
S1641		801	3	OAR3_202596537.1	OAR3_205542104.1	2945.57	56
S1641		507	3	OAR3_179244393.1	OAR3_211362922_X.1	32118.5	604
S1641	CON	3	3	OAR3_205307357.1	OAR3_205542104.1	234.747	5
S1641	UNION	3	3	OAR3_179244393.1	OAR3_211362922_X.1	32118.5	604
S1642		801	3	OAR3_202596537.1	OAR3_205542104.1	2945.57	56
S1642		507	3	OAR3_179244393.1	OAR3_211362922_X.1	32118.5	604
S1642		123	3	s09050.1	OAR3_202943170.1	11054.8	191
S1642	CON	3	3	OAR3_202596537.1	OAR3_202943170.1	346.633	8
S1642	UNION	3	3	OAR3_179244393.1	OAR3_211362922_X.1	32118.5	604
S1643		702	3	OAR3_190128314.1	OAR3_194730311.1	4602	79
S1643		140	3	OAR3_188369387.1	s01421.1	2146.03	47
S1643		507	3	OAR3_179244393.1	OAR3_211362922_X.1	32118.5	604
S1643	CON	3	3	OAR3_190128314.1	s01421.1	387.108	9
S1643	UNION	3	3	OAR3_179244393.1	OAR3_211362922_X.1	32118.5	604
S1644		152	3	s44438.1	OAR3_178996653.1	3191.08	37
S1644		606	3	OAR3_177909045.1	s26854.1	6000.29	115
S1644		702	3	OAR3_178121896.1	s16918.1	11927	230
S1644	CON	3	3	OAR3_178121896.1	OAR3_178996653.1	874.757	14
S1644	UNION	3	3	s44438.1	s16918.1	14243.3	253
S1645		627	3	OAR3_155236610.1	s75549.1	19415.4	375
S1645		629	3	OAR3_155236610.1	s75549.1	19415.4	375
S1645		140	3	OAR3_169586431.1	s05066.1	1509.69	33
S1645	CON	3	3	OAR3_169586431.1	s05066.1	1509.69	33
S1645	UNION	3	3	OAR3_155236610.1	s75549.1	19415.4	375
S1653		126	3	OAR3_152741614.1	OAR3_163942175.1	11200.6	212
S1653		702	3	OAR3_151694139.1	s22496.1	2159.1	45
S1653		507	3	OAR3_151463131.1	s47510.1	2199.78	44
S1653	CON	3	3	OAR3_152741614.1	s47510.1	921.292	16
S1653	UNION	3	3	OAR3_151463131.1	OAR3_163942175.1	12479	240
S1654		152	3	OAR3_134370389.1	OAR3_137497257.1	3126.87	67
S1654		79	3	OAR3_136528538.1	DU373896_534.1	2936.22	55
S1654		616	3	OAR3_137215234.1	s39414.1	5855.2	106
S1654	CON	3	3	OAR3_137215234.1	OAR3_137497257.1	282.023	5
S1654	UNION	3	3	OAR3_134370389.1	s39414.1	8700.05	168
S1657		79	3	OAR3_136528538.1	DU373896_534.1	2936.22	55
S1657		616	3	OAR3_134547477.1	OAR3_136948456.1	2400.98	51

S1657		152	3	OAR3_134370389.1	OAR3_137497257.1	3126.87	67
S1657	CON	3	3	OAR3_136528538.1	OAR3_136948456.1	419.918	10
S1657	UNION	3	3	OAR3_134370389.1	DU373896_534.1	5094.37	101
S1661		90	3	OAR3_129044189.1	OAR3_134747538.1	5703.35	108
S1661		627	3	OAR3_129958976.1	OAR3_131847250.1	1888.27	35
S1661		606	3	OAR3_130714168.1	OAR3_133664108.1	2949.94	54
S1661	CON	3	3	OAR3_130714168.1	OAR3_131847250.1	1133.08	22
S1661	UNION	3	3	OAR3_129044189.1	OAR3_134747538.1	5703.35	108
S1664		90	3	OAR3_129044189.1	OAR3_134747538.1	5703.35	108
S1664		606	3	OAR3_119895458.1	OAR3_130397300.1	10501.8	199
S1664		627	3	OAR3_129958976.1	OAR3_131847250.1	1888.27	35
S1664	CON	3	3	OAR3_129958976.1	OAR3_130397300.1	438.324	7
S1664	UNION	3	3	OAR3_119895458.1	OAR3_134747538.1	14852.1	282
S1668		90	3	OAR3_129044189.1	OAR3_134747538.1	5703.35	108
S1668		507	3	OAR3_124899752.1	OAR3_129413358.1	4513.61	83
S1668		606	3	OAR3_119895458.1	OAR3_130397300.1	10501.8	199
S1668	CON	3	3	OAR3_129044189.1	OAR3_129413358.1	369.169	7
S1668	UNION	3	3	OAR3_119895458.1	OAR3_134747538.1	14852.1	282
S1671		629	3	OAR3_125694577.1	OAR3_127113616.1	1419.04	32
S1671		606	3	OAR3_119895458.1	OAR3_130397300.1	10501.8	199
S1671		507	3	OAR3_124899752.1	OAR3_129413358.1	4513.61	83
S1671	CON	3	3	OAR3_125694577.1	OAR3_127113616.1	1419.04	32
S1671	UNION	3	3	OAR3_119895458.1	OAR3_130397300.1	10501.8	199
S1676		507	3	OAR3_124899752.1	OAR3_129413358.1	4513.61	83
S1676		627	3	OAR3_122721511.1	OAR3_124899752.1	2178.24	39
S1676		606	3	OAR3_119895458.1	OAR3_130397300.1	10501.8	199
S1676	CON	3	3	OAR3_124899752.1	OAR3_124899752.1	0	1
S1676	UNION	3	3	OAR3_119895458.1	OAR3_130397300.1	10501.8	199
S1677		627	3	OAR3_122721511.1	OAR3_124899752.1	2178.24	39
S1677		702	3	OAR3_120965794.1	OAR3_123473821.1	2508.03	48
S1677		606	3	OAR3_119895458.1	OAR3_130397300.1	10501.8	199
S1677	CON	3	3	OAR3_122721511.1	OAR3_123473821.1	752.31	12
S1677	UNION	3	3	OAR3_119895458.1	OAR3_130397300.1	10501.8	199
S1681		629	3	OAR3_112096089.1	OAR3_121762751.1	9666.66	172
S1681		801	3	s65769.1	s18106.1	5900.44	108
S1681		140	3	OAR3_112035267.1	OAR3_115292618.1	3257.35	39
S1681	CON	3	3	OAR3_112096089.1	s18106.1	847.867	12
S1681	UNION	3	3	s65769.1	OAR3_121762751.1	14719.2	268
S1684		45	3	s67646.1	OAR3_106059938.1	1613.14	37
S1684		40	3	s10346.1	OAR3_104545117_X.1	3555.22	73
S1684		844	3	OAR3_99622529.1	OAR3_104878417_X.1	5255.89	100
S1684	CON	3	3	s67646.1	OAR3_104545117_X.1	98.318	4
S1684	UNION	3	3	OAR3_99622529.1	OAR3_106059938.1	6437.41	125
S1685		40	3	s10346.1	OAR3_104545117_X.1	3555.22	73
S1685		844	3	OAR3_99622529.1	OAR3_104878417_X.1	5255.89	100
S1685		507	3	s19649.1	OAR3_103992674.1	3201.34	65

S1685	CON	3	3	s10346.1	OAR3_103992674.1	3002.77	62
S1685	UNION	3	3	OAR3_99622529.1	OAR3_104878417_X.1	5255.89	100
S1688		140	3	OAR3_84830738_X.1	s18949.1	13571.1	269
S1688		606	3	OAR3_95822218.1	OAR3_97385996.1	1563.78	31
S1688		79	3	OAR3_97340255.1	s66125.1	2133.68	44
S1688	CON	3	3	OAR3_97340255.1	OAR3_97385996.1	45.741	2
S1688	UNION	3	3	OAR3_84830738_X.1	s66125.1	14643.2	289
S1691		140	3	OAR3_84830738_X.1	s18949.1	13571.1	269
S1691		40	3	OAR3_93979359.1	OAR3_97036519.1	3057.16	59
S1691		606	3	OAR3_95822218.1	OAR3_97385996.1	1563.78	31
S1691	CON	3	3	OAR3_95822218.1	OAR3_97036519.1	1214.3	23
S1691	UNION	3	3	OAR3_84830738_X.1	s18949.1	13571.1	269
S1692		79	3	s73877.1	OAR3_84522223.1	2064.17	49
S1692		844	3	s55986.1	s04306.1	2324.82	51
S1692		507	3	OAR3_77718162.1	OAR3_82856188.1	5138.03	112
S1692	CON	3	3	s73877.1	OAR3_82856188.1	398.134	10
S1692	UNION	3	3	OAR3_77718162.1	OAR3_84522223.1	6804.06	151
S1695		606	3	OAR3_77361732.1	OAR3_78678231.1	1316.5	34
S1695		49	3	s53138.1	OAR3_78678231.1	1353.04	35
S1695		702	3	OAR3_75163906.1	OAR3_77576280.1	2412.37	43
S1695	CON	3	3	OAR3_77361732.1	OAR3_77576280.1	214.548	7
S1695	UNION	3	3	OAR3_75163906.1	OAR3_78678231.1	3514.32	70
S1696		702	3	DU259120_464.1	OAR3_59494261.1	59494.3	1111
S1696		507	3	OAR3_43871305.1	OAR3_52722619.1	8851.31	160
S1696		556	3	OAR3_51091030.1	OAR3_53737721.1	2646.69	51
S1696	CON	3	3	OAR3_51091030.1	OAR3_52722619.1	1631.59	31
S1696	UNION	3	3	DU259120_464.1	OAR3_59494261.1	59494.3	1111
S1697		801	3	OAR3_31522579_X.1	OAR3_33093517.1	1570.94	33
S1697		702	3	DU259120_464.1	OAR3_59494261.1	59494.3	1111
S1697		27	3	OAR3_28398665.1	OAR3_32271305.1	3872.64	80
S1697	CON	3	3	OAR3_31522579_X.1	OAR3_32271305.1	748.726	15
S1697	UNION	3	3	DU259120_464.1	OAR3_59494261.1	59494.3	1111
S1698		90	3	s32978.1	OAR3_10168366.1	3091.08	50
S1698		29	3	OAR3_6512841.1	s11633.1	8605.75	132
S1698		702	3	DU259120_464.1	OAR3_59494261.1	59494.3	1111
S1698	CON	3	3	s32978.1	OAR3_10168366.1	3091.08	50
S1698	UNION	3	3	DU259120_464.1	OAR3_59494261.1	59494.3	1111
S1699		27	3	DU259120_464.1	s46170.1	2457.2	36
S1699		45	3	OARUn.2445_26203.1	s25572.1	1688.49	31
S1699		702	3	DU259120_464.1	OAR3_59494261.1	59494.3	1111
S1699	CON	3	3	DU259120_464.1	s25572.1	1688.49	24
S1699	UNION	3	3	OARUn.2445_26203.1	OAR3_59494261.1	59494.3	1118
S1700		152	2	OAR2_231739122.1	OAR2_233585592.1	1846.47	33
S1700		45	2	s55941.1	s46164.1	2104	34
S1700		616	2	OAR2_229781196.1	s00795.1	3081.64	50
S1700	CON	3	2	OAR2_231739122.1	s46164.1	112.707	2

S1700	UNION	3	2	s55941.1	OAR2_233585592.1	3837.76	65
S1703		40	2	OAR2_227627755.1	s54339.1	2648.12	33
S1703		606	2	OAR2_226206105.1	OAR2_228251894.1	2045.79	30
S1703		507	2	OAR2_224687649.1	s08704.1	6568.89	90
S1703	CON	3	2	OAR2_227627755.1	OAR2_228251894.1	624.139	16
S1703	UNION	3	2	OAR2_224687649.1	s08704.1	6568.89	90
S1705		29	2	OAR2_223862860_X.1	OAR2_226636096.1	2773.24	53
S1705		507	2	OAR2_224687649.1	s08704.1	6568.89	90
S1705		606	2	OAR2_226206105.1	OAR2_228251894.1	2045.79	30
S1705	CON	3	2	OAR2_226206105.1	OAR2_226636096.1	429.991	9
S1705	UNION	3	2	OAR2_223862860_X.1	s08704.1	7393.68	106
S1706		29	2	OAR2_223862860_X.1	OAR2_226636096.1	2773.24	53
S1706		140	2	OAR2_221746917.1	OAR2_225636672.1	3889.76	77
S1706		507	2	OAR2_224687649.1	s08704.1	6568.89	90
S1706	CON	3	2	OAR2_224687649.1	OAR2_225636672.1	949.023	16
S1706	UNION	3	2	OAR2_221746917.1	s08704.1	9509.62	151
S1707		627	2	OAR2_217423378.1	OAR2_219603926.1	2180.55	38
S1707		606	2	OAR2_202788294.1	OAR2_217785599.1	14997.3	301
S1707		702	2	OAR2_212387600.1	OAR2_223862860_X.1	11475.3	237
S1707	CON	3	2	OAR2_217423378.1	OAR2_217785599.1	362.221	10
S1707	UNION	3	2	OAR2_202788294.1	OAR2_223862860_X.1	21074.6	418
S1708		606	2	OAR2_202788294.1	OAR2_217785599.1	14997.3	301
S1708		616	2	OAR2_211573792.1	OAR2_213240793.1	1667	32
S1708		702	2	OAR2_212387600.1	OAR2_223862860_X.1	11475.3	237
S1708	CON	3	2	OAR2_212387600.1	OAR2_213240793.1	853.193	19
S1708	UNION	3	2	OAR2_202788294.1	OAR2_223862860_X.1	21074.6	418
S1710		49	2	OAR2_209746295.1	OAR2_210988344.1	1242.05	30
S1710		629	2	OAR2_209681146.1	OAR2_211110090_X.1	1428.94	34
S1710		606	2	OAR2_202788294.1	OAR2_217785599.1	14997.3	301
S1710	CON	3	2	OAR2_209746295.1	OAR2_210988344.1	1242.05	30
S1710	UNION	3	2	OAR2_202788294.1	OAR2_217785599.1	14997.3	301
S1713		606	2	OAR2_202788294.1	OAR2_217785599.1	14997.3	301
S1713		123	2	OAR2_203435142.1	OAR2_206393167.1	2958.03	57
S1713		49	2	s33389.1	OAR2_208766144.1	3724.85	63
S1713	CON	3	2	s33389.1	OAR2_206393167.1	1351.88	25
S1713	UNION	3	2	OAR2_202788294.1	OAR2_217785599.1	14997.3	301
S1721		29	2	s66780.1	OAR2_199372044.1	2067.56	41
S1721		606	2	DU367350_210.1	OAR2_198741802.1	1560.61	31
S1721		140	2	OAR2_191308232.1	OAR2_203356427.1	12048.2	217
S1721	CON	3	2	s66780.1	OAR2_198741802.1	1437.32	29
S1721	UNION	3	2	OAR2_191308232.1	OAR2_203356427.1	12048.2	217
S1725		627	2	OAR2_176575230.1	OAR2_177997527_X.1	1422.3	34
S1725		507	2	OAR2_175831096.1	OAR2_177656132.1	1825.04	37
S1725		29	2	OAR2_177398860.1	OAR2_179267347.1	1868.49	42
S1725	CON	3	2	OAR2_177398860.1	OAR2_177656132.1	257.272	8
S1725	UNION	3	2	OAR2_175831096.1	OAR2_179267347.1	3436.25	71

S1733		507	2	OAR2_175831096.1	OAR2_177656132.1	1825.04	37
S1733		616	2	OAR2_172094342.1	OAR2_175943638.1	3849.3	82
S1733		123	2	OAR2_167959232.1	OAR2_176091475.1	8132.24	171
S1733	CON	3	2	OAR2_175831096.1	OAR2_175943638.1	112.542	3
S1733	UNION	3	2	OAR2_167959232.1	OAR2_177656132.1	9696.9	202
S1736		123	2	OAR2_167959232.1	OAR2_176091475.1	8132.24	171
S1736		507	2	OAR2_167852063.1	OAR2_173821801.1	5969.74	125
S1736		629	2	OAR2_166452564.1	OAR2_168122790.1	1670.23	31
S1736	CON	3	2	OAR2_167959232.1	OAR2_168122790.1	163.558	4
S1736	UNION	3	2	OAR2_166452564.1	OAR2_176091475.1	9638.91	198
S1745		29	2	s49391.1	OAR2_141900838.1	2573.2	56
S1745		829	2	OAR2_136846566.1	OAR2_139588207.1	2741.64	53
S1745		702	2	s54108.1	OAR2_162167662.1	23623.2	482
S1745	CON	3	2	s49391.1	OAR2_139588207.1	260.572	5
S1745	UNION	3	2	OAR2_136846566.1	OAR2_162167662.1	25321.1	515
S1748		79	2	OAR2_106891652.1	s60168.1	4096.59	85
S1748		126	2	OAR2_103114233.1	OAR2_110709711.1	7595.48	145
S1748		157	2	OAR2_108603499.1	s07815.1	2262.86	45
S1748	CON	3	2	OAR2_108603499.1	OAR2_110709711.1	2106.21	41
S1748	UNION	3	2	OAR2_103114233.1	s60168.1	7874.01	151
S1749		606	2	s14873.1	OAR2_96066817.1	6724.02	118
S1749		507	2	OAR2_87914191.1	OAR2_89841590.1	1927.4	35
S1749		29	2	OAR2_86182877.1	s14873.1	3159.92	65
S1749	CON	3	2	s14873.1	s14873.1	0	1
S1749	UNION	3	2	OAR2_86182877.1	OAR2_96066817.1	9883.94	182
S1750		606	2	OAR2_56768579.1	OAR2_61008309.1	4239.73	80
S1750		801	2	s20468.1	OAR2_60156510.1	3907.53	77
S1750		616	2	s49388.1	s53985.1	2568.69	49
S1750	CON	3	2	OAR2_56768579.1	s53985.1	911.377	16
S1750	UNION	3	2	s49388.1	OAR2_61008309.1	5897.04	113
S1753		45	2	OAR2_17890079.1	s50931.1	32810.2	566
S1753		49	2	OAR2_33558399.1	OAR2_36125921.1	2567.52	49
S1753		152	2	OAR2_34308093.1	OAR2_36149474.1	1841.38	37
S1753	CON	3	2	OAR2_34308093.1	OAR2_36125921.1	1817.83	36
S1753	UNION	3	2	OAR2_17890079.1	s50931.1	32810.2	566
S1754		627	1	OAR1_273879646.1	OAR1_276539228.1	2659.58	46
S1754		629	1	OAR1_273879646.1	OAR1_276539228.1	2659.58	46
S1754		844	1	s06737.1	s06522.1	3462.3	57
S1754	CON	3	1	OAR1_273879646.1	s06522.1	2133.22	38
S1754	UNION	3	1	s06737.1	OAR1_276539228.1	3988.66	65
S1755		507	1	OAR1_249756050.1	OAR1_252458792.1	2702.74	32
S1755		126	1	OAR1_244520631.1	OAR1_250407027.1	5886.4	110
S1755		40	1	s00653.1	OAR1_252263073.1	2641.09	32
S1755	CON	3	1	OAR1_249756050.1	OAR1_250407027.1	650.977	11
S1755	UNION	3	1	OAR1_244520631.1	OAR1_252458792.1	7938.16	131

S1756		801	1	OAR1_243458636.1	OAR1_248022836.1	4564.2	88
S1756		702	1	OAR1_244304067.1	OAR1_246585728.1	2281.66	41
S1756		126	1	OAR1_244520631.1	OAR1_250407027.1	5886.4	110
S1756	CON	3	1	OAR1_244520631.1	OAR1_246585728.1	2065.1	37
S1756	UNION	3	1	OAR1_243458636.1	OAR1_250407027.1	6948.39	133
S1757		829	1	s65978.1	OAR1_199615737.1	2744.25	51
S1757		79	1	OAR1_195607285.1	DU464218_590.1	2528.45	41
S1757		507	1	OAR1_194627962.1	OAR1_201343673.1	6715.71	128
S1757	CON	3	1	s65978.1	DU464218_590.1	1264.24	20
S1757	UNION	3	1	OAR1_194627962.1	OAR1_201343673.1	6715.71	128
S1763		627	1	OAR1_177619398.1	OAR1_179537892.1	1918.49	37
S1763		629	1	OAR1_177619398.1	OAR1_179537892.1	1918.49	37
S1763		61	1	OAR1_178320448.1	OAR1_182243205.1	3922.76	64
S1763	CON	3	1	OAR1_178320448.1	OAR1_179537892.1	1217.44	26
S1763	UNION	3	1	OAR1_177619398.1	OAR1_182243205.1	4623.81	75
S1764		702	1	s14189.1	s40751.1	42088.8	712
S1764		844	1	DU295978_729.1	s38341.1	1626.29	33
S1764		829	1	OAR1_106406464.1	s71474.1	2041.81	38
S1764	CON	3	1	s14189.1	s38341.1	460.645	9
S1764	UNION	3	1	OAR1_106406464.1	s40751.1	43406.5	739
S1766		829	1	OAR1_106406464.1	s71474.1	2041.81	38
S1766		152	1	s58109.1	OAR1_107512651.1	2376.12	34
S1766		844	1	DU295978_729.1	s38341.1	1626.29	33
S1766	CON	3	1	DU295978_729.1	OAR1_107512651.1	954.141	20
S1766	UNION	3	1	s58109.1	s71474.1	3311.74	49
S1775		627	1	s66549.1	OAR1_98252911_X.1	4469.18	83
S1775		629	1	s66549.1	OAR1_98252911_X.1	4469.18	83
S1775		29	1	s13298.1	s72240.1	6757.67	123
S1775	CON	3	1	s13298.1	OAR1_98252911_X.1	4283.53	79
S1775	UNION	3	1	s66549.1	s72240.1	6943.32	127
S1778		126	1	OAR1_57941516.1	OAR1_59376965.1	1435.45	30
S1778		507	1	OAR1_54796405.1	OAR1_60267266.1	5470.86	89
S1778		702	1	OAR1_7717464.1	OAR1_58938560.1	51221.1	934
S1778	CON	3	1	OAR1_57941516.1	OAR1_58938560.1	997.044	21
S1778	UNION	3	1	OAR1_7717464.1	OAR1_60267266.1	52549.8	957
S1783		702	1	OAR1_7717464.1	OAR1_58938560.1	51221.1	934
S1783		123	1	s25087.1	OAR1_52085279.1	3294.22	54
S1783		126	1	OAR1_51781186.1	s20612.1	1812.25	34
S1783	CON	3	1	OAR1_51781186.1	OAR1_52085279.1	304.093	8
S1783	UNION	3	1	OAR1_7717464.1	OAR1_58938560.1	51221.1	934
S1787		629	1	s06710.1	OAR1_41607999.1	1701.38	41
S1787		627	1	OAR1_39162234_X.1	OAR1_41607999.1	2445.76	59
S1787		702	1	OAR1_7717464.1	OAR1_58938560.1	51221.1	934
S1787	CON	3	1	s06710.1	OAR1_41607999.1	1701.38	41
S1787	UNION	3	1	OAR1_7717464.1	OAR1_58938560.1	51221.1	934
S1791		702	1	OAR1_7717464.1	OAR1_58938560.1	51221.1	934

S1791		45	1	s60471.1	s62110.1	1480.32	30
S1791		79	1	s73231.1	OAR1_16795469.1	3934.26	80
S1791	CON	3	1	s73231.1	s62110.1	246.549	6
S1791	UNION	3	1	OAR1_7717464.1	OAR1_58938560.1	51221.1	934
S1801		45	1	s60471.1	s62110.1	1480.32	30
S1801		801	1	OAR1_10454967.1	s64523.1	2104.16	46
S1801		702	1	OAR1_7717464.1	OAR1_58938560.1	51221.1	934
S1801	CON	3	1	s60471.1	s64523.1	931.683	19
S1801	UNION	3	1	OAR1_7717464.1	OAR1_58938560.1	51221.1	934
S1808		702	1	OAR1_7717464.1	OAR1_58938560.1	51221.1	934
S1808		49	1	s52639.1	OAR1_11087132.1	1952.96	41
S1808		801	1	OAR1_10454967.1	s64523.1	2104.16	46
S1808	CON	3	1	OAR1_10454967.1	OAR1_11087132.1	632.165	16
S1808	UNION	3	1	OAR1_7717464.1	OAR1_58938560.1	51221.1	934
S1809		606	1	s65509.1	OAR1_9881817.1	5706.07	114
S1809		702	1	OAR1_7717464.1	OAR1_58938560.1	51221.1	934
S1809		49	1	s52639.1	OAR1_11087132.1	1952.96	41
S1809	CON	3	1	s52639.1	OAR1_9881817.1	747.645	15
S1809	UNION	3	1	s65509.1	OAR1_58938560.1	54762.8	1003
S1825		606	1	s65509.1	OAR1_9881817.1	5706.07	114
S1825		61	1	OAR1_4303667.1	s49872.1	3986.89	79
S1825		702	1	OAR1_7717464.1	OAR1_58938560.1	51221.1	934
S1825	CON	3	1	OAR1_7717464.1	s49872.1	573.092	13
S1825	UNION	3	1	s65509.1	OAR1_58938560.1	54762.8	1003
S1829		152	1	OAR1_2921089.1	s05524.1	1595.06	31
S1829		90	1	OAR1_2921089.1	s05524.1	1595.06	31
S1829		507	1	s16921.1	OAR1_2921089.1	2439.63	48
S1829	CON	3	1	OAR1_2921089.1	OAR1_2921089.1	0	1
S1829	UNION	3	1	s16921.1	s05524.1	4034.69	78
S1838		123	26	OAR26_31513764.1	s26909.1	2564.63	43
S1838		29	26	OAR26_26341151.1	OAR26_31513764.1	5172.61	112
S1838	CON	2	26	OAR26_31513764.1	OAR26_31513764.1	0	1
S1838	UNION	2	26	OAR26_26341151.1	s26909.1	7737.25	154
S1839		702	26	s61801.1	OAR26_8526649.1	4644.93	52
S1839		90	26	OAR26_6073159.1	OAR26_10130406.1	4057.25	46
S1839	CON	2	26	OAR26_6073159.1	OAR26_8526649.1	2453.49	15
S1839	UNION	2	26	s61801.1	OAR26_10130406.1	6248.69	83
S1840		702	26	s61801.1	OAR26_8526649.1	4644.93	52
S1840		152	26	s69000.1	s37421.1	2783.02	44
S1840	CON	2	26	s61801.1	s37421.1	212.698	4
S1840	UNION	2	26	s69000.1	OAR26_8526649.1	7215.25	92
S1845		152	26	s69000.1	s37421.1	2783.02	44
S1845		79	26	OAR26_222715_X.1	OAR26_2342925.1	2120.21	34
S1845	CON	2	26	s69000.1	OAR26_2342925.1	1031.53	16
S1845	UNION	2	26	OAR26_222715_X.1	s37421.1	3871.7	62

S1846		90	25	s55436.1	s16017.1	1399.6	33
S1846		126	25	s43830.1	OAR25_42361627.1	5972.37	102
S1846	CON	2	25	s55436.1	OAR25_42361627.1	493.581	10
S1846	UNION	2	25	s43830.1	s16017.1	6878.39	125
S1848		126	25	s43830.1	OAR25_42361627.1	5972.37	102
S1848		45	25	OAR25_34706036.1	s00537.1	2304.11	38
S1848	CON	2	25	s43830.1	s00537.1	620.89	5
S1848	UNION	2	25	OAR25_34706036.1	OAR25_42361627.1	7655.59	135
S1855		629	25	s32866.1	OAR25_31259191.1	2423.01	48
S1855		844	25	OAR25_30336172.1	OAR25_34247335.1	3911.16	81
S1855	CON	2	25	OAR25_30336172.1	OAR25_31259191.1	923.019	18
S1855	UNION	2	25	s32866.1	OAR25_34247335.1	5411.15	111
S1856		702	24	s35472.1	OAR24_22201078.1	16855.5	259
S1856		29	24	OAR24_20737428.1	OAR24_24557331.1	3819.9	58
S1856	CON	2	24	OAR24_20737428.1	OAR24_22201078.1	1463.65	22
S1856	UNION	2	24	s35472.1	OAR24_24557331.1	19211.8	295
S1857		702	24	s35472.1	OAR24_22201078.1	16855.5	259
S1857		844	24	s20579.1	OAR24_19275672.1	2066.83	31
S1857	CON	2	24	s20579.1	OAR24_19275672.1	2066.83	31
S1857	UNION	2	24	s35472.1	OAR24_22201078.1	16855.5	259
S1858		123	20	OAR20_46544221.1	OAR20_49453726.1	2909.51	63
S1858		90	20	OAR20_47650422.1	OAR20_48933969.1	1283.55	34
S1858	CON	2	20	OAR20_47650422.1	OAR20_48933969.1	1283.55	34
S1858	UNION	2	20	OAR20_46544221.1	OAR20_49453726.1	2909.51	63
S1859		702	20	OAR20_27119.1	OAR20_14072832.1	14045.7	264
S1859		79	20	OAR20_5451328.1	s10150.1	1705.68	36
S1859	CON	2	20	OAR20_5451328.1	s10150.1	1705.68	36
S1859	UNION	2	20	OAR20_27119.1	OAR20_14072832.1	14045.7	264
S1861		702	20	OAR20_27119.1	OAR20_14072832.1	14045.7	264
S1861		829	20	OAR20_1876702.1	OAR20_3795317.1	1918.62	33
S1861	CON	2	20	OAR20_1876702.1	OAR20_3795317.1	1918.62	33
S1861	UNION	2	20	OAR20_27119.1	OAR20_14072832.1	14045.7	264
S1867		507	19	OAR19_57425358.1	s72187.1	4215.44	84
S1867		140	19	OAR19_59472800.1	s42346.1	2466.57	50
S1867	CON	2	19	OAR19_59472800.1	s72187.1	2168	46
S1867	UNION	2	19	OAR19_57425358.1	s42346.1	4514.01	88
S1875		27	19	s21070.1	OAR19_58095077.1	2593.53	49
S1875		507	19	OAR19_57425358.1	s72187.1	4215.44	84
S1875	CON	2	19	OAR19_57425358.1	OAR19_58095077.1	669.719	17
S1875	UNION	2	19	s21070.1	s72187.1	6139.25	116
S1876		79	19	s55682.1	OAR19_34354181.1	2692.56	49
S1876		49	19	OAR19_21684191.1	s55682.1	9977.43	163
S1876	CON	2	19	s55682.1	s55682.1	0	1
S1876	UNION	2	19	OAR19_21684191.1	OAR19_34354181.1	12670	211

S1877		507	19	s21945.1	s40021.1	5779.07	97
S1877		45	19	OAR19_3773707.1	OAR19_13260250.1	9486.54	172
S1877	CON	2	19	OAR19_3773707.1	s40021.1	2005.36	30
S1877	UNION	2	19	s21945.1	OAR19_13260250.1	13260.2	239
S1880		123	18	s11395.1	s27043.1	2663.63	33
S1880		90	18	s25861.1	s27043.1	5385.42	82
S1880	CON	2	18	s11395.1	s27043.1	2663.63	33
S1880	UNION	2	18	s25861.1	s27043.1	5385.42	82
S1881		629	18	OAR18_61168943.1	OAR18_64788162_X.1	3619.22	69
S1881		829	18	OAR18_56445082.1	s36667.1	4906.48	93
S1881	CON	2	18	OAR18_61168943.1	s36667.1	182.617	6
S1881	UNION	2	18	OAR18_56445082.1	OAR18_64788162_X.1	8343.08	156
S1882		507	18	s44433.1	OAR18_29143813.1	9489.05	160
S1882		79	18	s67627.1	OAR18_32289792.1	5604.77	96
S1882	CON	2	18	s67627.1	OAR18_29143813.1	2458.79	41
S1882	UNION	2	18	s44433.1	OAR18_32289792.1	12635	215
S1901		702	17	OAR17_36966397.1	OAR17_74039265.1	37072.9	600
S1901		629	17	OAR17_35667529.1	OAR17_37633929.1	1966.4	31
S1901	CON	2	17	OAR17_36966397.1	OAR17_37633929.1	667.532	9
S1901	UNION	2	17	OAR17_35667529.1	OAR17_74039265.1	38371.7	622
S1902		29	17	s32218.1	OAR17_13356947.1	2562.88	39
S1902		126	17	OAR17_10638840.1	OAR17_12428758.1	1789.92	32
S1902	CON	2	17	s32218.1	OAR17_12428758.1	1634.69	28
S1902	UNION	2	17	OAR17_10638840.1	OAR17_13356947.1	2718.11	43
S1940		40	17	s28180.1	OAR17_1899290.1	1899.29	41
S1940		49	17	OAR17_1348956.1	s32327.1	2023.51	33
S1940	CON	2	17	OAR17_1348956.1	OAR17_1899290.1	550.334	12
S1940	UNION	2	17	s28180.1	s32327.1	3372.47	62
S1953		801	16	OAR16_74703566.1	OAR16_77087111.1	2383.55	40
S1953		627	16	OAR16_75395749.1	OAR16_77087111.1	1691.36	30
S1953	CON	2	16	OAR16_75395749.1	OAR16_77087111.1	1691.36	30
S1953	UNION	2	16	OAR16_74703566.1	OAR16_77087111.1	2383.55	40
S1954		702	16	OAR16_63959301.1	s59627.1	11378.9	220
S1954		801	16	OAR16_74703566.1	OAR16_77087111.1	2383.55	40
S1954	CON	2	16	OAR16_74703566.1	s59627.1	634.588	9
S1954	UNION	2	16	OAR16_63959301.1	OAR16_77087111.1	13127.8	251
S1955		40	16	OAR16_31845248.1	OAR16_33763548.1	1918.3	42
S1955		61	16	OAR16_32465649.1	OAR16_50643262_X.1	18177.6	326
S1955	CON	2	16	OAR16_32465649.1	OAR16_33763548.1	1297.9	31
S1955	UNION	2	16	OAR16_31845248.1	OAR16_50643262_X.1	18798	337
S1956		702	16	s52990.1	OAR16_10814090.1	10538	191
S1956		616	16	OAR16_10423797.1	OAR16_12590773.1	2166.98	39
S1956	CON	2	16	OAR16_10423797.1	OAR16_10814090.1	390.293	9
S1956	UNION	2	16	s52990.1	OAR16_12590773.1	12314.7	221

S1965		152	16	s01739.1	OAR16_1141118.1	1141.12	32
S1965		702	16	s52990.1	OAR16_10814090.1	10538	191
S1965	CON	2	16	s52990.1	OAR16_1141118.1	865.063	19
S1965	UNION	2	16	s01739.1	OAR16_10814090.1	10814.1	204
S1973		27	15	OAR15_75169402.1	OAR15_81649572.1	6480.17	139
S1973		829	15	s19862.1	OAR15_82598585.1	1679.87	37
S1973	CON	2	15	s19862.1	OAR15_81649572.1	730.856	18
S1973	UNION	2	15	OAR15_75169402.1	OAR15_82598585.1	7429.18	158
S1976		27	15	OAR15_75169402.1	OAR15_81649572.1	6480.17	139
S1976		629	15	OAR15_77451211.1	s20239.1	2428.37	49
S1976	CON	2	15	OAR15_77451211.1	s20239.1	2428.37	49
S1976	UNION	2	15	OAR15_75169402.1	OAR15_81649572.1	6480.17	139
S1979		27	15	s02392.1	OAR15_69475511.1	1333.85	32
S1979		29	15	OAR15_67681322.1	s08876.1	1591.56	36
S1979	CON	2	15	s02392.1	s08876.1	1131.22	26
S1979	UNION	2	15	OAR15_67681322.1	OAR15_69475511.1	1794.19	42
S1986		126	15	OAR15_66122063.1	s05923.1	1875.42	38
S1986		29	15	OAR15_67681322.1	s08876.1	1591.56	36
S1986	CON	2	15	OAR15_67681322.1	s05923.1	316.164	7
S1986	UNION	2	15	OAR15_66122063.1	s08876.1	3150.82	67
S1999		702	15	OAR15_63768341.1	OAR15_66653722.1	2885.38	65
S1999		126	15	OAR15_66122063.1	s05923.1	1875.42	38
S1999	CON	2	15	OAR15_66122063.1	OAR15_66653722.1	531.659	13
S1999	UNION	2	15	OAR15_63768341.1	s05923.1	4229.15	90
S2007		627	15	s61769.1	OAR15_63068221.1	3484.81	67
S2007		61	15	OAR15_62680174.1	OAR15_65584079.1	2903.91	67
S2007	CON	2	15	OAR15_62680174.1	OAR15_63068221.1	388.047	8
S2007	UNION	2	15	s61769.1	OAR15_65584079.1	6000.67	126
S2008		627	15	s61769.1	OAR15_63068221.1	3484.81	67
S2008		27	15	OAR15_50594253.1	s18566.1	9220.78	110
S2008	CON	2	15	s61769.1	s18566.1	231.623	4
S2008	UNION	2	15	OAR15_50594253.1	OAR15_63068221.1	12474	173
S2009		702	15	s33423.1	s34566.1	24919.4	406
S2009		507	15	OAR15_24033751.1	s73229.1	2732.28	57
S2009	CON	2	15	OAR15_24033751.1	s34566.1	885.657	16
S2009	UNION	2	15	s33423.1	s73229.1	26766	447
S2010		40	14	OAR14_33605988.1	OAR14_38944771.1	5338.78	86
S2010		801	14	OAR14_38675883.1	s10566.1	3947.09	73
S2010	CON	2	14	OAR14_38675883.1	OAR14_38944771.1	268.888	6
S2010	UNION	2	14	OAR14_33605988.1	s10566.1	9016.99	153
S2016		40	14	OAR14_33605988.1	OAR14_38944771.1	5338.78	86
S2016		616	14	s45533.1	OAR14_38144388.1	3606.21	60
S2016	CON	2	14	s45533.1	OAR14_38144388.1	3606.21	60
S2016	UNION	2	14	OAR14_33605988.1	OAR14_38944771.1	5338.78	86

S2023		702	13	s43103.1	s69997.1	32663.3	559
S2023		801	13	s03976.1	OAR13_77841865.1	1709.86	31
S2023	CON	2	13	s03976.1	OAR13_77841865.1	1709.86	31
S2023	UNION	2	13	s43103.1	s69997.1	32663.3	559
S2025		79	13	s27782.1	s05134.1	2127.73	36
S2025		702	13	s43103.1	s69997.1	32663.3	559
S2025	CON	2	13	s43103.1	s05134.1	1016.3	12
S2025	UNION	2	13	s27782.1	s69997.1	33774.8	583
S2026		702	13	s44144.1	OAR13_19332393.1	2130.3	31
S2026		829	13	s12849.1	OAR13_20578061.1	2410.3	38
S2026	CON	2	13	s12849.1	OAR13_19332393.1	1164.63	11
S2026	UNION	2	13	s44144.1	OAR13_20578061.1	3375.97	58
S2027		829	13	s50299.1	OAR13_4181900.1	2114.41	33
S2027		126	13	OAR13_2980585_X.1	s00832.1	9013.02	139
S2027	CON	2	13	OAR13_2980585_X.1	OAR13_4181900.1	1201.31	17
S2027	UNION	2	13	s50299.1	s00832.1	9926.12	155
S2030		829	13	s50299.1	OAR13_4181900.1	2114.41	33
S2030		606	13	s05862.1	s26388.1	2756.91	46
S2030	CON	2	13	s50299.1	s26388.1	689.417	14
S2030	UNION	2	13	s05862.1	OAR13_4181900.1	4181.9	65
S2031		616	12	s64119.1	s72379.1	1671.9	31
S2031		507	12	s13792.1	s57817.1	31188.2	514
S2031	CON	2	12	s13792.1	s72379.1	1671.9	25
S2031	UNION	2	12	s64119.1	s57817.1	31188.2	520
S2032		702	11	OAR11_57135966.1	OAR11_63846580.1	6710.61	109
S2032		629	11	OAR11_63756556.1	s75931.1	3121.75	63
S2032	CON	2	11	OAR11_63756556.1	OAR11_63846580.1	90.024	3
S2032	UNION	2	11	OAR11_57135966.1	s75931.1	9742.34	169
S2035		49	11	OAR11_38296265.1	s43569.1	2252.46	51
S2035		90	11	OAR11_38296265.1	OAR11_41384177.1	3087.91	65
S2035	CON	2	11	OAR11_38296265.1	s43569.1	2252.46	51
S2035	UNION	2	11	OAR11_38296265.1	OAR11_41384177.1	3087.91	65
S2036		702	11	OAR11_17172323.1	OAR11_33359712.1	16187.4	260
S2036		616	11	s48715.1	OAR11_17355206.1	1626.28	30
S2036	CON	2	11	OAR11_17172323.1	OAR11_17355206.1	182.883	3
S2036	UNION	2	11	s48715.1	OAR11_33359712.1	17630.8	287
S2042		606	11	OAR11_13995527.1	s64017.1	2212.32	36
S2042		616	11	s48715.1	OAR11_17355206.1	1626.28	30
S2042	CON	2	11	s48715.1	s64017.1	478.914	9
S2042	UNION	2	11	OAR11_13995527.1	OAR11_17355206.1	3359.68	57
S2055		606	11	OAR11_13995527.1	s64017.1	2212.32	36
S2055		152	11	OAR11_11155896.1	OAR11_14457337.1	3301.44	51
S2055	CON	2	11	OAR11_13995527.1	OAR11_14457337.1	461.81	9
S2055	UNION	2	11	OAR11_11155896.1	s64017.1	5051.95	78

S2056		152	11	OAR11_11155896.1	OAR11_14457337.1	3301.44	51
S2056		140	11	s17757.1	OAR11_13845361.1	6147.79	100
S2056	CON	2	11	OAR11_11155896.1	OAR11_13845361.1	2689.47	42
S2056	UNION	2	11	s17757.1	OAR11_14457337.1	6759.76	109
S2065		79	11	OAR11_7689262.1	OAR11_9760146.1	2070.88	37
S2065		140	11	s17757.1	OAR11_13845361.1	6147.79	100
S2065	CON	2	11	s17757.1	OAR11_9760146.1	2062.57	36
S2065	UNION	2	11	OAR11_7689262.1	OAR11_13845361.1	6156.1	101
S2075		90	11	s36390.1	OAR11_4977042.1	4977.04	75
S2075		627	11	s60426.1	OAR11_5531600.1	5531.6	79
S2075	CON	2	11	s36390.1	OAR11_4977042.1	4977.04	75
S2075	UNION	2	11	s60426.1	OAR11_5531600.1	5531.6	79
S2081		79	10	OAR10_79145098.1	s67060.1	9854.04	210
S2081		45	10	OAR10_88694929.1	s26573.1	3303.51	74
S2081	CON	2	10	OAR10_88694929.1	s67060.1	304.208	8
S2081	UNION	2	10	OAR10_79145098.1	s26573.1	12853.3	276
S2088		152	10	OAR10_56312119.1	OAR10_72914358.1	16602.2	277
S2088		61	10	OAR10_70486969.1	OAR10_71983430.1	1496.46	32
S2088	CON	2	10	OAR10_70486969.1	OAR10_71983430.1	1496.46	32
S2088	UNION	2	10	OAR10_56312119.1	OAR10_72914358.1	16602.2	277
S2089		702	9	OAR9_38323108.1	OAR9_96544082.1	58221	1087
S2089		126	9	OAR9_90292422.1	OAR9_92302903.1	2010.48	35
S2089	CON	2	9	OAR9_90292422.1	OAR9_92302903.1	2010.48	35
S2089	UNION	2	9	OAR9_38323108.1	OAR9_96544082.1	58221	1087
S2104		606	9	s18791.1	s12376.1	1409.01	30
S2104		126	9	DU189362_554.1	OAR9_18382789.1	2963.12	61
S2104	CON	2	9	s18791.1	OAR9_18382789.1	1194.62	25
S2104	UNION	2	9	DU189362_554.1	s12376.1	3177.51	66
S2105		844	9	OAR9_5773857.1	s10011.1	10109	172
S2105		126	9	DU189362_554.1	OAR9_18382789.1	2963.12	61
S2105	CON	2	9	DU189362_554.1	s10011.1	463.149	7
S2105	UNION	2	9	OAR9_5773857.1	OAR9_18382789.1	12608.9	226
S2110		844	9	OAR9_5773857.1	s10011.1	10109	172
S2110		157	9	OAR9_6141526.1	OAR9_8154368.1	2012.84	42
S2110	CON	2	9	OAR9_6141526.1	OAR9_8154368.1	2012.84	42
S2110	UNION	2	9	OAR9_5773857.1	s10011.1	10109	172
S2118		844	9	OAR9_1100990.1	OAR9_2578369.1	1477.38	35
S2118		123	9	OAR9_1203135.1	s25647.1	4723.76	95
S2118	CON	2	9	OAR9_1203135.1	OAR9_2578369.1	1375.23	32
S2118	UNION	2	9	OAR9_1100990.1	s25647.1	4825.9	98
S2119		61	8	OAR8_91007371.1	OAR8_93131673.1	2124.3	53
S2119		152	8	OAR8_89335331.1	OAR8_91007371.1	1672.04	33
S2119	CON	2	8	OAR8_91007371.1	OAR8_91007371.1	0	1
S2119	UNION	2	8	OAR8_89335331.1	OAR8_93131673.1	3796.34	85

S2120		152	8	OAR8_89335331.1	OAR8_91007371.1	1672.04	33
S2120		606	8	OAR8_86919224.1	OAR8_89769401.1	2850.18	48
S2120	CON	2	8	OAR8_89335331.1	OAR8_89769401.1	434.07	9
S2120	UNION	2	8	OAR8_86919224.1	OAR8_91007371.1	4088.15	72
S2121		556	8	OAR8_76403936.1	OAR8_78079638.1	1675.7	31
S2121		49	8	OAR8_77578890.1	s01826.1	8664.11	175
S2121	CON	2	8	OAR8_77578890.1	OAR8_78079638.1	500.748	13
S2121	UNION	2	8	OAR8_76403936.1	s01826.1	9839.07	193
S2122		29	8	s18645.1	OAR8_25237806.1	2587.54	51
S2122		45	8	OAR8_23402353.1	OAR8_27562398.1	4160.05	77
S2122	CON	2	8	OAR8_23402353.1	OAR8_25237806.1	1835.45	35
S2122	UNION	2	8	s18645.1	OAR8_27562398.1	4912.13	93
S2123		627	7	s69307.1	s26632.1	2216.96	41
S2123		829	7	OAR7_14417486.1	s34022.1	1808.19	30
S2123	CON	2	7	OAR7_14417486.1	s26632.1	932.8	16
S2123	UNION	2	7	s69307.1	s34022.1	3092.35	55
S2125		627	7	s69307.1	s26632.1	2216.96	41
S2125		45	7	s23542.1	s45087.1	5861.48	112
S2125	CON	2	7	s69307.1	s45087.1	999.191	20
S2125	UNION	2	7	s23542.1	s26632.1	7079.25	133
S2128		616	6	CZ925803_293.1	OAR6_1493254.1	1493.25	34
S2128		507	6	DU325267_788.1	OAR6_1493254.1	1493.25	33
S2128	CON	2	6	DU325267_788.1	OAR6_1493254.1	1493.25	33
S2128	UNION	2	6	CZ925803_293.1	OAR6_1493254.1	1493.25	34
S2141		90	5	OAR5_114683952_X.1	s35247.1	1658.6	34
S2141		61	5	DU254733_336.1	OAR5_114683952_X.1	4203.15	89
S2141	CON	2	5	OAR5_114683952_X.1	OAR5_114683952_X.1	0	1
S2141	UNION	2	5	DU254733_336.1	s35247.1	5861.74	122
S2158		157	5	OAR5_39158574.1	s68199.1	1599.72	30
S2158		29	5	s73882.1	s71821.1	3780.56	56
S2158	CON	2	5	s73882.1	s68199.1	1401.93	26
S2158	UNION	2	5	OAR5_39158574.1	s71821.1	3978.35	60
S2159		140	5	OAR5_3860366.1	OAR5_6287899.1	2427.53	44
S2159		61	5	s01567.1	s02696.1	1571.18	33
S2159	CON	2	5	OAR5_3860366.1	s02696.1	469.931	11
S2159	UNION	2	5	s01567.1	OAR5_6287899.1	3528.78	66
S2161		79	5	s27919.1	s61513.1	1626.57	33
S2161		61	5	s49055.1	s68430.1	1830.42	39
S2161	CON	2	5	s27919.1	s61513.1	1626.57	33
S2161	UNION	2	5	s49055.1	s68430.1	1830.42	39
S2165		126	4	OAR4_103661998.1	s45195.1	8630.78	167
S2165		507	4	s01174.1	OAR4_103958721.1	2865.96	52
S2165	CON	2	4	OAR4_103661998.1	OAR4_103958721.1	296.723	4
S2165	UNION	2	4	s01174.1	s45195.1	11200	215

S2169		507	4	OAR4_93485963.1	OAR4_100549333.1	7063.37	140
S2169		27	4	OAR4_95602342.1	OAR4_97182894.1	1580.55	33
S2169	CON	2	4	OAR4_95602342.1	OAR4_97182894.1	1580.55	33
S2169	UNION	2	4	OAR4_93485963.1	OAR4_100549333.1	7063.37	140
S2170		507	4	OAR4_93485963.1	OAR4_100549333.1	7063.37	140
S2170		702	4	OAR4_92612139.1	OAR4_94029674.1	1417.54	30
S2170	CON	2	4	OAR4_93485963.1	OAR4_94029674.1	543.711	9
S2170	UNION	2	4	OAR4_92612139.1	OAR4_100549333.1	7937.19	161
S2175		702	4	OAR4_92612139.1	OAR4_94029674.1	1417.54	30
S2175		123	4	OAR4_87201676.1	OAR4_92644037.1	5442.36	118
S2175	CON	2	4	OAR4_92612139.1	OAR4_92644037.1	31.898	2
S2175	UNION	2	4	OAR4_87201676.1	OAR4_94029674.1	6828	146
S2188		627	4	OAR4_31796734.1	OAR4_36438393.1	4641.66	71
S2188		79	4	OAR4_33506082.1	OAR4_37638709.1	4132.63	60
S2188	CON	2	4	OAR4_33506082.1	OAR4_36438393.1	2932.31	39
S2188	UNION	2	4	OAR4_31796734.1	OAR4_37638709.1	5841.98	92
S2215		152	3	s06412.1	CL635750_128.1	2705.55	50
S2215		616	3	OAR3_239536012.1	s35224.1	3234.43	56
S2215	CON	2	3	OAR3_239536012.1	CL635750_128.1	2662.22	49
S2215	UNION	2	3	s06412.1	s35224.1	3277.77	57
S2216		79	3	OAR3_229469681_X.1	s43578.1	2300.26	45
S2216		49	3	s51494.1	s55067.1	1446.29	32
S2216	CON	2	3	s51494.1	s43578.1	1323.88	28
S2216	UNION	2	3	OAR3_229469681_X.1	s55067.1	2422.67	49
S2251		616	3	OAR3_222498151.1	s64031.1	4093.34	42
S2251		29	3	s65742.1	OAR3_229832073.1	4118	90
S2251	CON	2	3	s65742.1	s64031.1	877.426	21
S2251	UNION	2	3	OAR3_222498151.1	OAR3_229832073.1	7333.92	111
S2252		616	3	OAR3_145545612.1	OAR3_147645542.1	2099.93	37
S2252		61	3	s68368.1	s28071.1	2515.12	41
S2252	CON	2	3	OAR3_145545612.1	s28071.1	385.672	8
S2252	UNION	2	3	s68368.1	OAR3_147645542.1	4229.38	70
S2265		140	3	OAR3_84830738_X.1	s18949.1	13571.1	269
S2265		606	3	s14327.1	OAR3_91597532.1	1663.8	34
S2265	CON	2	3	s14327.1	OAR3_91597532.1	1663.8	34
S2265	UNION	2	3	OAR3_84830738_X.1	s18949.1	13571.1	269
S2272		123	3	s26313.1	OAR3_86263758.1	2301.95	49
S2272		79	3	s73877.1	OAR3_84522223.1	2064.17	49
S2272	CON	2	3	s26313.1	OAR3_84522223.1	560.418	15
S2272	UNION	2	3	s73877.1	OAR3_86263758.1	3805.7	83
S2273		157	3	OAR3_70578986.1	OAR3_76536013.1	5957.03	117
S2273		702	3	OAR3_75163906.1	OAR3_77576280.1	2412.37	43
S2273	CON	2	3	OAR3_75163906.1	OAR3_76536013.1	1372.11	28
S2273	UNION	2	3	OAR3_70578986.1	OAR3_77576280.1	6997.29	132

S2281		702	3	DU259120_464.1	OAR3_59494261.1	59494.3	1111
S2281		140	3	OAR3_54716452.1	OAR3_59312021.1	4595.57	77
S2281	CON	2	3	OAR3_54716452.1	OAR3_59312021.1	4595.57	77
S2281	UNION	2	3	DU259120_464.1	OAR3_59494261.1	59494.3	1111
S2282		140	2	OAR2_191308232.1	OAR2_203356427.1	12048.2	217
S2282		606	2	OAR2_202788294.1	OAR2_217785599.1	14997.3	301
S2282	CON	2	2	OAR2_202788294.1	OAR2_203356427.1	568.133	14
S2282	UNION	2	2	OAR2_191308232.1	OAR2_217785599.1	26477.4	504
S2283		140	2	OAR2_191308232.1	OAR2_203356427.1	12048.2	217
S2283		79	2	OAR2_200229728.1	OAR2_202002170.1	1772.44	32
S2283	CON	2	2	OAR2_200229728.1	OAR2_202002170.1	1772.44	32
S2283	UNION	2	2	OAR2_191308232.1	OAR2_203356427.1	12048.2	217
S2284		29	2	OAR2_177398860.1	OAR2_179267347.1	1868.49	42
S2284		702	2	OAR2_178886050.1	s29646.1	4550.66	94
S2284	CON	2	2	OAR2_178886050.1	OAR2_179267347.1	381.297	11
S2284	UNION	2	2	OAR2_177398860.1	s29646.1	6037.85	125
S2297		629	2	OAR2_166452564.1	OAR2_168122790.1	1670.23	31
S2297		152	2	s39150.1	OAR2_166756467.1	5092.89	98
S2297	CON	2	2	OAR2_166452564.1	OAR2_166756467.1	303.903	5
S2297	UNION	2	2	s39150.1	OAR2_168122790.1	6459.22	124
S2306		829	2	OAR2_136846566.1	OAR2_139588207.1	2741.64	53
S2306		126	2	OAR2_136603669.1	s33638.1	1519.62	32
S2306	CON	2	2	OAR2_136846566.1	s33638.1	1276.73	26
S2306	UNION	2	2	OAR2_136603669.1	OAR2_139588207.1	2984.54	59
S2307		126	2	OAR2_103114233.1	OAR2_110709711.1	7595.48	145
S2307		61	2	OAR2_103477474.1	OAR2_105771325.1	2293.85	42
S2307	CON	2	2	OAR2_103477474.1	OAR2_105771325.1	2293.85	42
S2307	UNION	2	2	OAR2_103114233.1	OAR2_110709711.1	7595.48	145
S2310		27	2	OAR2_99492787.1	OAR2_101577512.1	2084.72	39
S2310		29	2	OAR2_99441563.1	OAR2_101087449.1	1645.89	31
S2310	CON	2	2	OAR2_99492787.1	OAR2_101087449.1	1594.66	30
S2310	UNION	2	2	OAR2_99441563.1	OAR2_101577512.1	2135.95	40
S2325		79	2	OAR2_85975965.1	OAR2_87651563.1	1675.6	39
S2325		29	2	OAR2_86182877.1	s14873.1	3159.92	65
S2325	CON	2	2	OAR2_86182877.1	OAR2_87651563.1	1468.69	33
S2325	UNION	2	2	OAR2_85975965.1	s14873.1	3366.83	71
S2346		49	2	OAR2_80768977.1	OAR2_82226321.1	1457.34	33
S2346		606	2	OAR2_74253113.1	OAR2_82226321.1	7973.21	148
S2346	CON	2	2	OAR2_80768977.1	OAR2_82226321.1	1457.34	33
S2346	UNION	2	2	OAR2_74253113.1	OAR2_82226321.1	7973.21	148
S2349		49	2	OAR2_61907594.1	OAR2_74652390.1	12744.8	265
S2349		140	2	OAR2_70498239.1	OAR2_72286121.1	1787.88	34
S2349	CON	2	2	OAR2_70498239.1	OAR2_72286121.1	1787.88	34
S2349	UNION	2	2	OAR2_61907594.1	OAR2_74652390.1	12744.8	265

S2355		49	2	OAR2_61907594.1	OAR2_74652390.1	12744.8	265
S2355		140	2	OAR2_61042748.1	OAR2_62188067.1	1145.32	30
S2355	CON	2	2	OAR2_61907594.1	OAR2_62188067.1	280.473	9
S2355	UNION	2	2	OAR2_61042748.1	OAR2_74652390.1	13609.6	286
S2360		29	2	s05611.1	OAR2_55308142.1	7693.97	128
S2360		616	2	s49388.1	s53985.1	2568.69	49
S2360	CON	2	2	s49388.1	OAR2_55308142.1	196.876	5
S2360	UNION	2	2	s05611.1	s53985.1	10065.8	172
S2390		45	2	OAR2_17890079.1	s50931.1	32810.2	566
S2390		79	2	DU469454_586.1	s67704.1	3578.21	58
S2390	CON	2	2	DU469454_586.1	s67704.1	3578.21	58
S2390	UNION	2	2	OAR2_17890079.1	s50931.1	32810.2	566
S2391		123	2	s60459.1	OAR2_7255676.1	1801.8	34
S2391		616	2	OAR2_5168646.1	OAR2_7255676.1	2087.03	37
S2391	CON	2	2	s60459.1	OAR2_7255676.1	1801.8	34
S2391	UNION	2	2	OAR2_5168646.1	OAR2_7255676.1	2087.03	37
S2398		801	1	s09883.1	s73870.1	6372.31	95
S2398		844	1	s06737.1	s06522.1	3462.3	57
S2398	CON	2	1	s06737.1	s73870.1	680.544	12
S2398	UNION	2	1	s09883.1	s06522.1	9154.06	140
S2405		152	1	OAR1_262561308.1	OAR1_266535457.1	3974.15	71
S2405		507	1	OAR1_266243405.1	OAR1_270405323.1	4161.92	75
S2405	CON	2	1	OAR1_266243405.1	OAR1_266535457.1	292.052	6
S2405	UNION	2	1	OAR1_262561308.1	OAR1_270405323.1	7844.02	140
S2412		61	1	OAR1_178320448.1	OAR1_182243205.1	3922.76	64
S2412		79	1	OAR1_182020254.1	OAR1_185615587.1	3595.33	73
S2412	CON	2	1	OAR1_182020254.1	OAR1_182243205.1	222.951	4
S2412	UNION	2	1	OAR1_178320448.1	OAR1_185615587.1	7295.14	133
S2417		152	1	s58109.1	OAR1_107512651.1	2376.12	34
S2417		606	1	OAR1_103790218.1	s53969.1	2417.3	31
S2417	CON	2	1	s58109.1	s53969.1	1070.98	7
S2417	UNION	2	1	OAR1_103790218.1	OAR1_107512651.1	3722.43	58
S2418		29	1	s13298.1	s72240.1	6757.67	123
S2418		40	1	OAR1_98760723.1	OAR1_100874172.1	2113.45	39
S2418	CON	2	1	OAR1_98760723.1	s72240.1	1966.32	36
S2418	UNION	2	1	s13298.1	OAR1_100874172.1	6904.8	126
S2423		507	1	OAR1_81699780.1	OAR1_83030838.1	1331.06	32
S2423		49	1	s55899.1	OAR1_83030838.1	2911.73	67
S2423	CON	2	1	OAR1_81699780.1	OAR1_83030838.1	1331.06	32
S2423	UNION	2	1	s55899.1	OAR1_83030838.1	2911.73	67
S2424		507	1	OAR1_77772595.1	OAR1_80338579.1	2565.98	53
S2424		49	1	s55899.1	OAR1_83030838.1	2911.73	67
S2424	CON	2	1	s55899.1	OAR1_80338579.1	219.467	6
S2424	UNION	2	1	OAR1_77772595.1	OAR1_83030838.1	5258.24	114
