# Supplementary material for: Microphthalmia in Texel Sheep Is Associated with a Missense Mutation in the Paired-Like Homeodomain 3 (PITX3) Gene
Source: PLoS One. 2010 Jan 13;5(1):e8689. doi: 10.1371/journal.pone.0008689 (PMC2805710; doi:10.1371/journal.pone.0008689)
Supplement: Table S2 — Microsatellites. (0.01 MB PDF) [file pone.0008689.s004.pdf]

Table S2. Microsatellites.

	Genomic position		Cases			Controls		
**Marker**	**Virtual Sheep GenomeBrowser v2 [Mb]**	**SheepMap v4.7 [cM]**	**n**	**No. of alleles**	**Obs. het. [%]**	**n**	**No. of alleles**	**Obs. het. [%]**
D9M1722	24.197	34.5	134	4	0.20	212	8	0.84
INRA81	24.901	35.6	134	4	0.03	212	21	0.90
BMS332	28.519	36.9	134	7	0.52	212	12	0.92
