# Supplementary material for: Microphthalmia in Texel Sheep Is Associated with a Missense Mutation in the Paired-Like Homeodomain 3 (PITX3) Gene
Source: PLoS One. 2010 Jan 13;5(1):e8689. doi: 10.1371/journal.pone.0008689 (PMC2805710; doi:10.1371/journal.pone.0008689)
Supplement: Table S3 — Primer sequences for the amplification of ovine PITX3 gene. (0.02 MB PDF) [file pone.0008689.s005.pdf]

Table S3. Primer sequences for the amplification of ovine *PITX3.*

	Exon			Coding sequence			Forward primer		Reverse primer		PCR product
PITX3	**Start***	**End***	**Length**	**Start***	**End***	**Length**	**Start***	**Sequence (5´-3´)**	**Start***	**Sequence (5´-3´)**	**size (bp)**
5'UTR							19'854	AGGCAGCTTAAGGGAGCAAT	20'576	TGGGCTTCTTGCTCTCAGAT	723
5'UTR							20'441	GGGAAGCCCCTGGATACTCT	21'392	AAGGTGGAAAATGGCTCAGA	952
5'UTR							21'242	CTCCTCAGTAGGTTGGTGTACCT	22'084	GCTTCGTGGAGAGTTTGGAG	843
5'UTR							21'831	GCCTTGACAAGTCTGGGAGA	22'767	CAGCGCCCATTCACTTTATG	937
exon 1	22'798	22'959	162				22'653	ACAGCTGGAAAGGGTGGAG	23'673	GGACATTGTGTCCGCTTTG	1021
intron 1							23'475	TTGTGAGAACCCGACAGCGA	24'265	AAAGGGCTGCAGTTTTCTCT	791
intron 1							24'110	TGACAGCTTCAGTTCCCTCA	24'546	TAACTGGGTTCTGGTTAAAG	437
exon 1A	24'467	24'586	120				24'382	GATCCAACACAGGACCCAGA	24'766	GCCAGTGATAAATGAACTTT	385
intron 1							24'635	TGCAGCATTATTCAGCAATG	25'612	AATGCCAGGAATGACCATTC	978
intron 1							25'493	AGGAGTGGCAAGAGCTCAG	26'536	ATGGGCCCAGTGTAGCCTGC	1044
intron 1							26'427	AGTGACTCCCACTGCTTGGA	27'379	GAGAATGTAGCTGCTCTGGA	953
intron 1							27'221	AATATGAACCCCCACCCTTC	28'086	GTAAGTGGCCAAGGCACAAG	866
intron 1							27'907	TTTCAGGTTCCATCCTGTTT	28'773	GCTAGGCAGGCAACAGAGAG	867
intron 1							28'642	TTAATCCTCAGCCAGCCTGT	29'500	GCTGAGGTGGGGAGAGTATG	859
intron 1							29'387	GGTATACTGGATTACTTTCC	29'964	CAGCATTCCATACCTGTGTC	578
intron 1							29'848	CACAGCCTGAACTCTGCTTT	30'306	GTCAGAGGCTTAGGAGTAGG	459
intron 1							30'155	TGAAGAGATTTCAGAGCACC	31'060	GTCTGCTTCTGTACATTGCA	906
intron 1							30'941	GCATGGGGAGTAATGGTCAG	31'918	CAGGTTATCTGGTTCCCAGT	978
exon 2	31'966	32'099	134	31'982	32'099	118	31'795	CCACTTAGGCTGCTGTTTCC	32'248	ACATCCTTGCCTGAGCAGAT	454
exon 3	32'278	32'480	203	32'278	32'480	203	32'121	GGTGGGGGAAATAAAAGCTC	32'708	TCTAGGGCGAGAGAGTGGAG	588
exon 4	32'985	33'907	923	32'985	33'572	588	32'791	GATACTGTCACCCCCGACTC	33'802	GGTCCCTATTCCTGGCCTTA	1012
3'UTR							33'629	GACTGGGGTCGTTTTGACTG	34'500	GTTTTCAGGGTTTGGGGATT	872

*position on EMBL accession FN432136
