# Supplementary material for: Microphthalmia in Texel Sheep Is Associated with a Missense Mutation in the Paired-Like Homeodomain 3 (PITX3) Gene
Source: PLoS One. 2010 Jan 13;5(1):e8689. doi: 10.1371/journal.pone.0008689 (PMC2805710; doi:10.1371/journal.pone.0008689)
Supplement: Table S4 — Primer sequences for the amplification of ovine PITX3 and PAX2 cDNA. (0.01 MB PDF) [file pone.0008689.s006.pdf]

Table S4. Primer sequences for the amplification of ovine *PITX3 and* PAX2 cDNA.

	PITX3 cDNA position		**Localization within ovine *PITX 3***	**Forward primer sequence (5´-3´)**	**Reverse primer sequence (5´-3´)**	**PCR product size (bp)**
	Start	End				
5' RACE outer primer	534		exon 4		GTTGACCGAGTTGAAGGCGAAC	
5' RACE inner primer		183	exon 3		CTTTTTCTTCAGCGAGCCGTCTT	
RT-PCR	-203	233 after Stop	exon 1 / 3'UTR	CCTGCTTGCCTTCCAGACTGC	ACTGGTCCCTATTCCTGGCCTTAGT	1345/1225

	PAX2 cDNA position		**Localization within ovine *PAX2***	**Forward primer sequence (5´-3´)**	**Reverse primer sequence (5´-3´)**	**PCR product size (bp)**
	Start	End				
RT-PCR	-88	30 after Stop	5'UTR / 3'UTR	CCCTCCCTTTTCTCCTCAAG	GCCTGAAGCTTGATGTGGTC	1303
