# Supplementary material for: Microphthalmia in Texel Sheep Is Associated with a Missense Mutation in the Paired-Like Homeodomain 3 (PITX3) Gene
Source: PLoS One. 2010 Jan 13;5(1):e8689. doi: 10.1371/journal.pone.0008689 (PMC2805710; doi:10.1371/journal.pone.0008689)

A

```
PITX3              ---MEFGLLSEAEARSPALSLSDAG------------------------- 22
PITX2 isoform a    -------METNCRKLVSACVQL---------------------------- 15
PITX2 isoform b    -------METNCRKLVSACVQLGVQPAAVECLFSKDSEIKKVEFTDSPES 43
PITX2 isoform c    MNCMKGPLHLEHRAAGTKLSAVSSSSCHHPQPLAMASVLAPGQPRSLDSS 50
                          :  : .   .

PITX3              ----TPHPQLPEHGCKGQEHSDSEKASASLPGGSPEDGSLKKKQRRQRTH 68
PITX2 isoform a    ------------------EKDKSQQGKNEDVG--AEDPSKKKRQRRQRTH 45
PITX2 isoform b    RKEAASSKFFPRQHPGANEKDKSQQGKNEDVG--AEDPSKKKRQRRQRTH 91
PITX2 isoform c    KHRLEVHTISDTSSPEAAEKDKSQQGKNEDVG--AEDPSKKKRQRRQRTH 98
                                     *:..*::.. .  *  .** * **:*******

PITX3              FTSQQLQELEATFQRNRYPDMSTREEIAVWTNLTEARVRVWFKNRRAKWR 118
PITX2 isoform a    FTSQQLQELEATFQRNRYPDMSTREEIAVWTNLTEARVRVWFKNRRAKWR 95
PITX2 isoform b    FTSQQLQELEATFQRNRYPDMSTREEIAVWTNLTEARVRVWFKNRRAKWR 141
PITX2 isoform c    FTSQQLQELEATFQRNRYPDMSTREEIAVWTNLTEARVRVWFKNRRAKWR 148
                   **************************************************

PITX3              KRERSQQAELCKGSFAAPLGGLVPPYEEVYPGYSYGNWPPKALAPP-LAA 167
PITX2 isoform a    KRERNQQAELCKNGFGPQFNGLMQPYDDMYPGYSYNNWAAKGLTSASLST 145
PITX2 isoform b    KRERNQQAELCKNGFGPQFNGLMQPYDDMYPGYSYNNWAAKGLTSASLST 191
PITX2 isoform c    KRERNQQAELCKNGFGPQFNGLMQPYDDMYPGYSYNNWAAKGLTSASLST 198
                   ****.*******..*.. :.**: **:::******.**..*.*:.. *::

PITX3              KTFPFAFNSVNVGPLASQPVFSPPSSIAASMVPSAAAAPGTVPG-PGALQ 216
PITX2 isoform a    KSFPF-FNSMNVNPLSSQSMFSPPNSIS-SMSMSSSMVPSAVTGVPGSSL 193
PITX2 isoform b    KSFPF-FNSMNVNPLSSQSMFSPPNSIS-SMSMSSSMVPSAVTGVPGSSL 239
PITX2 isoform c    KSFPF-FNSMNVNPLSSQSMFSPPNSIS-SMSMSSSMVPSAVTGVPGSSL 246
                   *:*** ***:**.**:**.:****.**: **  *:: .*.:*.* **:

PITX3              GLGGGPPGLAPAAVSSGAVSCPYASAAAAAAAAASSPYVYRDPCNSSLAS 266
PITX2 isoform a    NSLNNLNNLSSPSLNS-AVPTP-----ACPYAPPTPPYVYRDTCNSSLAS 237
PITX2 isoform b    NSLNNLNNLSSPSLNS-AVPTP-----ACPYAPPTPPYVYRDTCNSSLAS 283
PITX2 isoform c    NSLNNLNNLSSPSLNS-AVPTP-----ACPYAPPTPPYVYRDTCNSSLAS 290
                   .  ..  .*:..::.* **.  *    *.. *..:.******.*******

PITX3              LRLKAKQHASFSYPAVHGPPPAANLSPCQYAVERPV 302
PITX2 isoform a    LRLKAKQHSSFGYASVQN--PASNLSACQYAVDRPV 271
PITX2 isoform b    LRLKAKQHSSFGYASVQN--PASNLSACQYAVDRPV 317
PITX2 isoform c    LRLKAKQHSSFGYASVQN--PASNLSACQYAVDRPV 324
                   ********:**.*.:*:.  **:***.*****:***
```

B PITX2

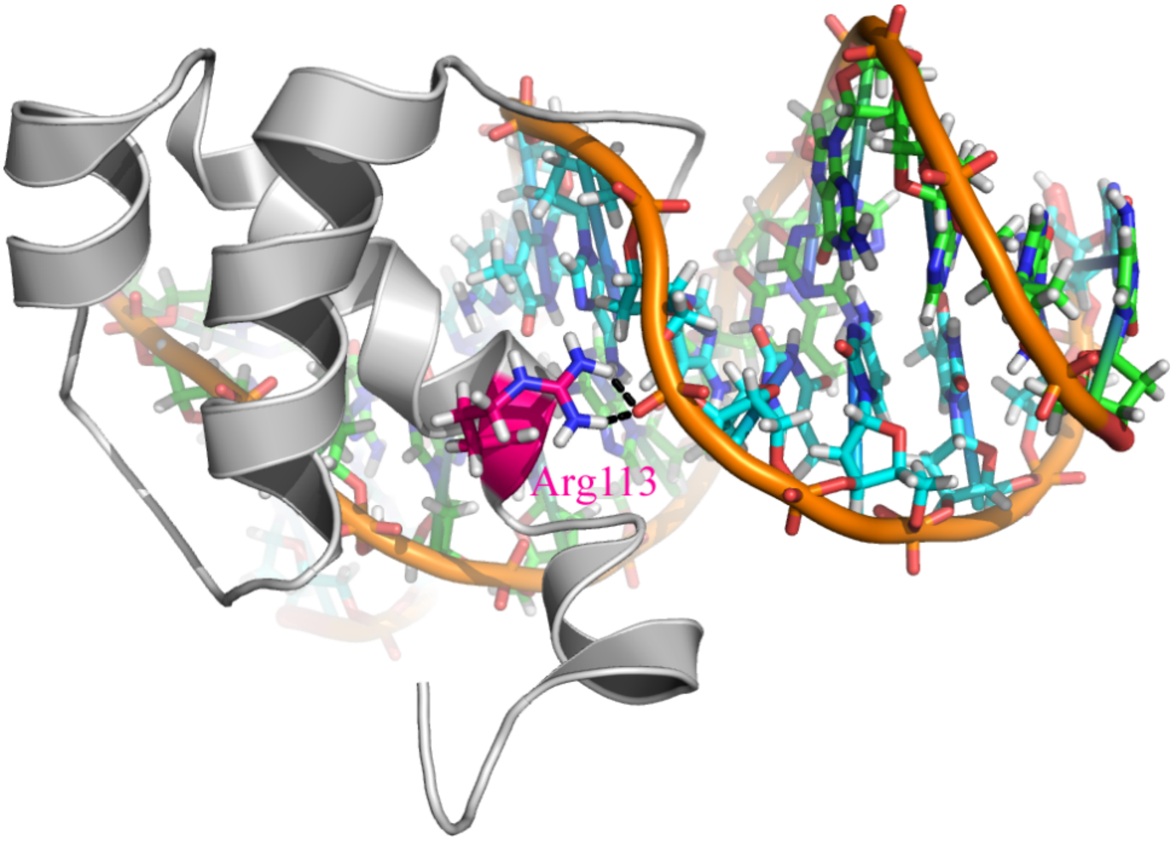

Supplement: Figure S2 — Impact of the microphthalmia associated PITX3 substitution. (A) Alignment of human PITX3 (NP_005020) and human PITX2 isoform a, b, and c (NP_700476; NP_700475; NP_000316) protein sequences. The conserved domains (homeodomain and the C-terminal OAR domain) are indicated in bold face type. The mutated arginine at PITX3 position 113 is shown in red. (B) Structure of the PITX2 homeodomain-DNA complex (Code 1YZ8 taken from PDB [54]). The tertiary structure of the PITX2 homeodomain (96% identical to sheep PITX3 homeodomain) is composed of three alpha helices (shown in light grey). The wildtype PITX3 Arg113 equivalent PITX2 Arg90 (shown with carbon atoms in pink) is located in the third alpha helix and binds with its positively charged capped guanidinium group (shown with blue nitrogen atoms) via two hydrogen bonds (indicated in black) to the DNA backbone (oxygens are shown in red, nitrogen atoms in blue, and carbon atoms in green and light blue). (0.53 MB PDF) [file pone.0008689.s002.pdf]
